# Design, Synthesis and Biological Evaluation of Novel MDH Inhibitors Targeting Tumor Microenvironment

**DOI:** 10.3390/ph16050683

**Published:** 2023-05-02

**Authors:** Sreenivasulu Godesi, Jeong-Ran Han, Jang-Keun Kim, Dong-Ik Kwak, Joohan Lee, Hossam Nada, Minkyoung Kim, Hyun-A Yang, Joo-Young Im, Hyun Seung Ban, Chang Hoon Lee, Yongseok Choi, Misun Won, Kyeong Lee

**Affiliations:** 1 BK21 FOUR Team and Integrated Research Institute for Drug Development, College of Pharmacy, Dongguk University-Seoul, Goyang 10326, Republic of Korea; 2 Personalized Genomic Medicine Research Center, Korea Research Institute of Bioscience and Biotechnology, Daejeon 34141, Republic of Koreaimjy@kribb.re.kr (J.-Y.I.); 3 Biotherapeutics Translational Research Center, KRIBB Korea Research Institute of Bioscience and Biotechnology, Daejeon 34141, Republic of Korea; 4 Department of Biotechnology, Korea University, Seoul 02841, Republic of Korea

**Keywords:** MDH1/2, inhibitors, HIF-1α, lung cancer

## Abstract

MDH1 and MDH2 enzymes play an important role in the survival of lung cancer. In this study, a novel series of dual MDH1/2 inhibitors for lung cancer was rationally designed and synthesized, and their SAR was carefully investigated. Among the tested compounds, compound **50** containing a piperidine ring displayed an improved growth inhibition of A549 and H460 lung cancer cell lines compared with **LW1497**. Compound **50** reduced the total ATP content in A549 cells in a dose-dependent manner; it also significantly suppressed the accumulation of hypoxia-inducible factor 1-alpha (HIF-1α) and the expression of HIF-1α target genes such as *GLUT1* and pyruvate dehydrogenase kinase 1 (*PDK1)* in a dose-dependent manner. Furthermore, compound **50** inhibited HIF-1α-regulated CD73 expression under hypoxia in A549 lung cancer cells. Collectively, these results indicate that compound **50** may pave the way for the development of next-generation dual MDH1/2 inhibitors to target lung cancer.

## 1. Introduction

The rapid growth of cancer cells requires an efficient ATP supply. Cancer cells show alterations in the metabolic pathways that are related to energy production and biosynthetic processes, such as increased uptake of glucose and amino acids and increased breakdown of glutamine and fatty acids. Mutations in *EGFR*, *KRAS*, *MYC*, *PI3K*, *AKT*, *LKB1*, and *p53* are known to be involved in cancer metabolism. Targeting energy metabolism has been suggested as an effective strategy for inhibiting cancer cells [1,2,3,4].

To obtain ATP in the electron transport pathway in cells, the cytosolic NADH must be transported into the mitochondria. Because NADH itself cannot be transported into the mitochondria, cells transport reducing equivalents across the mitochondrial membrane using a malate–aspartate shuttle (MAS). The MAS is operated by two pairs of enzymes, malate dehydrogenases (MDH1 and MDH2) and glutamate oxaloacetate transaminases (GOT1 and GOT2), which are localized in the mitochondria and cytoplasm. MDH1 and MDH2 catalyze the reversible conversion of malate to oxaloacetate (OAA) using the NAD/NADH cofactor system [5,6,7,8,9]. Aminooxyacetic acid (AOA), a specific MAS inhibitor, decreases the proliferation of breast adenocarcinoma cells by suppressing GOT1 and GOT2 [10].

The association of MDH1 and MDH2 have been reported in several cancers such as pancreatic and lung cancers. The expression levels of MDH1 and MDH2 are high in patients with lung cancer [4,10]. High expression of MDH1 is significantly correlated with patient survival, and its knockdown affects cell viability compared with that of MDH2 [10,11]. A previous report presented **LW1497**, a dual inhibitor of MDH1 and MDH2, which showed significant in vivo antitumor effects against colon cancer through the inhibition of mitochondrial respiration and hypoxia-inducible factor-1 alpha (HIF-1α) accumulation [12,13]. Another report showed that **LW1497** downregulates Slug expression to impede the progression of A549 lung cancer cells by inhibiting epithelial-mesenchymal transition [14].

**Figure 1:**
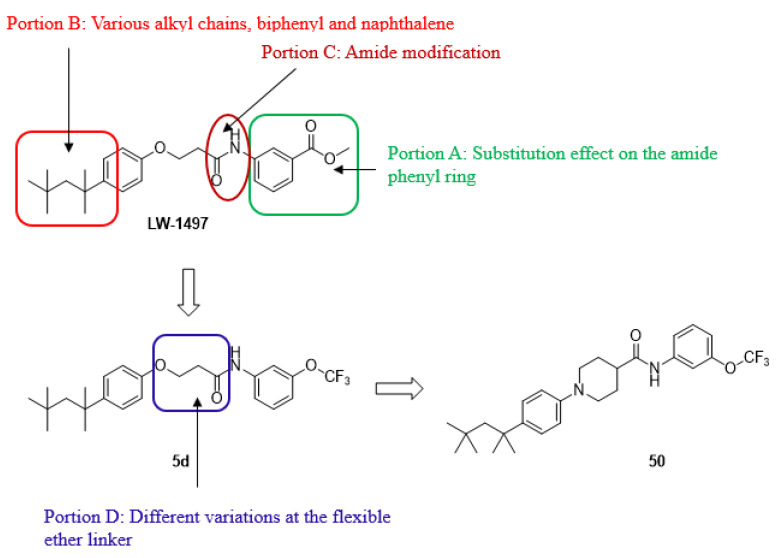
Chemical structure of a known MDH inhibitor and schematic design for SAR exploration.

In this study, to develop a potent dual inhibitor of MDH1/2 for lung cancer, **LW1497** derivatives were synthesized based on their structure–activity relationships (SARs). Finally, we identified compound **50** as a dual inhibitor of MDH1/2 in lung cancer cells (Figure 1).

## 2. Results

### 2.1. Chemistry

**Scheme 1:**
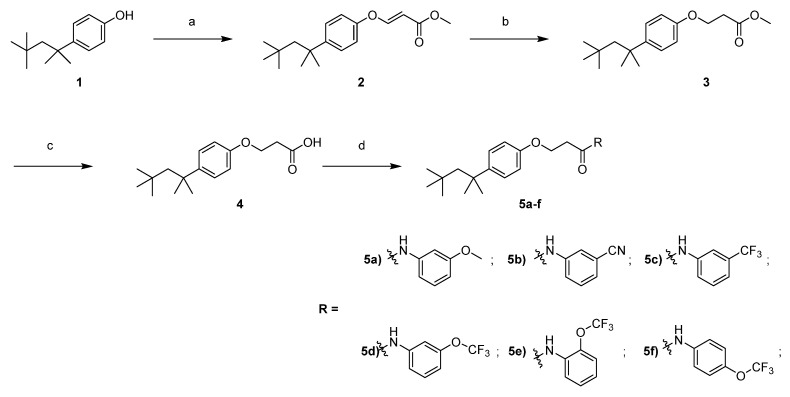
**Synthesis of trimethylpentane derivatives 5a**–**f ^a^**. ^a^ Reagents and conditions: (a) PPh_3_, methyl propiolate, toluene, rt, 12 h, 71%; (b) 10% Pd/C, H_2_ (balloon), EtOAc, rt, 12 h; (c) LiOH, THF, H_2_O, rt, 12 h, 81% over two steps; (d) amines, T3P, DIPEA, THF, rt, 12 h, 11–47%.

The synthesis of the amide compounds **5a**–**f** was completed in four steps, as shown in Scheme 1. The hydroxyl group of phenol **1** was alkylated with methyl propiolate in the presence of triphenylphosphine (PPh_3_) in toluene, and the resulting (*E*)-phenoxyacrylic methyl ester (**2**) was saturated with palladium on carbon (Pd/C) under H_2_ balloon pressure followed by hydrolysis with lithium hydroxide to furnish carboxylic acid **4** at 81% yield [12]. Finally, the T3P-mediated coupling of the resultant carboxylic acid **4** with corresponding amines yielded a series of amide derivatives **5a**–**f**.

**Scheme 2:**
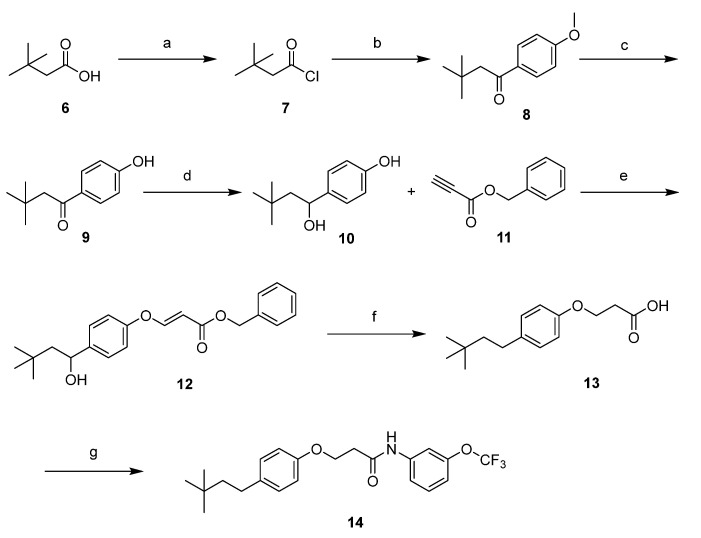
**Synthesis of trimethylpentane-modified derivative 14 ^a^**. ^a^ Reagents and conditions: (a) SOCl_2_, rt, 12 h; (b) anisole, AlCl_3_, DCM, 0 °C to rt, 1 h, 48% over two steps; (c) AlCl_3_, DCE, 75 °C, 16 h, 84%; (d) LiAlH_4_, THF, rt, 12 h, 69%; (e) *N*-methyl morpholine, acetonitrile, rt, 12 h, 73%; (f) 10% Pd/C, H_2_ (balloon), EtOAc, rt, 12 h, 82%; (g) 3-(trifluoromethoxy)aniline, T3P, DIPEA, THF, rt, 12 h, 55%.

Compound **14** was synthesized in seven steps, as illustrated in Scheme 2. Friedel–Crafts acylation of anisole with in situ-generated acid chloride yielded **8** [15], which was then demethylated with aluminum chloride (AlCl_3_) to yield phenol **9**. The reduction of **9** with lithium aluminum hydride (LiAlH_4_) followed by the alkylation of phenol **10** with commercially available benzyl propiolate yielded phenoxy acrylate **12**. Saturation of the phenoxy acrylate portion and debenzylation of **12** were completed using palladium carbon (Pd/C) under H_2_ balloon pressure, yielding **13**. Interestingly, under the palladium hydrogenation conditions, along with double bond reduction and debenzylation, dehydroxylation of **12** also occurred. The T3P-mediated coupling of the resultant carboxylic acid **13** with the corresponding amine yielded **14**.

**Scheme 3:**
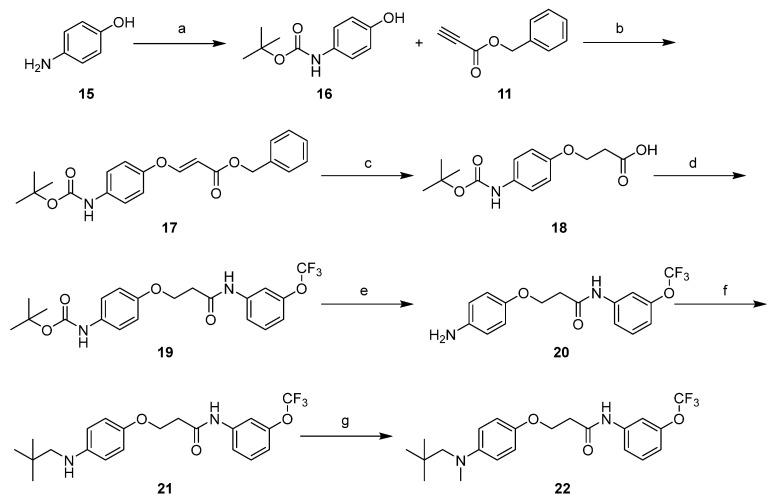
**Synthesis of trimethylpentane-modified derivatives 21 and 22 ^a^**. ^a^ Reagents and conditions: (a) Boc_2_O, Et_3_N, THF, rt, 12 h, 79%; (b) *N*-methyl morpholine, acetonitrile, rt, 12 h, 11%; (c) 10% Pd/C, H_2_ (balloon), EtOAc, rt, 12 h, 69%; (d) 3-(trifluoromethoxy)aniline, T3P, DIPEA, THF, rt, 12 h, 51%; (e) TFA, DCM, rt, 2 h, 99%; (f) pivalaldehyde, NaCNBH_3_, AcOH, DCM, MeOH, rt, 12 h, 56%; (g) paraformaldehyde, NaCNBH_3_, AcOH, DCM, MeOH, rt, 12 h, 63%.

Trimethylpentane-modified derivatives **21** and **22** were synthesized in seven steps, as shown in Scheme 3. 4-Aminophenol (**15**) was treated with Boc anhydride to yield **16,** which was then alkylated with benzyl propiolate to yield **17**. The reduction of the double bond and removal of the benzyl group in **17** using palladium carbon (Pd/C) under H_2_ balloon pressure yielded **18**. The T3P-mediated coupling of the resultant carboxylic acid **18** with 3-(trifluoromethoxy)aniline yielded **19**. The removal of the Boc group in **19** with TFA followed by the reductive amination of **20** with pivalaldehyde yielded **21**. The reductive amination of **21** with paraformaldehyde provided **22**.

**Scheme 4:**
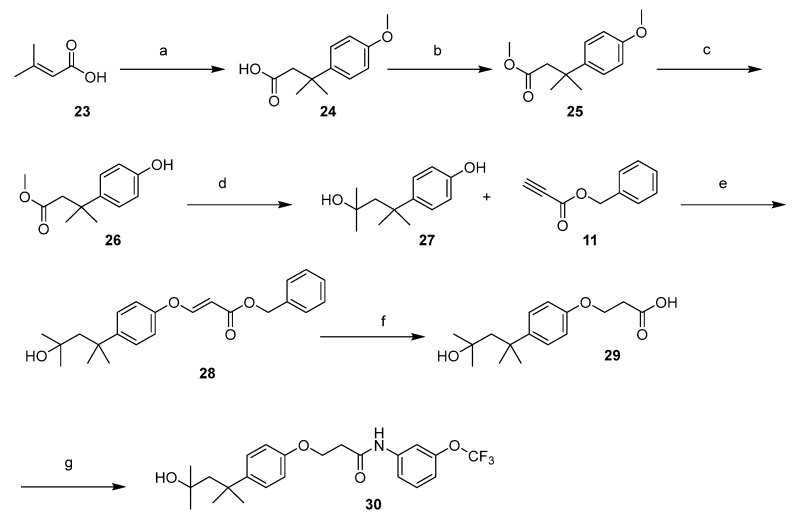
**Synthesis of 3-(4-(4-hydroxy-2,4-dimethylpentan-2-yl)phenoxy)propanamide derivative 30 ^a^**. ^a^ Reagents and conditions: (a) anisole, AlCl_3_, DCM, 65 °C, 2 h, 20%; (b) H_2_SO_4_, MeOH, rt, 12 h, 68%; (c) 1.0 M BBr_3_ in DCM, DCM, rt, 2 h, 66%; (d) 3.0 M CH_3_MgBr in ether, THF, rt, 16 h, 71%; (e) *N*-methyl morpholine, acetonitrile, rt, 12 h, 95%; (f) 10% Pd/C, H_2_ (balloon), EtOAc, rt, 12 h, 83%; (g) 3-(trifluoromethoxy)aniline, T3P, DIPEA, THF, rt, 12 h, 76%.

Compound **30** was synthesized as shown in Scheme 4. Friedel–Crafts alkylation of anisole with 3-methylbut-2-enoic acid (**23**) yielded **24** [16]. The resulting acid **24** was converted to methyl ester **25** and followed by demethylation; then, the Grignard reaction of ester **26** with methyl magnesium bromide yielded the tertiary alcohol **27**. The hydroxy group of phenol **27** was alkylated with benzyl propiolate, followed by the double bond reduction and benzyl ester cleavage of **28** by treatment with palladium carbon (Pd/C) under H_2_ balloon pressure to yield **29**. The T3P-mediated coupling of the resultant carboxylic acid **29** with 3-(trifluoromethoxy)aniline yielded **30**.

**Scheme 5:**
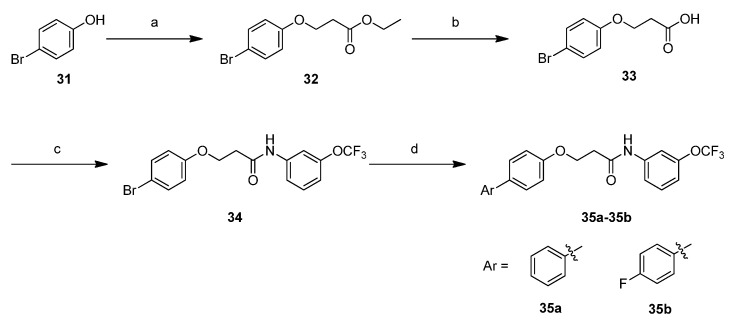
**Synthesis of biphenyloxypropanamide derivatives 35a**–**35b ^a^.** ^a^ Reagents and conditions: (a) DMAP, ethyl acrylate, 100 °C, 3 h, 39%; (b) LiOH, THF, H_2_O, rt, 12 h, 69%; (c) 3-(trifluoromethoxy)aniline, T3P, DIPEA, THF, rt, 12 h, 62%; (d) boronic acids, Pd(PPh_3_)_4_, Cs_2_CO_3_, 1,4-dioxnae, H_2_O, 100 °C, 12 h, 60% for **35a** and 41% for **35b**.

**Scheme 6:**
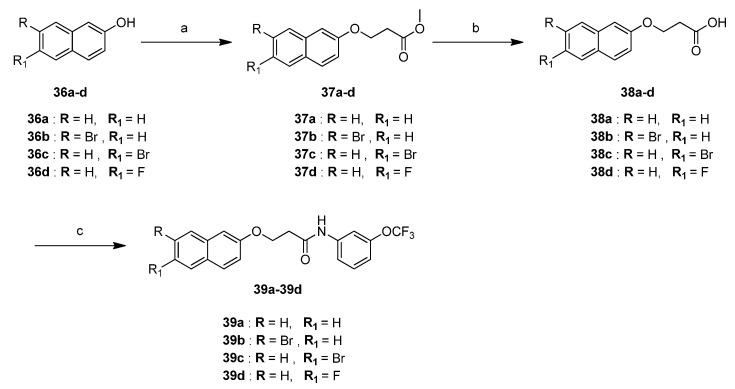
**Synthesis of naphthalene derivatives 39a**–**d ^a^**. ^a^ Reagents and conditions: (a) DMAP, 100 °C, 2 h, 16–38%; (b) LiOH, THF, H_2_O, rt, 12 h, 31–86%; (c) 3-(trifluoromethoxy)aniline, T3P, DIPEA, THF, rt, 12 h 17–27%.

Biphenyl and naphthalene derivatives **35a**–**b** and **39a**–**d** were synthesized as shown in Scheme 5 and Scheme 6. The hydroxy group of phenols **31** and **36a**–**d** was treated with an excess of ethyl and/or methyl acrylate in the presence of DMAP under microwave irradiation to produce methyl and/or ethyl esters **32** and **37a**–**b**. The esters were hydrolyzed with lithium hydroxide, and the resulting acids were coupled with 3-(trifluoromethoxy)aniline using T3P-mediated amide formation to produce **34** and **39a**–**d**. A Suzuki coupling reaction of **34** with corresponding boronic acids in the presence of Pd(PPh_3_)_4_ yielded **35a**–**b**.

**Scheme 7:**
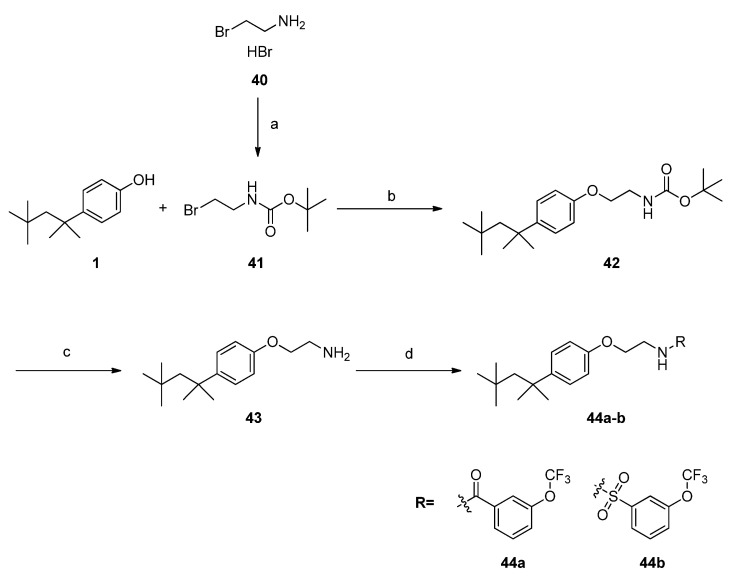
**Synthesis of intermediate 43 and its amide and sulfonamide derivatives 44a**–**b ^a^**. ^a^ Reagents and conditions: (a) Boc_2_O, Et_3_N, DCM, rt, 1 h, 78%; (b) K_2_CO_3_, acetone, 70 °C, 12 h, 47%; (c) TFA, DCM, rt, 12 h, 77%; (d) acid chloride (**44a**) or sulfonyl chloride (**44b**) Et_3_N, DCM, rt, 1 h, 24% for **44a** and 8% for **44b**.

Compounds **44a**–**b** were synthesized in four steps, as illustrated in Scheme 7. The precursor for the synthesis of **42** was obtained by Boc protection of 2-bromoethanamine HBr with Boc anhydride [17]; then, the hydroxy group of phenol **1** was alkylated to yield **42**. Boc was then deprotected using TFA to yield **43**. Subsequently, **43** was treated with acid and/or sulfonyl chloride to obtain compounds **44a**–**b**.

**Scheme 8:**
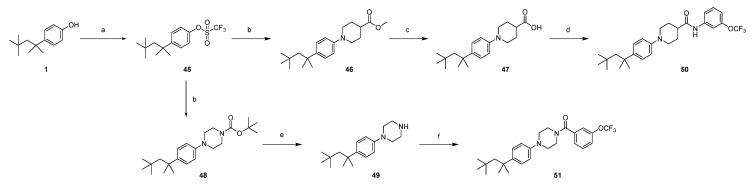
**Synthesis of piperidine, piperazine derivatives 50 and 51 ^a^**. ^a^ Reagents and conditions: (a) trifluoromethanesulfonic anhydride, Et_3_N, DCM, 0 °C, 1 h, 83%; (b) methyl piperidine-4-carboxylate for **46** and *tert*-butyl piperazine-1-carboxylate for **48**, XPhos, Pd_2_(dba)_3_, Cs_2_CO_3_, toluene, 110 °C, 12 h, 59% for **46** and 93% for **48**; (c) LiOH, THF, H_2_O, rt, 6 h, 99%; (d) 3-(trifluoromethoxy)aniline, T3P, DIPEA, THF, rt, 12 h, 59%; (e) TFA, DCM, rt, 12 h, 83%; (f) 3-(trifluoromethoxy)benzoyl chloride, Et_3_N, DCM, rt, 1 h, 36%.

Compounds **50** and **51** were synthesized, as illustrated in Scheme 8. The hydroxyl group of phenol **1** was reacted with trifluoromethanesulfonic anhydride to yield **45** [18]. The Buchwald–Hartwig coupling of **45** with methyl piperidine-4-carboxylate and/or *tert*-butyl piperazine-1-carboxylate yielded **46** and **48**, respectively. The base-mediated hydrolysis of **46** yielded the acid **47**. Amide coupling of **47** with 3-(trifluoromethoxy)aniline yielded compound **50**. The trifluoroacetic-acid-mediated deprotection of the Boc from **48** provided **49**, which was then treated with 3-(trifluoromethoxy)benzoyl chloride in the presence of triethylamine to yield **51**.

**Scheme 9:**
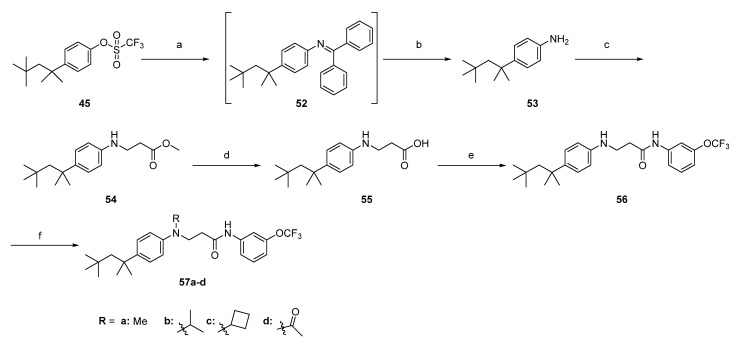
**Synthesis of linker-modified derivatives 56 and 57a**–**d ^a^**. ^a^ Reagents and conditions: (a) diphenylmethanimine, BINAP, Pd(dba)_2_, Cs_2_CO_3_, toluene, 100 °C, 12 h; (b) HCl, MeOH, 80 °C, 6 h, 39% over two steps; (c) NaI, methyl 3-bromopropionate, Cs_2_CO_3_, acetonitrile, 70 °C, 12 h, 35%; (d) LiOH, THF, H_2_O, rt, 12 h, 98%; (e) 3-(trifluoromethoxy)aniline, T3P, DIPEA, THF, rt, 12 h, 28%; (f) **73a–c**: aldehyde or ketone, NaCNBH_3_, AcOH, DCM, MeOH, rt, 12 h, 9–55%; **57d**: acetyl chloride, Et_3_N, DCM, rt, 1 h, 61%.

Compounds **56** and **57a**–**d** were synthesized as shown in Scheme 9. The Buchwald–Hartwig coupling reaction of **45** with benzophenone imine followed by the deprotection of benzophenone yielded **53**. Alkylation of **53** with methyl 3-bromopropionate in acetonitrile yielded **54**. The methyl ester was hydrolyzed using lithium hydroxide to yield **55**, and the resultant acid was coupled with 3-(trifluoromethoxy)aniline to give **56**. The reductive amination of **56** with ketones or aldehydes in the presence of sodium cyanoborohydride (NaBH_3_CN) yielded **57a**–**c**, respectively. Acetyl chloride was reacted with **56** in the presence of triethylamine to yield **57d**.

### 2.2. Biological Evaluation

#### 2.2.1. MDH Enzyme Activity

To develop the dual inhibitors of MDH1 and MDH2, we screened the newly synthesized **LW1497** derivatives using MDH and MDH2 enzyme assays. **LW1497** was used as the positive control for the MDH1/2 dual inhibitor. We evaluated the ability of the synthesized compounds to inhibit human recombinant MDH1 and MDH2 enzymatic activity using an oxaloacetate-dependent NADH oxidation assay (Table 1, Table 2, Table 3, Table 4, Table 5 and Table 6). Our SAR optimization strategy focused on four different parts of **LW1497**: (A) the introduction of various groups at different positions on the amide phenyl ring; (B) the replacement of the 3-(4-(2,4,4-trimethylpentan-2-yl) alkyl chain; (C) the alteration of the amide portion; and (D) the modification of the oxypropanamide linker portion (Figure 1).

As a starting point for the SAR studies, we first examined the substitution of various groups on the amide phenyl ring (Part A). We started with *N*-(3-methoxyphenyl)-3-(4-(2,4,4-trimethylpentan-2-yl)phenoxy)propanamide **5a** having an electron-donating group (OMe) at the meta position, which showed loss of activity in MDH1 and moderate inhibitory activity in MDH2 (IC_50_ = 6.18 ± 0.74 and 1.5 ± 0.01 μM, respectively) (Table 1). The replacement of the -OMe group at the meta position of the phenyl ring with electron-withdrawing groups such as -CN and -CF_3_ (**5b** and **5c**) resulted in a loss of activity. Interestingly, the -OCF_3_ group (**5d**) regained inhibitory activity against both MDH1 and MDH2 enzymes (IC_50_ = 0.94 ± 0.05 and 1.24 ± 0.13 μM, respectively) and showed equivalent potency compared with **LW1497**. However, when the OCF_3_ group was shifted from the meta to the ortho and para positions of the phenyl ring (**5e** and **5f**), both led to an approximately three-fold activity loss against both MDH1 and MDH2 compared with **5d**. These results implied that the -OCF_3_ group at the meta position of the amide phenyl ring was well tolerated (Table 1).

Based on the results for compound **5d**, we focused our SAR exploration on the replacement of the 3-(4-(2,4,4-trimethylpentan-2-yl) side chain (portion B) by fixing the -OCF_3_ group at the meta position on the amide phenyl ring. The 3-(4-(2,4,4-trimethylpentan-2-yl) moiety was replaced with the 4-(3,3-dimethylbutyl) moiety (**14**), which maintained moderate MDH1 and MDH2 inhibition (IC_50_ = 3.38 ± 0.36 and 1.53 ± 0.08 μM, respectively). Further replacement with the amine-linked aliphatic chains (**21** and **22**) resulted in the loss of activity against both MDH1 and MDH2. In addition, one of the methyl groups from terminal trimethyl was replaced with a hydroxy group (**30**), resulting in a decrease in the MDH1 and MDH2 inhibitory activities (IC_50_ = >5 μM). Replacing the portion B fragment from the aliphatic chain to an aromatic ring (**35a**) retained dual activity against both MDH1 and MDH2 (IC_50_ = 0.78 ± 0.08 and 1.38 ± 0.59 μM, respectively) compared with the active compound **5d**, whereas the 4-F substituted phenyl ring (**35b**) led to significant activity loss compared with **35a** (Table 2). However, when we modified the 3-(4-(2,4,4-trimethylpentan-2-yl)phenoxy) moiety with 3-(naphthalen-2-yloxy) moiety (**39a**), loss of activity against both MDH enzymes was noted (IC_50_ = >5 μM). Interestingly, substitutions on the naphthalene ring with electron withdrawing groups, such as 7-bromo (**39b**), 6-bromo (**39c**), and 6-fluoro (**39d**), demonstrated selective inhibitory activity against MDH1 than MDH2 (IC_50_ = 1.61 ± 0.25, 1.61 ± 0.21, 2.25 ± 0.77 and >5 μM, respectively; Table 3). These results suggest that the 4-(3,3-dimethylbutyl)-like alkyl chain moiety is required for the dual inhibitory activity. Replacing the amide linkage of **5d** with the reverse amide linkage (**44a**) maintained moderate inhibitory activity against MDH1 and MDH2 enzymes (IC_50_ = 3.15 ± 0.11, 2.22 ± 0.28 μM, respectively). Switching the amide group from (**44a**) to the sulfonamide group (**44b**) showed moderate activity against MDH1 (IC_50_ = 2.85 ± 0.45 μM), while it lost activity against MDH2 (Table 4).

For the SAR exploration of portion D, we chose to start with compound **5d** by keeping the -OCF_3_ group intact at the meta position on the amide phenyl ring in portion A and (4-(3,3-dimethylbutyl) alkyl chain in portion B. Replacement of the flexible ether-linked aliphatic chain from **5d** with an amine-linked piperidine ring (**50**) exhibited dual inhibitory activity against MDH1 and MDH2 (IC_50_ = 3.33 ± 0.18 and 2.24 ± 0.09 μM, respectively). Compound **51**, which contains a piperazine group, showed decreased dual inhibitory activity (Table 5). Replacing the amine-linked piperidine with a more flexible amine-linked aliphatic chain (**56**) led to an increase in the activity against MDH1 (IC_50_ = 1.79 ± 0.04 μM) but also maintained similar inhibitory activity against MDH2. When the hydrogen atom in **56** was substituted with aliphatic groups, such as methyl (**57a**), isopropyl (**57b**), and cyclobutyl (**57c**), it retained similar potency against both MDH1 and resulted in a two- to three-fold improvement in the inhibitory activity against MDH2. Interestingly, the *N*-acylated derivative (**57d**) was more selective for MDH1 than for MDH2 (IC_50_ = 1.41 ± 0.47 and >5 μM, respectively) (Table 6).

The combined structure optimization and SAR studies resulted in the identification of novel compounds, such as **5d**, **5f**, **14**, **35a**, **44a**, **50**, **56**, **57a**, and **57b,** which showed strong inhibition on the activities of both MDH1 and MDH2. However, compounds **39b**, **39c**, **39d**, and **57d** showed MDH1 activity, whereas **5b** and **5c** only showed MDH2 activity.

#### 2.2.2. Molecular Docking Study for Compound **50**

**Figure 2:**
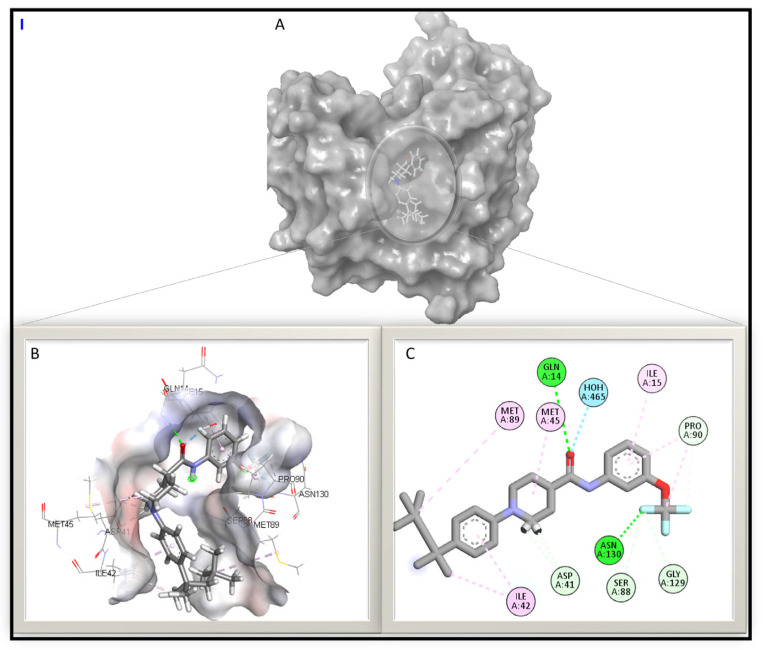
Docked complex of compound **50** with MDH1. (**A**) a 3D model of MDH1 complexed with compound **50**; (**B**) a 3D depiction of the binding cavity of compound **50** complexed with MDH1 (PDB ID: 5MDH); (**C**) a 2D interaction pattern of compound **50** with the MDH1 active site. Favorable interactions are color-coded as follows: green—hydrogen bonds, light blue—halogen interaction, orange—π–charge, dark pink—π–π stacking interactions, purple—π–sigma interactions, light pink—hydrophobic interactions. Residues shown in light green form weak van der Waals interactions.

Molecular docking is a vital component of computer-aided drug design as it offers a range of valuable applications, including the prediction of ligand–target binding modes, the ranking of compounds based on their docking scores, and the correlation of these scores with potential activity [19,20]. Visualizations of the interactions generated through docking studies serves as a guide for optimizing the affinity characteristics of the studied ligands. In this study, compound **50** was subjected to an in-depth molecular examination against both MDH1 and MDH2 to determine its binding patterns. The results showed that compound **50** displayed a docking score of −4.9 Kcal/mol against MDH1, which can be attributed to the number of hydrogen bonds formed by compound **50** against MDH1, as depicted in Figure 2. Compound **50** formed two hydrogen bonds with Gln14 and Asn130, highlighting the importance of forming a hydrogen bond with this amino acid residue for activity.

**Figure 3:**
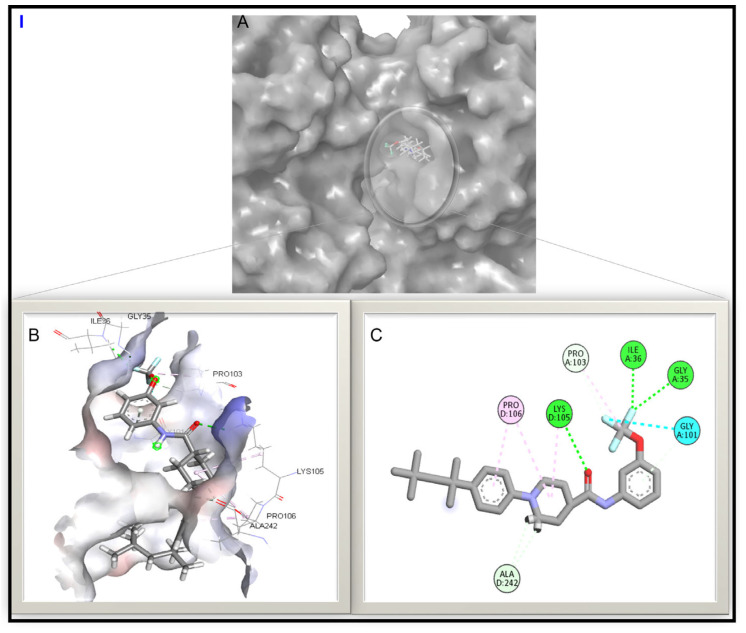
Docked complex of compound **50** with MDH2. (**A**) A 3D model of MDH2 complexed with compound **50**; (**B**) a 3D depiction of the binding cavity of compound **50** complexed with MDH2 (PDB ID: 2DFD); (**C**) a 2D interaction pattern of compound **50** with the MDH2 active site. Favorable interactions are color coded as follows: green—hydrogen bonds, light blue—halogen interaction, orange—π–charge, dark pink—π–π stacking interactions, purple—π–sigma interactions, light pink—hydrophobic interactions. Residues shown in light green form weak van der Waals interactions.

The molecular docking study of compound **50** against MDH2 revealed a docking score of approximately −5.9 Kcal/mol. Compound **50** formed a hydrogen bond with the Gly35 residue within the binding site and two additional hydrogen bonds with the Ile36 and Lys105 residues, as shown in Figure 3.

#### 2.2.3. Growth Inhibition Assay of Lung Cancer Cells

At the same time, we investigated the effect of the compounds on the growth inhibition of A549 and H460 lung cancer cells bearing KRAS and LKB1 mutations. Compounds **5f**, **57**, and **57a** showed moderate growth inhibition of lung cancer cells A549 (IC_50_ = 5.97 ± 0.71, 6.06 ± 0.01, 4.41 ± 0.06 μM, respectively) and H460 (IC_50_ = 3.77 ± 0.02, 5.3 ± 0.01, 4.34 ± 0.03 μM, respectively). Especially, compound **50** demonstrated a two-fold improved growth inhibition of lung cancer cells A549 (IC_50_ = 3.94 ± 0.04 μM) and H460 (IC_50_ = 3.67 ± 0.06 μM) compared with **LW1497** in A549 (IC_50_ = 7.98 ± 0.53 μM) and H460 (IC_50_ = 6.14 ± 0.29 μM) cells. Compound **50** containing a piperidine linker was selected for further analysis.

#### 2.2.4. ATP Production in the Presence of MDH Inhibitor

**Figure 4:**
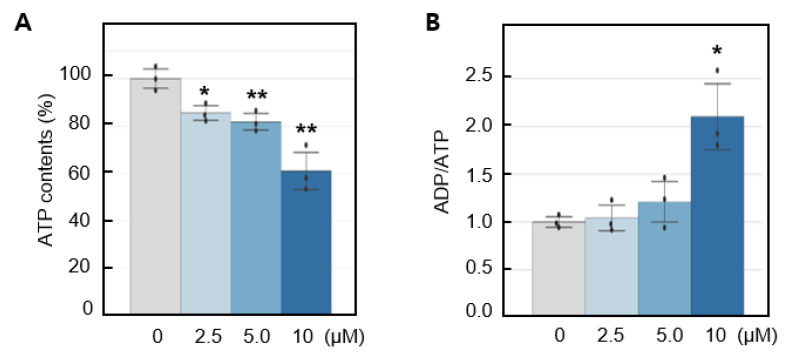
(**A**) The intracellular ATP content of compound **50** treated with A549 cells. (**B**) Determination of ADP/ATP ratio; * *p* ≤ 0.05 and ** *p* ≤ 0.01, compared with the control.

Malate transported to the interior of the mitochondria is oxidized to OAA by mitochondrial MDH2, thereby generating NADH, which then enters the ETC to produce ATP. Because inhibition of MAS is expected to reduce ATP production in cancer cells, the total amount of ATP was measured after treatment with compound **50**. Cells treated with compound **50** showed reduced ATP level in A549 cells (Figure 4A). In addition, an increase in ADP/ATP was observed, indicating the inhibition of ATP synthesis by oxidative phosphorylation (Figure 4B).

**Figure 5:**
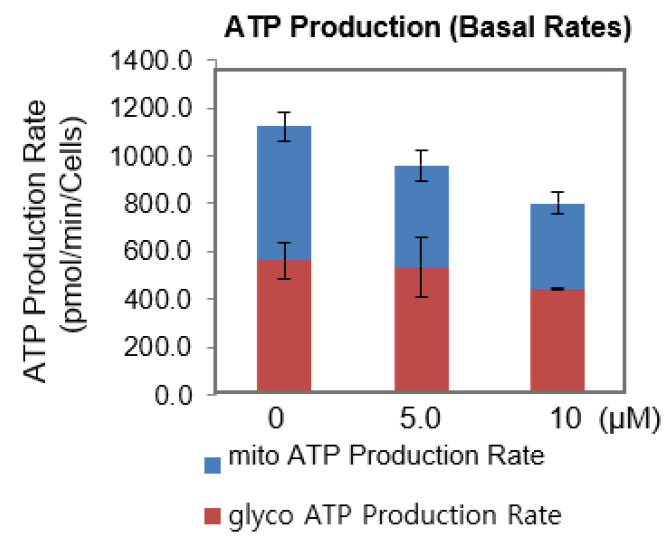
A measurement of the intracellular ATP production rates in A549 cells in a dose-dependent manner.

We then examined whether the ATP production pattern was altered by drug treatment using an XF analyzer for real-time metabolic analysis (Figure 5). As expected, the total ATP decreased by 14.6% and 28.6% in cells treated with 5 µM and 10 µM of compound **50,** respectively (Figure 5).

#### 2.2.5. Compound **50** Inhibits Hypoxia-Induced Accumulation of HIF-1α

**Figure 6:**
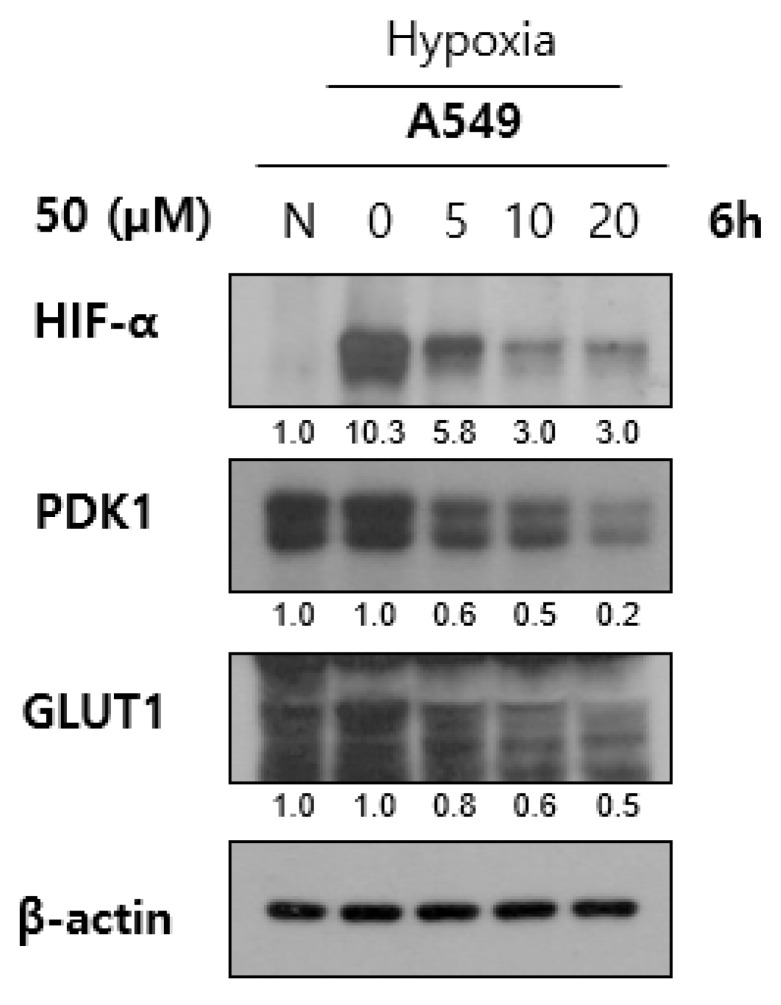
Dose-dependent inhibition of hypoxia-induced hypoxia-inducible factor-1 expression and its target genes by compound **50** in A549 cells.

Previously, it has been reported that the inhibition of MDH1 or MDH2 enzymes is related to HIF-1a expression levels [12]. We investigated whether compound **50** decreases HIF-1α accumulation in A549 cells (Figure 6). Compound **50** decreased HIF-1α accumulation in a dose-dependent manner (Figure 6). Additionally, we confirmed that compound **50** suppressed the protein expression of HIF-1α target genes, such as *GLUT1* and *PDK1* (Figure 6), as clearly shown by the Western blot assay.

#### 2.2.6. Compound **50** Suppresses the Expression of CD73 Regulated by HIF-1α

**Figure 7:**
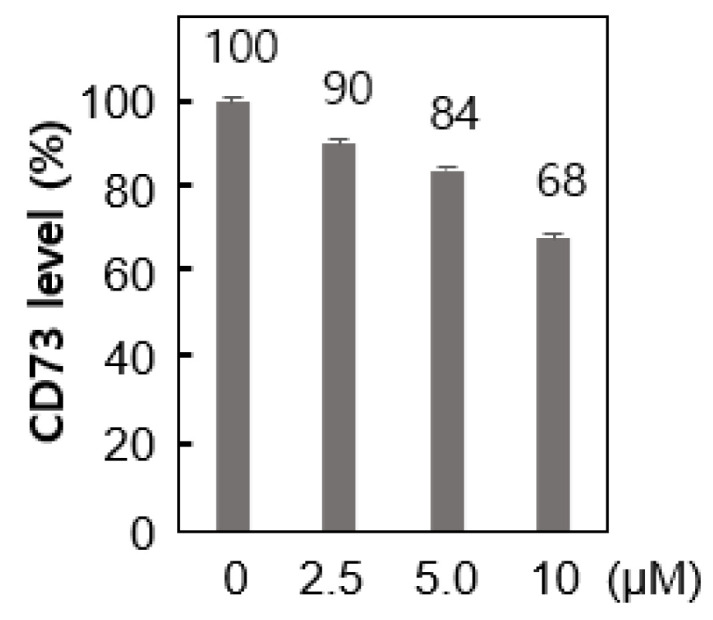
Dose-dependent inhibition of CD73 expression under hypoxic conditions by compound **50** in A549 cells.

It has been reported that CD73 expression is induced by HIF-1α under hypoxic conditions [21]. Therefore, we examined whether the expression of CD73 is inhibited by compound **50** under hypoxia in A549 lung cancer cells. As shown, CD73 expression increased by HIF-1α was significantly decreased by compound **50** in a concentration-dependent manner. To confirm the effect of compound **50** on CD73 expression, we performed ELISA for CD73. Compound **50** reduced CD73 levels in A549 cells in a dose-dependent manner (Figure 7). These results confirmed that compound **50** showed a significant inhibition of hypoxia-induced transcription and CD73 activity.

## 3. Discussion

The reprogramming of the core metabolism in tumors confers selective growth advantages, such as the ability to enhance cell proliferation and promote tumor growth and progression [22,23,24,25,26,27]. One of the mechanisms to suppress tumor growth is the impairment of the cancer cell’s metabolism. MAS plays a crucial role in the net transfer of the NADH produced in the cytoplasm into the mitochondria. The transportation of cytosolic NADH as an electron donor into the mitochondria is a major purpose of MAS in cancer cells. Therefore, MDH1 and MDH2, which are components of MAS, are the key molecules involved in ATP production via the process of mitochondrial oxidative phosphorylation [7,28,29]. In this study, we synthesized compound **50**, a potent MDH1/MDH2 dual inhibitor that suppressed MAS function in A549 lung cancer cells.

First, we measured ATP production to elucidate the inhibitory effects of compound **50** on mitochondrial respiration. Total ATP production was significantly reduced in A549 cells treated with compound **50**, which increased the ADP/ATP ratio. These results suggest that compound **50** inhibits the growth of A549 cells by inhibiting energy metabolism. In all cancer types, ATP supply is solely dependent on glycolysis and OXPHOS. Therefore, the selective inhibition of prevalent tumor energy metabolism may have a significant impact on cancer treatment. However, it can be argued that such a treatment may also severely affect the host’s energy metabolism.

Cancer cells significantly depend on cytosolic NADH production from glucose, fatty acids, and glutamine for ATP production [30]. Elevated glycolysis in cancer cells has been proposed as a mechanism that accelerates oxidative phosphorylation. MAS exerts control over NADH/NAD^+^ homeostasis to maintain the activity of mitochondrial lactate dehydrogenase and enables the aerobic oxidation of glycolytic l-lactate in the mitochondria.

In contrast, high aerobic glycolysis distinguishes cancer cells from normal cells and has been exploited to detect tumors in vivo. Lactate consumption in cells treated with compound **50** did not change significantly. Cancer cells consume the secreted lactate to produce ATP through the TCA cycle and oxidative phosphorylation processes [31,32]. Some cancer cells use lactate as a substrate for TCA intermediates via monocarboxylate transporters (MCT1/4) as well as for ATP production [32]. Lactate can be converted to pyruvate using lactate dehydrogenase (LDH) and can be further converted to acetyl-CoA by ATP-citrate lyase for fatty acid synthesis.

Under hypoxia, HIF-1α increases the transcriptional expression of various genes that are involved in cancer progression, metastasis, angiogenesis, and resistance to therapy [33]. We found that compound **50** decreased the expression of HIF-1α and its target genes, such as glucose transporters (*GLUT1* and *GLUT3*) and *PDK1*, preventing pyruvate entry into the TCA cycle [5,6]. We also observed a decrease in CD73 expression in the HIF-dependent adenosinergic pathway, which impairs NK cell function in tumors. Recently, the development of agents that can either block CD73 and/or target HIFs concurrently with NK cell-based therapies has emerged as an immunotherapeutic strategy with significant potential for the treatment of solid tumors.

## 4. Materials and Methods

### 4.1. Chemistry

#### 4.1.1. General Procedures

The commercial chemicals and solvents used were of reagent grade. All reactions were performed under a nitrogen atmosphere in oven-dried glassware. Reactions were monitored using thin-layer chromatography on 0.25-milimeter silica plates (E. Merck; silica gel 60 F254). The products were purified using flash column chromatography (Biotage) using silica gel 60 (230–400-mesh Kieselgel 60). Proton nuclear magnetic resonance (NMR) spectra were recorded on a Varian 400 MHz spectrometer (Varian Medical Systems, Inc., Palo Alto, CA, USA). The chemical shifts are provided in *δ* values (ppm), and the coupling constants are in hertz (Hz). ^13^C NMR spectra were recorded on the Varian 100 MHz spectrometer. The melting points were measured using the Thermo Scientific 9200 melting point apparatus. The mass spectra were recorded using high-resolution mass spectrometry (HRMS) (electron ionization MS) on a Waters G2 QTOF mass spectrometer. The purity of the final products was determined using reversed-phase high-performance liquid chromatography (RP-HPLC). The Waters Corp. HPLC system employed a YMC C18 (HS12S05-1564WT) column (5 μM, 12 nm) that had the dimensions of 4.6 mm × 150 mm and was equipped with an ultraviolet (UV) detector set at 254 nm. The RP-HPLC mobile phases used were (A) water with 0.05% trifluoroacetic acid (TFA) and (B) acetonitrile. The purity of the compounds was assessed using a gradient of 25% B to 100% B in 35 min. The purity of all biologically evaluated compounds was >95%.

#### 4.1.2. General Procedure for Acid–Amine Coupling

To a solution of acid (1.0 equiv), amine (1.1 equiv), and *N*, *N*-diisopropylethylamine (DIPEA; 3.0 equiv) in tetrahydrofuran (THF; 25 mL), we added 50% propylphosphonic anhydride (T3P) in ethyl acetate (EtOAc; 2.0 equiv). The resulting mixture was stirred at room temperature for 12 h. The reaction mixture was diluted with water (20 mL), and the aqueous phase was extracted using EtOAc (3 × 30 mL). The combined organic layers were dried over anhydrous sodium sulfate, filtered, and evaporated under reduced pressure. The residue was purified using silica gel column chromatography (15–45% EtOAc in hexane).

#### 4.1.3. General Procedure for the Michael Addition of Phenol with Acrylates

To a solution of phenol (1.0 equiv) in methyl or ethyl acrylate (20 mL), we added 4-dimethylaminopyridine (DMAP; 1.0 equiv) at room temperature. The resulting mixture was stirred at 100 °C in a microwave for 2 h. The reaction was quenched using saturated sodium bicarbonate solution (20 mL), and the aqueous phase was extracted using EtOAc (3 × 30 mL). The combined organic layers were dried over anhydrous sodium sulfate, filtered, and evaporated under reduced pressure. The residue was purified using silica gel column chromatography (0–15% EtOAc in hexane).

#### 4.1.4. General Procedure for Ester Hydrolysis

To a solution of methyl or ethyl ester (1.0 equiv) in THF:H_2_O (10:1), we added lithium hydroxide (LiOH; 2.0 equiv). The resulting mixture was stirred at room temperature for 12 h. The reaction mixture was evaporated under reduced pressure, and the crude residue was redissolved in water (20 mL) and acidified with 3 N hydrochloric acid (HCl; pH 6). The resulting precipitate was collected via suction filtration and dried under vacuum.

#### 4.1.5. General Procedure for Suzuki Coupling

To a solution of halogen compound (1.0 equiv) and corresponding boronic acid (1.2 equiv) in 1,4-dioxane: H_2_O (9:1), we added cesium carbonate (Cs_2_CO_3_; 3.0 equiv). The resulting mixture was degassed with nitrogen for 5 min. Tetrakis(triphenylphosphine)palladium (Pd(PPh_3_)_4_; 0.05 equiv) was added to the degassed solution, and the mixture was heated at 100 °C for 12 h in a sealed tube. The reaction mixture was cooled to room temperature and diluted with water (20 mL), and the aqueous phase was extracted using ethyl acetate (3 × 30 mL). The combined organic layers were dried over anhydrous sodium sulfate, filtered, and evaporated under reduced pressure. The crude residue was purified using silica gel column chromatography (10–15% EtOAc in hexane).

#### 4.1.6. General Procedure for Reductive Amination

To a solution of amine (1 equiv) in dichloromethane (DCM; 30 mL), we added aldehyde and/or ketone (1.2 equiv) and acetic acid (0.1 mL). The mixture was stirred at room temperature for 2 h, and sodium cyanoborohydride (NaCNBH_3_; 1.0 equiv.) was then added to the stirring solution. The resulting mixture was stirred at room temperature for 12 h. The reaction was quenched with aqueous sodium bicarbonate solution (30 mL), and the aqueous phase was extracted using DCM (3 × 40 mL). The combined organic layers were dried over anhydrous sodium sulfate, filtered, and evaporated under reduced pressure. The residue was purified using silica gel column chromatography (0–25% EtOAc in hexane). The spectral data for the final compounds is available in the supplementary information (Figures S5–S98).

##### (*E*)-Methyl 3-(4-(2,4,4-trimethylpentan-2-yl)phenoxy)acrylate (**2**)

To a solution of 4-(2,4,4-trimethylpentan-2-yl)phenol (**1**) (2.00 g, 9.69 mmol) and methyl propiolate (1.60 g, 19.3 mmol) in toluene (30 mL), we added triphenylphosphine (2.54 g, 9.69 mmol) at −10 °C. The resulting mixture was heated at 115 °C for 2 h. The mixture was cooled to room temperature, and the solvent was evaporated under reduced pressure. The residue was purified using silica gel column chromatography (15–20% EtOAc in hexane) to yield **2** as a white solid (2.00 g, 71.4% yield). ^1^H NMR (400 MHz, deuterated chloroform [CDCl_3_]): *δ* 7.80 (d, *J* = 12.3 Hz, 1H), 7.36 (d, *J* = 8.7 Hz, 2H), 6.97 (d, *J* = 8.4 Hz, 2H), 5.53 (d, *J* = 12.0 Hz, 1H), 3.73 (s, 3H), 1.73 (s, 2H), 1.36 (s, 6H), 0.71 (s, 9H); HRMS (electrospray ionization [ESI]): [M + H]^+^ calculated for C_18_H_27_O_3_ 291.1960, found 291.1950.

##### 3-(4-(2,4,4-Trimethylpentan-2-yl)phenoxy)propanoic acid (**4**)

To a solution of (*E*)-methyl 3-(4-(2,4,4-trimethylpentan-2-yl)phenoxy)acrylate (**2**) (1.00 g, 3.44 mmol) in methanol (20 mL), we added 10% Pd/C (73.0 mg, 0.68 mmol). The resulting mixture was stirred at room temperature for 12 h under a hydrogen gas atmosphere (1 atm) using a balloon. After the completion of the reaction, the mixture was filtered through a celite pad, and the crude filtrate was evaporated under reduced pressure. To a suspension of methyl ester (1.0 equiv) in THF:H2O (1:1), we added lithium hydroxide monohydrate (0.57 g, 13.7 mmol), and the mixture was stirred at room temperature for 12 h. The reaction mixture was evaporated and acidified with 3N HCl (pH 6), and the resulting precipitate was isolated via suction filtration and dried under vacuum to obtain **4** as a white solid (0.78 g, 81.5% yield). ^1^H NMR (400 MHz, dimethyl sulfoxide [DMSO]-*d_6_*): *δ* 12.35 (s, 1H), 7.12 (d, *J* = 8.0 Hz, 2H), 6.65 (d, *J* = 8.0 Hz, 2H), 4.11 (t, *J* = 4.0 Hz, 2H), 2.65 (t, *J* = 4.0 Hz, 2H), 1.68 (s, 2H), 1.36 (s, 6H), 0.71 (s, 9H); HRMS (ESI): [M + H]^+^ calculated for C_17_H_27_O_3_ 279.1960, found 279.1950.

##### *N*-(3-Methoxyphenyl)-3-(4-(2,4,4-trimethylpentan-2-yl)phenoxy)propanamide (**5a**)

The compound was prepared from 3-(4-(2,4,4-trimethylpentan-2-yl)phenoxy)propanoic acid (**4**) according to the general acid–amine coupling procedure. Purification via silica gel column chromatography (15–20% EtOAc in hexane) yielded **5a** as an off-white solid (65.0 mg, 47.4% yield). Mp: 97−99 °C. ^1^H NMR (400 MHz, CDCl_3_): *δ* 8.01 (s, 1H), 7.30 (t, *J* = 8.4 Hz, 3H), 7.19 (t, *J* = 8.2 Hz, 1H), 6.97 (d, *J* = 8.0 Hz, 1H), 6.85 (d, *J* = 11.2 Hz, 2H), 6.65 (d, *J* = 8.0 Hz, 1H), 4.30 (t, *J* = 6.0 Hz, 2H), 3.78 (s, 3H), 2.87 (t, *J* = 6.0 Hz, 2H), 1.70 (s, 2H), 1.33 (s, 6H), 0.71 (s, 9H); ^13^C NMR (100 MHz, CDCl_3_): *δ* 169.11, 160.11, 155.62, 143.23, 139.07, 129.63, 127.26, 113.79, 112.0, 110.14, 115.14, 105.61, 64.09, 56.93, 55.29, 55.26, 37.99, 37.79, 32.32, 31.77, 31.65; HRMS (ESI): [M + H]^+^ calculated for C_24_H_34_NO_3_ 384.2539, found 384.2530; RP-HPLC purity ≥ 99.7%, *t*_R_ = 25.9 min.

##### *N*-(3-Cyanophenyl)-3-(4-(2,4,4-trimethylpentan-2-yl)phenoxy)propanamide (**5b**)

The compound was prepared from 3-(4-(2,4,4-trimethylpentan-2-yl)phenoxy)propanoic acid (**4**) according to the general acid–amine coupling procedure. Purification via silica gel column chromatography (15–20% EtOAc in hexane) yielded **5b** as an off-white solid (50.0 mg, 37.0% yield). Mp: 112−114 °C. ^1^H NMR (400 MHz, CDCl_3_): *δ* 8.10 (br s, 1H), 7.93 (s, 1H), 7.70 (d, *J* = 7.6 Hz, 1H), 7.41 (q, *J* = 8.3 Hz, 2H), 7.32 (d, *J* = 8.8 Hz, 2H), 6.87 (d, *J* = 8.8 Hz, 2H), 4.33 (t, *J* = 5.6 Hz, 2H), 2.87 (t, *J* = 5.4 Hz, 2H), 1.71 (s, 2H), 1.35 (s, 6H), 0.71 (s, 9H); ^13^C NMR (100 MHz, CDCl_3_): *δ* 169.45, 155.39, 146.63, 138.66, 129.85, 127.69, 127.39, 123.88, 122.90, 118.49, 113.76, 112.99, 63.96, 56.91, 38.03, 37.71, 32.32, 31.77, 31.64; HRMS (ESI): [M + H]^+^ calculated for C_24_H_31_N_2_O_2_ 379.2386, found 379.2377; RP-HPLC purity ≥99.8%, *t*_R_ = 25.7 min.

##### *N*-(3-(Trifluoromethyl)phenyl)-3-(4-(2,4,4-trimethylpentan-2-yl)phenoxy)propanamide (**5c**)

The compound was prepared from 3-(4-(2,4,4-trimethylpentan-2-yl)phenoxy)propanoic acid (**4**) according to the general acid–amine coupling procedure. Purification via silica gel column chromatography (15–20% EtOAc in hexane) yielded **5c** as an off-white solid (55.0 mg, 36.4% yield). Mp: 88−90 °C. ^1^H NMR (400 MHz, CDCl_3_): *δ* 8.12 (s, 1H), 7.83 (s, 1H), 7.7 (d, *J* = 8.4 Hz, 1H), 7.42 (t, *J* = 8.0 Hz, 1H), 6.87 (d, *J* = 8.8 Hz, 2H), 4.33 (t, *J* = 5.8 Hz, 2H), 2.86 (t, *J* = 5.6 Hz, 2H), 1.70 (s, 2H), 1.34 (s, 6H), 0.71 (s, 9H); ^13^C NMR (100 MHz, CDCl_3_): *δ* 169.34, 155.49, 143.51, 138.51, 131.54, 131.23, 129.52, 127.35, 125.17, 122.88, 122.47, 120.83, 116.55, 113.80, 64.02, 56.93, 38.03, 37.76, 32.32, 31.76, 31.65; ^19^F NMR (376 MHz, CDCl_3_): *δ* -62.80; HRMS (ESI): [M + H]^+^ calculated for C_24_H_31_F_3_NO_2_ 422.2307, found 422.2304; RP-HPLC purity ≥97.6%, *t*_R_ = 28.2 min.

##### *N*-(3-(Trifluoromethoxy)phenyl)-3-(4-(2,4,4-trimethylpentan-2-yl)phenoxy)propanamide (**5d**)

The compound was prepared from 3-(4-(2,4,4-trimethylpentan-2-yl)phenoxy)propanoic acid (**4**) according to the general acid–amine coupling procedure. Purification via silica gel column chromatography (15–20% EtOAc in hexane) yielded **5d** as an off-white semisolid (60.0 mg, 62.7% yield). ^1^H NMR (400 MHz, CDCl_3_): *δ* 8.02 (s, 1H), 7.58 (s, 1H), 7.33 (q, *J* = 8.0 Hz, 4H), 6.96(d, *J* = 7.6 Hz, 2H), 6.87 (d, *J* = 8.4 Hz, 2H), 4.32 (t, *J* = 5.6 Hz, 2H), 2.84(t, *J* = 5.8 Hz, 2H), 1.71(s, 2H), 1.34(s, 6H), 0.71(s, 9H); ^13^C NMR (100 MHz, DMSO-*d_6_*): *δ* 169.73, 156.39, 148.95, 148.93, 141.96, 141.19, 130.89, 127.34, 121.79, 119.23, 117.99, 115.60, 114.00, 111.54, 63.89, 56.73, 37.98, 36.91, 32.42, 31.97; ^19^F NMR (376 MHz, CDCl_3_): *δ* -57.78; HRMS (ESI): [M + H]^+^ calculated for C_24_H_31_F_3_NO_3_ 438.2256, found 438.2250; RP-HPLC purity ≥99.1%, *t*_R_ = 28.6 min.

##### *N*-(2-(Trifluoromethoxy)phenyl)-3-(4-(2,4,4-trimethylpentan-2-yl)phenoxy)propanamide (**5e**)

The compound was prepared from 3-(4-(2,4,4-trimethylpentan-2-yl)phenoxy)propanoic acid (**4**) according to the general acid–amine coupling procedure. Purification via silica gel column chromatography (15−20% EtOAc in hexane) yielded **5e** as a colorless liquid (45.0 mg, 11.9% yield). ^1^H NMR (400 MHz, CDCl_3_): *δ* 8.34 (s, 1H), 7.32–7.25 (m, 4H), 7.10 (dd, *J* = 11.3, 4.3 Hz, 1H), 6.87 (d, *J* = 8.8 Hz, 2H), 5.30 (s, 1H), 4.32 (t, *J* = 5.6 Hz, 2H), 2.89 (t, *J* = 5.6 Hz, 2H), 2.04 (s, 3H), 1.71 (s, 2H), 1.34 (s, 6H), 0.71 (s, 9H); ^13^C NMR (100 MHz, CDCl_3_): *δ* 169.09, 155.86, 149.57, 139.14, 134.41, 130.34, 129.53, 129.25, 126.95, 126.27, 124.43, 123.70, 121.70, 119.14, 118.56, 118.18, 117.47, 116.51, 113.02, 107.53, 63.93, 37.55; ^19^F NMR (376 MHz, CDCl_3_): *δ* -57.58; HRMS (ESI): [M + H]^+^ calculated for C_24_H_31_F_3_NO_3_ 438.2256, found 438.2247; RP-HPLC purity ≥ 100%, *t*_R_ = 28.5 min.

##### *N*-(4-(Trifluoromethoxy)phenyl)-3-(4-(2,4,4-trimethylpentan-2-yl)phenoxy)propanamide (**5f**)

The compound was prepared from 3-(4-(2,4,4-trimethylpentan-2-yl)phenoxy)propanoic acid (**4**) according to the general acid–amine coupling procedure. Purification via silica gel column chromatography (15–20% EtOAc in hexane) yielded **5f** as a colorless liquid (37.0 mg, 35.0% yield). ^1^H NMR (400 MHz, DMSO-*d_6_*): *δ* 8.01 (s, 1H), 7.54 (d, *J* = 8.8 Hz, 2H),7.31 (d, *J* = 8.4 Hz, 2H), 7.17 (d, *J* = 8.8 Hz, 2H), 6.87 (d, *J* = 8.8 Hz, 2H), 4.32 (t, *J* = 5.6 Hz, 2H), 2.85 (t, *J* = 5.4 Hz, 2H), 1.71 (s, 2H), 1.35 (s, 6H), 0.71 (s, 9H); ^13^C NMR (100 MHz, CDCl_3_): *δ* 169.14, 155.46, 145.23, 143.50, 136.47, 127.36, 121.78, 121.74, 120.98, 119.18, 113.76, 64.08, 56.92, 38.02, 37.73, 32.33, 31. 77, 31.65; ^19^F NMR (376 MHz, CDCl_3_): *δ* -58.18; HRMS (ESI): [M + H]^+^ calculated for C_24_H_31_F_3_NO_3_ 438.2256, found 438.2261; RP-HPLC purity ≥ 99.9%, *t*_R_ = 28.8 min.

##### 1-(4-Methoxyphenyl)-3,3-dimethylbutan-1-one (**8**)

3,3-Dimethylbutanoic acid (**6**) (6.00 g, 51.6 mmol) was dissolved in thionyl chloride (10 mL), and the mixture was stirred for 12 h at room temperature under an inert condition. The excess thionyl chloride was removed via evaporation. The residue was redissolved in dichloromethane (DCM; 10 mL) and then added portion-wise to a solution of AlCl_3_ (10.3 g, 77.4 mmol) and anisole (5.58 g, 51.6 mmol) in DCM (50 mL) at room temperature. The resulting solution was stirred at room temperature for 2 h. The reaction was quenched with a mixture of HCl (40 mL) in ice-cold water (200 mL), and the aqueous phase was extracted using DCM (3 × 100 mL). The combined organic layers were dried over anhydrous sodium sulfate, filtered, and evaporated under reduced pressure. The residue was purified using silica gel column chromatography (0–15% EtOAc in hexane) to yield **8** as an off-white liquid (5.20 g, 48.8% yield). ^1^H NMR (400 MHz, CDCl_3_): *δ* 7.92–7.88 (m, 2H), 6.98–6.93 (m, 2H), 3.86 (s, 3H), 2.82 (s, 2H), 1.05 (s, 9H); HRMS (ESI): [M + H]^+^ calculated for C_13_H_19_O_2_ 207.1385, found 207.1376.

##### 1-(4-Hydroxyphenyl)-3,3-dimethylbutan-1-one (**9**)

To a solution of 1-(4-methoxyphenyl)-3,3-dimethylbutan-1-one (**8**) (4.00 g, 19.3 mmol) in DCE (25 mL), we added aluminum chloride (AlCl_3_; 5.17 g, 38.6 mmol) at 0 °C. The resulting mixture was stirred at 75 °C for 16 h. The reaction mixture was cooled to room temperature and quenched with a mixture of HCl (20 mL) in ice-cold water (100 mL), and the aqueous phase was extracted using DCM (3 × 100 mL). The combined organic layers were dried over anhydrous sodium sulfate, filtered, and evaporated under reduced pressure. The residue was purified using silica gel column chromatography (0–12% EtOAc in hexane) to yield **9** as an off-white oil (3.10 g, 83.7% yield). ^1^H NMR (400 MHz, CDCl_3_): *δ* 7.92–7.88 (m, 2H), 6.98–6.93 (m, 2H), 2.82 (s, 2H), 1.05 (s, 9H); HRMS (ESI): [M + H]^+^ calculated for C_12_H_17_O_2_ 193.1229, found 193.1221.

##### 4-(1-Hydroxy-3,3-dimethylbutyl)phenol (**10**)

To a solution of 1-(4-hydroxyphenyl)-3,3-dimethylbutan-1-one (**9**) (1.30 g, 6.76 mmol) in THF (20 mL) at 0 °C, we added lithium aluminum hydride (LAH; 0.38 g, 10.1 mmol) portion-wise. The resulting mixture was stirred at the same temperature for 2 h. The reaction was quenched with saturated ammonium chloride solution (20 mL), and the aqueous phase was extracted using EtOAc (3 × 50 mL). The combined organic layers were dried over anhydrous sodium sulfate, filtered, and evaporated under reduced pressure. The residue was purified using silica gel column chromatography (0–20% EtOAc in hexane) to yield **10** as an off-white solid (0.91 g, 69.2% yield)**.** ^1^H NMR (400 MHz, DMSO-*d_6_*): *δ* 9.18 (s, 1H), 7.09 (d, *J* = 8.4 Hz, 2H), 6.70–6.63 (m, 2H), 4.72 (d, *J* = 4.5 Hz, 1H), 4.53 (dt, *J* = 8.3, 4.1 Hz, 1H), 1.57 (dd, *J* = 14.0, 8.4 Hz, 1H), 1.37 (dd, *J* = 14.0, 3.8 Hz, 1H), 0.91 (s, 9H); HRMS (ESI): [M + H]^+^ calculated for C_12_H_19_O_2_ 195.1385; the mass was not observed.

##### (*E*)-Benzyl-3-(4-(1-hydroxy-3,3-dimethylbutyl)phenoxy)acrylate (**12**)

To a solution of 4-(1-hydroxy-3,3-dimethylbutyl)phenol (**10**) (0.90 g, 4.63 mmol) and *N*-methyl morpholine (0.05 mL, 0.463 mmol) in acetonitrile (25 mL), we added benzyl propiolate (0.89 g, 5.55 mmol). The resulting mixture was stirred at room temperature for 2 h. The reaction mixture was evaporated under reduced pressure. The residue was purified using silica gel column chromatography (0–10% EtOAc in hexane) to yield **12** as a colorless oil (1.20 g, 73.1% yield). ^1^H NMR (400 MHz, DMSO-*d_6_*): *δ* 7.87 (d, *J* = 12.2 Hz, 1H), 7.41–7.29 (m, 6H), 7.14 (d, *J* = 8.7 Hz, 2H), 5.59 (d, *J* = 12.2 Hz, 1H), 5.15 (s, 2H), 5.00 (d, *J* = 4.8 Hz, 1H), 4.70–4.62 (m, 1H), 1.58 (dd, *J* = 14.1, 8.7 Hz, 1H), 1.39 (dd, *J* = 14.1, 3.1 Hz, 1H), 0.94 (s, 9H); HRMS (ESI): [M + H]^+^ calculated for C_22_H_27_O_4_ 355.1909, found 355.1909.

##### 3-(4-(3,3-Dimethylbutyl)phenoxy)propanoic acid (**13**)

To a solution of ((*E*)-benzyl 3-(4-(1-hydroxy-3,3-dimethylbutyl)phenoxy)acrylate (**12**) (1.20 g, 3.38 mmol) in THF (25 mL), we added 10% Pd/C (0.35 g, 0.33 mmol). The resulting mixture was stirred at room temperature for 12 h under a hydrogen gas atmosphere (1 atm) using a balloon. After completion of the reaction, the reaction mixture was filtered through a celite pad, and the crude filtrate was evaporated under reduced pressure to yield **13** as an off-white powder (0.70 g, 82.7% yield). ^1^H NMR (400 MHz, DMSO-*d_6_*): *δ* 7.08 (d, *J* = 8.5 Hz, 2H), 6.81 (d, *J* = 8.5 Hz,2H), 4.11 (t, *J* = 6.1 Hz, 2H), 2.64 (t, *J* = 6.1 Hz, 2H), 2.48–2.42 (m, 2H), 1.45–1.32 (m, 2H), 0.92 (s, 9H); HRMS (ESI): [M + H]^+^ calculated for C_15_H_23_O 251.1647, found 251.1656.

##### Methyl-3-(3-(4-(4-hydroxy-2,4-dimethylpentan-2-yl)phenoxy)propanamido)benzoate (**14**)

The compound was prepared from 3-(4-(3,3-dimethylbutyl)phenoxy)propanoic acid (**13**) according to the general acid–amine coupling procedure. Purification via silica gel column chromatography (20% EtOAc in hexane) yielded **14** as an off-white oil (0.09 g, 55.2% yield). ^1^H NMR (400 MHz, CDCl_3_): *δ* 8.00 (s, 1H), 7.59 (s, 1H), 7.36–7.28 (m, 2H), 7.13 (d, *J* = 8.1 Hz, 2H), 6.96 (d, *J* = 7.0 Hz, 1H), 6.87 (d, *J* = 7.7 Hz, 2H), 4.32 (t, *J* = 5.6 Hz, 2H), 2.85 (t, *J* = 5.6 Hz, 2H), 2.57–2.47 (m, 2H), 1.46 (dd, *J* = 10.6, 6.5 Hz, 2H), 0.95 (s, 9H); ^13^C NMR (100 MHz, CD_3_OD): *δ* 170.68, 156.62, 149.23, 140.14, 135.78, 129.42, 128.90, 121.75, 119.20, 117.38, 117.08, 116.35, 115.76, 114.87, 114.12, 113.57, 63.73, 36.67, 29.95, 28.33; ^19^F NMR (376 MHz, CDCl_3_): *δ* -57.81; HRMS (ESI): [M + H]^+^ calculated for C_22_H_27_F_3_NO_3_ 410.1943, found 410.1932; RP-HPLC purity ≥ 98.3%, *t*_R_ = 27.1 min.

##### *tert*-Butyl (4-hydroxyphenyl)carbamate (**16**)

To a solution of 4-aminophenol (**15**) (3.00 g, 27.4 mmol) in THF (25 mL), we added triethylamine (Et_3_N; 11.5 mL, 82.4 mmol) followed by Boc anhydride (7.47 mL, 32.8 mmol). The resulting mixture was stirred at room temperature for 16 h. The reaction mixture was diluted with ice-cold water (100 mL), and the aqueous phase was extracted using EtOAc (3 × 70 mL). The combined organic layers were dried over anhydrous sodium sulfate, filtered, and evaporated under reduced pressure. The residue was purified using silica gel column chromatography (0–30% EtOAc in hexane) to yield **16** (4.50 g, 79.6% yield) as an off-white solid. ^1^H NMR (400 MHz, CDCl_3_): *δ* 7.19 (d, *J* = 8.4 Hz, 2H), 6.77–6.73 (m, 2H), 6.33 (s, 1H), 4.99 (s, 1H), 1.51 (s, 9H); HRMS (ESI): [M + H]^+^ calculated for C_11_H_16_NO_3_ 210.1130; the mass was not observed.

##### (*E*)-Benzyl-3-(4-((*tert*-butoxycarbonyl)amino)phenoxy)acrylate (**17**)

To a solution of *tert*-butyl(4-hydroxyphenyl)carbamate (**16**) (4.00 g, 19.1 mmol) and *N*-methyl morpholine (0.84 mL, 7.64 mmol) in acetonitrile (25 mL), was added benzyl propiolate (3.67 g, 22.9 mmol). The reaction mixture was stirred at ambient temperature for 2 h. The reaction mixture was evaporated under reduced pressure. The residue was purified using silica gel column chromatography (0–20% EtOAc in hexane) to yield **17** (0.80 g, 11.3% yield) as a colorless semisolid. ^1^H NMR (400 MHz, DMSO-*d_6_*): *δ* 9.39 (s, 1H), 7.82 (d, *J* = 12.2 Hz, 1H), 7.48 (d, *J* = 8.8 Hz, 2H), 7.37 (t, *J* = 5.6 Hz, 4H), 7.33 (dd, *J* = 8.5, 3.9 Hz, 1H), 7.10 (d, *J* = 8.9 Hz, 2H), 5.54 (d, *J* = 12.2 Hz, 1H), 5.15 (s, 2H), 1.47 (s, 9H); HRMS (ESI): [M + H]^+^ calculated for C_21_H_24_NO_5_ 370.1654, found 370.1650.

##### 3-(4-((*tert*-Butoxycarbonyl)amino)phenoxy)propanoic acid (**18**)

To a solution of (*E*)-benzyl 3-(4-((*tert*-butoxycarbonyl)amino)phenoxy)acrylate (**17**) (0.80 g, 2.16 mmol) in THF (25 mL), was added 10% Pd/C (0.46 g, 0.43 mmol). The resulting mixture was stirred at room temperature for 12 h under a hydrogen gas atmosphere (1 atm) using a balloon. After completion of the reaction, the reaction mixture was filtered through a celite pad, and the crude filtrate was evaporated under reduced pressure to yield **18** as an off-white solid (0.35 g, 69.8% yield). ^1^H NMR (400 MHz, DMSO-*d_6_*): *δ* 9.11 (s, 1H), 7.33 (d, *J* = 8.5 Hz, 2H), 6.82 (d, *J* = 8.9 Hz, 2H), 4.09 (t, *J* = 6.0 Hz, 2H), 2.65 (t, *J* = 6.0 Hz, 2H), 1.46 (s, 9H); HRMS (ESI): [M + H]^+^ calculated for C_14_H_20_NO_5_ 282.1341, found 282.1333.

##### *tert*-Butyl(4-(3-oxo-3-((3-(trifluoromethoxy)phenyl)amino)propoxy)phenyl)carbamate (**19**)

The compound was prepared from 3-(4-((*tert*-butoxycarbonyl)amino)phenoxy)propanoic acid (**18**) according to the general acid–amine coupling procedure. Purification via silica gel column chromatography (20% EtOAc in hexane) yielded **19** as an off-white solid (1.35 g, 51.0% yield). ^1^H NMR (400 MHz, CDCl_3_): *δ* 7.92 (s, 1H), 7.59 (s, 1H), 7.32 (d, *J* = 6.5 Hz, 2H), 7.30 (d, *J* = 8.7 Hz, 2H), 6.97 (s, 1H), 6.89 (d, *J* = 9.0 Hz, 2H), 6.37 (s, 1H), 4.31 (t, *J* = 5.8 Hz, 2H), 2.83 (t, *J* = 5.7 Hz, 2H), 1.51 (s, 9H); HRMS (ESI): [M + H]^+^ calculated for C_21_H_24_F_3_N_2_O_5_ 441.1637, found 441.1635.

##### 3-(4-Aminophenoxy)-*N*-(3-(trifluoromethoxy)phenyl)propanamide (**20**)

To a solution of *tert*-butyl(4-(3-oxo-3-((3-(trifluoromethoxy)phenyl)amino)propoxy)phenyl)carbamate (**19**) (1.30 g, 2.95 mmol) in DCM (25 mL), we added trifluoroacetic acid (TFA; 5 mL). The resulting mixture was stirred at room temperature for 12 h. The reaction mixture was basified with sodium bicarbonate solution (50 mL), and the aqueous phase was extracted using DCM (3 × 50 mL). The combined organic layers were dried over anhydrous sodium sulfate, filtered, and evaporated under reduced pressure. The residue was purified using silica gel column chromatography (0–60% EtOAc in hexane) to yield **20** as an off-brown solid (1.00 g, 99.0% yield). ^1^H NMR (400 MHz, DMSO-*d_6_*): *δ* 10.38 (s, 1H), 7.80 (s, 1H), 7.47 (d, *J* = 8.6 Hz, 1H), 7.42 (t, *J* = 8.1 Hz, 1H), 7.17 (d, *J* = 8.8 Hz, 2H), 7.02 (d, *J* = 7.2 Hz, 1H), 6.99 (d, *J* = 8.9 Hz, 2H), 4.24 (t, *J* = 5.9 Hz, 2H), 2.80 (t, *J* = 5.8 Hz, 2H), NH_2_ protons were not observed; HRMS (ESI): [M + H]^+^ calculated for C_16_H_16_F_3_N_2_O_3_ 341.1113, found 341.1119.

##### 3-(4-(Neopentylamino)phenoxy)-*N*-(3-(trifluoromethoxy)phenyl)propanamide (**21**)

To a solution of 3-(4-aminophenoxy)-*N*-(3-(trifluoromethoxy)phenyl)propanamide (**20**) (1.0 g, 2.93 mmol) in DCM:MeOH (30 mL, 9:1), we added pivalaldehyde (0.5 mL, 4.40 mmol) followed by three drops of acetic acid; the mixture was stirred at room temperature for 1 h. Then, NaCNBH_3_ (0.37 g, 5.86 mmol) was added to the stirring solution. The resulting mixture was stirred at room temperature for 16 h. The reaction mixture was basified with 50 mL of a sodium bicarbonate solution, and the aqueous phase was extracted using DCM (3 × 50 mL). The combined organic layers were dried over anhydrous sodium sulfate, filtered, and evaporated under reduced pressure. The residue was purified using silica gel column chromatography (0–30% EtOAc in hexane) to yield **21** as a colorless liquid (0.60 g, 56.4% yield). ^1^H NMR (400 MHz, CDCl_3_): *δ* 8.20 (s, 1H), 7.58 (s, 1H), 7.36–7.27 (m, 2H), 6.95 (d, *J* = 7.4 Hz, 1H), 6.82 (d, *J* = 8.8 Hz, 2H), 6.60 (d, *J* = 8.8 Hz, 2H), 4.25 (t, *J* = 5.7 Hz, 2H), 2.85 (s, 2H), 2.81 (t, *J* = 5.6 Hz, 2H), 0.99 (s, 9H); ^13^C NMR (100 MHz, CDCl_3_): *δ* 169.58, 149.70, 144.49, 139.32, 130.27, 121.69, 119.16, 118.15, 116.91, 116.14, 114.00, 112.96, 65.29, 56.75, 37.78, 31.78, 27.73; ^19^F NMR (376 MHz, CDCl_3_): *δ* -57.77; HRMS (ESI): [M + H]^+^ calculated for C_21_H_26_F_3_N_2_O_3_ 411.1896, found 411.1901; RP-HPLC purity ≥ 97.2%, *t*_R_ = 12.1 min.

##### 3-(4-(Methyl(neopentyl)amino)phenoxy)-*N*-(3-(trifluoromethoxy)phenyl)propanamide (**22**)

To a solution of 3-(4-(neopentylamino)phenoxy)-*N*-(3-(trifluoromethoxy)phenyl)propanamide (**21**) (0.40 g, 0.97 mmol) in DCM:MeOH (30 mL, 9:1), we added para formaldehyde (0.06 g, 1.94 mmol) followed by three drops of acetic acid; the mixture was stirred at room temperature for 1 h. NaCNBH_3_ (0.122 g, 1.94 mmol) was added to the stirring solution. The resulting mixture was stirred at room temperature for 16 h. The reaction mixture was basified with sodium bicarbonate solution (50 mL), and the aqueous phase was extracted using DCM (3 × 50 mL). The combined organic layers were dried over anhydrous sodium sulfate, filtered, and evaporated under reduced pressure. The residue was purified by silica gel column chromatography (0–30% EtOAc in hexane) to yield **22** as a colorless liquid (0.26 g, 63.2% yield). ^1^H NMR (400 MHz, CDCl_3_): *δ* 8.23 (s, 1H), 7.59 (s, 1H), 7.35 (d, *J* = 8.4 Hz, 1H), 7.32 (d, *J* = 7.9 Hz, 1H), 6.95 (d, *J* = 7.8 Hz, 1H), 6.86 (d, *J* = 9.2 Hz, 2H), 6.71 (d, *J* = 9.2 Hz, 2H), 4.26 (t, *J* = 5.6 Hz, 2H), 3.07 (s, 2H), 2.94 (s, 3H), 2.81 (t, *J* = 5.6 Hz, 2H), 0.98 (s, 9H); ^13^C NMR (100 MHz, DMSO-*d_6_*): *δ* 169.86, 149.70, 148.95, 148.93, 145.88, 141.20, 130.85, 121.79, 119.24, 117.99, 115.72, 115.57, 113.25, 111.53, 64.68, 64.36, 41.81, 37.14, 35.12, 28.67; ^19^F NMR (376 MHz, CDCl_3_): *δ* -57.76; HRMS (ESI): [M + H]^+^ calculated for C_22_H_28_F_3_N_2_O_3_ 425.2052, found 425.2059; RP-HPLC purity ≥ 97.1%, *t*_R_ = 12.8 min.

##### 3-(4-Methoxyphenyl)-3-methylbutanoic acid (**24**)

To a mixture of 3-bromo-3-methylbutanoic acid (**23**) (12.8 g, 71.1 mmol) and anisole (7.69 g, 71.1 mmol) in DCM (100 mL), we added AlCl_3_ (28.4 g, 213.3 mmol) portion-wise at 0 °C. The resulting mixture was stirred at 65 °C for 3 h. The reaction was quenched with a mixture of HCl (40 mL) in 200 mL of ice-cold water, and the aqueous phase was extracted using DCM (3 × 100 mL). The combined organic layers were washed with 10% sodium hydroxide (NaOH) solution. The aqueous phase was acidified to pH 1 using 2 M of HCl and extracted using DCM (3 × 100 mL). The combined organic layers were dried over anhydrous sodium sulfate, filtered, and evaporated under reduced pressure. The residue was purified using silica gel column chromatography (0–40% EtOAc in hexane) to yield **24** as an off-white solid (3.00 g, 20.2% yield). ^1^H NMR (400 MHz, DMSO-*d_6_*): *δ* 11.83 (s, 1H), 7.31–7.26 (m, 2H), 6.85–6.82 (m, 2H), 3.72 (s, 3H), 2.52 (s, 2H), 1.35 (s, 6H); HRMS (ESI): [M + H]^+^ calculated for C_12_H_17_O_3_ 209.2650; the mass was not observed.

##### Methyl 3-(4-methoxyphenyl)-3-methylbutanoate (**25**)

To a solution of 3-(4-methoxyphenyl)-3-methylbutanoic acid (**24**) (2.93 g, 14.0 mmol) in MeOH (20.0 mL), we added concentrated sulfuric acid (H_2_SO_4_; 1.00 mL). The resulting mixture was stirred at room temperature for 12 h. The reaction was quenched with saturated sodium bicarbonate solution (40 mL), and the aqueous phase was extracted using DCM (3 × 100 mL). The combined organic layers were dried over anhydrous sodium sulfate, filtered, and evaporated under reduced pressure. The residue was purified using silica gel column chromatography (0–5% EtOAc in hexane) to yield **25** as an off-colorless liquid (2.12 g, 67.9% yield). ^1^H NMR (400 MHz, DMSO-*d_6_*): *δ* 7.27 (d, *J* = 8.6 Hz, 2H), 6.83 (d, *J* = 8.3 Hz, 2H), 3.72 (s, 3H), 3.44 (s, 3H), 2.60 (s, 2H), 1.34 (s, 6H); HRMS (ESI): [M + H]^+^ calculated for C_13_H_19_O_3_ 223.1334, found 223.1335.

##### Methyl 3-(4-hydroxyphenyl)-3-methylbutanoate (**26**)

To a solution of methyl 3-(4-methoxyphenyl)-3-methylbutanoate (**25**) (2.04 g, 9.16 mmol) in DCM (25 mL), we added boron tribromide (1.0 M BBr_3_ in DCM; 1.37 mL, 13.7 mmol) at 0 °C. The resulting mixture was stirred at room temperature for 2 h. The reaction was quenched with methanol (MeOH; 5 mL) and diluted with water (30 mL). The aqueous phase was extracted using DCM (3 × 100 mL). The combined organic layers were dried over anhydrous sodium sulfate, filtered, and evaporated under reduced pressure. The residue was purified using silica gel column chromatography (0–5% EtOAc in hexane) to yield **26** as an off-brown solid (1.27 g, 66.5% yield). ^1^H NMR (400 MHz, DMSO-*d_6_*): *δ* 7.27 (d, *J* = 8.6 Hz, 2H), 6.83 (d, *J* = 8.3 Hz, 2H), 3.72 (s, 3H), 3.44 (s, 3H), 2.60 (s, 2H), 1.34 (s, 6H); HRMS (ESI): [M + H]^+^ calculated for C_12_H_17_O_3_ 209.1178, found 209.1171.

##### 4-(4-Hydroxy-2,4-dimethylpentan-2-yl)phenol (**27**)

To a solution of methyl 3-(4-hydroxyphenyl)-3-methylbutanoate (**26**) (1.21 g, 5.83 mmol) in THF (25 mL), we added 3.0 M of methyl magnesium bromide in ether (2.09 g, 17.5 mmol) at 0 °C. The resulting mixture was stirred at room temperature for 2 h. The reaction was quenched with water (40 mL), and the aqueous phase was extracted using EtOAc (3 × 100 mL). The combined organic layers were dried over anhydrous sodium sulfate, filtered, and evaporated under reduced pressure. The residue was purified using silica gel column chromatography (5−15% EtOAc in hexane) to yield **27** as an off-white solid (0.86 g, 71.1% yield). ^1^H NMR (400 MHz, DMSO-*d_6_*): *δ* 9.05 (s, 1H), 7.13 (d, *J* = 8.7 Hz, 2H), 6.65 (d, *J* = 8.7 Hz, 2H), 3.85 (s, 1H), 1.81 (s, 2H), 1.28 (s, 6H), 0.79 (s, 6H). HRMS (ESI): [M + H]^+^ calculated for C_13_H_21_O_3_ 209.3090; the mass was not observed.

##### Benzyl (*E*)-3-(4-(4-hydroxy-2,4-dimethylpentan-2-yl)phenoxy)acrylate (**28**)

To a mixture of 4-(4-hydroxy-2,4-dimethylpentan-2-yl)phenol (**27**) (0.77 g, 3.70 mmol) and benzyl propiolate (0.65 g, 4.07 mmol) in acetonitrile (25 mL), we added *N*-methyl morpholine (0.05 g, 0.52 mmol). The resulting mixture was stirred at room temperature for 2 h. The reaction mixture was evaporated under reduced pressure. The residue was purified using silica gel column chromatography (0–10% EtOAc in hexane) to yield **28** as an off-white solid (1.3 g, 95.6% yield). ^1^H NMR (400 MHz, DMSO-*d_6_*): *δ* 7.87 (d, *J* = 12.2 Hz, 1H), 7.42 (d, *J* = 8.8 Hz, 2H), 7.38 (d, *J* = 4.4 Hz, 4H), 7.35–7.31 (m, 1H), 7.10 (d, *J* = 8.8 Hz, 2H), 5.57 (d, *J* = 12.2 Hz, 1H), 5.15 (s, 2H), 3.94 (s, 1H), 1.87 (s, 2H), 1.34 (s, 6H), 0.79 (s, 6H).

##### 3-(4-(4-Hydroxy-2,4-dimethylpentan-2-yl)phenoxy)propanoic acid (**29**)

To a solution of benzyl (*E*)-3-(4-(4-hydroxy-2,4-dimethylpentan-2-yl)phenoxy)acrylate (**28**) (1.40 g, 3.80 mmol) in THF (100 mL), we added 10% Pd/C (0.80 g, 0.75 mmol). The resulting mixture was stirred at room temperature for 18 h under a hydrogen gas atmosphere (1 atm) using a balloon. After completion of the reaction, the reaction mixture was filtered through a celite pad, and the crude filtrate was evaporated under reduced pressure to yield **29** as an off-brown liquid (0.89 g, 83.5% yield). ^1^H NMR (400 MHz, DMSO-*d_6_*): *δ* 7.26 (d, *J* = 8.8 Hz, 2H), 6.81 (d, *J* = 8.8 Hz, 2H), 4.11 (t, *J* = 6.1 Hz, 2H), 3.89 (s, 1H), 2.66 (t, *J* = 6.0 Hz, 2H), 1.84 (s, 2H), 1.30 (s, 6H), 0.79 (s, 6H); HRMS (ESI): [M + H]^+^ calculated for C_16_H_25_O_4_ 281.1753, found 281.1758.

##### 3-(4-(4-Hydroxy-2,4-dimethylpentan-2-yl)phenoxy)-*N*-(3-(trifluoromethoxy)phenyl)propanamide (**30**)

The compound was prepared from 3-(4-(4-hydroxy-2,4-dimethylpentan-2-yl)phenoxy)propanoic acid (**29**) according to the general acid–amine coupling procedure. Purification via silica gel column chromatography (0–25% EtOAc in hexane) yielded **30** as an off-yellow oil (0.97 g, 76.4% yield). ^1^H NMR (400 MHz, DMSO-*d_6_*): *δ* 7.26 (d, *J* = 8.8 Hz, 2H), 6.81 (d, *J* = 8.8 Hz, 2H), 4.11 (t, *J* = 6.1 Hz, 2H), 3.89 (s, 1H), 2.66 (t, *J* = 6.0 Hz, 2H), 1.84 (s, 2H), 1.30 (s, 6H), 0.79 (s, 6H); ^13^C NMR (101 MHz, DMSO-*d_6_*): δ 169.76 (s), 156.31 (s), 148.95 (s), 142.61 (s), 141.16 (s), 130.90 (s), 127.27 (s), 124.33 (s), 121.79 (s), 119.24 (s), 118.02 (s), 116.69 (s), 115.63 (s), 114.06 (s), 111.53 (s), 70.66 (s), 63.92 (s), 56.22 (s), 37.20 (s), 36.92 (s), 31.71 (s), 31.51 (s); ^19^F NMR (376 MHz, CDCl_3_): *δ* -57.77; HRMS (ESI): [M + H]^+^ calculated for C_23_H_29_F_3_NO_4_ 440.2048, found 440.2038; RP-HPLC purity ≥ 97.4%, *t*_R_ = 19.6 min.

##### Ethyl 3-(4-bromophenoxy)propanoate (**32**)

The compound was prepared from 4-bromophenol (**31**) according to the general procedure for the Michael addition of phenol with acrylates. Purification via silica gel column chromatography (0–5% EtOAc in hexane) yielded **32** as an off-white solid (2.30 g, 38.9% yield). ^1^H NMR (400 MHz, CDCl_3_): *δ* 7.37 (d, *J* = 8.6 Hz, 2H), 6.79 (d, *J* = 8.7 Hz, 2H), 4.24–4.15 (m, 4H), 2.77 (t, *J* = 6.4 Hz, 2H), 1.27 (t, *J* = 7.1 Hz, 3H); HRMS (ESI): [M + H]^+^ calculated for C_10_H_12_BrO_3_ 258.9970; the mass was not observed.

##### 3-(4-Bromophenoxy)propanoic acid (**33**)

The compound was prepared from ethyl 3-(4-bromophenoxy)propanoate (**32**) according to the general procedure for ester hydrolysis, yielding **33** as an off-white solid (1.50 g, 69.0% yield). ^1^H NMR (400 MHz, CDCl_3_): *δ* 7.38 (d, *J* = 9.2 Hz, 2H), 6.79 (d, *J* = 8.8 Hz, 2H), 4.22 (t, *J* = 6.2 Hz, 2H), 2.82 (t, *J* = 6.2 Hz, 2H); HRMS (ESI): [M + H]^+^ calculated for C_9_H_10_BrO_3_ 248.9813; the mass was not observed.

##### 3-(4-Bromophenoxy)-*N*-(3-(trifluoromethoxy)phenyl)propanamide (**34**)

The compound was prepared from 3-(4-bromophenoxy)propanoic acid (**33**) according to the general acid–amine coupling procedure. Purification via silica gel column chromatography (0–20% EtOAc in hexane) yielded **34** as an off-white solid (0.31 g, 62.7% yield). ^1^H NMR (400 MHz, CDCl_3_): *δ* 8.25 (s, 1H), 7.57 (s, 1H), 7.35 (dd, *J* = 6.7, 2.3 Hz, 3H), 7.27 (dd, *J* = 10.8, 5.1 Hz, 1H), 6.95 (d, *J* = 8.0 Hz, 1H), 6.76 (d, *J* = 8.9 Hz, 2H), 4.25 (t, *J* = 5.9 Hz, 2H), 2.80 (t, *J* = 5.9 Hz, 2H); HRMS (ESI): [M + H]^+^ calculated for C_16_H_14_BrF_3_NO_3_ 404.0109, found 404.0096.

##### 3-([1,1′-Biphenyl]-4-yloxy)-*N*-(3-(trifluoromethoxy)phenyl)propanamide (**35a**)

The compound was prepared from 3-(4-bromophenoxy)-*N*-(3-(trifluoromethoxy)phenyl)propanamide (**34**) according to the general Suzuki coupling procedure. Purification via silica gel column chromatography (0–25% EtOAc in hexane) yielded (**35a**) as a white solid (0.05 g, 60.4% yield). Mp: 132–134 °C. ^1^H NMR (400 MHz, CDCl_3_): *δ* 7.87–7.84 (s, 1H), 7.60 (s, 1H), 7.55 (d, *J* = 8.7 Hz, 4H), 7.42 (t, *J* = 7.4 Hz, 2H), 7.35 (s, 3H), 7.03 (d, *J* = 8.6 Hz, 2H), 6.99–6.96 (m, 1H), 4.39 (d, *J* = 5.5 Hz, 2H), 2.88 (s, 2H); ^13^C NMR (151 MHz, CDCl_3_): *δ* 168.97, 157.46, 149.55, 140.50, 139.11, 134.80, 130.01, 128.76, 128.37, 126.87, 126.75, 121.26, 119.56, 117.77, 116.43, 114.88, 112.67, 64.13, 37.69; ^19^F NMR (376 MHz, CDCl_3_): *δ* -57.78; HRMS (ESI): [M + H]^+^ calculated for C_22_H_19_F_3_NO_3_ 402.1317, found 402.1312; RP-HPLC purity ≥ 99.8%, *t*_R_ = 22.8 min.

##### 3-((4′-Fluoro-[1,1′-biphenyl]-4-yl)oxy)-*N*-(3-(trifluoromethoxy)phenyl)propanamide (**35b**)

The compound was prepared from 3-(4-bromophenoxy)-*N*-(3-(trifluoromethoxy)phenyl)propanamide (**34**) according to the general Suzuki coupling procedure. Purification via silica gel column chromatography (0–25% EtOAc in hexane) yielded (**35b**) as a white solid (0.04 g, 41.5% yield). Mp: 101–103 °C. ^1^H NMR (400 MHz, CDCl_3_): *δ* 7.84 (s, 1H), 7.60 (s, 1H), 7.49 (dt, *J* = 8.9, 2.7 Hz, 4H), 7.34 (dt, *J* = 16.0, 8.2 Hz, 2H), 7.11 (t, *J* = 8.7 Hz, 2H), 7.00 (dd, *J* = 15.4, 8.0 Hz, 3H), 4.39 (t, *J* = 5.8 Hz, 2H), 2.88 (t, *J* = 5.7 Hz, 2H); ^13^C NMR (100 MHz, CDCl_3_): *δ* 169.03, 163.36, 160.96, 157.50, 149.61, 139.14, 136.64, 133.75, 130.39, 128.56, 127.88, 121.69, 119.13, 117.49, 116.51, 115.68, 115.01, 114.20, 113.00, 112.38, 64.10, 37.62; ^19^F NMR (376 MHz, CDCl_3_): *δ* -57.78, -116.36; HRMS (ESI): [M + H]^+^ calculated for C_22_H_18_F_4_NO_3_ 420.1223, found 420.1216; RP-HPLC purity ≥ 97.6%, *t*_R_ = 23.0 min.

##### Methyl 3-(naphthalen-2-yloxy)propanoate (**37a**)

The compound was prepared from naphthalen-2-ol (**36a**) according to the general procedure for the Michael addition of phenol with acrylates. Purification via silica gel column chromatography (0–5% EtOAc in hexane) yielded **37a** as an off-colorless liquid (0.50 g, 31.4% yield). ^1^H NMR (400 MHz, CDCl_3_): *δ* 7.76 (d, *J* = 7.8 Hz, 1H), 7.73 (d, *J* = 6.1 Hz, 2H), 7.46–7.41 (m, 1H), 7.36–7.31 (m, 1H), 7.16 (d, *J* = 2.3 Hz, 1H), 7.13 (dd, *J* = 8.8, 2.5 Hz,1H), 4.38 (t, *J* = 6.5 Hz, 2H), 3.75 (s, 3H), 2.88 (t, *J* = 6.5 Hz, 2H); HRMS (ESI): [M + H]^+^ calculated for C_14_H_15_O_3_ 231.1021, found 231.1015.

##### Methyl 3-((7-bromonaphthalen-2-yl)oxy)propanoate (**37b**)

The compound was prepared from 7-bromonaphthalen-2-ol (**36b**) according to the general procedure for the Michael addition of phenol with acrylates. Purification via silica gel column chromatography (0–5% EtOAc in hexane) yielded **37b** as an off-colorless liquid (0.43 g, 31.1% yield). ^1^H NMR (400 MHz, CDCl_3_): *δ* 7.89 (s, 1H), 7.70 (d, *J* = 9.6 Hz, 1H), 7.62 (d, *J* = 7.7 Hz, 1H), 7.41 (d, *J* = 8.6 Hz, 1H), 7.14 (d, *J* = 6.7 Hz, 1H), 7.06 (s, 1H), 4.36 (t, *J* = 5.1 Hz, 2H), 3.76 (s, 3H), 2.88 (t, *J* = 6.1 Hz, 2H); HRMS (ESI): [M + H]^+^ calculated for C_14_H_14_BrO_3_ 309.0126; the mass was not observed.

##### Methyl 3-((6-bromonaphthalen-2-yl)oxy)propanoate (**37c**)

The compound was prepared from 6-bromonaphthalen-2-ol (**36c**) according to the general procedure for the Michael addition of phenol with acrylates. Purification via silica gel column chromatography (0–5% EtOAc in hexane) yielded **37c** as an off-colorless liquid (0.53 g, 38.9% yield). ^1^H NMR (400 MHz, CDCl_3_): *δ* 7.92 (s, 1H), 7.64 (d, *J* = 8.8 Hz, 1H), 7.60 (d, *J* = 8.8 Hz, 1H), 7.50 (d, *J* = 9.1 Hz, 1H), 7.14 (d, *J* = 15.2 Hz, 2H), 4.36 (t, *J* = 6.4 Hz, 2H), 3.75 (s, 3H), 2.88 (t, *J* = 6.4 Hz, 2H); HRMS (ESI): [M + H]^+^ calculated for C_14_H_14_BrO_3_ 309.0126; the mass was not observed.

##### Ethyl 3-((6-fluoronaphthalen-2-yl)oxy)propanoate (**37d**)

The compound was prepared from 6-fluoronaphthalen-2-ol (**36d**) according to the general procedure for the Michael addition of phenol with acrylates. Purification via silica gel column chromatography (0–5% EtOAc in hexane) yielded **37d** as an off-white solid (0.27 g, 16.8% yield). ^1^H NMR (400 MHz, CDCl_3_): *δ* 7.72–7.69 (m, 1H), 7.67 (d, *J* = 9.5 Hz, 1H), 7.38 (dd, *J* = 9.8, 2.6 Hz, 1H), 7.22 (td, *J* = 8.8, 2.6 Hz, 1H), 7.18–7.15 (m, 2H), 4.36 (t, *J* = 6.5 Hz, 2H), 4.21 (q, *J* = 7.1 Hz, 2H), 2.86 (t, *J* = 6.4 Hz, 2H), 1.29 (t, *J* = 7.1 Hz, 3H); HRMS (ESI): [M + H]^+^ calculated for C_15_H_16_FO_3_ 263.1083, found 263.1090.

##### 3-(Naphthalen-2-yloxy)propanoic acid (**38a**)

The compound was prepared from methyl 3-(naphthalen-2-yloxy)propanoate (**37a**) according to the general procedure for ester hydrolysis, yielding **38a** as an off-white solid (0.35 g, 74.6% yield). ^1^H NMR (400 MHz, DMSO-*d_6_*): *δ* 7.73 (dd, *J* = 8.4, 5.0 Hz, 1H), 7.44 (t, *J* = 8.0 Hz, 1H), 7.38–7.30 (m, 3H), 7.23 (t, *J* = 7.0 Hz, 1H), 7.12 (dd, *J* = 8.9, 2.5 Hz, 1H), 4.27 (t, *J* = 6.1 Hz, 2H), 2.75 (t, *J* = 6.1 Hz, 2H); the acid proton was not observed; HRMS (ESI): [M + H]^+^ calculated for C_13_H_13_O_3_ 217.0865, found 217.0859.

##### 3-((7-Bromonaphthalen-2-yl)oxy)propanoic acid (**38b**)

The compound was prepared from methyl 3-((7-bromonaphthalen-2-yl)oxy)propanoate (**37b**) according to the general procedure for ester hydrolysis, yielding **38b** as an off-white solid (0.43 g, 31.1% yield). ^1^H NMR (400 MHz, DMSO-*d_6_*): *δ* 8.09 (s, 1H), 7.85 (d, *J* = 8.9 Hz, 1H), 7.81 (d, *J* = 8.9 Hz, 1H), 7.46 (d, *J* = 8.4 Hz, 1H), 7.37 (s, 1H), 7.18 (d, *J* = 10.8 Hz, 1H), 4.28 (t, *J* = 5.9 Hz, 2H), 2.78 (t, *J* = 5.9 Hz, 2H); the acid proton was not observed; HRMS (ESI): [M + H]^+^ calculated for C_13_H_12_BrO_3_ 294.9970; the mass was not observed.

##### 3-((6-Bromonaphthalen-2-yl)oxy)propanoic acid (**38c**)

The compound was prepared from methyl 3-((6-bromonaphthalen-2-yl)oxy)propanoate (**37c**) according to the general procedure for ester hydrolysis, yielding **38c** as an off-white solid (0.23 g, 53.6% yield). ^1^H NMR (400 MHz, DMSO-*d_6_*): *δ* 8.01 (s, 1H), 7.79 (d, *J* = 9.9 Hz, 1H), 7.63 (d, *J* = 8.8 Hz, 1H), 7.54 (d, *J* = 8.8 Hz, 1H), 7.37 (s,1H), 7.10 (d, *J* = 7.7 Hz, 1H), 4.25 (t, *J* = 6.0 Hz, 2H), 2.74 (t, *J* = 6.0 Hz, 2H); the acid proton was not observed; HRMS (ESI): [M + H]^+^ calculated for C_13_H_12_BrO_3_ 294.9970; the mass was not observed.

##### 3-((6-Fluoronaphthalen-2-yl)oxy)propanoic acid (**38d**)

The compound was prepared from ethyl 3-((6-fluoronaphthalen-2-yl)oxy)propanoate (**37d**) according to the general procedure for ester hydrolysis, yielding **38d** as an off-white solid (0.20 g, 86.2% yield). ^1^H NMR (400 MHz, DMSO-*d_6_*): *δ* 9.72 (s, 1H), 7.77(d, 1H), 7.72 (d, 1H), 7.41 (d, *J* = 5.7 Hz, 1H), 7.20 (d, *J* = 8.9 Hz, 1H), 7.17–7.10 (m, 2H), 4.28 (t, *J* = 6.1 Hz, 2H), 2.77 (t, *J* = 6.1 Hz, 2H); HRMS (ESI): [M + H]^+^ calculated for C_13_H_12_FO_3_ 235.0770; the mass was not observed.

##### 3-(Naphthalen-2-yloxy)-*N*-(3-(trifluoromethoxy)phenyl)propanamide (**39a**)

The compound was prepared from 3-(naphthalen-2-yloxy)propanoic acid (**38a**) according to the general acid–amine coupling procedure. Purification via silica gel column chromatography (0–20% EtOAc in hexane) yielded **39a** as an off-ivory solid (0.15 g, 27.8% yield). Mp: 82–85 °C. ^1^H NMR (400 MHz, CDCl_3_): *δ* 7.87 (s, 1H), 7.78 (d, *J* = 8.7 Hz, 2H), 7.74 (d, *J* = 8.4 Hz, 1H), 7.60 (s, 1H), 7.46 (t, *J* = 6.9 Hz, 1H), 7.37 (s, 1H), 7.35 (s, 1H), 7.33 (d, *J* = 7.8 Hz, 1H), 7.21 (s, 1H), 7.17 (dd, J = 8.9, 2.5 Hz, 1H), 6.97 (d, *J* = 7.1 Hz, 1H), 4.48 (t, *J* = 5.8 Hz, 2H), 2.93 (t, *J* = 5.8 Hz, 2H); ^13^C NMR (100 MHz, CDCl_3_): *δ* 169.09, 155.86, 149.57, 139.14, 134.41, 130.34, 129.53, 129.25, 126.95, 126.27, 124.43, 123.70, 121.70, 119.14, 118.56, 118.18, 117.47, 116.51, 113.02, 107.53, 63.93, 37.55; ^19^F NMR (376 MHz, CDCl_3_): *δ* -57.78; HRMS (ESI): [M + H]^+^ calculated for C_20_H_17_F_3_NO_3_ 376.1161, found 376.1163; RP-HPLC purity ≥ 97.9%, *t*_R_ = 21.5 min.

##### 3-((7-Bromonaphthalen-2-yl)oxy)-*N*-(3-trifluoromethoxy)phenyl)propanamide (**39b**)

The compound was prepared from 3-((7-bromonaphthalen-2-yl)oxy)propanoic acid (**38b**) according to the general acid–amine coupling procedure. Purification via silica gel column chromatography (0–20% EtOAc in hexane) yielded **39b** as an off-ivory solid (0.02 g, 17.5% yield). Mp: 113–115 °C. ^1^H NMR (400 MHz, CDCl_3_): *δ* 7.90 (s, 1H), 7.74 (d, *J* = 8.9 Hz, 2H), 7.66–7.59 (m, 2H), 7.43 (d, *J* = 8.4 Hz, 1H), 7.34 (d, *J* = 8.7 Hz, 2H), 7.16 (d, *J* = 8.6 Hz, 1H), 7.11 (s, 1H), 6.98 (d, *J* = 8.4 Hz, 1H), 4.46 (t, *J* = 5.7 Hz, 2H), 2.93 (t, *J* = 5.8 Hz, 2H); ^13^C NMR (150 MHz, CDCl_3_): *δ* 169.02, 156.68, 149.53, 139.01, 135.61, 130.03, 129.65, 129.26, 128.79, 127.58, 127.33, 121.25, 120.78, 118.74, 117.89, 116.54, 112.74, 106.23, 63.90, 31.58; ^19^F NMR (376 MHz, CDCl_3_): *δ* -57.78; HRMS (ESI): [M + H]^+^ calculated for C_20_H_16_BrF_3_NO_3_ 454.0266, found 454.0254; RP-HPLC purity ≥ 98.8%, *t*_R_ = 24.3 min.

##### 3-((6-Bromonaphthalen-2-yl)oxy)-*N*-(3-(trifluoromethoxy)phenyl)propanamide (**39c**)

The compound was prepared from 3-((6-bromonaphthalen-2-yl)oxy)propanoic acid (**38c**) according to the general acid–amine coupling procedure. Purification via silica gel column chromatography (0–20% EtOAc in hexane) yielded **39c** as an off-white solid (0.06 g, 24.7% yield). Mp: 128–130 °C. ^1^H NMR (400 MHz, CDCl_3_): *δ* 7.93 (s, 1H), 7.76 (s, 1H), 7.68 (d, *J* = 8.6 Hz, 1H), 7.61 (d, *J* = 8.3 Hz, 2H), 7.52 (d, *J* = 8.7 Hz, 1H), 7.34 (d, *J* = 7.9 Hz, 2H), 7.17 (s, 2H), 6.98 (d, *J* = 8.4 Hz, 1H), 4.46 (t, *J* = 5.7 Hz, 2H), 2.92 (t, *J* = 5.8 Hz, 2H); ^13^C NMR (100 MHz, CDCl_3_): *δ* 168.73, 156.19, 149.54, 139.00, 132.85, 130.27, 130.01, 129.76, 129.55, 128.93, 128.67, 128.62, 128.30, 119.45, 119.10, 117.54, 116.50, 107.20, 63.95, 37.56; ^19^F NMR (376 MHz, CDCl_3_): *δ* -57.79; HRMS (ESI): [M + H]^+^ calculated for C_20_H_16_BrF_3_NO_3_ 454.0266, found 454.0251; RP-HPLC purity ≥ 98.2%, *t*_R_ = 24.4 min.

##### 3-((6-Fluoronaphthalen-2-yl)oxy)-*N*-(3-(trifluoromethoxy)phenyl)propanamide (**39d**)

The compound was prepared from 3-((6-fluoronaphthalen-2-yl)oxy)propanoic acid (**38d**) according to the general acid–amine coupling procedure. Purification via silica gel column chromatography (0–20% EtOAc in hexane) yielded **39d** as an off-white solid (0.04 g, 26.0% yield). Mp: 102–104 °C. ^1^H NMR (400 MHz, CDCl_3_): *δ* 7.78 (s, 1H), 7.73 (d, *J* = 4.6 Hz, 1H), 7.71 (d, *J* = 4.3 Hz, 1H), 7.60 (s, 1H), 7.40 (d, *J* = 7.9 Hz, 1H), 7.35 (s, 1H), 7.32 (d, *J* = 7.9 Hz, 1H), 7.21 (s, 2H), 7.19 (s, 1H), 6.98 (d, *J* = 8.1 Hz, 1H), 4.46 (t, *J* = 6.0 Hz, 2H), 2.92 (t, *J* = 5.7 Hz, 2H); ^13^C NMR (100 MHz, cdcl3): *δ* 168.81, 160.73, 158.31, 155.36–155.35, 149.58, 139.02, 131.22, 129.05, 128.82, 119.44, 119.32, 117.84, 117.01, 116.48, 112.74, 112.53, 110.85, 107.46, 64.01, 37.62; ^19^F NMR (376 MHz, CDCl_3_): *δ* -57.78, -117.6; HRMS (ESI): [M + H]^+^ calculated for C_20_H_16_F_4_NO_3_ 394.1066, found 394.1060; RP-HPLC purity ≥ 97.0%, *t*_R_ = 22.1 min.

##### *tert*-Butyl(2-bromoethyl)carbamate (**41**)

To a solution of 2-bromoethanamine HBr salt (**40**) (3.00 g, 14.7 mmol) in DCM (20 mL), we added Et_3_N (4.12 mL, 29.5 mmol) followed by Boc anhydride (4.07 mL, 17.7 mmol). The resulting mixture was stirred at room temperature for 12 h. The reaction mixture was diluted with ice-cold water (100 mL), and the aqueous phase was extracted using DCM (3 × 70 mL). The combined organic layers were dried over anhydrous sodium sulfate, filtered, and evaporated under reduced pressure. The residue was purified using silica gel column chromatography (0–10% EtOAc in hexane) to yield **41** as an off-colorless liquid (2.5 g, 78.0% yield). ^1^H NMR (400 MHz, CDCl_3_): *δ* 4.94 (br s, 1H), 3.67 (t, *J* = 8.2 Hz, 2H), 3.45 (t, *J* = 7.2 Hz, 2H), 1.45 (s, 9H); HRMS (ESI): [M + H]^+^ calculated for C_7_H_15_BrNO_2_ 224.0286, found 224.0288.

##### *tert*-Butyl(2-(4-(2,4,4-trimethylpentan-2-yl)phenoxy)ethyl)carbamate (**42**)

To a solution of 4-(2,4,4-trimethylpentan-2-yl)phenol (**1**) (0.25 g, 12.1 mmol) in acetone 50 mL, we added potassium carbonate (K_2_CO_3_; 5.02 g, 36.3 mmol) followed by *tert*-butyl(2-bromoethyl)carbamate (**41**) (3.25 g, 14.5 mmol). The resulting mixture was stirred at 80 °C for 12 h. The reaction mixture was cooled to room temperature and evaporated under reduced pressure to remove acetone. The residue was diluted with water (50 mL), and the aqueous phase was extracted using EtOAc (3 × 50 mL). The combined organic layers were dried over anhydrous sodium sulfate, filtered, and evaporated under reduced pressure. The residue was purified using silica gel column chromatography (0–15% EtOAc in hexane) to yield **42** as an off-white solid (2.00 g, 47.2% yield). ^1^H NMR (400 MHz, CDCl_3_): *δ* 7.22 (d, *J* = 7.2 Hz, 2H), 6.80 (d, *J* = 8.4 Hz, 2H), 4.03 (t, *J* = 19.4 Hz, 2H), 3.52 (d, *J* = 5.2 Hz, 2H), 1.70 (s, 2H), 1.45 (s, 9H), 1.33 (s, 6H), 0.71 (s, 9H); HRMS (ESI): [M + H]^+^ calculated for C_21_H_35_NO_3_ 350.2695, found 350.2685.

##### 2-(4-(2,4,4-Trimethylpentan-2-yl)phenoxy)ethanamine (**43**)

To a solution of *tert*-butyl (2-(4-(2,4,4-trimethylpentan-2-yl)phenoxy)ethyl)carbamate (**42**) (2.0 g, 5.72 mmol) in DCM (25 mL), we added TFA (10 mL) at room temperature. The resulting mixture was stirred at room temperature for 12 h. The reaction mixture was evaporated under reduced pressure. The residue was purified using silica gel column chromatography (0–80% EtOAc in hexane) to yield **43** as an off-white solid (1.1 g, 77.1% yield). ^1^H NMR (400 MHz, DMSO): *δ* 7.97 (d, *J* = 808 Hz, 2H), 7.31 (d, *J* = 8.0 Hz, 2H), 4.13 (t, *J* = 2.6 Hz, 2H), 3.22 (d, *J* = 2.4 Hz, 2H), 1.69 (s, 2H), 1.30 (s, 6H), 0.67 (s, 9H); HRMS (ESI): [M + H]^+^ calculated for C_16_H_28_NO 250.2171, found 250.2162.

##### 3-(Trifluoromethoxy)-*N*-(2-(4-(2,4,4-trimethylpentan-2-yl)phenoxy)ethyl)benzamide (**44a**)

To a solution of 2-(4-(2,4,4-trimethylpentan-2-yl)phenoxy)ethanamine (**43**) (0.2 g, 0.80 mmol), and Et_3_N (0.33 mL, 2.40 mmol) in DCM (20 mL), we added 3-(trifluoromethoxy)benzoyl chloride (0.21 g, 0.96 mmol). The resulting mixture was stirred at room temperature for 12 h. The reaction mixture was diluted with ice-cold water (20 mL), and the aqueous phase was extracted using DCM (3 × 20 mL). The combined organic layers were dried over anhydrous sodium sulfate, filtered, and evaporated under reduced pressure. The residue was purified using silica gel column chromatography (0–20% EtOAc in hexane) to yield **44a** as an off-white solid (0.085 g, 24.2% yield). Mp: 65–67 °C. ^1^H NMR (400 MHz, CDCl_3_): *δ* 7.68 (d, *J* = 7.2 Hz, 2H), 7.47 (t, *J* = 8.2 Hz, 1H), 7.36 (d, *J* = 7.9 Hz, 1H), 7.29 (d, *J* = 8.4 Hz, 2H), 6.84 (d, *J* = 8.6 Hz, 2H), 6.59 (s, 1H), 4.15 (t, *J* = 4.8 Hz, 2H), 3.88 (dd, *J* = 10.3, 5.1 Hz, 2H), 1.70 (s, 2H), 1.34 (s, 5H), 0.71 (s, 8H); ^13^C NMR (100 MHz, CDCl_3_): *δ* 166.10, 155.98, 149.36, 142.97, 136.47, 130.03, 127.19, 125.07, 123.82, 121.64, 120.02, 119.04, 113.61, 66.47, 56.89, 39.75, 37.95, 32.28, 31.74, 31.64; ^19^F NMR (376 MHz, CDCl_3_): *δ* -57.84; HRMS (ESI): [M + H]^+^ calculated for C_24_H_31_F_3_NO_3_ 438.2256, found 438.2250; RP-HPLC purity ≥ 98.0%, *t*_R_ = 28.1 min.

##### 3-(Trifluoromethoxy)-*N*-(2-(4-(2,4,4-trimethylpentan-2-yl)phenoxy)ethyl)benzenesulfonamide (**44b**)

To a solution of 2-(4-(2,4,4-trimethylpentan-2-yl)phenoxy)ethanamine (**43**) (0.2 g, 0.80 mmol), and Et_3_N (0.33 mL, 2.40 mmol) in DCM (20 mL), we added 3-(trifluoromethoxy)benzenesulfonyl chloride (0.25 g, 0.96 mmol). The resulting mixture was stirred at room temperature for 12 h. The reaction mixture was diluted with ice-cold water (20 mL), and the aqueous phase was extracted using DCM (3 × 20 mL). The combined organic layers were dried over anhydrous sodium sulfate, filtered, and evaporated under reduced pressure. The residue was purified using silica gel column chromatography (0–20% EtOAc in hexane) to yield **44b** as an off-colorless oil (0.03 g, 7.90% yield). ^1^H NMR (400 MHz, CDCl_3_): *δ* 7.82 (d, *J* = 8.0 Hz, 1H), 7.75 (s, 1H), 7.56 (t, *J* = 8.0 Hz, 1H), 7.42 (d, *J* = 7.7 Hz, 1H), 7.24 (s, 1H), 6.71 (d, *J* = 8.4 Hz, 2H), 5.01 (s, 1H), 3.99 (t, *J* = 4.7 Hz, 2H), 3.40 (dd, *J* = 10.6, 5.4 Hz, 2H), 1.69 (s, 2H), 1.33 (s, 6H), 0.70 (s, 9H); ^13^C NMR (100 MHz, CDCl_3_): *δ* 155.44, 149.35, 143.21, 142.14, 130.85, 127.18, 125.21, 125.03, 119.68, 113.49, 65.95, 56.89, 42.74, 37.95, 32.29, 31.73, 31.63; ^19^F NMR (376 MHz, CDCl_3_): *δ* -57.95; HRMS (ESI): [M + H]^+^ calculated for C_23_H_31_F_3_NO_4_S 474.1926, found 474.1931; RP-HPLC purity ≥ 99.9%, *t*_R_ = 28.1 min.

##### 4-(2,4,4-Trimethylpentan-2-yl)phenyl trifluoromethanesulfonate (**45**)

To a solution of 4-(2,4,4-trimethylpentan-2-yl)phenol (**1**) (15.0 g, 72.7 mmol) in DCM (20 mL), we added Et_3_N (3.4 mL, 87.2 mmol). The reaction mixture was cooled to 0 °C, and trifluoromethanesulfonic anhydride (14.6 mL, 87.2 mmol) was added dropwise to the stirring solution. The resulting mixture was stirred at the same temperature for 1 h. The reaction mixture was diluted with ice-cold water (100 mL), and the aqueous phase was extracted using DCM (3 × 70 mL). The combined organic layers were dried over anhydrous sodium sulfate, filtered, and evaporated under reduced pressure. The residue was purified using silica gel column chromatography (0–1% EtOAc in hexane) to yield **45** as an off-colorless oil (20.5 g, 83.3% yield). ^1^H NMR (400 MHz, CDCl_3_): *δ* 7.43 (d, *J* = 8.4 Hz, 2H), 7.17 (d, *J* = 9.2 Hz, 2H), 1.74 (s, 2H), 1.37 (s, 6H), 0.70 (s, 9H); HRMS (ESI): [M + H]^+^ calculated for C1_5_H_22_F_3_O_3_S 339.1242; the mass was not observed.

##### Methyl 1-(4-(2,4,4-trimethylpentan-2-yl)phenyl)piperidine-4-carboxylate (**46**)

To a solution of 4-(2,4,4-trimethylpentan-2-yl)phenyl trifluoromethanesulfonate (**45**) (3.0 g, 8.86 mmol), methyl piperidine-4-carboxylate (1.9 g, 10.6 mmol) in toluene (30 mL), we added Cs_2_CO_3_ (8.67 g, 26.5 mmol). The mixture was degassed with argon for 10 min, and 2-(dicyclohexylphosphino)-2′,4′,6′-triisopropylbiphenyl (Xphos; 0.84 g, 1.77 mmol) and tris(dibenzylideneacetone)dipalladium (Pd_2_(dba)_3_; 1.62 g, 1.77 mmol) were added. The resulting mixture was stirred at 110 °C for 12 h in a sealed tube. The reaction mixture was cooled to room temperature, diluted with EtOAc (50 mL), and filtered through a celite bed. The filtrate was evaporated under reduced pressure. The residue was purified using silica gel column chromatography (0–7% EtOAc in hexane) to yield **46** as an off-white solid (1.74 g, 59.3% yield). ^1^H NMR (400 MHz, CDCl_3_): *δ* 7.25 (d, *J* = 4.6 Hz, 2H), 6.86 (d, *J* = 8.7 Hz, 2H), 3.70 (s, 3H), 3.61 (d, *J* = 12.3 Hz, 2H), 2.74 (td, *J* = 11.9, 2.5 Hz, 2H), 2.06–1.99 (m, 2H), 1.89 (dt, *J* = 21.0, 7.5 Hz, 2H), 1.69 (s, 2H), 1.33 (s, 6H), 0.71 (s, 9H); HRMS (ESI): [M + H]^+^ calculated for C_21_H_34_NO_2_ 332.2590, found 332.2577.

##### 1-(4-(2,4,4-Trimethylpentan-2-yl)phenyl)piperidine-4-carboxylic acid (**47**)

The compound was prepared from methyl 1-(4-(2,4,4-trimethylpentan-2-yl)phenyl)piperidine-4-carboxylate (**46**) according to the general procedure for ester hydrolysis, yielding **47** as an off-white solid (1.58 g, 99.1% yield). ^1^H NMR (400 MHz, DMSO-*d_6_*) *δ* 7.19 (d, *J* = 8.8 Hz, 2H), 6.83 (d, *J* = 8.8 Hz, 2H), 3.56 (d, *J* = 9.1 Hz, 2H), 2.67 (t, *J* = 13.0 Hz, 2H), 1.88 (d, *J* = 10.8 Hz, 2H), 1.66 (s, 2H), 1.60 (dd, *J* = 17.8, 6.6 Hz, 2H), 1.28 (s, 6H), 0.68 (s 9H); the acid proton was not observed; HRMS (ESI): [M + H]^+^ calculated for C_20_H_33_NO_2_ 318.2433, found 318.2417.

##### *tert*-Butyl 4-(4-(2,4,4-trimethylpentan-2-yl)phenyl)piperazine-1-carboxylate (**48**)

To a solution of 4-(2,4,4-trimethylpentan-2-yl)phenyl trifluoromethanesulfonate (**45**) (1.0 g, 2.95 mmol), 1-boc-piperazine (0.66 g, 3.54 mmol) in 30 mL of toluene, we added Cs_2_CO_3_ (2.88 g, 8.86 mmol). The mixture was degassed with argon for 10 min, and Xphos (0.28 g, 0.59 mmol) and Pd_2_(dba)_3_ (0.54 g, 0.59 mmol) were added. The resulting mixture was stirred at 110 °C for 12 h in a sealed tube. The reaction mixture was cooled to room temperature, diluted with EtOAc (50 mL), and filtered through a celite bed. The filtrate was evaporated under reduced pressure. The residue was purified using silica gel column chromatography (0–15% EtOAc in hexane) to yield **48** as an off-white solid (1.03 g, 93.2% yield). ^1^H NMR (400 MHz, CDCl_3_): *δ* 7.26 (d, 2H), 6.85 (d, *J* = 8.7 Hz, 2H), 3.60–3.54 (m, 4H), 3.13–3.06 (m, 4H), 1.69 (s, 2H), 1.48 (s, 9H), 1.33 (s, 6H), 0.71 (s, 9H); HRMS (ESI): [M + H]^+^ calculated for C_23_H_39_N_2_O_2_ 375.3012; the mass was not observed.

##### 1-(4-(2,4,4-Trimethylpentan-2-yl)phenyl)piperazine (**49**)

To a solution of *tert*-butyl 4-(4-(2,4,4-trimethylpentan-2-yl)phenyl)piperazine-1-carboxylate (**48**) (1.0 g) in DCM (30 mL), we added TFA (5 mL) at room temperature. The resulting mixture was stirred at room temperature for 12 h. The reaction mixture was evaporated under reduced pressure. The residue was purified using silica gel column chromatography (0–10% EtOAc in hexane) to yield **49** as an off-white solid (0.61 g, 83.3% yield). ^1^H NMR (400 MHz, DMSO-*d_6_*): *δ* 8.86 (s, 1H), 7.25 (d, *J* = 8.7 Hz, 2H), 6.89 (d, *J* = 8.7 Hz, 2H), 3.27 (dd, *J* = 13.2 Hz, 4H), 3.24 (s, *J* = 13.2 Hz, 4H), 1.68 (s, 2H), 1.29 (s, 6H), 0.68 (s, 9H); HRMS (ESI): [M + H]^+^ calculated for C_18_H_31_N_2_ 275.2487, found 275.2475.

##### *N*-(3-(Trifluoromethoxy)phenyl)-1-(4-(2,4,4-trimethylpentan-2-yl)phenyl)piperidine-4-carboxamide (**50**)

The compound was prepared from 1-(4-(2,4,4-trimethylpentan-2-yl)phenyl)piperidine-4-carboxylic acid (**47**) according to the general acid–amine coupling procedure. Purification via silica gel column chromatography (0–20% EtOAc in hexane) yielded **50** as an off-white solid (0.11 g, 59.0% yield). Mp: 139–140 °C. ^1^H NMR (400 MHz, DMSO-*d_6_*): *δ* 10.26 (s, 1H), 7.84 (s, 1H), 7.50 (d, *J* = 7.3 Hz, 1H), 7.43 (t, *J* = 8.2 Hz, 1H), 7.21 (d, *J* = 8.8 Hz, 2H), 7.02 (d, *J* = 9.2 Hz, 1H), 6.87 (d, *J* = 8.8 Hz, 2H), 3.72 (d, *J* = 12.5 Hz, 2H), 2.64 (t, *J* = 11.8 Hz, 2H), 1.88 (d, *J* = 14.9 Hz, 2H), 1.75 (t, *J* = 10.4 Hz, 2H), 1.67 (s, 2H), 1.28 (s, 6H), 0.69 (s, 9H); ^13^C NMR (100 MHz, DMSO): *δ* 174.34, 149.11, 148.90, 141.42, 140.09, 130.82, 126.80, 121.78, 119.23, 118.02, 116.68, 115.80, 115.48, 111.50, 56.71, 48.95, 43.24, 37.82, 32.45, 32.02, 31.93, 28.48; ^19^F NMR (376 MHz, CDCl_3_): *δ* -57.78; HRMS (ESI): [M + H]^+^ calculated for C_27_H_37_F_3_N_2_O_2_ 477.2729, found 477.2726; RP-HPLC purity ≥ 98.3%, *t*_R_ = 16.0 min.

##### (3-(Trifluoromethoxy)phenyl)(4-(4-(2,4,4-trimethylpentan-2-yl)phenyl)piperazin-1-yl)methanone (**51**)

To a solution of 1-(4-(2,4,4-trimethylpentan-2-yl)phenyl)piperazine (**49**) (0.1 g, 0.36 mmol) and Et_3_N (0.15 mL, 1.09 mmol) in DCM (20 mL), we added 3-(trifluoromethoxy)benzoyl chloride (0.1 mL, 0.54 mmol). The resulting mixture was stirred at room temperature for 12 h. After the completion of the reaction, the solvent was evaporated under reduced pressure. The residue was purified using silica gel column chromatography (0–15% EtOAc in hexane) to yield **51** as an off-white solid (0.06 g, 36.0% yield). Mp: 102–104 °C. ^1^H NMR (400 MHz, CDCl_3_): *δ* 7.50–7.45 (m, 1H), 7.38 (d, *J* = 7.7 Hz, 1H), 7.30 (d, *J* = 6.2 Hz, 3H), 7.27 (s, 1H), 6.86 (d, *J* = 8.8 Hz, 2H), 3.94 (s, 2H), 3.57 (s, 2H), 3.18 (d, *J* = 59.8 Hz, 4H), 1.70 (s, 2H), 1.34 (s, 6H), 0.71 (s, 9H); ^13^C NMR (100 MHz, CDCl_3_): *δ* 168.53, 149.14, 148.18, 142.52, 137.52, 126.96, 126.80, 125.54, 125.35, 122.24, 122.05, 121.63, 119.89, 119.72, 119.07, 116.14, 116.10, 77.31, 76.99, 76.68, 56.87, 37.88, 32.29, 31.80, 31.69, 31.55, 31.49.; ^19^F NMR (376 MHz, CDCl_3_): *δ* -57.85; HRMS (ESI): [M + H]^+^ calculated for C_26_H_34_F_3_N_2_O_2_ 463.2572, found 463.2564; RP-HPLC purity ≥ 99.8%, *t*_R_ = 28.2 min.

##### 4-(2,4,4-Trimethylpentan-2-yl)aniline (**53**)

To a solution of 4-(2,4,4-trimethylpentan-2-yl)phenyl trifluoromethanesulfonate (**45**) (5.0 g, 14.7 mmol) and diphenylmethanimine (2.42 mL, 17.7 mmol) in toluene (30 mL), we added Cs_2_CO_3_ (14.4 g, 44.3 mmol). The mixture was degassed with argon for 10 min, and 2,2′-bis(diphenylphosphino)-1,1′-binaphthalene (BINAP; 1.8 g, 2.95 mmol) and bis(dibenzylideneacetone)palladium (Pd(dba)_2_; 1.7 g, 2.95 mmol) were added. The resulting mixture was stirred at 100 °C for 12 h in a sealed tube. The reaction mixture was cooled to room temperature, diluted with EtOAc (20 mL), and filtered through a celite bed. The filtrate was evaporated under reduced pressure to yield crude (**52**). The crude product was dissolved in methanol (40 mL) followed by the addition of 35% HCl (10 mL). The resulting mixture was stirred at 70 °C for 2 h. The reaction mixture was cooled to room temperature, and excess methanol was evaporated under pressure. The obtained crude extract was basified with 100 mL of saturated sodium bicarbonate solution, and the aqueous phase was extracted using EtOAc (3 × 100 mL). The combined organic layers were dried over anhydrous sodium sulfate, filtered, and evaporated under reduced pressure. The residue was purified using silica gel column chromatography (0–20% EtOAc in hexane) to yield **52** as a yellow oil (1.19 g, 39.2% yield). ^1^H NMR (400 MHz, DMSO): *δ* 7.00 (d, *J* = 8.3 Hz, 2H), 6.50 (dd, *J* = 26.5, 8.2 Hz, 2H), 4.76 (s, 2H), 1.61 (s, 2H), 1.24 (s, 6H), 0.67 (s, 9H); HRMS (ESI): [M + H]^+^ calculated for C_14_H_25_N 206.1909, found 206.1903.

##### Methyl 3-((4-(2,4,4-trimethylpentan-2-yl)phenyl)amino)propanoate (**54**)

Methyl 3-bromopropionate (5.00 mL, excess) was added to a stirring solution of 4-(2,4,4-trimethylpentan-2-yl)aniline (**53**) (1.20 g, 5.84 mmol), Cs_2_CO_3_ (5.71 g, 17.5 mmol) and sodium iodide (0.43 g, 2.92 mmol) in acetonitrile (30 mL). The resulting mixture was stirred at 70 °C for 12 h in a sealed tube. The reaction mixture was cooled to room temperature, diluted with EtOAc (20 mL), and filtered through a celite bed. The filtrate was evaporated under reduced pressure. The residue was purified using silica gel column chromatography (0–7% EtOAc in hexane) to yield **54** as a yellow solid (0.61 g, 34.7% yield). ^1^H NMR (400 MHz, CDCl_3_): *δ* 7.17 (d, *J* = 8.6 Hz, 2H), 6.56 (d, *J* = 8.3 Hz, 2H), 3.70 (s, 3H), 3.44 (t, *J* = 6.4 Hz, 2H), 2.62 (t, *J* = 6.4 Hz, 2H), 1.67 (s, 2H), 1.32 (s, 6H), 0.72 (s, 9H); the NH proton was not observed; HRMS (ESI): [M + H]^+^ calculated for C_18_H_30_NO_2_ 292.2277, found 292.2290.

##### 3-((4-(2,4,4-Trimethylpentan-2-yl)phenyl)amino)propanoic acid (**55**)

The compound was prepared from methyl 3-((4-(2,4,4-trimethylpentan-2-yl)phenyl)amino)propanoate (**54**) according to the general procedure for ester hydrolysis, yielding **55** as a white solid (0.56 g, 98.0% yield). ^1^H NMR (400 MHz, DMSO): *δ* 7.53 (d, *J* = 8.1 Hz, 2H), 7.41 (d, *J* = 8.0 Hz, 2H), 3.43 (t, *J* = 7.1 Hz, 2H), 2.71 (t, *J* = 7.1 Hz, 2H), 1.74 (s, 2H), 1.33 (s, 6H), 0.68 (s, 9H); NH, the acid proton was not observed; HRMS (ESI): [M + H]^+^ calculated for C_17_H_28_NO_2_ 278.2120, found 278.2122.

##### *N*-(3-(Trifluoromethoxy)phenyl)-3-((4-(2,4,4-trimethylpentan-2-yl)phenyl)amino)propanamide (**56**)

The compound was prepared from 3-((4-(2,4,4-trimethylpentan-2-yl)phenyl)amino)propanoic acid (**55**) according to the general acid–amine coupling procedure. Purification via silica gel column chromatography (0–25% EtOAc in Hexane) yielded **56** as an off-white oil (0.12 g, 27.7% yield). ^1^H NMR (400 MHz, CDCl_3_): *δ* 8.09 (s, 1H), 7.53 (s, 1H), 7.38–7.31 (m, 1H), 7.28 (s, 1H), 7.22 (d, *J* = 8.1 Hz, 1H), 7.09 (d, *J* = 7.9 Hz, 1H), 6.99–6.90 (m, 1H), 6.66 (d, *J* = 8.6 Hz, 1H), 6.37 (d, *J* = 7.9 Hz, 1H), 5.30 (s, 1H), 3.55 (t, *J* = 5.5 Hz, 2H), 2.72–2.52 (m, 2H), 1.68 (s, 2H), 1.33 (s, 6H), 0.72 (s, 9H); ^13^C NMR (100 MHz, CDCl_3_): *δ* 170.44, 149.49, 144.57, 140.67, 139.17, 130.28, 129.54, 127.40, 126.76, 124.24, 121.68, 119.12, 118.19, 117.46, 117.06, 116.31, 115.55, 113.66, 113.28, 113.00, 112.32, 56.95, 40.61, 37.82, 36.75, 32.31, 31.68; ^19^F NMR (376 MHz, CDCl_3_): *δ* -57.79; HRMS (ESI): [M + H]^+^ calculated for C_24_H_32_F_3_N_2_O_2_ 437.2416, found 437.2401; RP-HPLC purity ≥ 99.9%, *t*_R_ = 19.6 min.

##### 3-(Methyl(4-(2,4,4-trimethylpentan-2-yl)phenyl)amino)-*N*-(3-(trifluoromethoxy)phenyl)propanamide (**57a**)

The compound was prepared from *N*-(3-(trifluoromethoxy)phenyl)-3-((4-(2,4,4-trimethylpentan-2-yl)phenyl)amino)propanamide (**56**) according to the general reductive amination procedure. Purification via silica gel column chromatography (0–20% EtOAc in hexane) yielded ***57*a** as an off-white solid (0.05 g, 55.3% yield). Mp: 101–103 °C. ^1^H NMR (400 MHz, CDCl_3_): *δ* 8.53 (s, 1H), 7.50 (s, 1H), 7.29 (d, *J* = 8.9 Hz, 2H), 7.23 (d, *J* = 8.1 Hz, 1H), 6.93 (d, *J* = 7.1 Hz, 1H), 6.83 (d, *J* = 8.6 Hz, 2H), 3.61 (t, *J* = 6.1 Hz, 2H), 2.92 (s, 3H), 2.62 (t, *J* = 6.1 Hz, 2H), 1.70 (s, 2H), 1.35 (s, 6H), 0.72 (s,9H); ^13^C NMR (100 MHz, CD_3_OD): *δ* 171.88, 149.25, 146.53, 140.21, 138.05, 129.94, 129.36, 126.70, 126.23, 121.75, 119.21, 118.06, 117.43, 116.21, 115.59, 114.74, 113.16, 112.58, 112.35, 111.86, 56.54, 49.17, 37.11, 33.89, 31.68, 30.75; ^19^F NMR (376 MHz, CDCl_3_): *δ* -57.77; HRMS (ESI): [M + H]^+^ calculated for C_25_H_34_F_3_N_2_O_2_ 451.2572, found 451.2571; RP-HPLC purity ≥ 99.7%, *t*_R_ = 19.4 min.

##### 3-(Isopropyl(4-(2,4,4-trimethylpentan-2-yl)phenyl)amino)-*N*-(3-(trifluoromethoxy)phenyl)propanamide (**57b**)

The compound was prepared from *N*-(3-(trifluoromethoxy)phenyl)-3-((4-(2,4,4-trimethylpentan-2-yl)phenyl)amino)propanamide (**56**) according to the general reductive amination procedure. Purification via silica gel column chromatography (0–20% EtOAc in hexane) yielded **57b** as an off-green liquid (0.01 g, 9.10% yield). ^1^H NMR (400 MHz, CDCl_3_): *δ* 10.10 (s, 1H), 7.59 (s, 1H), 7.38 (d, *J* = 8.3 Hz, 1H), 7.32 (d, *J* = 8.5 Hz, 3H), 6.98 (d, *J* = 8.5 Hz, 2H), 6.93 (d, *J* = 8.3 Hz, 1H), 3.63 (dt, *J* = 13.0, 6.5 Hz, 1H), 3.44–3.38 (t, 2H), 2.46–2.41 (t, 2H), 1.70 (s, 2H), 1.36 (s, 6H), 1.13 (d, *J* = 6.6 Hz, 6H), 0.70 (s, 9H); ^13^C NMR (100 MHz, CDCl_3_): *δ* 170.00, 148.66, 144.39, 142.55, 138.93, 129.24, 126.26, 121.34, 120.76, 120.57, 118.20, 116.61, 114.70, 111.50, 56.12, 54.10, 41.55, 37.21, 33.31, 31.38, 30.81, 30.32, 18.70, 0.09; ^19^F NMR (376 MHz, CDCl_3_): *δ* -57.74; HRMS (ESI): [M + H]^+^ calculated for C_27_H_39_F_3_N_2_O_2_ 479.2885, found 479.2873; RP-HPLC purity ≥ 96.1%, *t*_R_ = 15.3 min.

##### 3-(Cyclobutyl(4-(2,4,4-trimethylpentan-2-yl)phenyl)amino)-*N*-(3-(trifluoromethoxy)phenyl)propanamide (**57c**)

The compound was prepared from *N*-(3-(trifluoromethoxy)phenyl)-3-((4-(2,4,4-trimethylpentan-2-yl)phenyl)amino)propanamide (**56**) according to the general reductive amination procedure. Purification via silica gel column chromatography (0–20% EtOAc in hexane) yielded **57c** as an off-colorless liquid (0.04 g, 38.0% yield). ^1^H NMR (400 MHz, CDCl_3_): *δ* 10.10 (s, 1H), 7.62 (s, 1H), 7.41 (d, *J* = 8.3 Hz, 1H), 7.31 (d, *J* = 8.8 Hz, 3H), 6.96 (t, *J* = 7.9 Hz, 3H), 3.71 (dt, *J* = 15.9, 8.1 Hz, 1H), 3.35 (t, *J* = 6.0 Hz, 2H), 2.38 (t, *J* = 6.0 Hz, 2H), 2.11 (dd, *J* = 15.9, 8.0 Hz, 2H), 2.03–1.89 (m, 2H), 1.70 (s, 2H), 1.64 (m, 2H), 1.35 (s, 6H), 0.69 (s, 9H); ^13^C NMR (100 MHz, CDCl_3_): *δ* 170.89, 149.61, 145.84, 143.38, 139.91, 127.38, 126.85, 121.83, 121.68, 121.04, 115.83, 112.43, 111.88, 58.29, 58.09, 57.08, 46.02, 38.19, 34.19, 32.32, 31.74, 31.54, 31.27, 28.56, 14.34; ^19^F NMR (376 MHz, CDCl_3_): *δ* -57.74; HRMS (ESI): [M + H]^+^ calculated for C_28_H_39_F_3_N_2_O_2_ 491.2885, found 491.2888; RP-HPLC purity ≥ 96.3%, *t*_R_ = 16.8 min.

##### *N*-(3-(Trifluoromethoxy)phenyl)-3-(N-(4-(2,4,4-trimethylpentan-2-yl)phenyl)acetamido)propanamide (**57d**)

To a solution of *N*-(3-(trifluoromethoxy)phenyl)-3-((4-(2,4,4-trimethylpentan-2-yl)phenyl)amino)propanamide (**56**) (0.06 g, 0.13 mmol) in DCM (20 mL), we added triethyl amine (0.04 mL, 0.41 mmol) followed by acetyl chloride (0.03 mL, 0.41 mmol). The resulting mixture was stirred at room temperature for 1 h. The reaction was quenched with saturated sodium bicarbonate solution (40 mL), and the aqueous phase was extracted using DCM (3 × 50 mL). The combined organic layers were dried over anhydrous sodium sulfate, filtered, and evaporated under reduced pressure. The residue was purified using silica gel column chromatography (0–15% EtOAc in Hexane) to yield **57d** as an off-white solid (0.04 g, 60.8% yield). Mp: 164–166 °C. ^1^H NMR (400 MHz, CDCl_3_): *δ* 7.72 (d, *J* = 13.1 Hz, 1H), 7.48 (d, *J* = 8.0 Hz, 1H), 7.43 (d, *J* = 8.3 Hz, 1H), 7.39 (d, *J* = 8.4 Hz, 1H), 7.31 (t, *J* = 8.2 Hz, 1H), 7.07 (d, *J* = 8.3 Hz, 2H), 6.95 (d, *J* = 8.2 Hz, 1H), 4.09 (t, *J* = 6.3 Hz, 2H), 2.70 (t, *J* = 6.3 Hz, 2H), 1.87 (s, 2H), 1.74 (s, 3H) 1.38 (s, 6H), 0.71 (s, 9H); amide proton was not observed; ^13^C NMR (100 MHz, CDCl_3_): *δ* 172.34, 168.90, 151.01, 149.48, 139.84, 139.02, 130.10, 128.18, 127.73, 127.52, 127.14, 126.79, 126.22, 121.72, 119.17, 57.04, 45.18, 38.61, 36.82, 32.37, 31.75, 31.58, 31.44, 31.23; ^19^F NMR (376 MHz, CDCl_3_): *δ* -57.74; HRMS (ESI): [M + H]^+^ calculated for C_26_H_33_F_3_N_2_O_3_ 479.2522, found 479.2508; RP-HPLC purity ≥ 99.1%, *t*_R_ = 26.5 min.

### 4.2. Biology

#### 4.2.1. Cell Culture

Human lung carcinoma A549 cells were cultured in Dulbecco’s modified Eagle’s medium and H460 cells were cultured in RPMI 1640 medium containing 10% fetal bovine serum and 1% penicillin/streptomycin at 37 °C with a 5% Co_2_ hypoxia experimental condition. The cells were grown in the hypoxia chamber (1% O_2_, 94% N_2_, and 5% Co_2_ in a multigas incubator)

#### 4.2.2. Cell Growth Inhibition Assay

Cell growth inhibition was assessed using sulforhodamine B (SRB) as described in [34]. The cells were fixed with 10% formalin (Sigma-Aldrich) and stained with 0.4% SRB. Protein-bound dye was dissolved in 10 mM of Tris, and its optical density was measured at 540 nm.

#### 4.2.3. MDH1 and MDH2 Enzyme Activity Assay

The activities of MDH1 and MDH2 were confirmed using an oxaloacetate-dependent NADH oxidation assay. Experiments were conducted using 100 mM of potassium phosphate buffer (pH 7.4) and human recombinant MDH1 (BioVision) and MDH2 (BioVision), and the results were obtained by measuring the absorbance at 340 nm. Enzyme activity was measured using a plate reader at a wavelength of 340 nm.

#### 4.2.4. Western Blot Analysis

The cells were lysed with RIPA buffer (Millipore, Billerica, MA, USA) containing a protease inhibitor cocktail (Roche, Basel, Switzerland) for 15 min at 4 °C and centrifuged at 12,000 rpm for 10 min. The lysates were quantified using a protein assay kit (Bio-Rad, Hercules, CA, USA). The lysates were subjected to an SDS-PAGE analysis and transferred to PVDF membranes (Millipore, Billerica, MA, USA). The proteins were identified using the appropriate antibodies: HIF-1α (BD Transduction Laboratories, San Diego, CA, USA), pyruvate dehydrogenase kinase 1 (PDK1) (Cell Signaling, Danvers, MA, USA), GLUT1 and β-action (Santa Cruz Biotechnology, Danvers, MA, USA), and CD73 (Proteintech, Rosemont, IL, USA).

#### 4.2.5. APT Contents

A549 cells (1 × 10^4^ cells/96-well plate) were incubated for 6 h with or without drugs. After removing the supernatant, an ATP assay was performed using ATPlite (PerkinElmer, Waltham, MA, USA) according to the manufacturer’s instructions. ATP content was measured using a luminometer (Synergy HTX multimode reader, Winoski, VT, USA).

#### 4.2.6. ADP and ATP Measurement

The ADP/ATP ratio assay kit (Sigma-Aldrich, USA) was used for ADP/ATP determination according to the manufacturer’s instructions. Samples for analysis were prepared using the ADP/ATP ratio assay kit. A549 cells were cultured directly in an assay microplate. The culture medium was removed, and then 90 µL of ATP reagent was added to each well and incubated for 1 min at room temperature. Luminescence was measured on a luminometer for ATP (RLU_A_), and the plate was incubated for an additional 10 min. Next, the luminescence was read on a luminometer for ATP (RLU_B_). Five microliters of ADP reagent were added to each well. After 1 min, the luminescence was read (RLU_C_).

#### 4.2.7. Enzyme-Linked Immunosorbent Assay (ELISA)

The prepared samples were incubated with the same amount of antibody cocktail for 1 h at room temperature and then washed three times with PT buffer. After washing, the plates were developed by adding 100 µL of TMB development solution, and the reaction was stopped by adding 100 µL of stop solution. The amount of CD73 was quantitatively measured according to the instructions on the Human CD73 Simplestep ELISA kit (Abcam, Cambridge, UK).

### 4.3. Docking Methodology

#### 4.3.1. Ligand Setting

The chemical structure preparation procedure was performed using ChemDraw software. Ligands were prepared by sketching the 2D structure of each ligand, and 3D structures of all ligands were produced using a molecular mechanical (MM) geometry optimization approach. The Epik function in the LigPrep module of Maestro was used in accordance with the Hammett and Taft methods to return the pKa values and 3D structure files for multiple tautomers and ionization states that are likely to exist under the specified conditions. This was accomplished by setting the physiological pH (pH 7.4) at the protonation state step and the geometry by repeating the optimization step. Accordingly, Epik can be automatically used by LigPrep to enumerate the tautomers and protonation states.

#### 4.3.2. Receptor Preparation

The crystal structures of MDH1 (PDB:5MDH, resolution 2.4 Å) and MDH2 (PDB:4WLO, resolution 2.5 Å) were retrieved from the Protein Data Bank server [35,36]. The protein structures were subjected to the protein preparation procedure using Schrodinger software 2020, which included: (i) removing all water molecules from the crystal structure (implicit solvation was used); (ii) assignment of bond orders; (iii) adding hydrogen atoms; (iv) setting the physiological pH (pH 7) via the protonation states of the corresponding amino acid residues using PROPKA software 20 in the Schrodinger molecular modeling package; and (v) restraining the minimization of added hydrogen atoms. The active site used in the docking study was constructed from chain A of the crystal structure.

## 5. Conclusions

In this study, various structural modifications were employed for the hit compound **LW1497** to design new scaffolds. Accordingly, a series of novel (2,4,4-trimethylpentan-2-yl)benzene derivatives were synthesized and screened for their inhibitory effect against MDH1 and MDH2 enzymes. Optimization of the SAR study resulted in the identification of a series of novel dual or selective MDH1 and MDH2 inhibitors. Compounds **5d** and **35a** showed significant inhibitory activity against both MDH1 and MDH2, whereas compounds **5f**, **14**, **44a**, **50**, **56**, **57a**, and **57b** showed similar activity against both enzymes, compared with the positive control **LW1497**. Compounds **39b**, **39c**, **39d**, and **57d** displayed selectivity towards MDH1, whereas **5b** and **5c** showed selective MDH2 activity. In addition, the growth inhibition results of all tested compounds revealed that compound **50**, which contains a piperidine linker, showed a two-fold improved potency in A549 (IC_50_ = 3.94 ± 0.04 μM) and H460 (IC_50_ = 3.67 ± 0.06 μM) cell lines compared with the hit compound **LW1497**. The most potent compound **50** decreased ATP production by inhibiting the MAS shuttle. As expected, compound **50** suppressed the expression of HIF-1α target genes as well as hypoxia-induced HIF-1α accumulation. Therefore, compound **50,** now **LW2393**, may serve as a promising lead for the development of novel MDH1/2 inhibitors for the treatment of lung cancer.

## Figures and Tables

**Table 1:** In vitro MDH inhibitory activity and cell viability of amide derivatives **5a**−**f**.

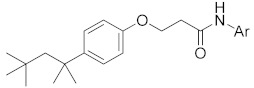						
Compd.	Ar	MDH1 IC_50_ (µM) ^a^	MDH2 IC_50_ (µM) ^a^		Cell Viability IC_50_ (µM) ^a^	
					A549	H460
**5a**	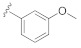	6.18 ± 0.74		1.5 ± 0.01	11.1 ± 0.77	8.83 ± 0.34
**5b**	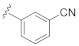	6.96 ± 0.12		2 ± 0.05	5.25 ± 0.21	5.84 ± 0.74
**5c**	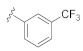	8.35 ± 2.86		1.04 ± 0	6.47 ± 0.06	5.68 ± 0.23
**5d**	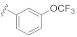	0.94 ± 0.05		1.24 ± 0.13	6.42 ± 0.06	6.81 ± 0.04
**5e**	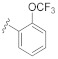	3.14 ± 0.14		3.09 ± 0.2	>10	>10
**5f**	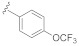	3.45 ± 0.07		2.12 ± 0.21	5.97 ± 0.71	3.77 ± 0.02
**LW1497**		2.24 ± 0.11		1.59 ± 0.04	7.98 ± 0.53	6.14 ± 0.29

^a^ Values are the means of three experiments.

**Table 2:** In vitro MDH inhibitory activity and cell viability of trimethylpentane-modified derivatives **14**, **21**, **22**, **30**, and **35a**–**b**.

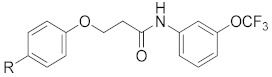					
Compd.	R	MDH1 IC_50_ (µM) ^a^	MDH2 IC_50_ (µM) ^a^	Cell Viability IC_50_ (µM) ^a^	
				A549	H460
**14**	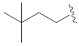	3.05 ± 0.29	1.53 ± 0.08	14.44 ± 0.1	12.77 ± 1.36
**21**	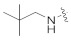	>5	>5	>10	>10
**22**	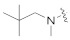	>5	3.13 ± 0.29	>10	>10
**30**	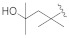	>5	>5	8.28 ± 0.76	ND
**35a**	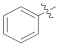	0.78 ± 0.08	1.38 ± 0.59	>10	>10
**35b**	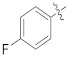	>5	>5	>10	>10
**LW1497**		2.24 ± 0.11	1.59 ± 0.04	7.98 ± 0.53	6.14 ± 0.29

^a^ Values are the means of three experiments.

**Table 3:** In vitro MDH inhibitory activity and cell viability of naphthalene derivatives **39a**–**d**.

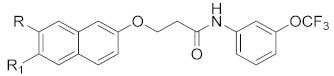						
Compd.	R	R_1_	MDH1 IC_50_ (µM) ^a^	MDH2 IC_50_ (µM) ^a^	Cell Viability IC_50_ (µM) ^a^	
					A549	H460
**39a**	H	H	>5	>5	>20	>20
**39b**	Br	H	1.61 ± 0.25	>5	14.19 ± 0.2	14.86 ± 0.07
**39c**	H	Br	1.61 ± 0.21	>5	17.66 ± 0.18	14.75 ± 0.18
**39d**	H	F	2.25 ± 0.77	18.29 ± 1.8	>20	ND
**LW1497**			2.24 ± 0.11	1.59 ± 0.04	7.98 ± 0.53	6.14 ± 0.29

^a^ Values are the means of three experiments.

**Table 4:** In vitro MDH inhibitory activity and cell viability of amide and sulfonamide derivatives **44a**–**b**.

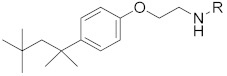					
Compd.	R	MDH1 IC_50_ (µM) ^a^	MDH2 IC_50_ (µM) ^a^	Cell Viability IC_50_ (µM) ^a^	
				A549	H460
**44a**	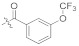	3.15 ± 0.11	2.22 ± 0.28	13.15 ± 1.3	12.41 ± 0.21
**44b**	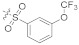	2.85 ± 0.45	>5	7.32 ± 0.02	7.61 ± 0.24
**LW1497**		2.24 ± 0.11	1.59 ± 0.04	7.98 ± 0.53	6.14 ± 0.29

^a^ Values are the means of three experiments.

**Table 5:** In vitro MDH inhibitory activity and cell viability of linker-modified derivatives **50** and **51**.

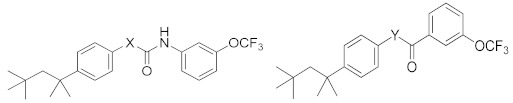					
Compd.	X	MDH1 IC_50_ (µM) ^a^	MDH2 IC_50_ (µM) ^a^	Cell Viability IC_50_ (µM) ^a^	
				A549	H460
**50**	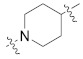	3.33 ± 0.18	2.24 ± 0.09	3.94 ± 0.04	3.67 ± 0.06
	**Y**				
**51**	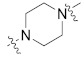	5 ± 2.49	>5	20>	18.4 ± 1.55
**LW1497**		2.24 ± 0.11	1.59 ± 0.04	7.98 ± 0.53	6.14 ± 0.29

^a^ Values are the means of three experiments.

**Table 6:** In vitro MDH inhibitory activity and cell viability of linker-modified derivatives **56**, **57a**–**d**.

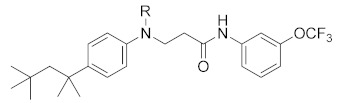					
Compd.	R	MDH1 IC_50_ (µM) ^a^	MDH2 IC_50_ (µM) ^a^	Cell Viability IC_50_ (µM) ^a^	
				A549	H460
**56**	H	1.79 ± 0.04	3.18 ± 0.18	6.06 ± 0.01	5.3 ± 0.01
**57a**	Me	2.42 ± 0.04	1.54 ± 0.07	4.41 ± 0.06	4.34 ± 0.03
**57b**	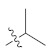	1.61 ± 0.02	1.09 ± 0.06	6.47 ± 0.06	6.06 ± 0.06
**57c**	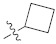	2.16 ± 0.01	1.18 ± 0.01	7.24 ± 0.11	7.11 ± 0.03
**57d**	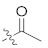	1.41 ± 0.47	>5	>20	>20
**LW1497**		2.24 ± 0.11	1.59 ± 0.04	7.98 ± 0.53	6.14 ± 0.29

^a^ Values are the means of three experiments.

## Data Availability

Data is contained within the article and Supplementary Materials.

## Supplementary Materials

The following supporting information can be downloaded at: https://www.mdpi.com/article/10.3390/ph16050683/s1, Figures S1–S4: ATP contents, production and CD73 expression; Figures S5–S98: 1H NMR, ^13^C NMR and ^19^F NMR spectra of the final compounds, and HPLC and HR-ESIMS data.
