# Supplementary material for: Design, Synthesis and Biological Evaluation of Novel MDH Inhibitors Targeting Tumor Microenvironment
Source: Pharmaceuticals (Basel). 2023 May 2;16(5):683. doi: 10.3390/ph16050683 (PMC10263210; doi:10.3390/ph16050683)

Design, Synthesis and Biological Evaluation of Novel MDH inhibitors Targeting Tumor Microenvironment

Sreenivasulu Godesi [1,†], Jeong-Ran Han [2,†], Jang-Keun Kim [2], Dong-Ik Kwak [1], Joohan Lee [1], Hossam Nada [1], Minkyoung Kim [1], Hyun-A Yang [2], Joo-Young Im [2], Hyun Seung Ban [3], Chang Hoon Lee [1], Yongseok Choi [4], Misun Won [2,*] and Kyeong Lee [1,*]

[1] BK21 FOUR Team and Integrated Research Institute for Drug Development, College of Pharmacy, Dongguk University-Seoul, Goyang 10326, Republic of Korea

[2] Personalized Genomic Medicine Research Center, Korea Research Institute of Bioscience and Biotechnology, Daejeon 34141, Republic of Korea

[3] Biotherapeutics Translational Research Center, KRIBB, Daejeon 34141, Republic of Korea

[4] Department of Biotechnology, Korea University, Seoul 02841, Republic of Korea

[†]These authors contributed equally to this work

[*] Correspondence: kaylee@dongguk.edu (K.L.); misun@kribb.re.kr (M.W.)

Supporting Information

Table of Contents

ATP contents, production, CD73 expression	S1−S4
^{1}H NMR, ^{13}C NMR and ^{19}F NMR spectra of compounds	S5−S76
HPLC of compounds	S77−S86
HR-ESIMS of compounds	S87−S98

Supplementary Figure S1. ATP contents of compound **57a**

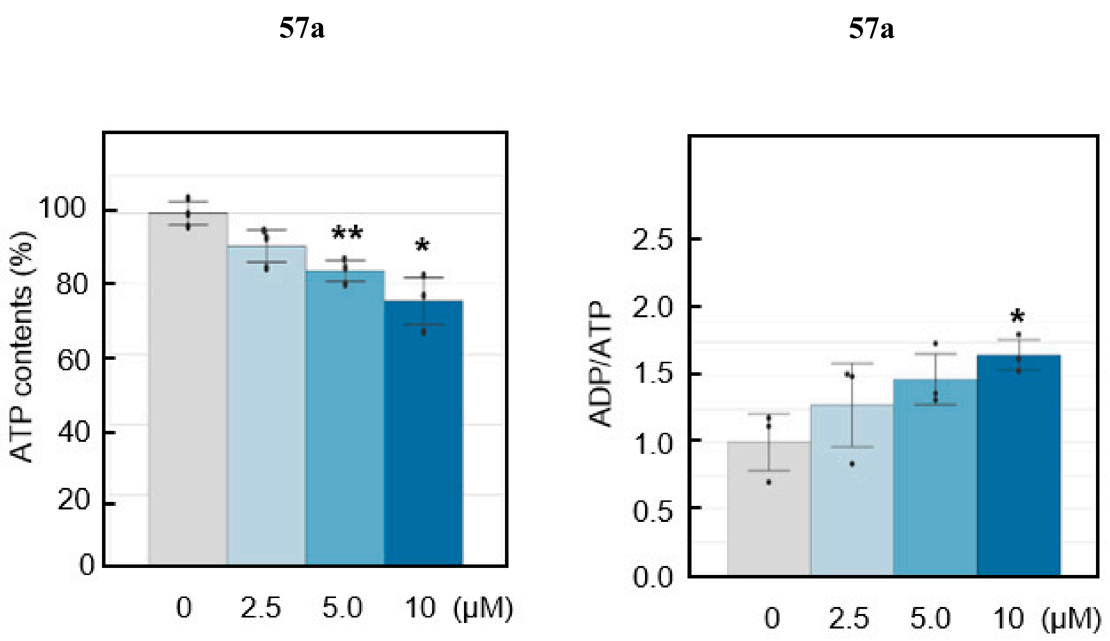

Supplementary Figure S2. ATP production of compound **57a**

mito/gly ATP	total ATP	mito ATP (%)	mito ATP reduction	glyco ATP reduction
560/561.7	1121.7(100%)	49.92%	100	100
459.5/487.9	947.4(84.46%)	48.50%	18.03%	13.17
379.6/446.4	826 (73.63%)	45.96%	27.75%	20.52

Supplementary Figure S3. Measurement of the intracellular ATP production rates in A549 cells in a dose-dependent manner by compound **57a**

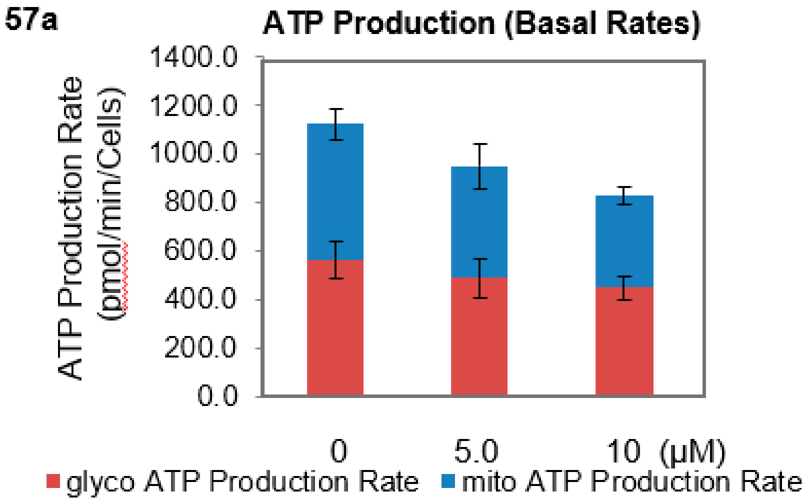

Supplementary Figure S4. Decrease in expression of HIF-1α and its target genes by compound **57a**

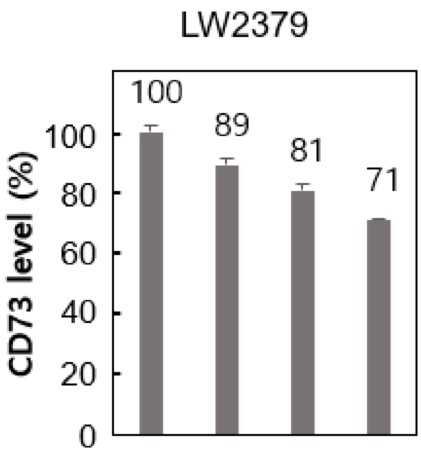

Figure S5. ^{1}H NMR (400 MHz, $CDCl_3$) spectrum of compound **5a**

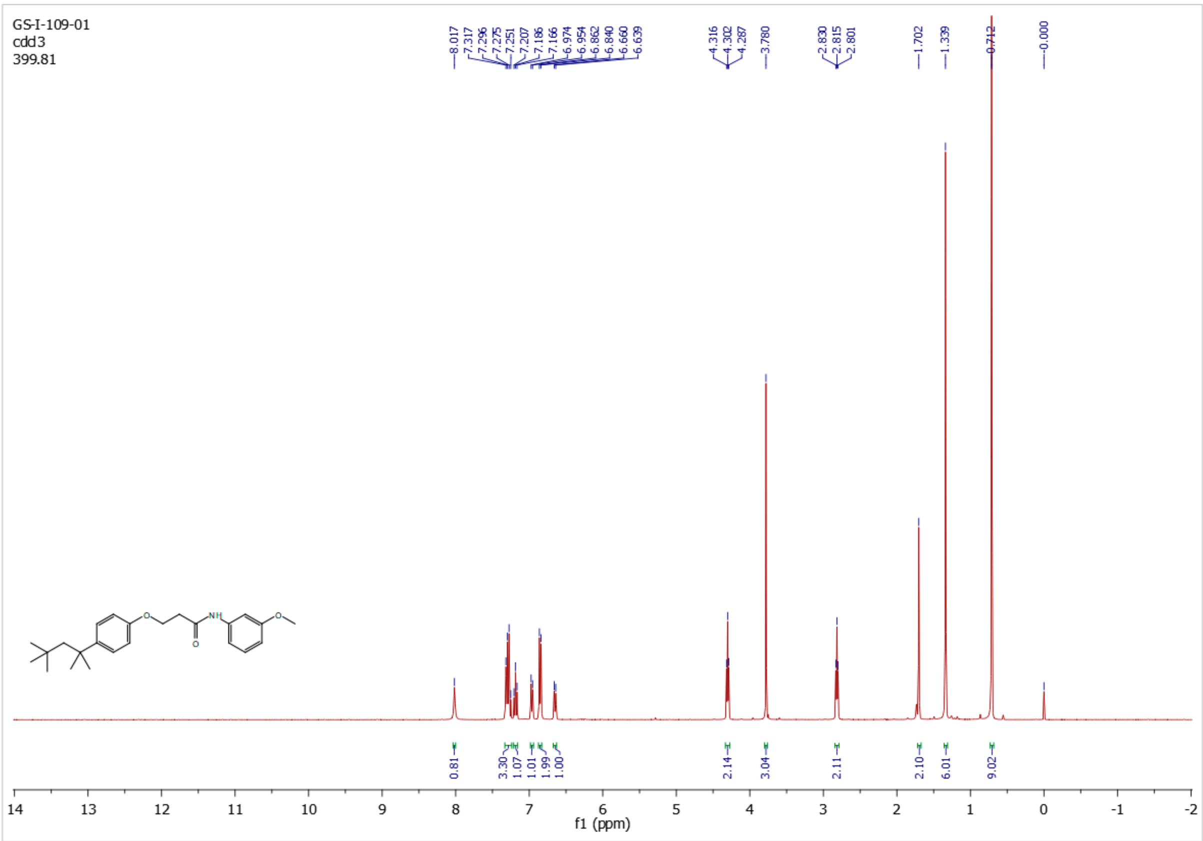

Figure S6. ^{13}C NMR (100 MHz, $CDCl_3$) spectrum of compound **5a**

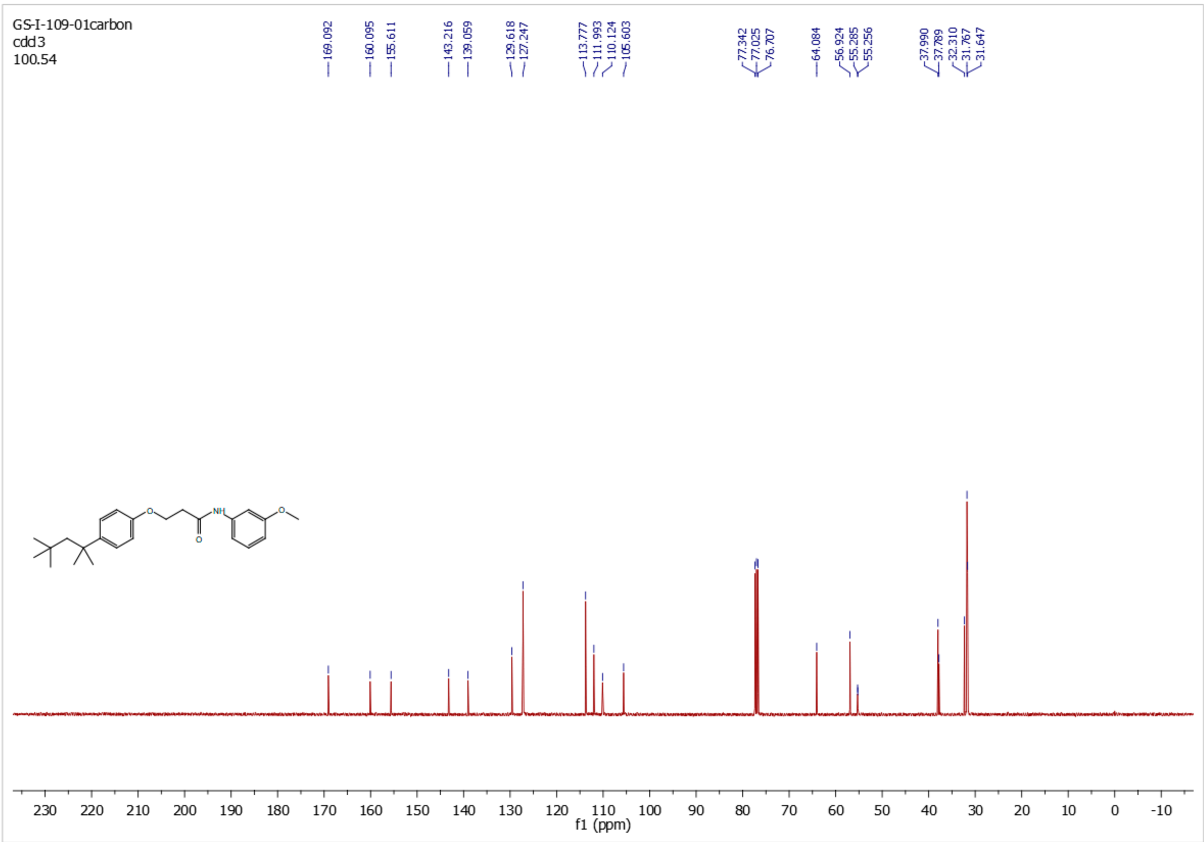

Figure S7. ^{1}H NMR (400 MHz, $CDCl_3$) spectrum of compound **5b**

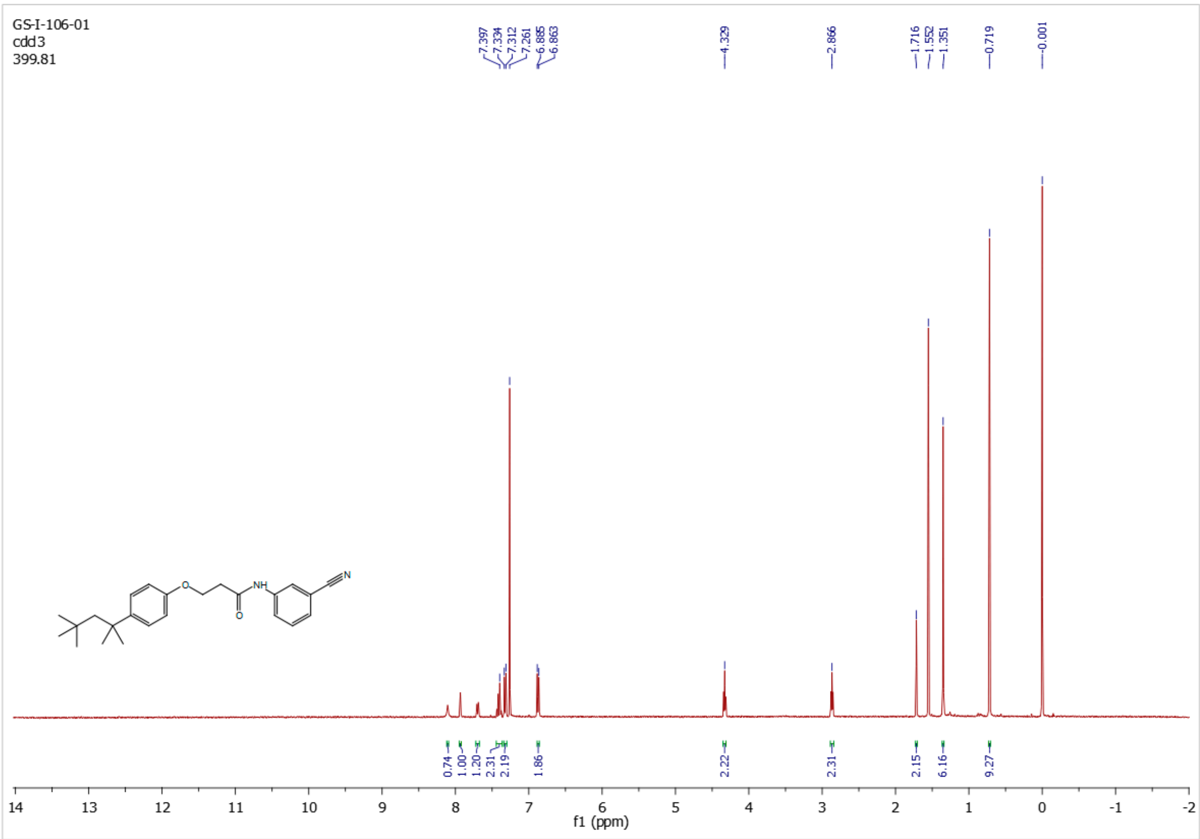

Figure S8. ^{13}C NMR (100 MHz, $CDCl_3$) spectrum of compound **5b**

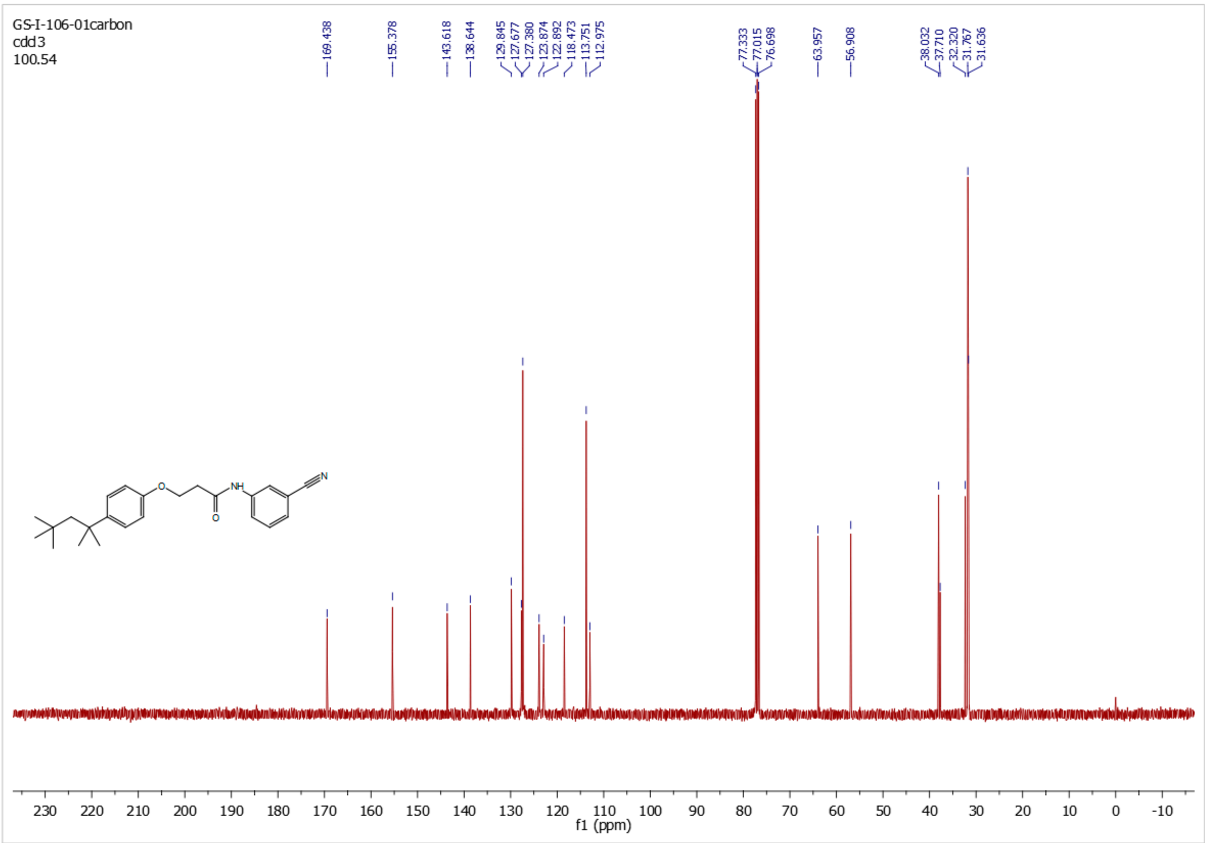

Figure S9. ^{1}H NMR (400 MHz, $CDCl_3$) spectrum of compound **5c**

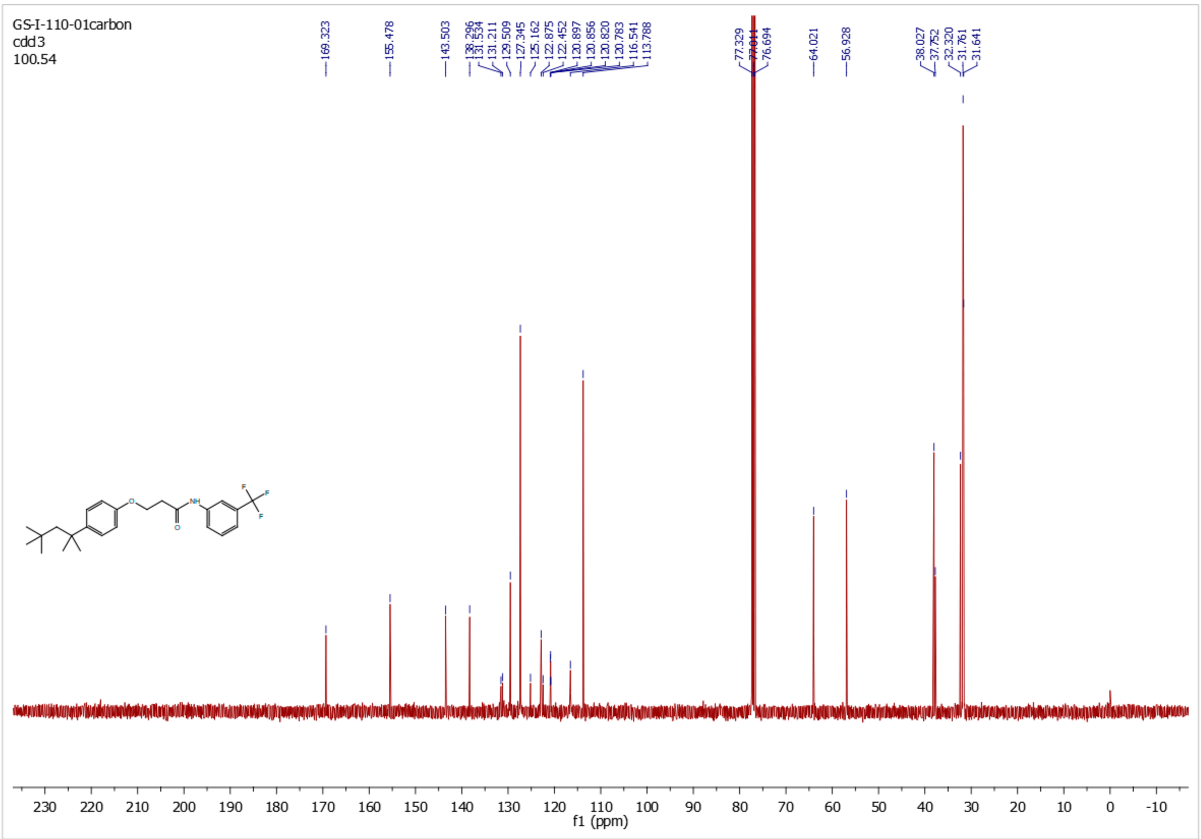

Figure S10. ^{13}C NMR (100 MHz, $CDCl_3$) spectrum of compound **5c**

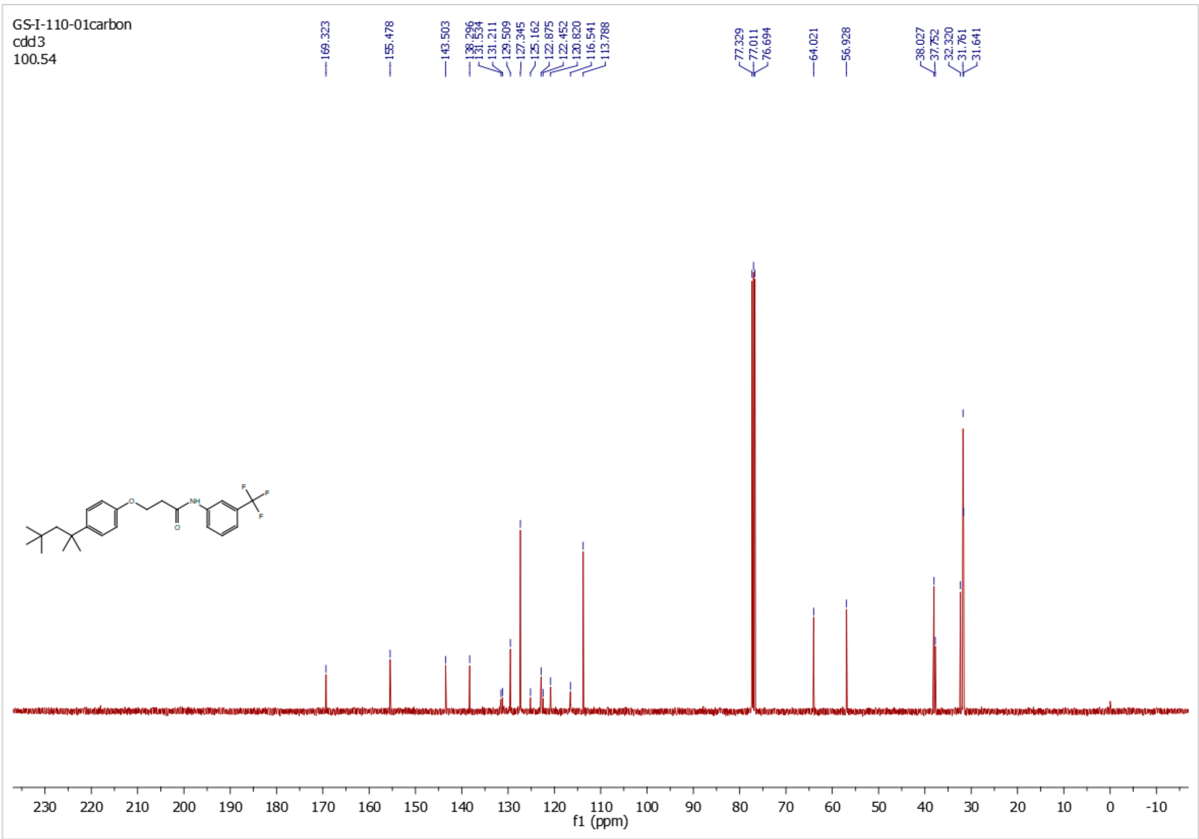

Figure S11. ^{19}F NMR (376 MHz, $CDCl_3$) spectrum of compound **5c**

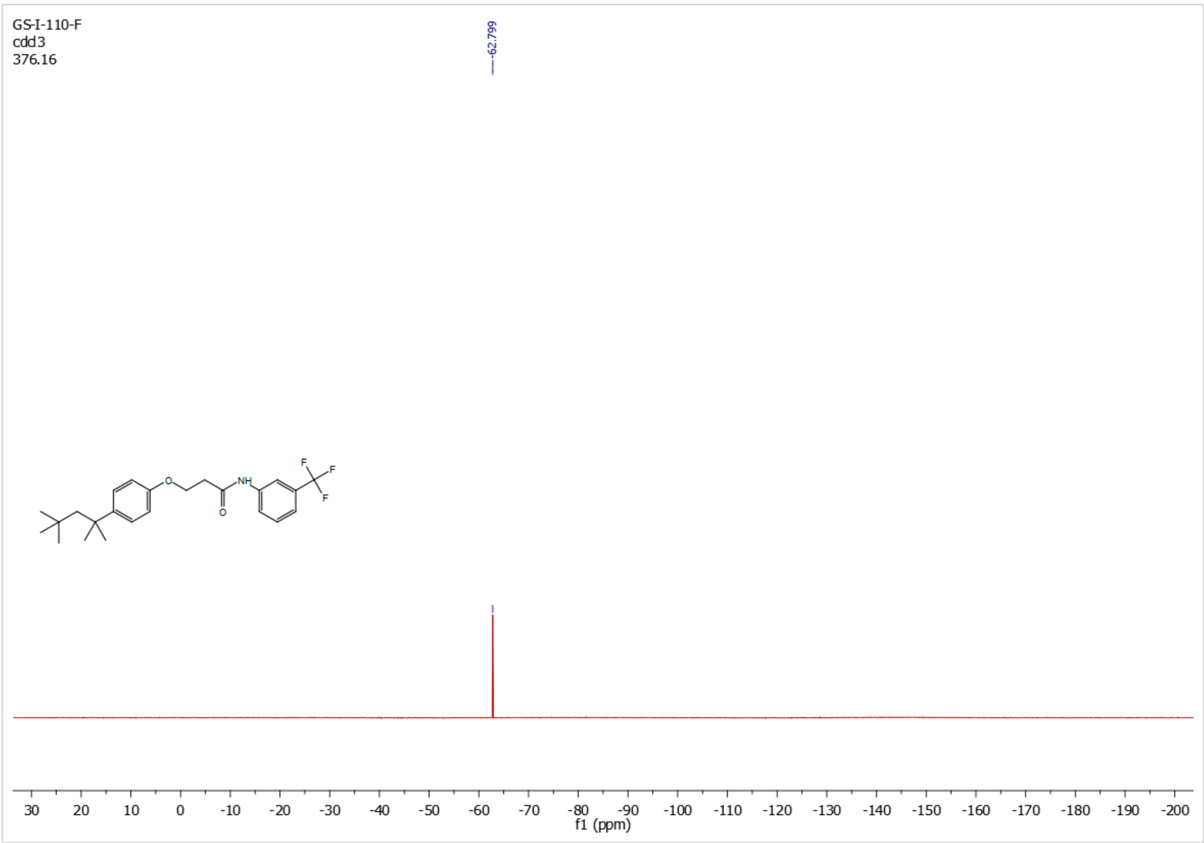

Figure S12. ^{1}H NMR (400 MHz, $CDCl_3$) spectrum of compound **5d**

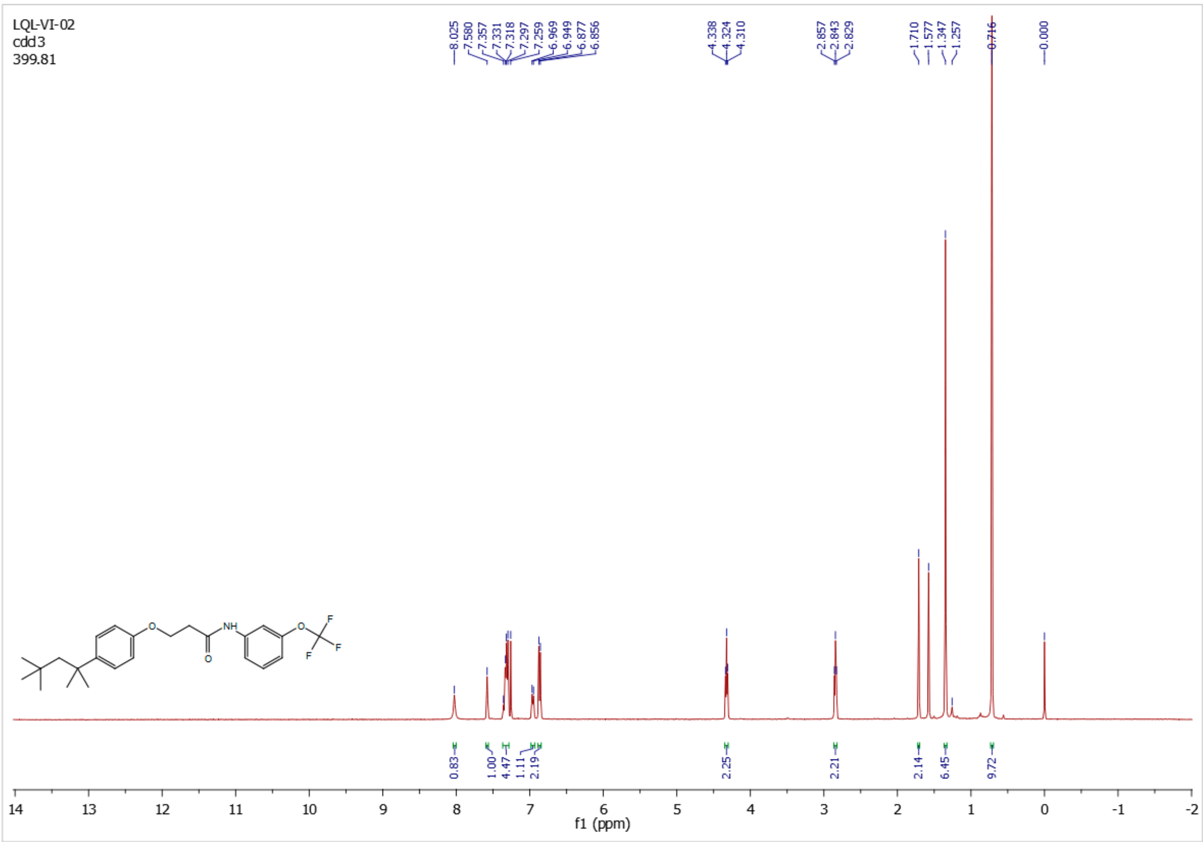

Figure S13. ^{13}C NMR (100 MHz, $CDCl_3$) spectrum of compound **5d**

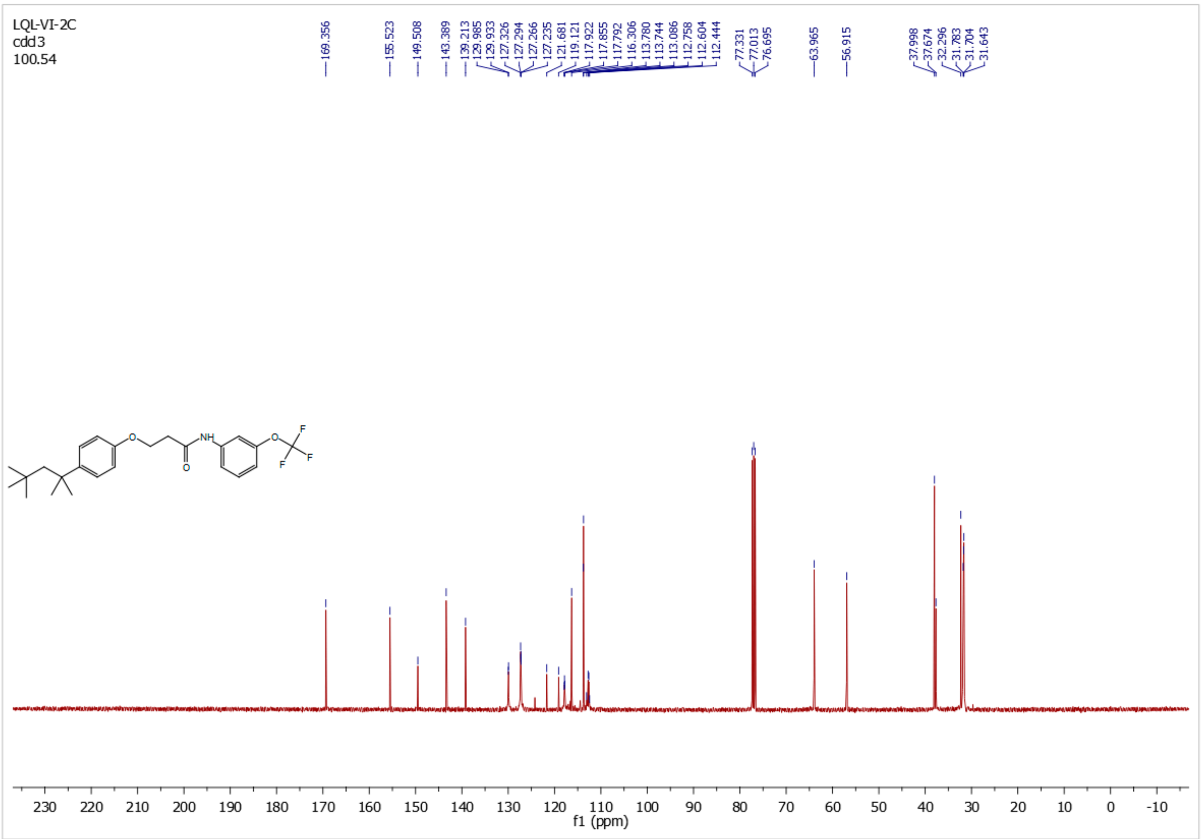

Figure S14. ^{19}F NMR (376 MHz, $CDCl_3$) spectrum of compound **5d**

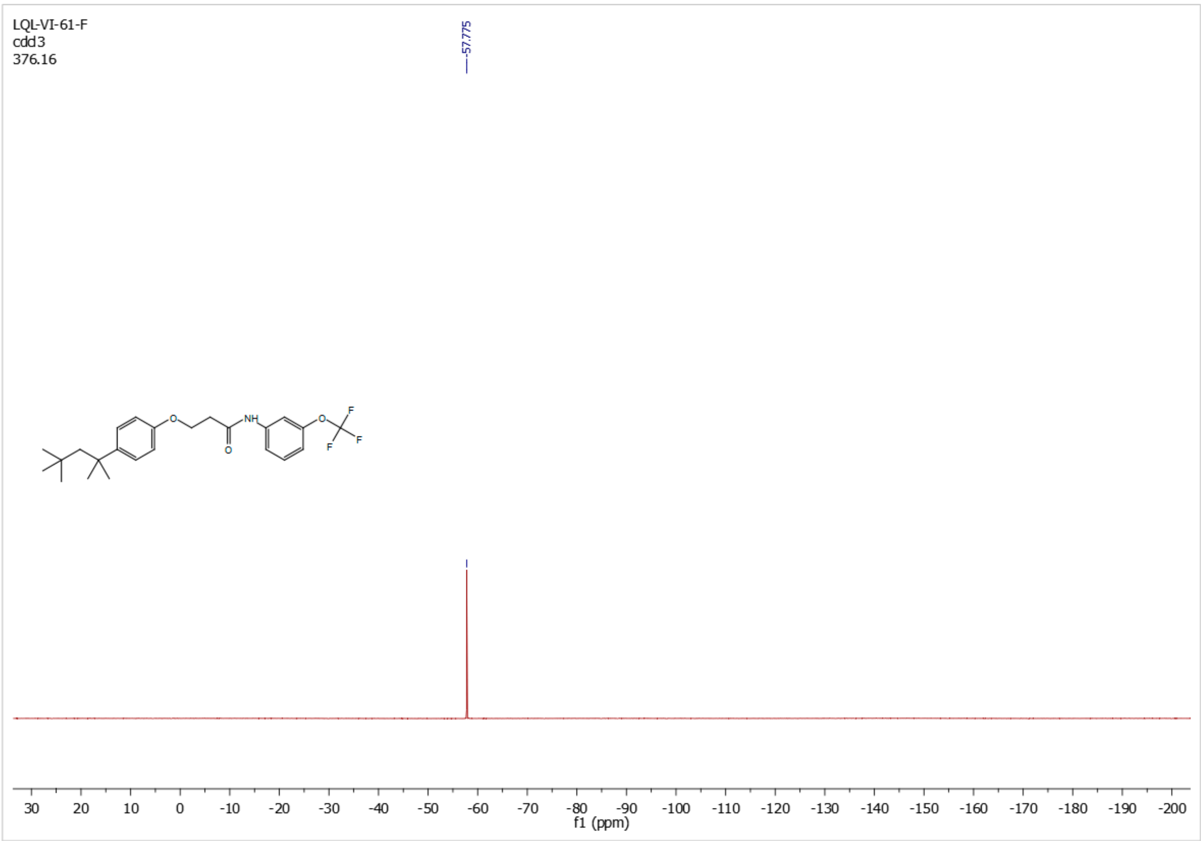

Figure S15. ^{1}H NMR (400 MHz, $CDCl_3$) spectrum of compound **5e**

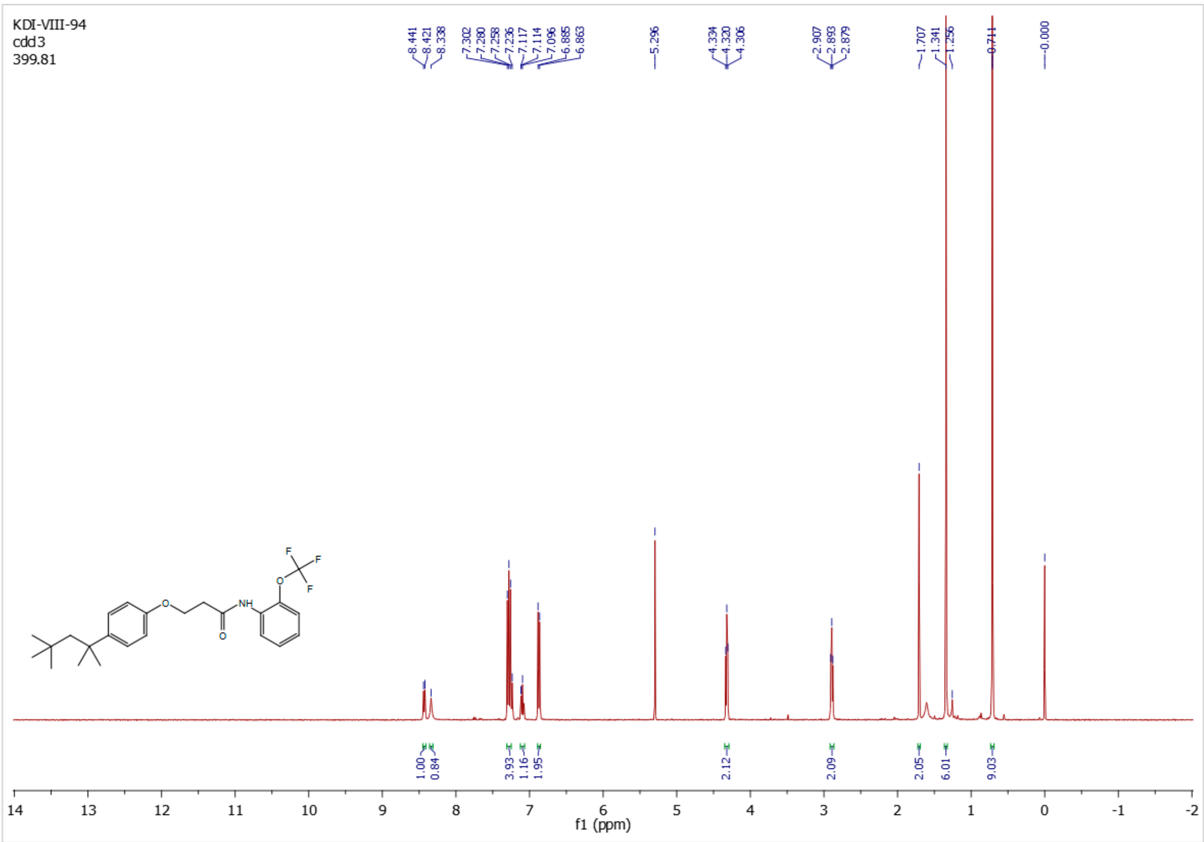

Figure S16. ^{13}C NMR (100 MHz, $CDCl_3$) spectrum of compound **5e**

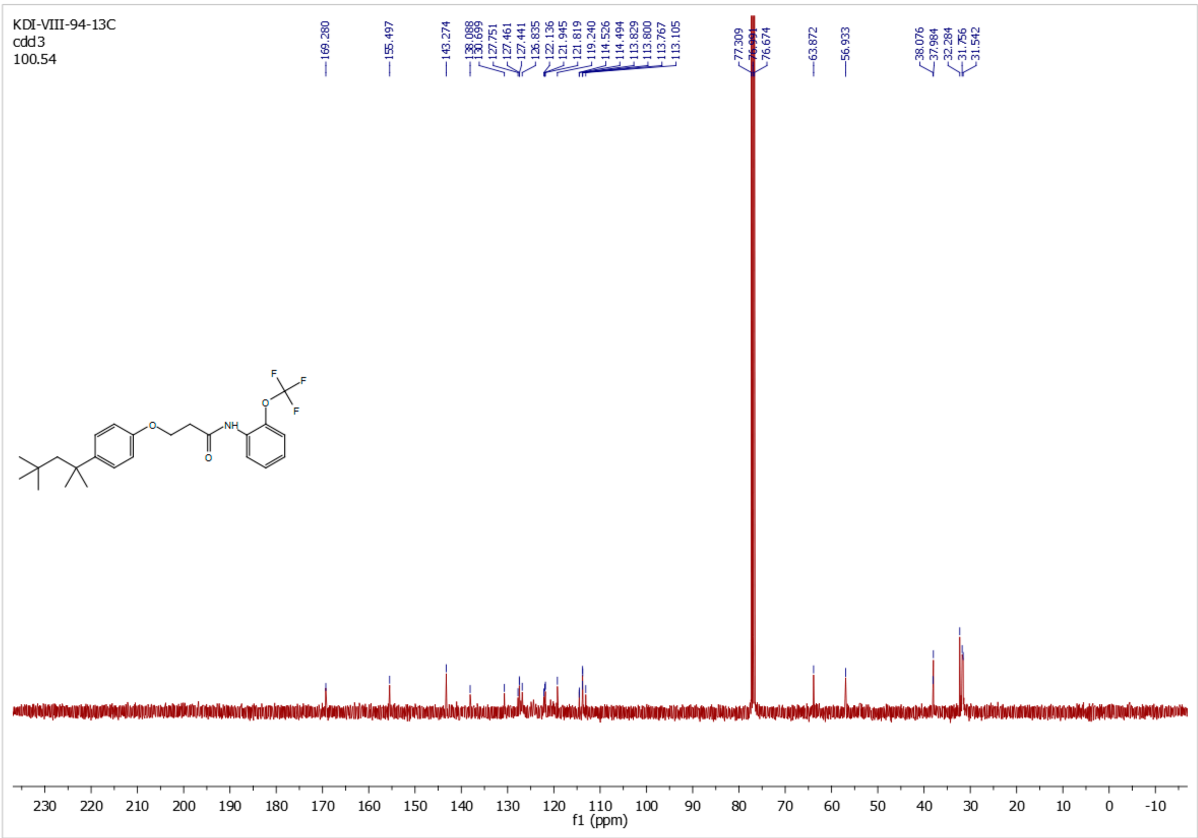

Figure S17. ^{19}F NMR (376 MHz, $CDCl_3$) spectrum of compound **5e**

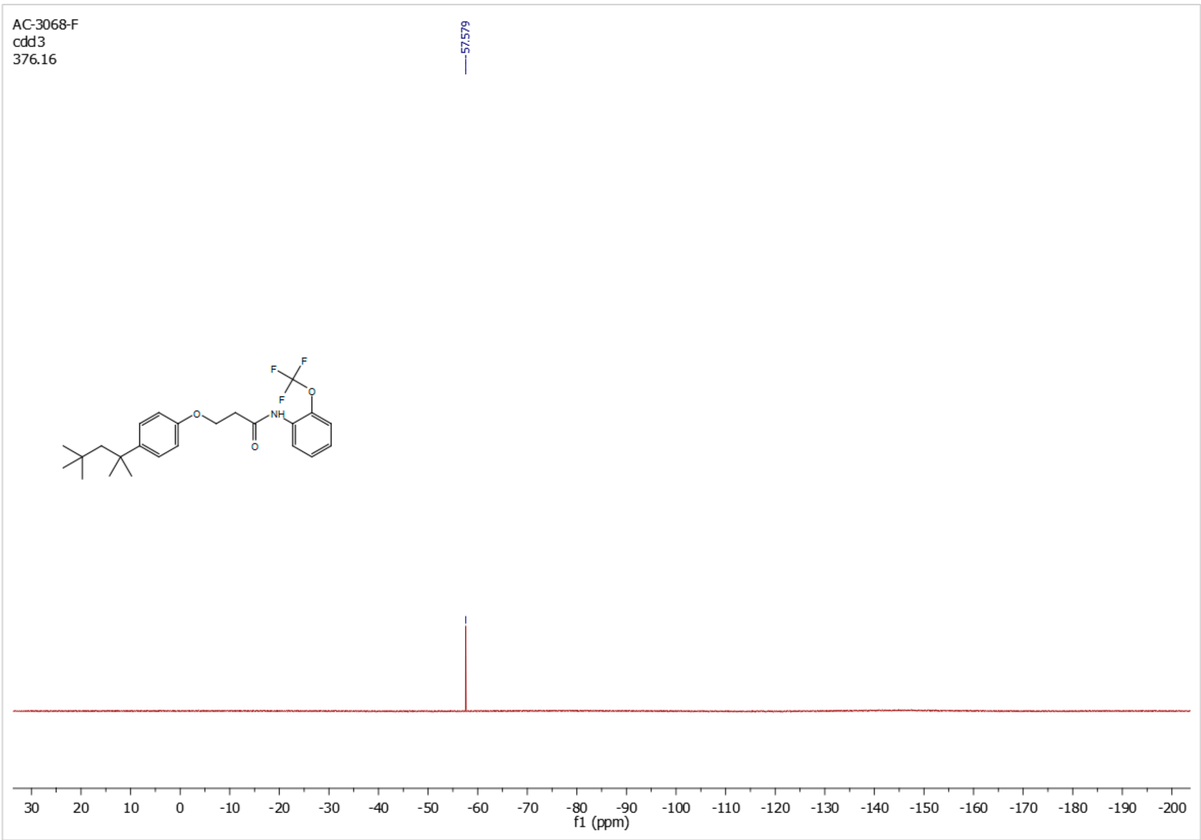

Figure S18. ^{1}H NMR (400 MHz, $CDCl_3$) spectrum of compound **5f**

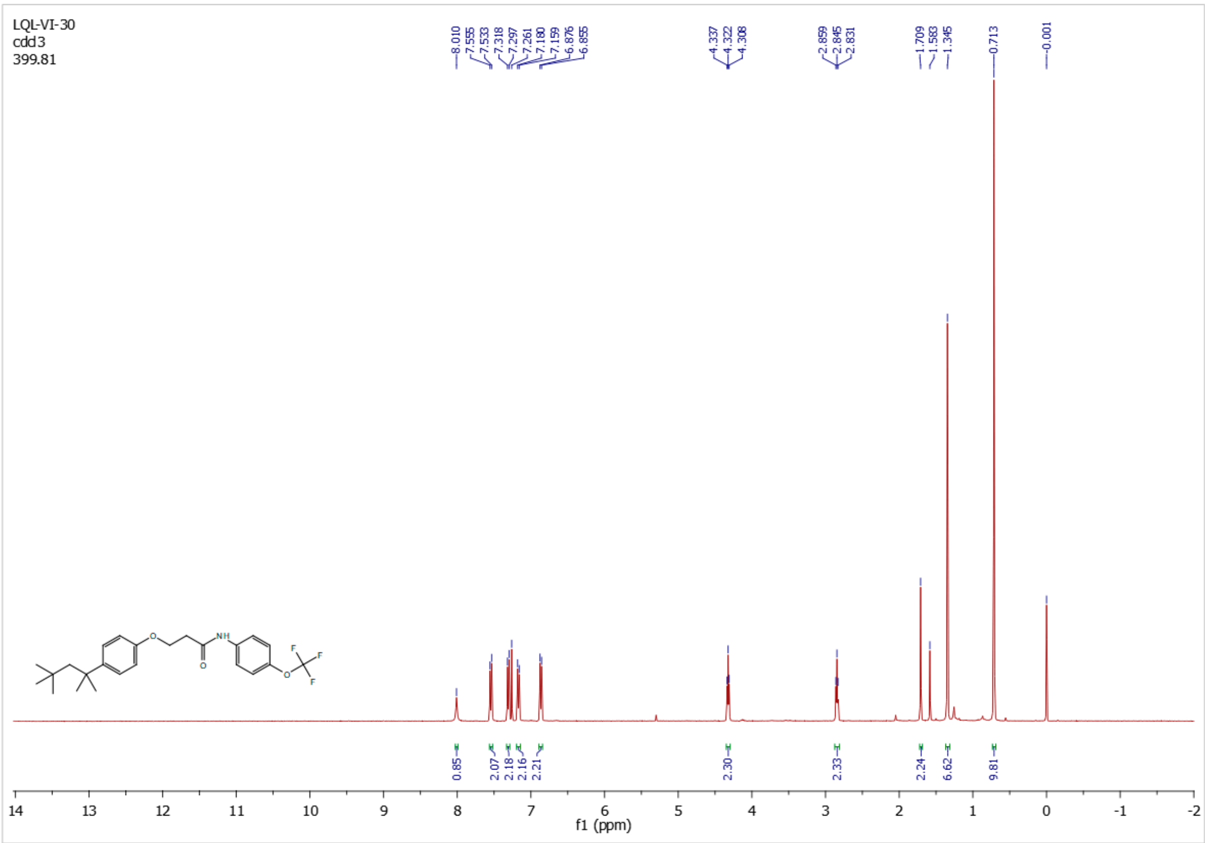

Figure S19. ^{19}F NMR (376 MHz, $CDCl_3$) spectrum of compound **5f**

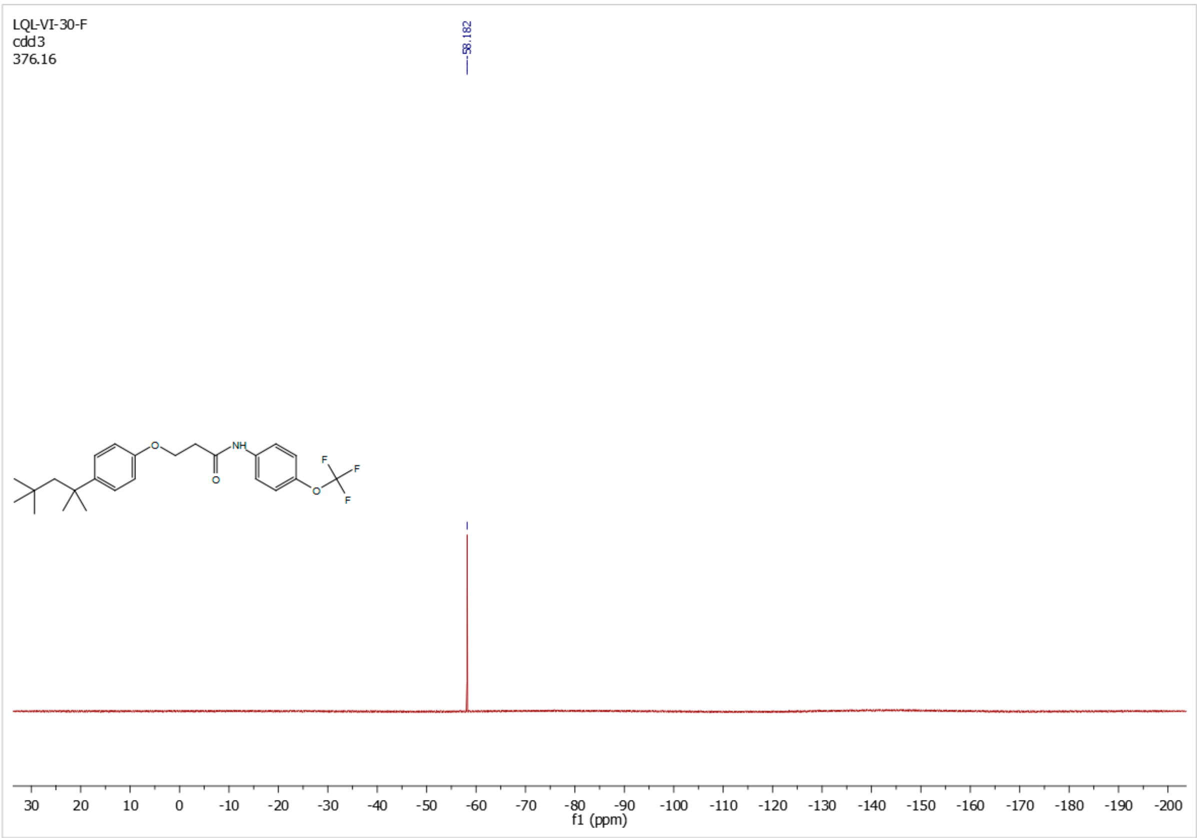

Figure S20. ^{1}H NMR (400 MHz, $CDCl_3$) spectrum of compound **14**

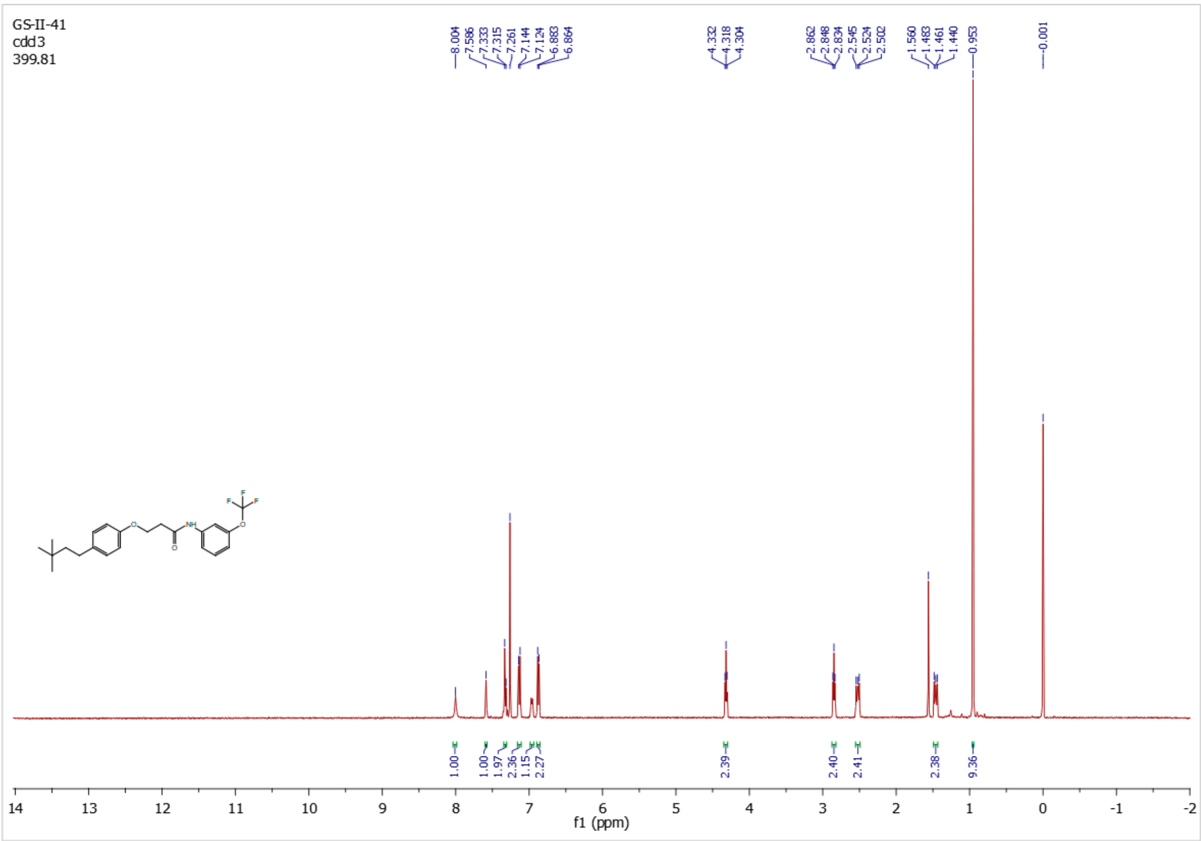

Figure S21. ^{13}C NMR (100 MHz, CD_3OD) spectrum of compound **14**

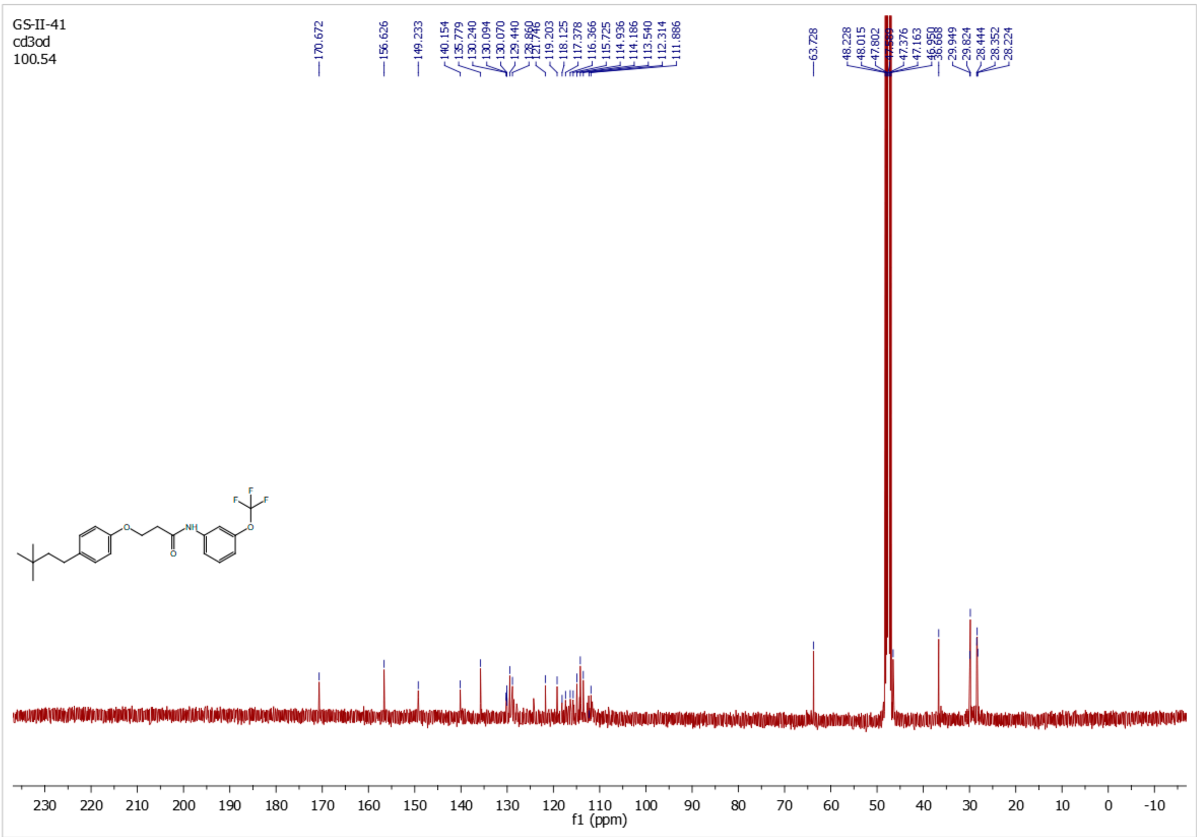

Figure S22. ^{19}F NMR (376 MHz, $CDCl_3$) spectrum of compound **14**

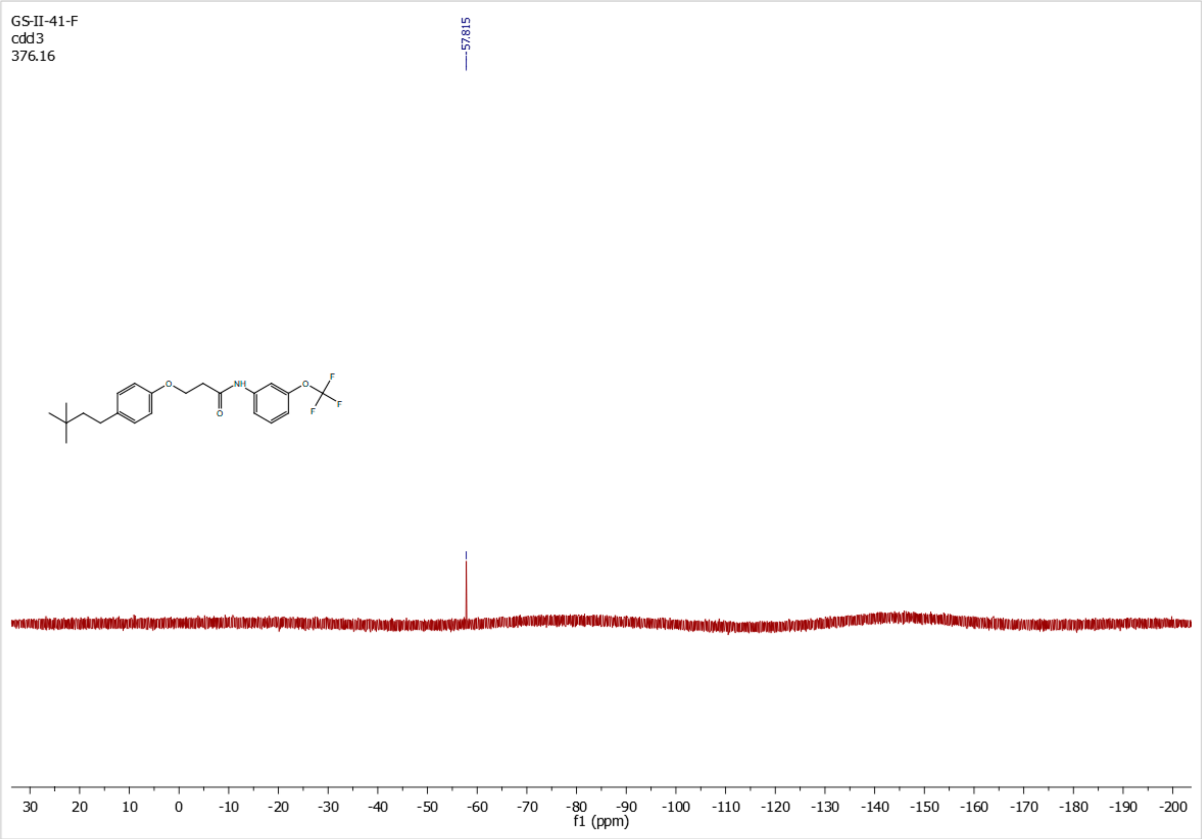

Figure S23. ^{1}H NMR (400 MHz, $CDCl_3$) spectrum of compound **21**

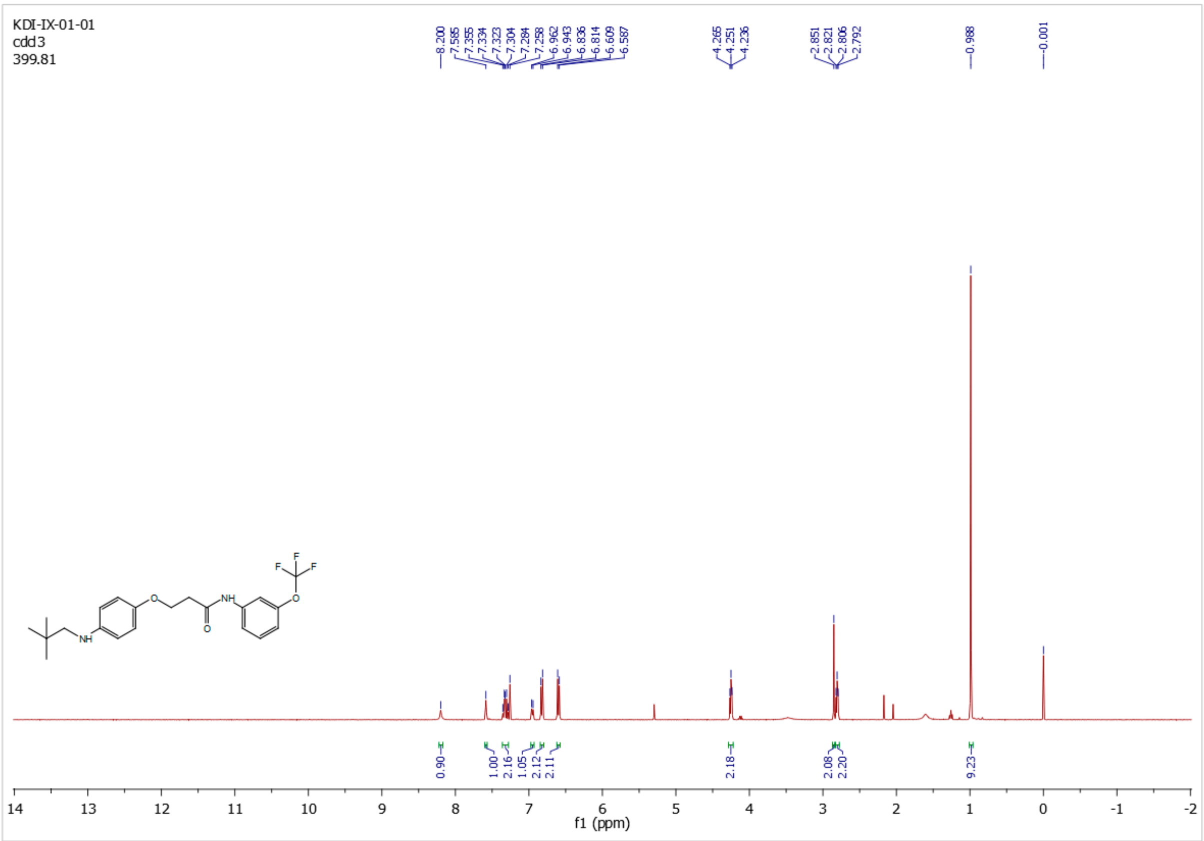

Figure S24. ^{13}C NMR (100 MHz, $CDCl_3$) spectrum of compound **21**

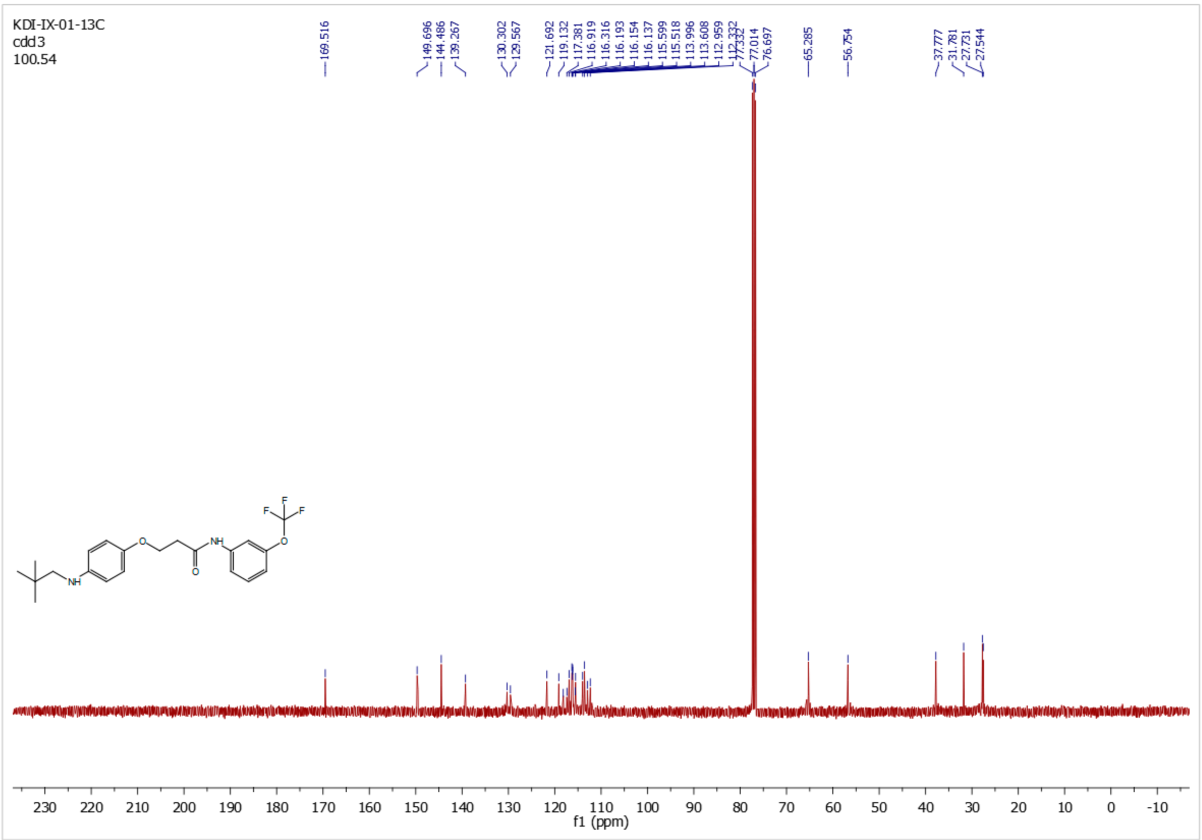

Figure S25. ^{19}F NMR (376 MHz, $CDCl_3$) spectrum of compound **21**

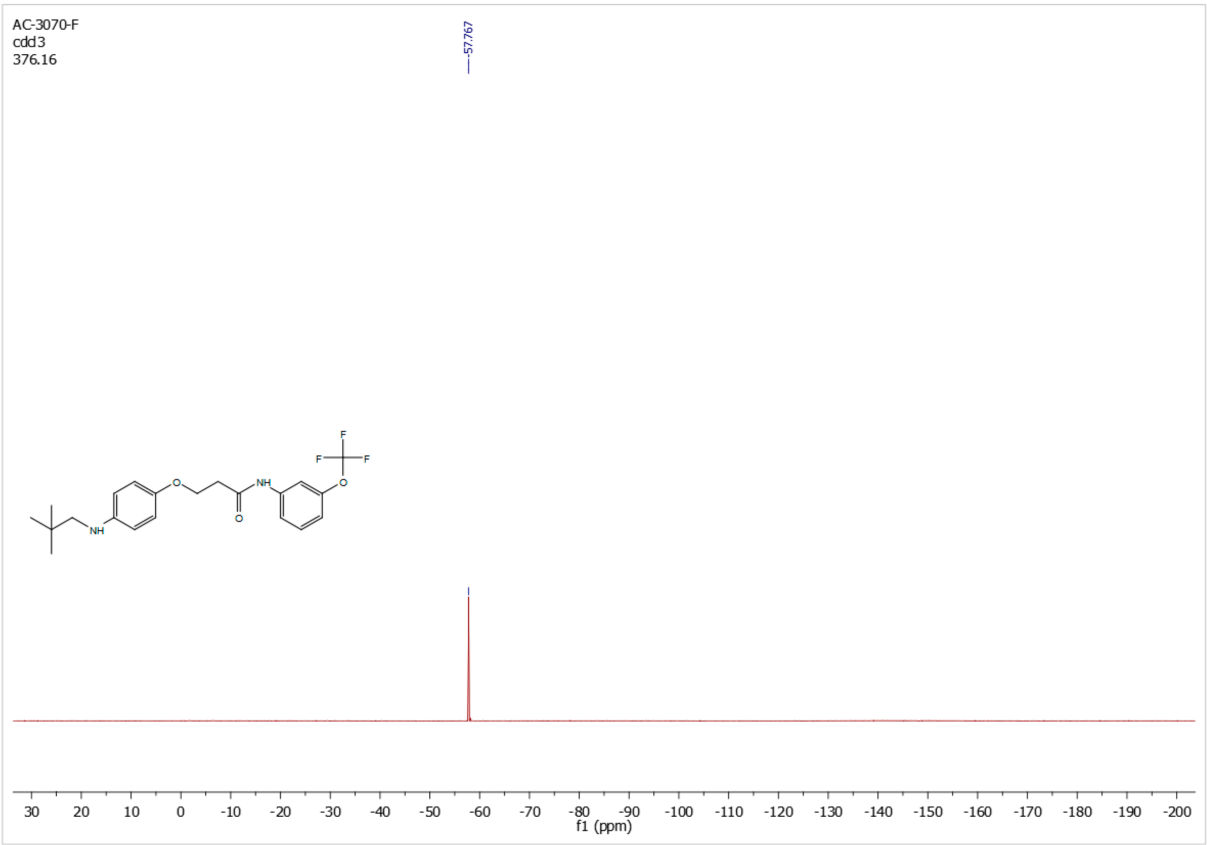

Figure S26. ^{1}H NMR (400 MHz, $CDCl_3$) spectrum of compound **22**

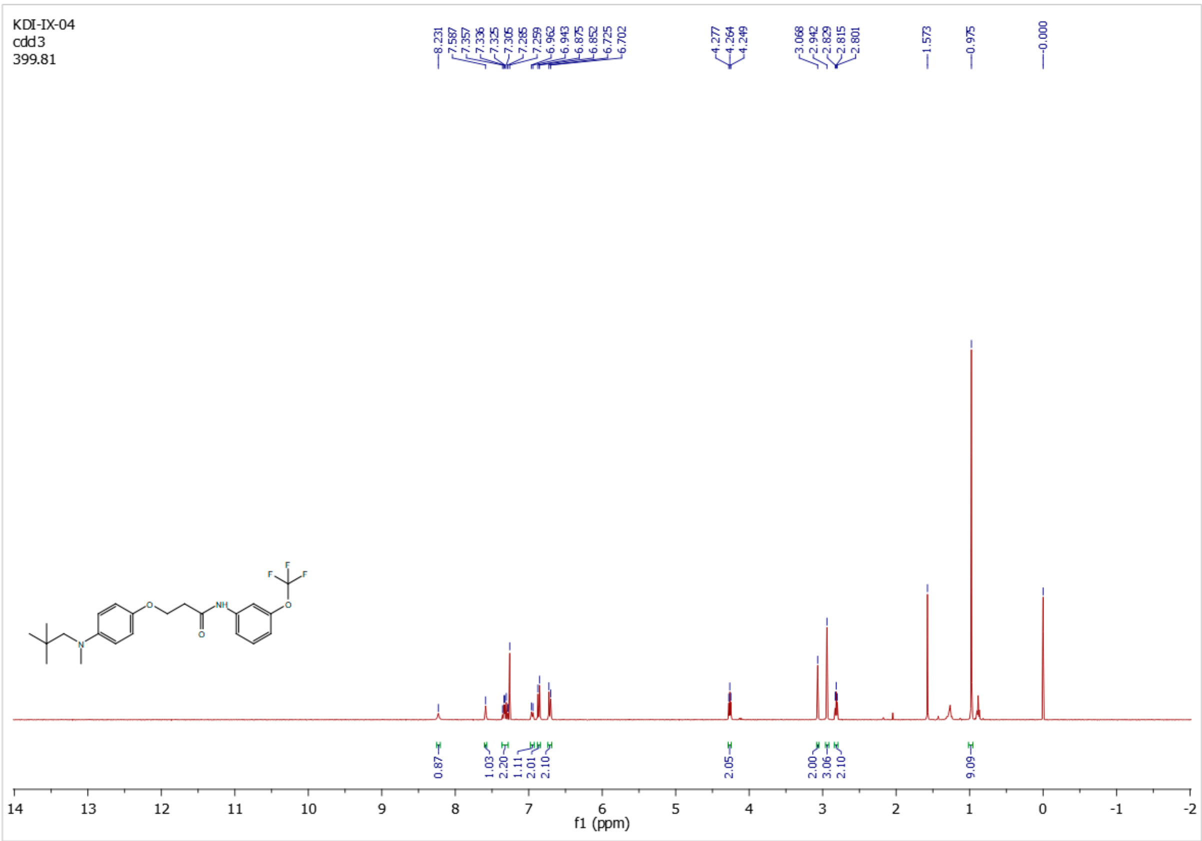

Figure S27. ^{13}C NMR (100 MHz, DMSO-d_6) spectrum of compound **22**

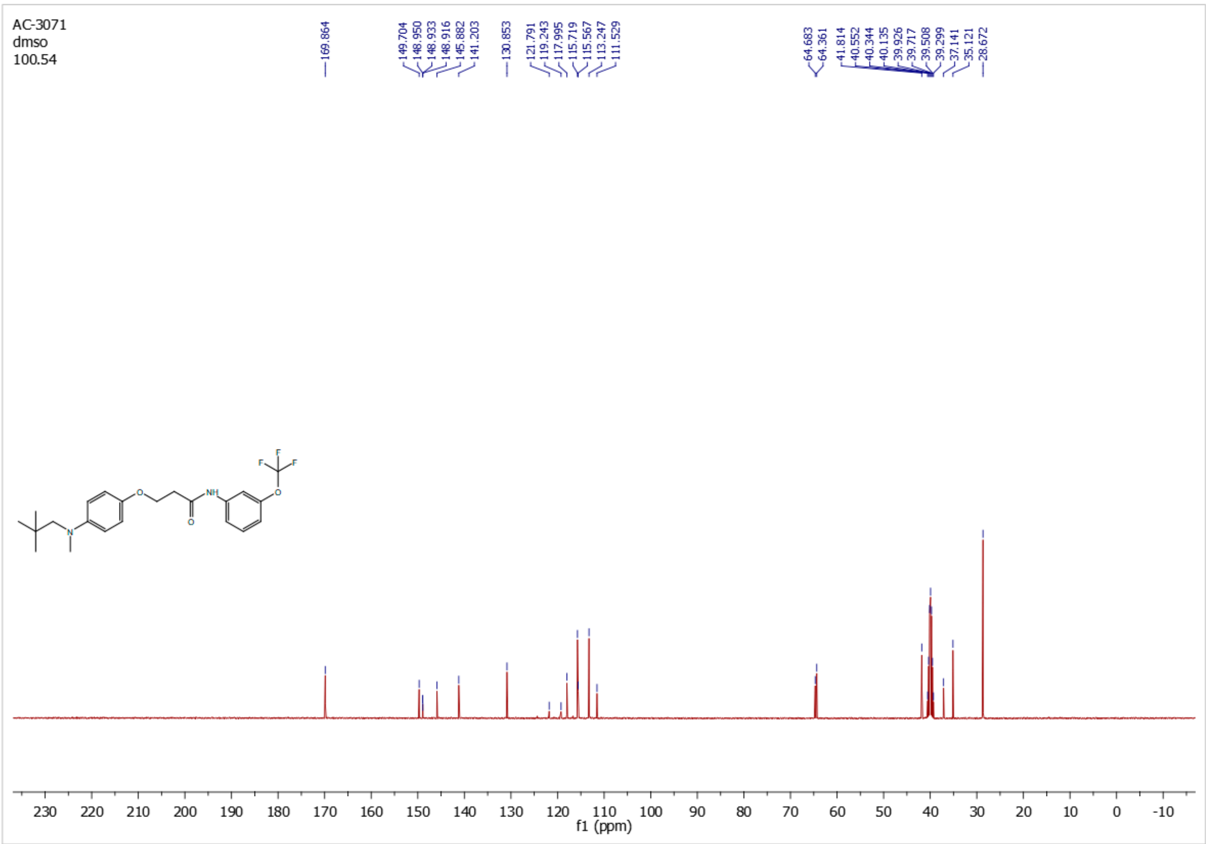

Figure S28. ^{19}F NMR (376 MHz, $CDCl_3$) spectrum of compound **22**

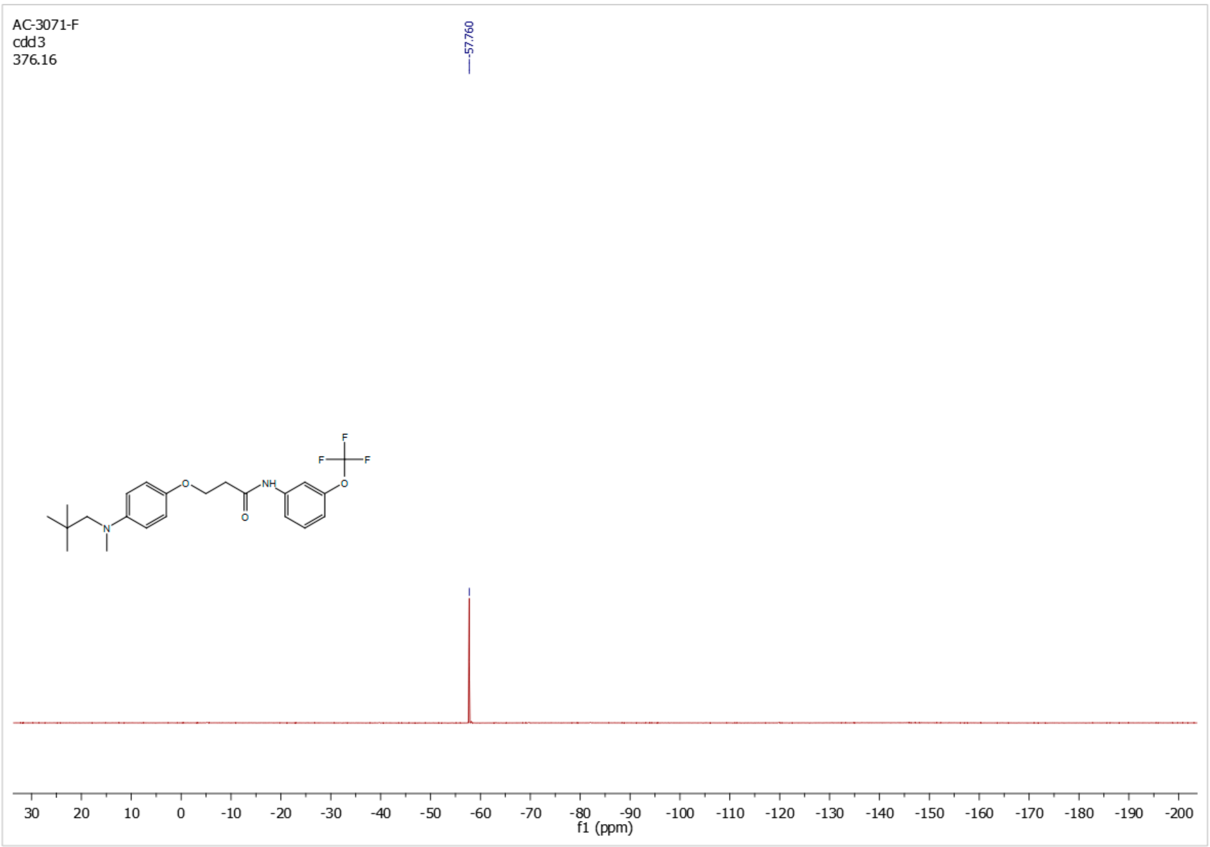

Figure S29. ^{1}H NMR (400 MHz, DMSO-d_6) spectrum of compound **30**

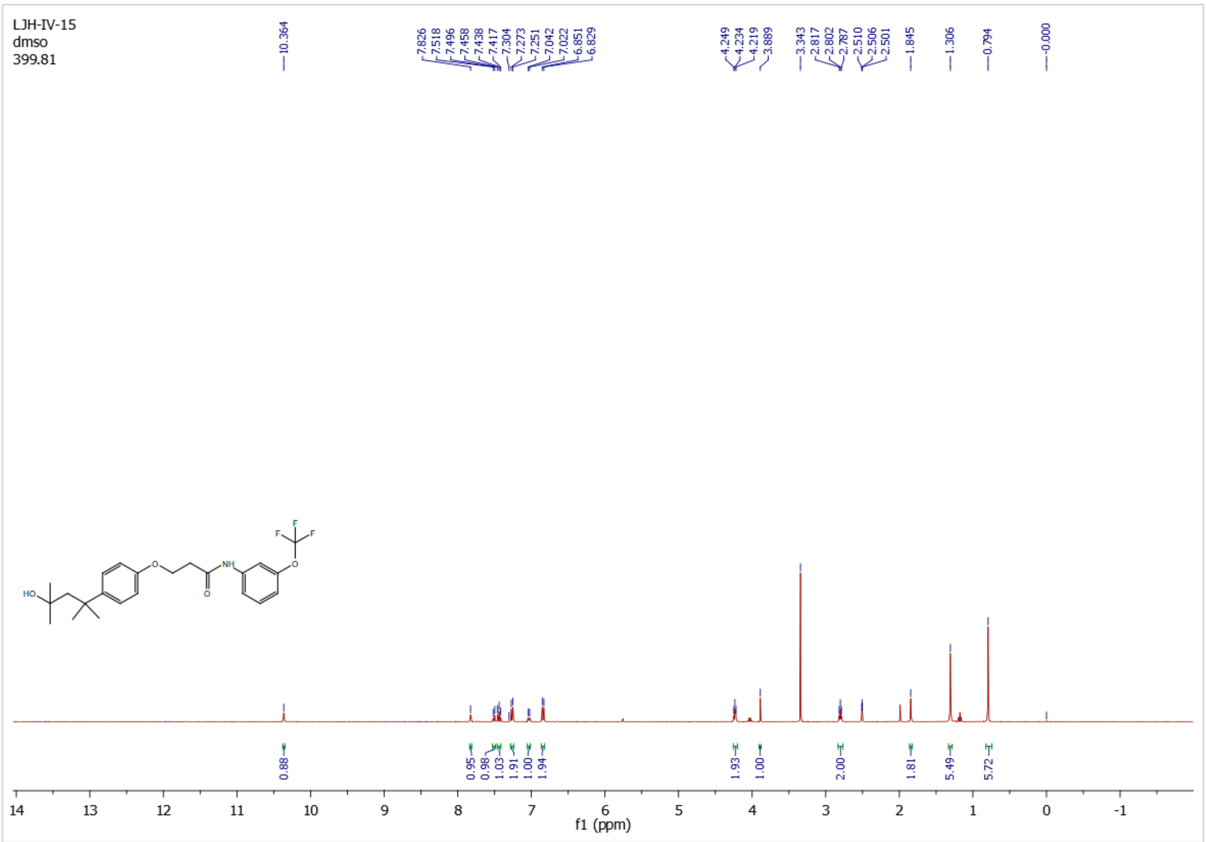

Figure S30. ^{13}C NMR (100 MHz, DMSO-d_6) spectrum of compound **30**

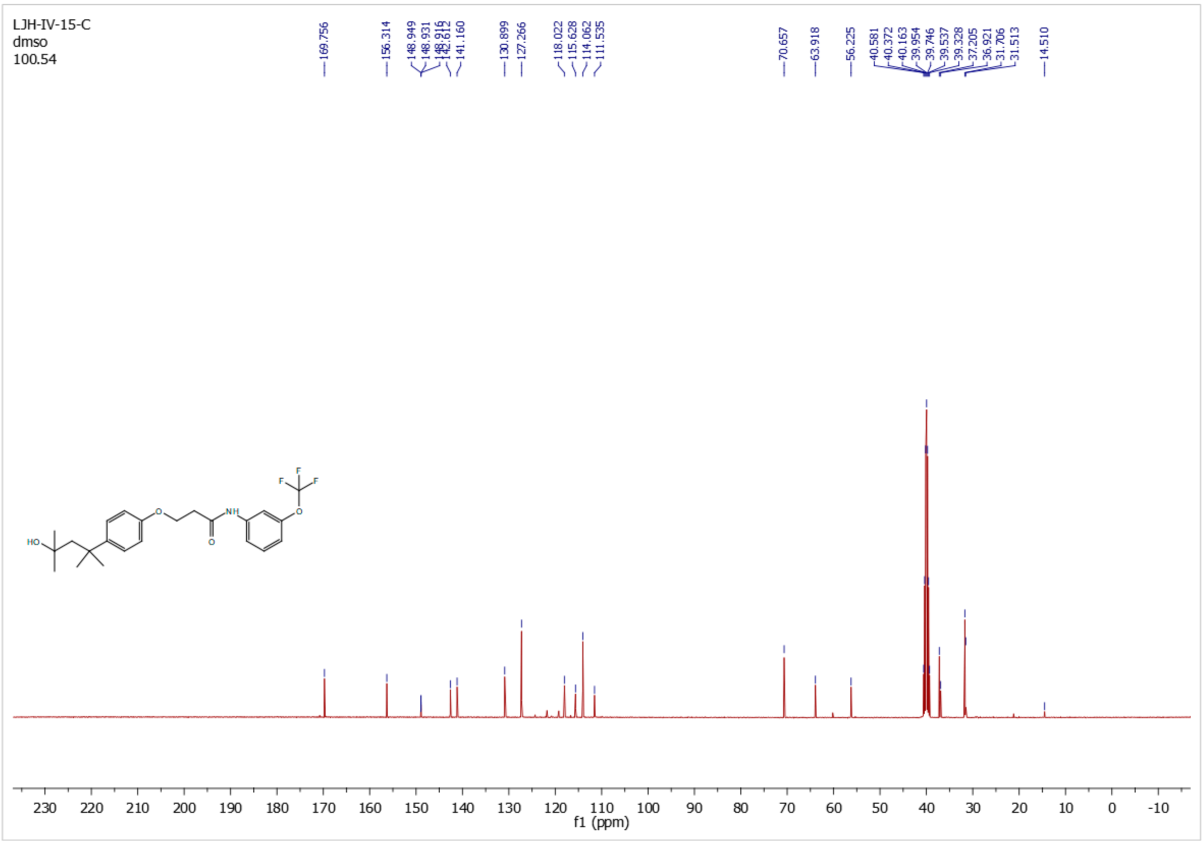

Figure S31. ^{19}F NMR (376 MHz, $CDCl_3$) spectrum of compound **30**

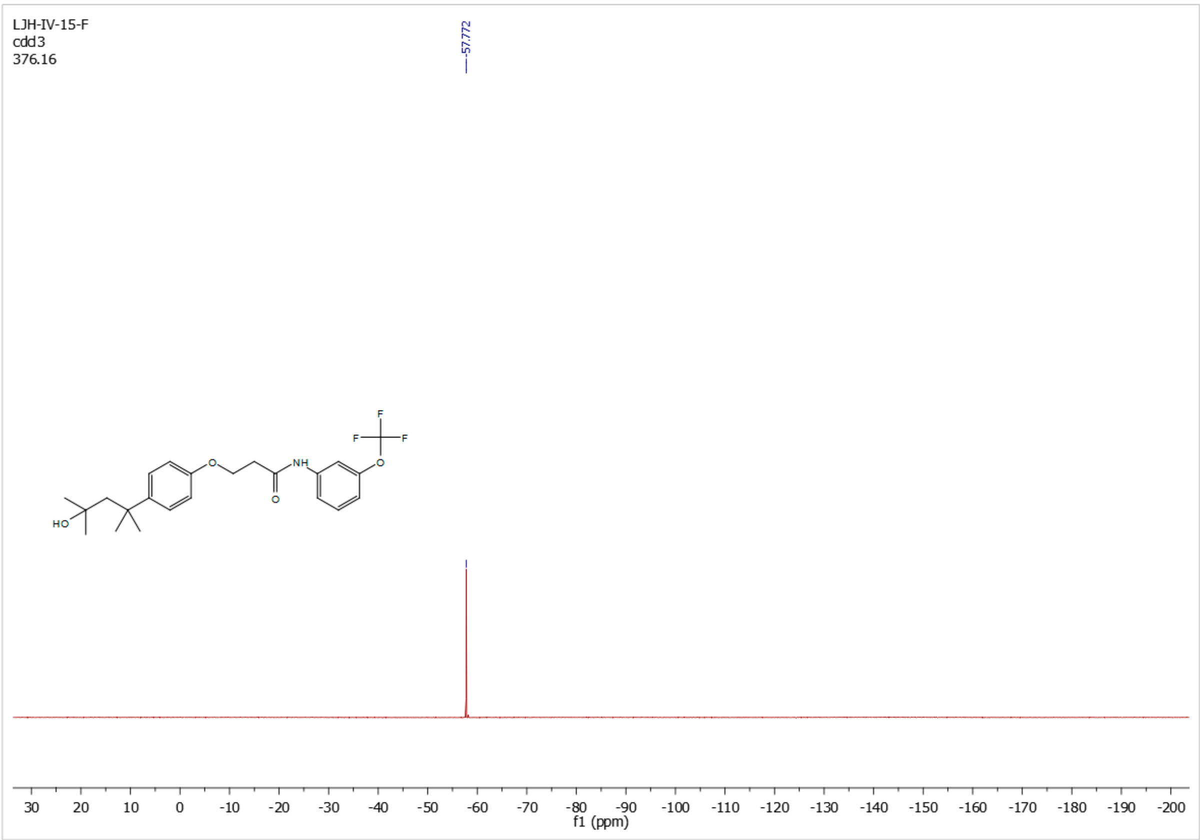

Figure S32. ^{1}H NMR (400 MHz, $CDCl_3$) spectrum of compound **35a**

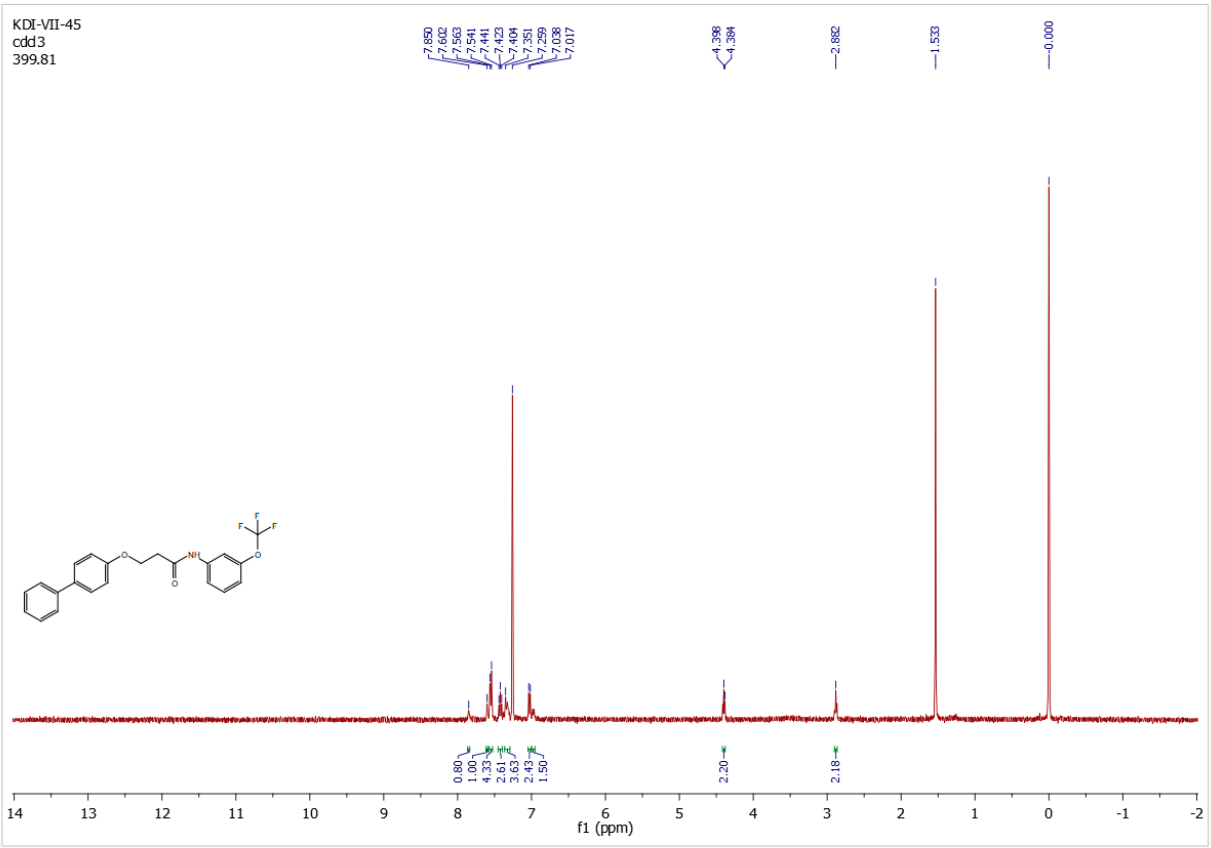

Figure S33. ^{13}C NMR (100 MHz, $CDCl_3$) spectrum of compound **35a**

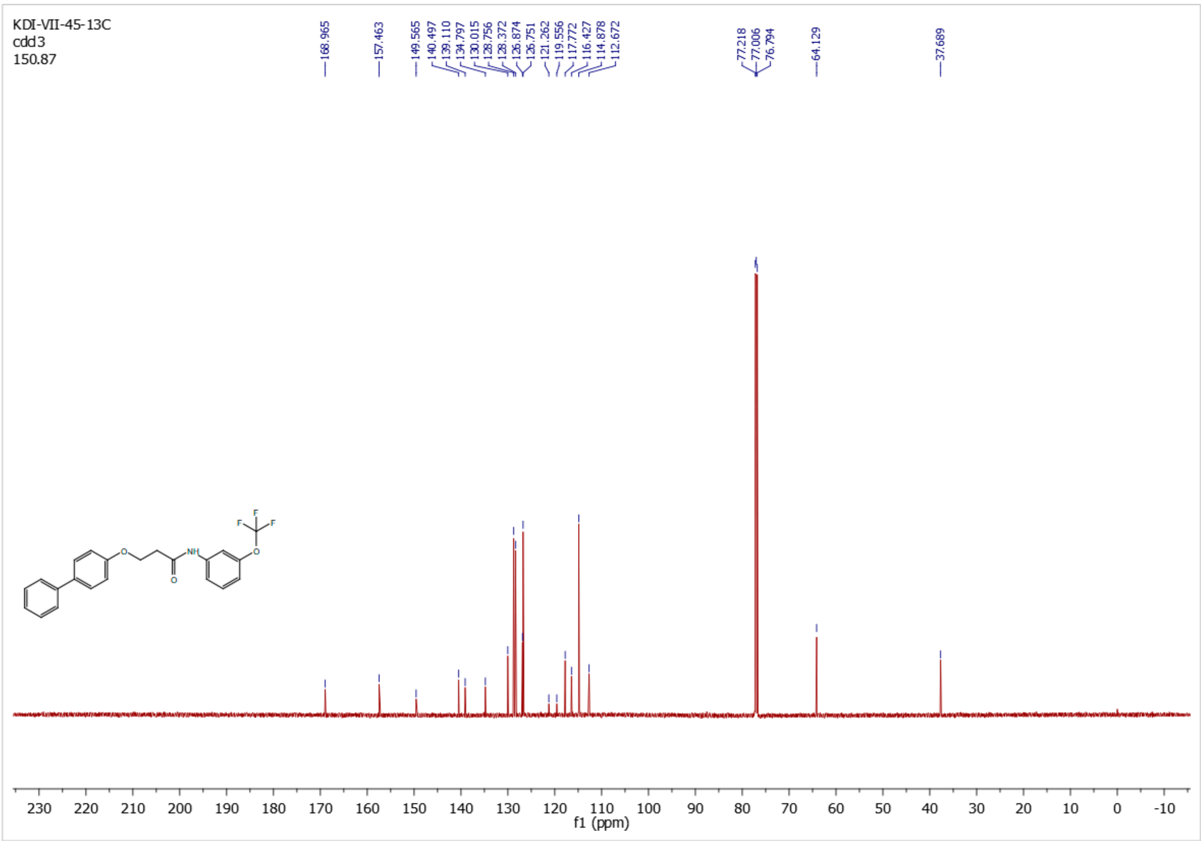

Figure S34. ^{19}F NMR (376 MHz, $CDCl_3$) spectrum of compound **35a**

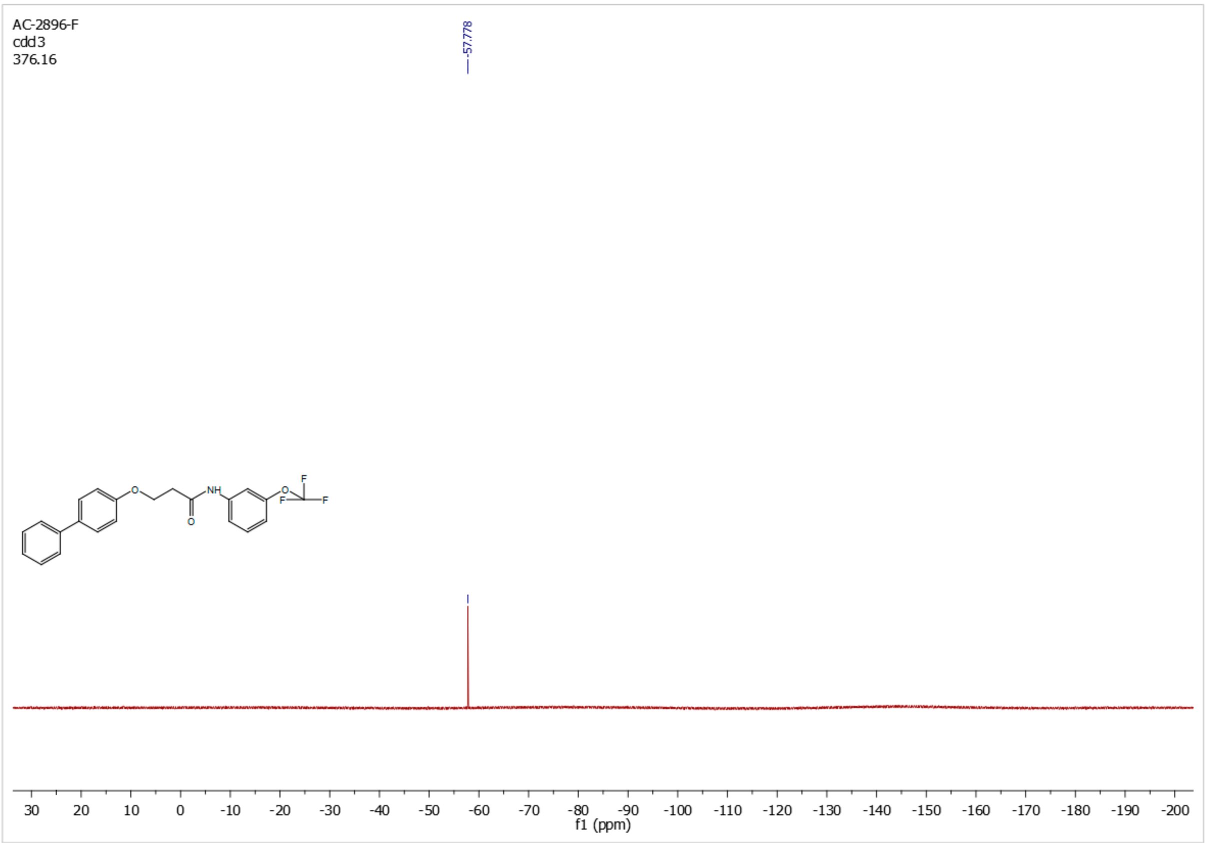

Figure S35. ^{1}H NMR (400 MHz, $CDCl_3$) spectrum of compound **35b**

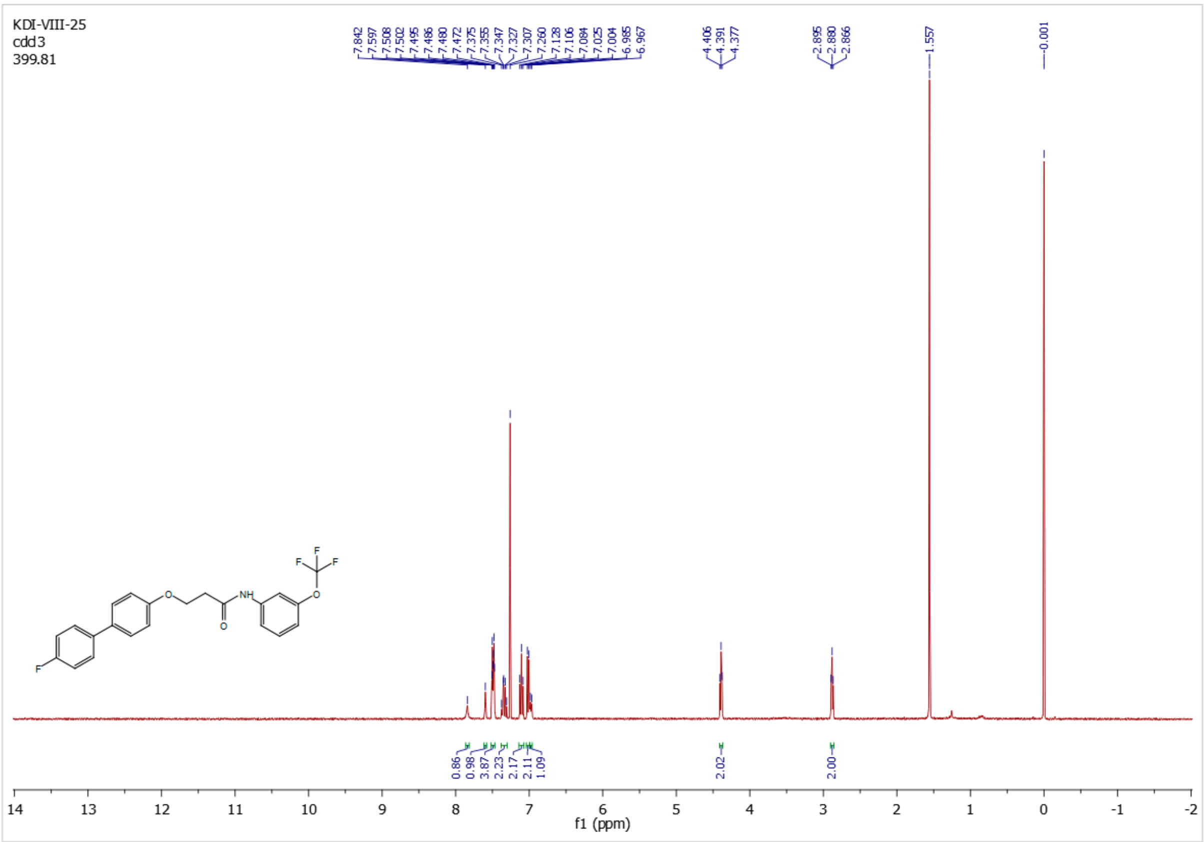

Figure S36. ^{13}C NMR (100 MHz, $CDCl_3$) spectrum of compound **35b**

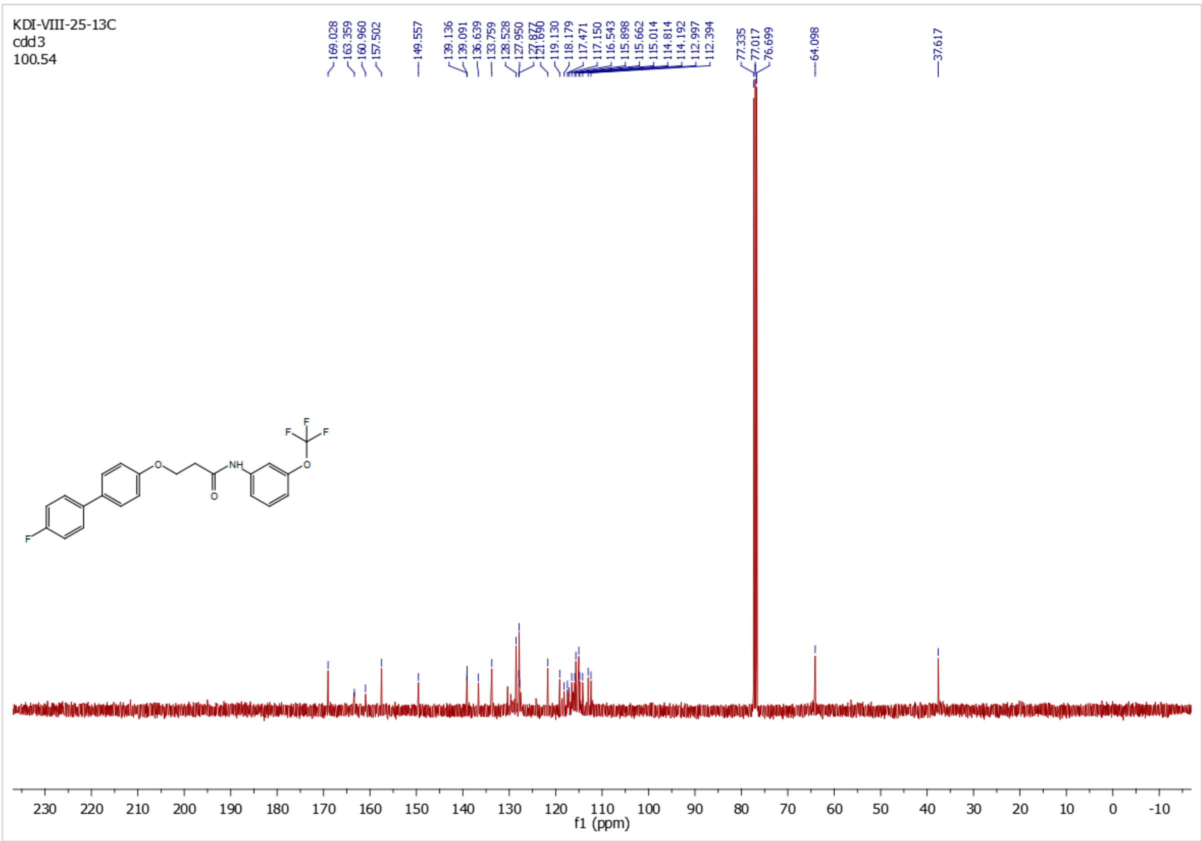

Figure S37. ^{19}F NMR (376 MHz, $CDCl_3$) spectrum of compound **35b**

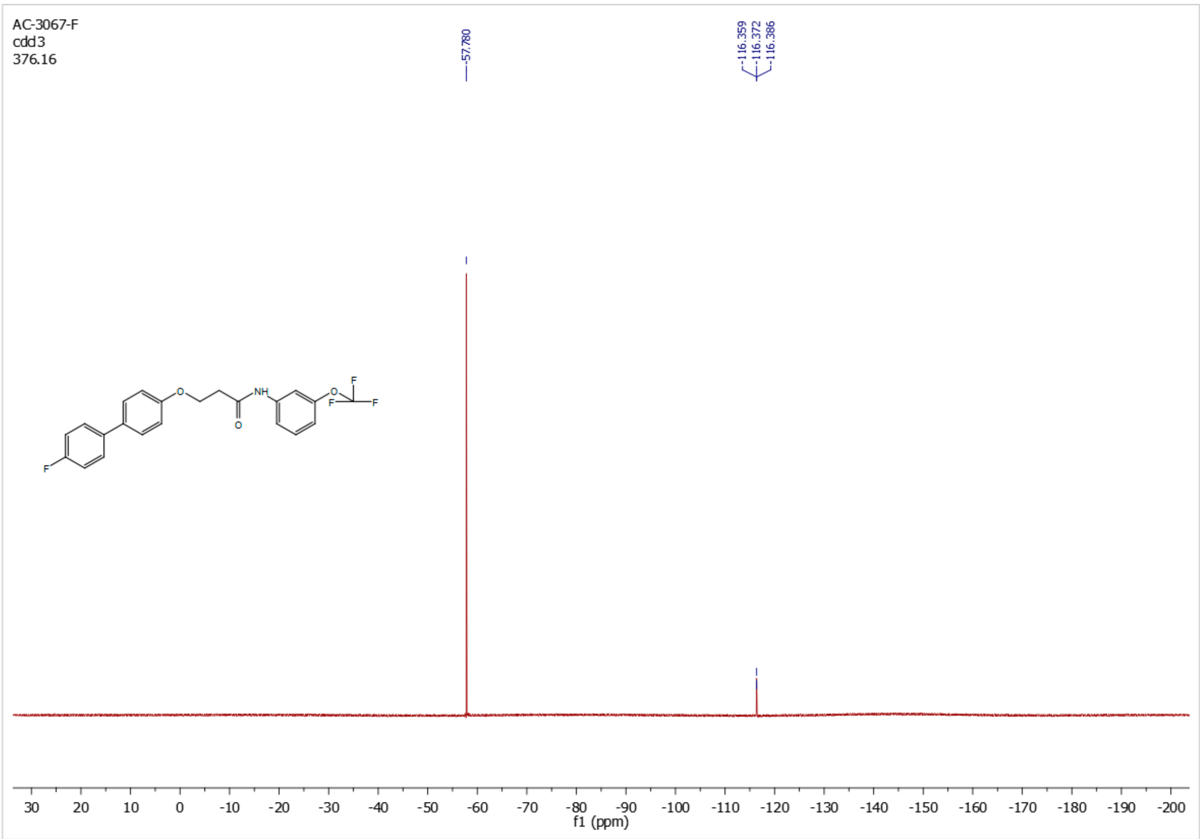

Figure S38. ^{1}H NMR (400 MHz, $CDCl_3$) spectrum of compound **39a**

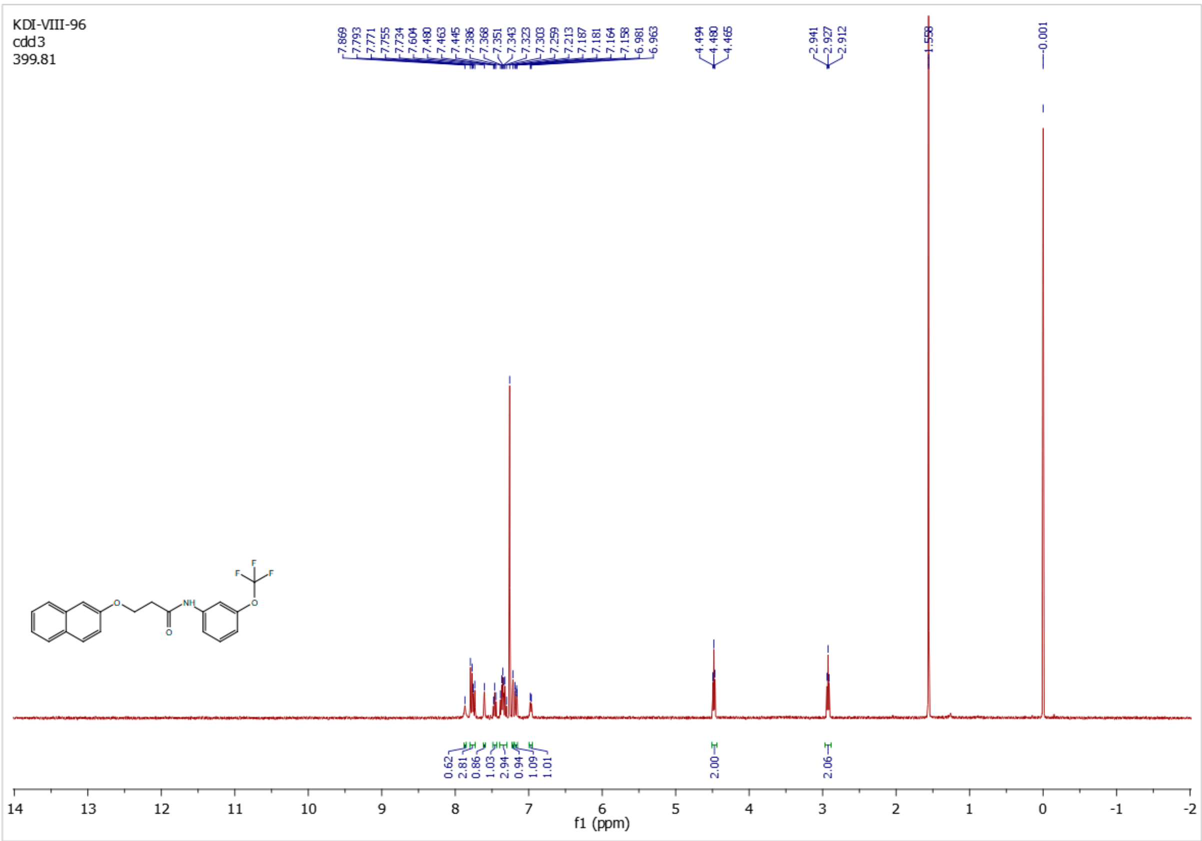

Figure S39. ^{13}C NMR (100 MHz, $CDCl_3$) spectrum of compound **39a**

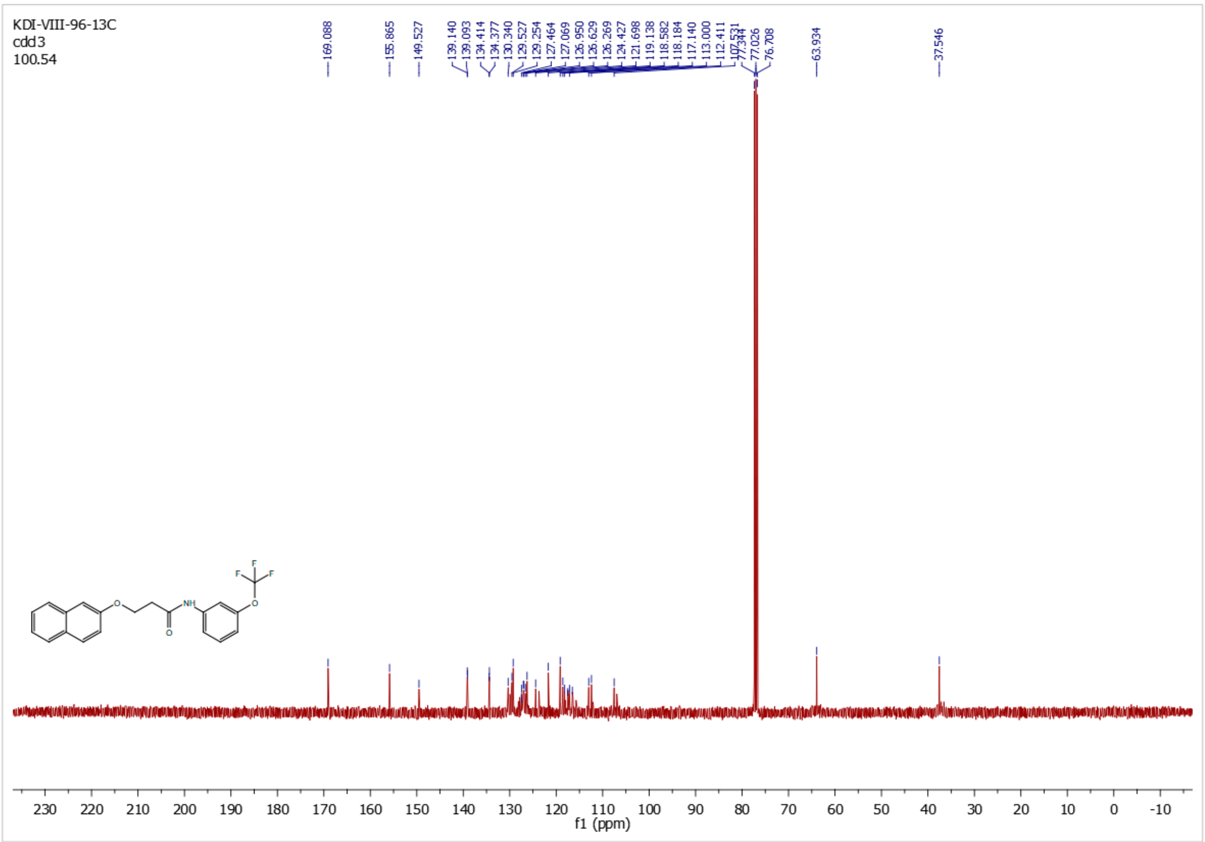

Figure S40. ^{19}F NMR (376 MHz, $CDCl_3$) spectrum of compound **39a**

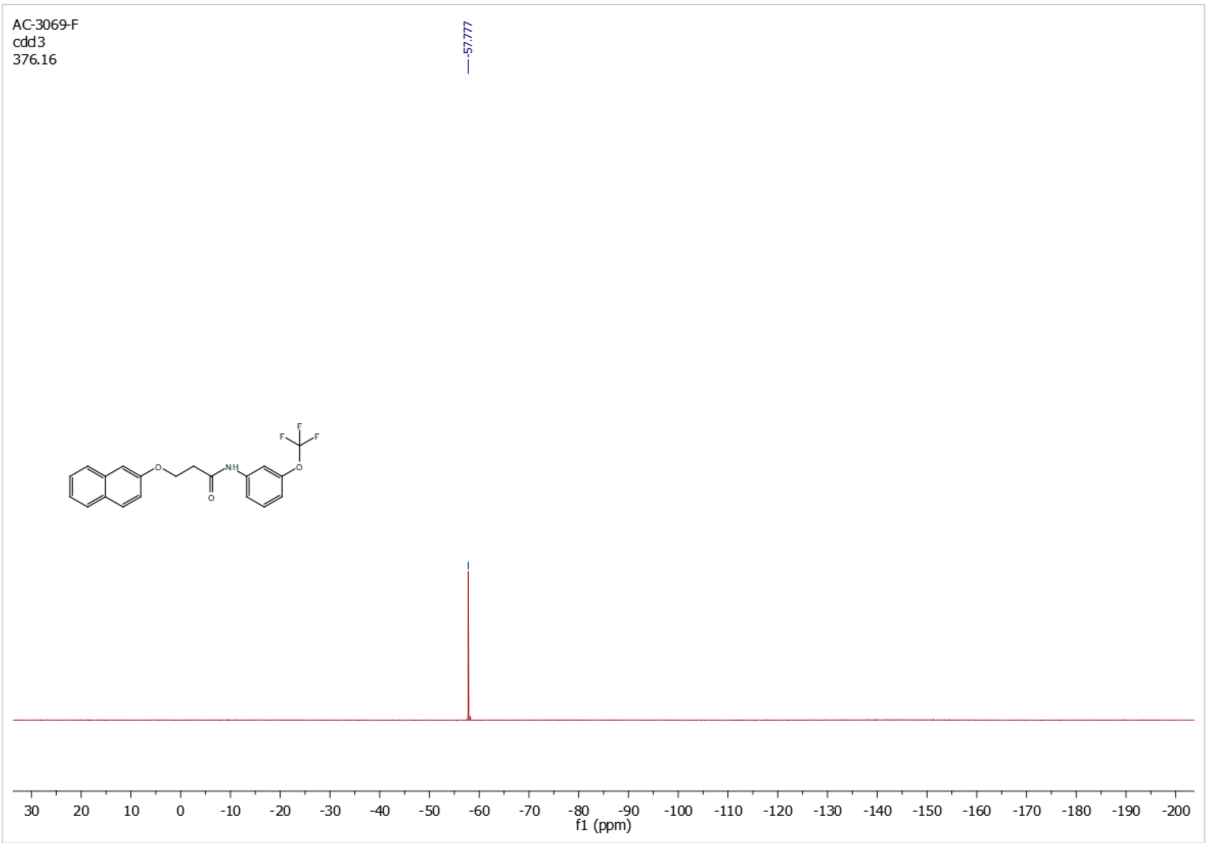

Figure S41. ^{1}H NMR (400 MHz, $CDCl_3$) spectrum of compound **39b**

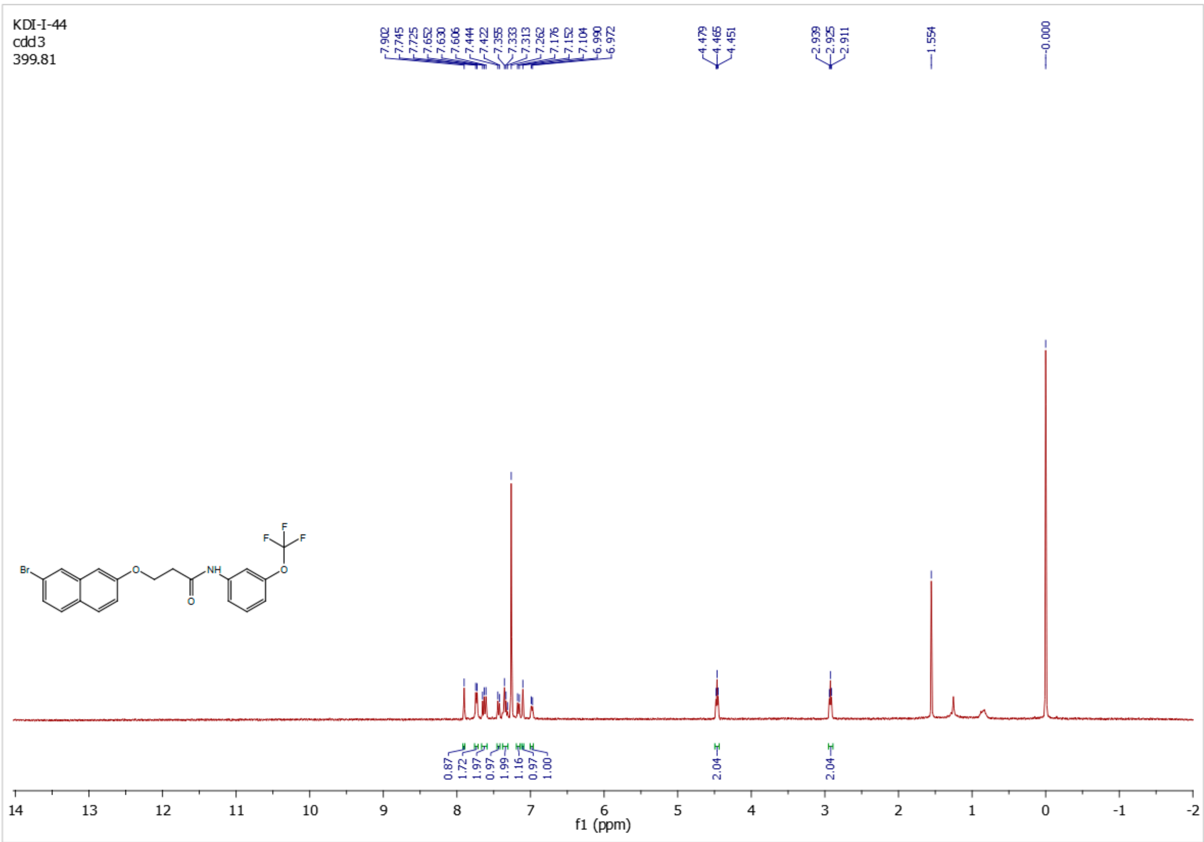

Figure S42. ^{13}C NMR (100 MHz, $CDCl_3$) spectrum of compound **39b**

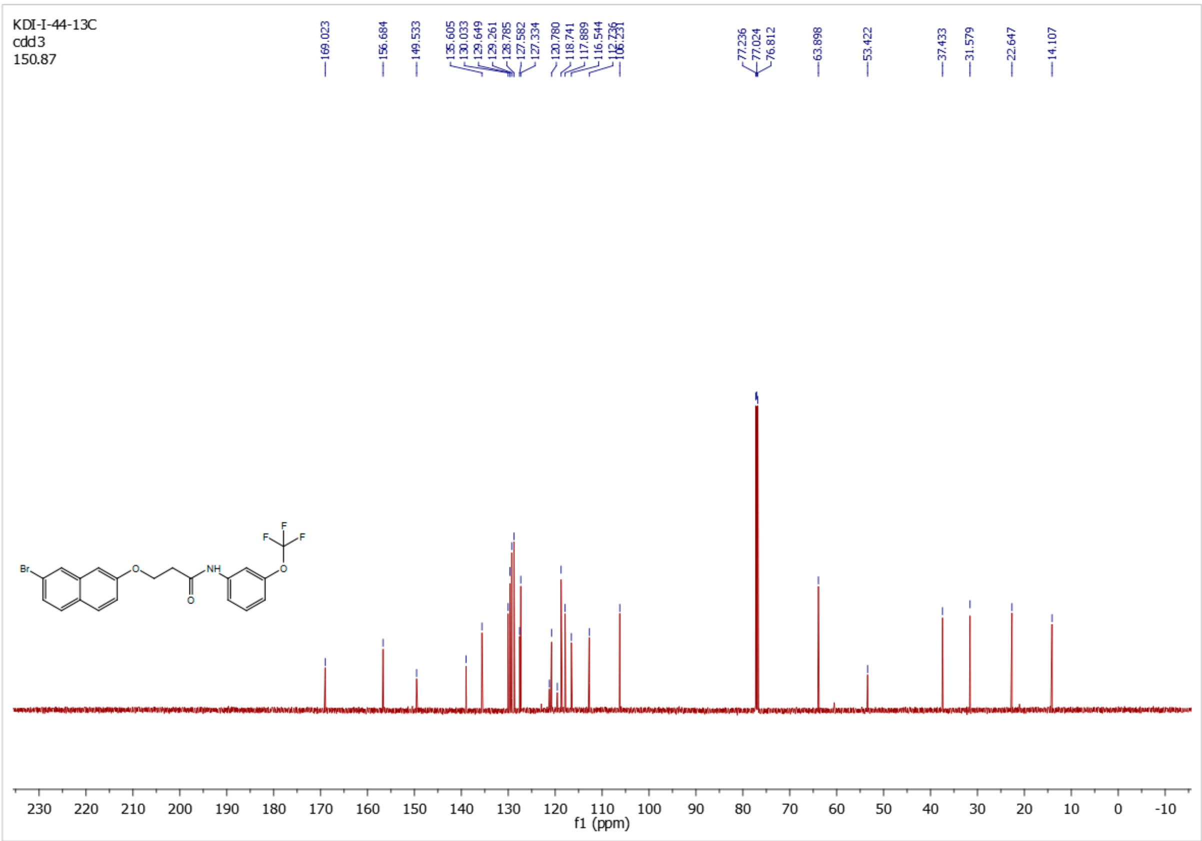

Figure S43. ^{19}F NMR (376 MHz, $CDCl_3$) spectrum of compound **39b**

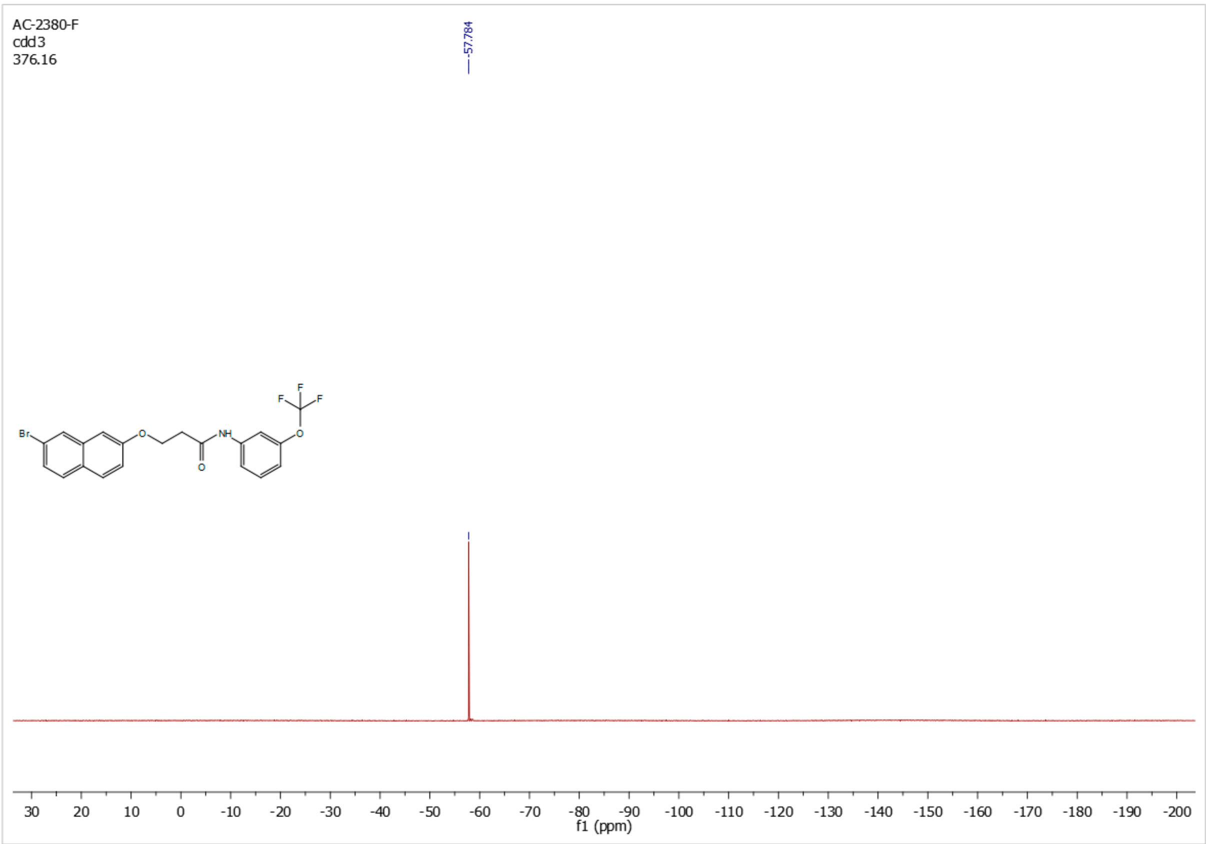

Figure S44. ^{1}H NMR (400 MHz, $CDCl_3$) spectrum of compound **39c**

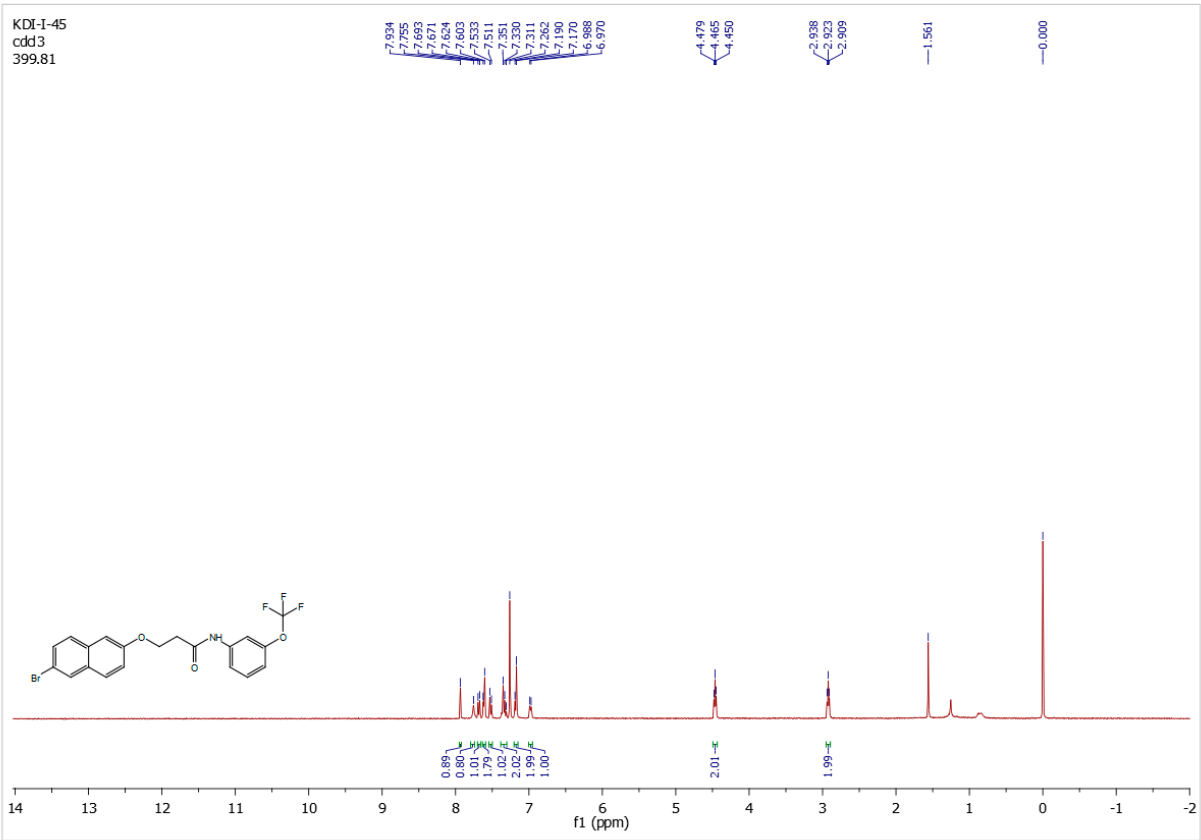

Figure S45. ^{13}C NMR (100 MHz, $CDCl_3$) spectrum of compound **39c**

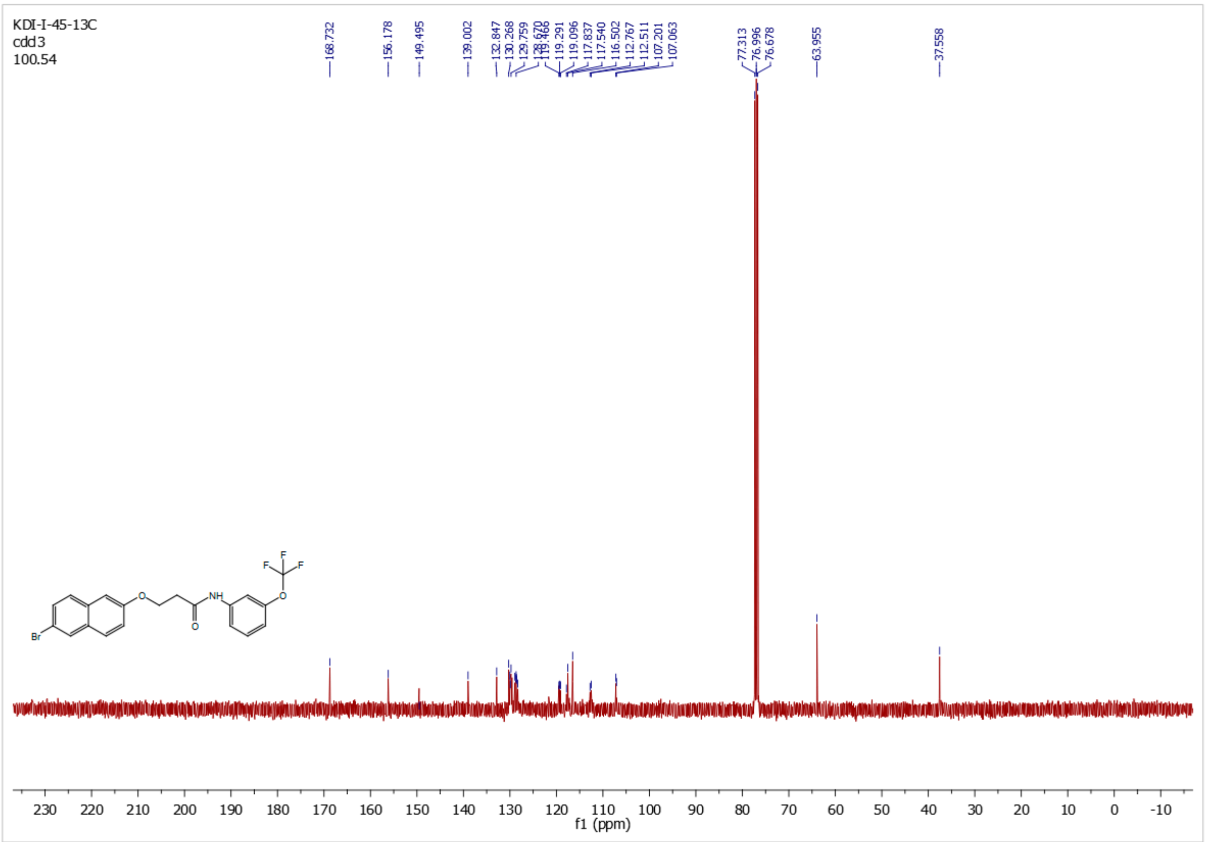

Figure S46. ^{19}F NMR (376 MHz, $CDCl_3$) spectrum of compound **39c**

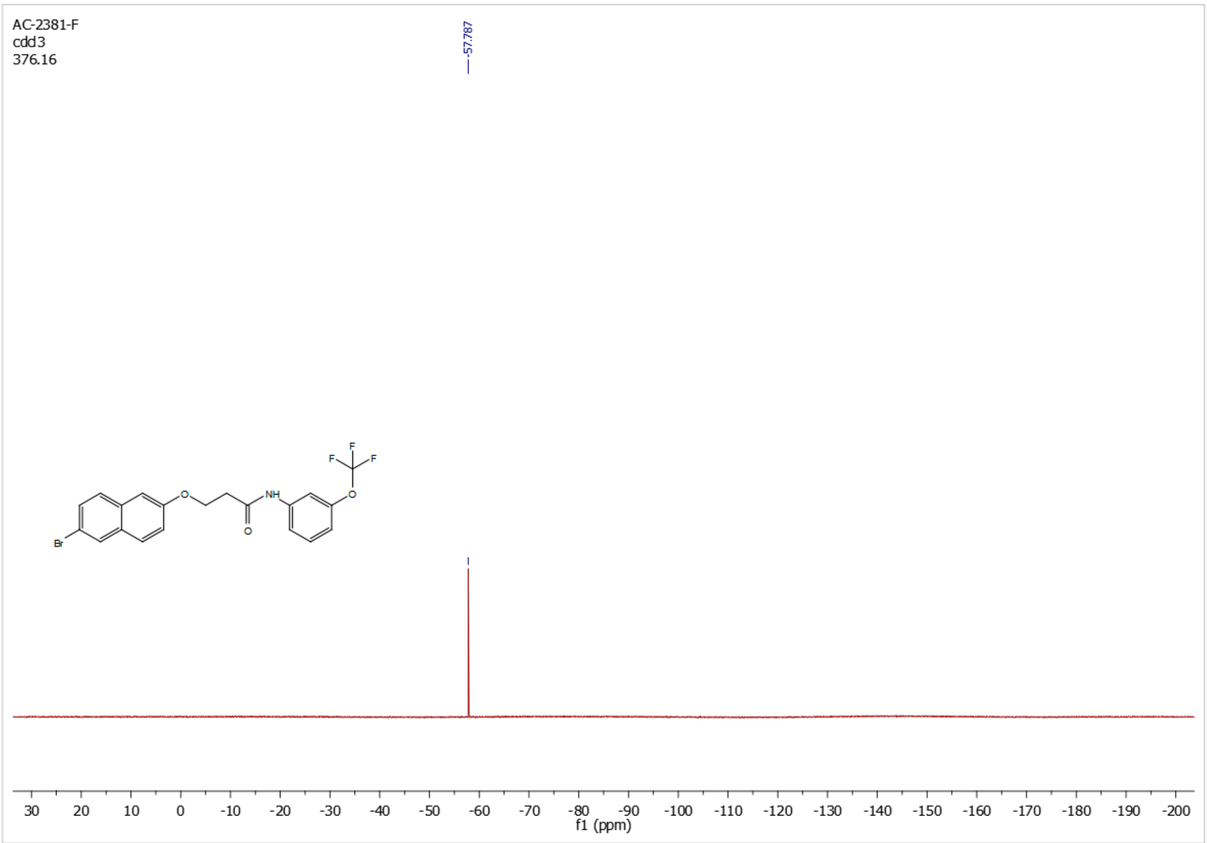

Figure S47. ^{1}H NMR (400 MHz, $CDCl_3$) spectrum of compound **39d**

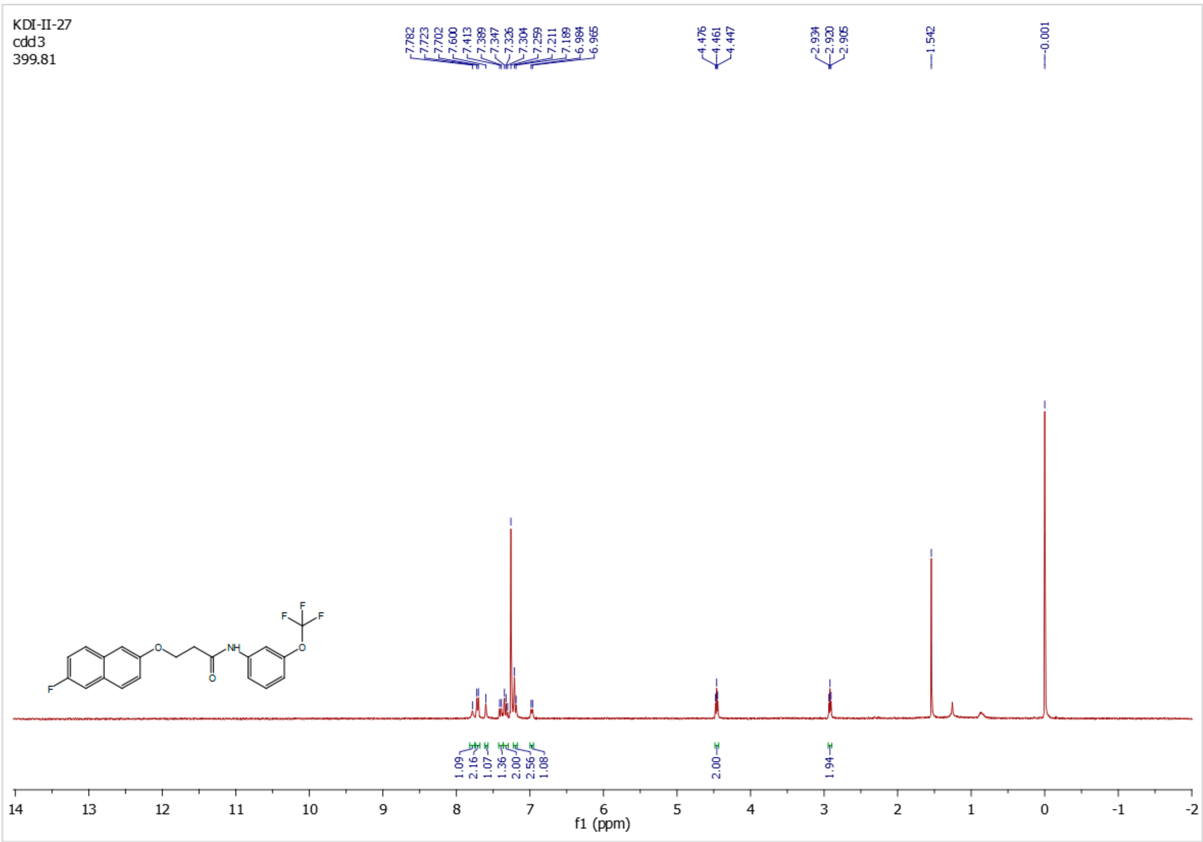

Figure S48. ^{13}C NMR (100 MHz, $CDCl_3$) spectrum of compound **39d**

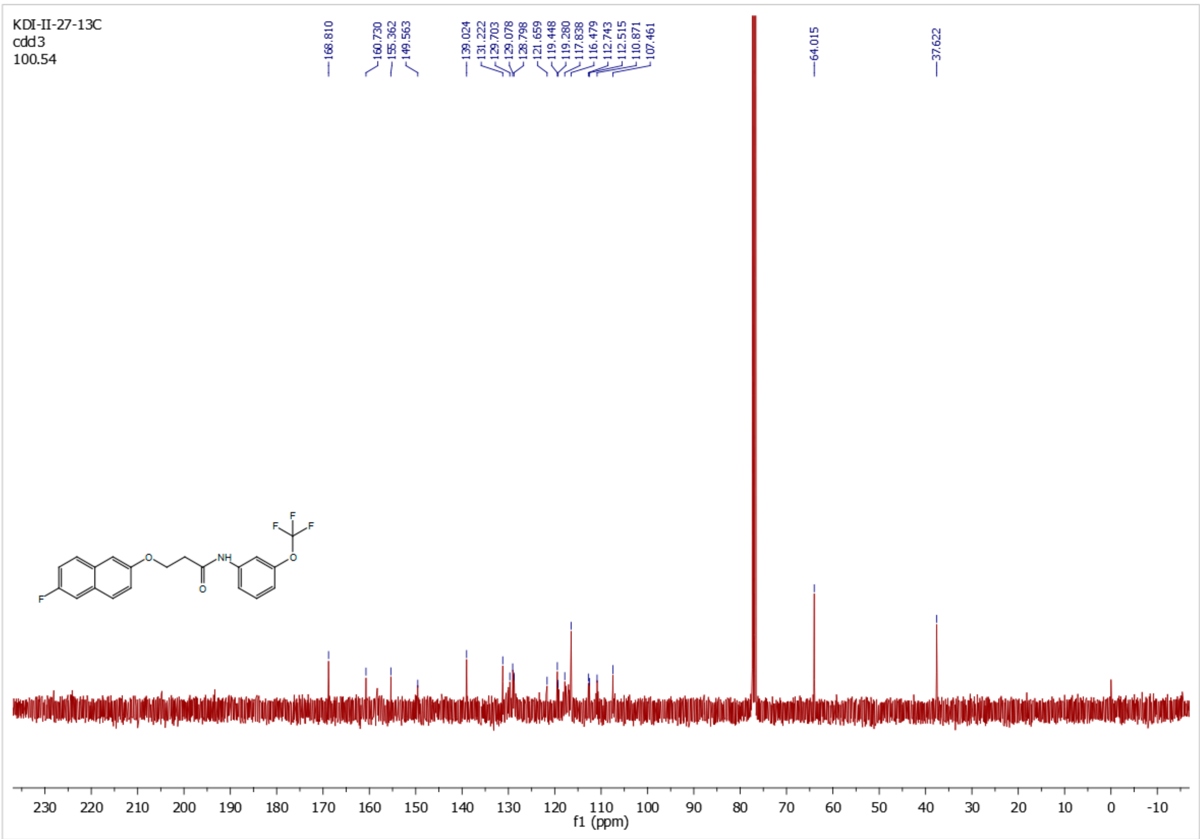

Figure S49. ^{19}F NMR (376 MHz, $CDCl_3$) spectrum of compound **39d**

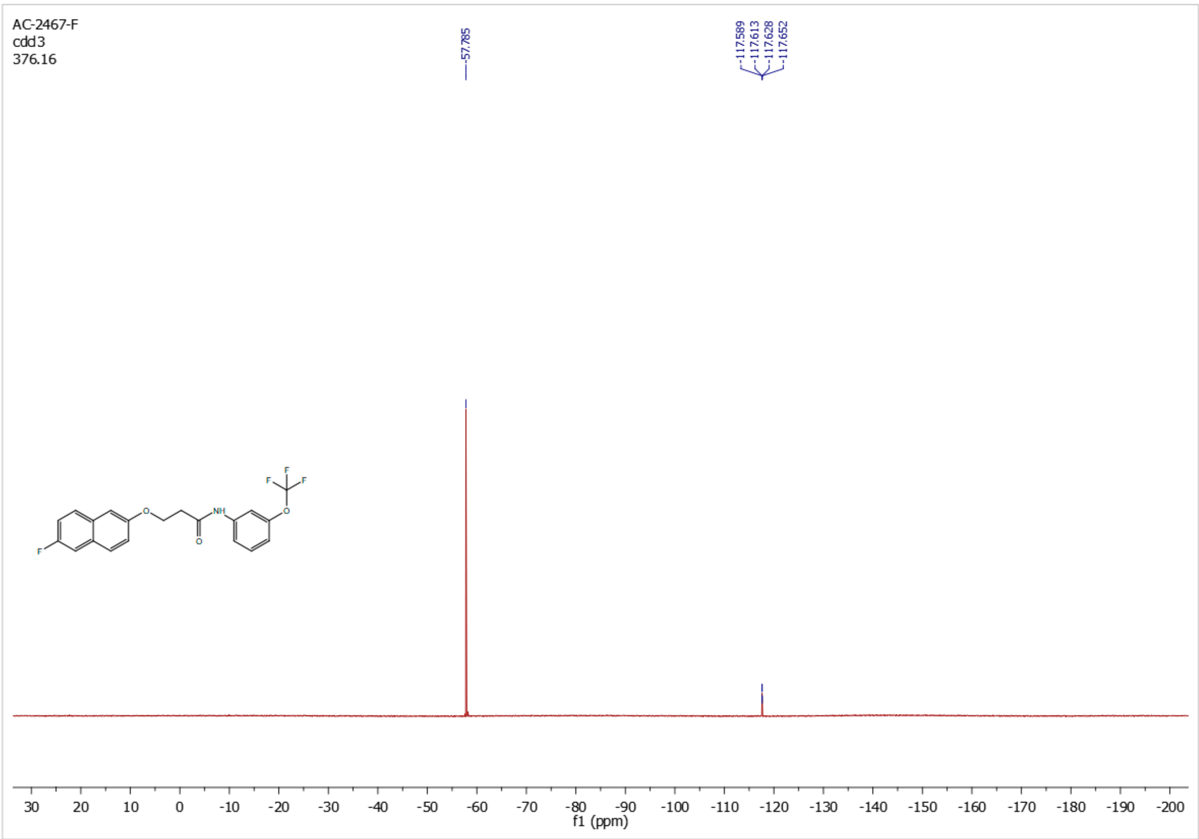

Figure S50. ^{1}H NMR (400 MHz, $CDCl_3$) spectrum of compound **44a**

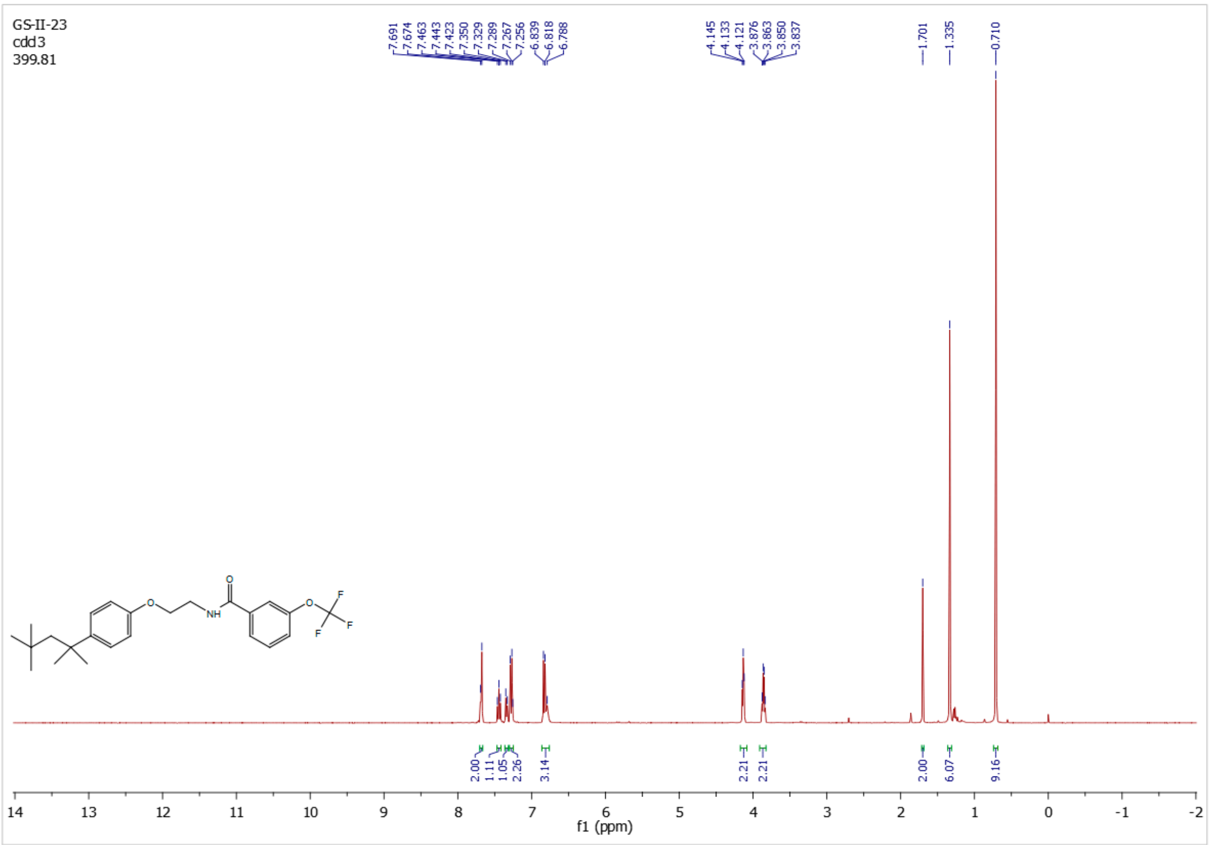

Figure S51. ^{13}C NMR (100 MHz, $CDCl_3$) spectrum of compound **44a**

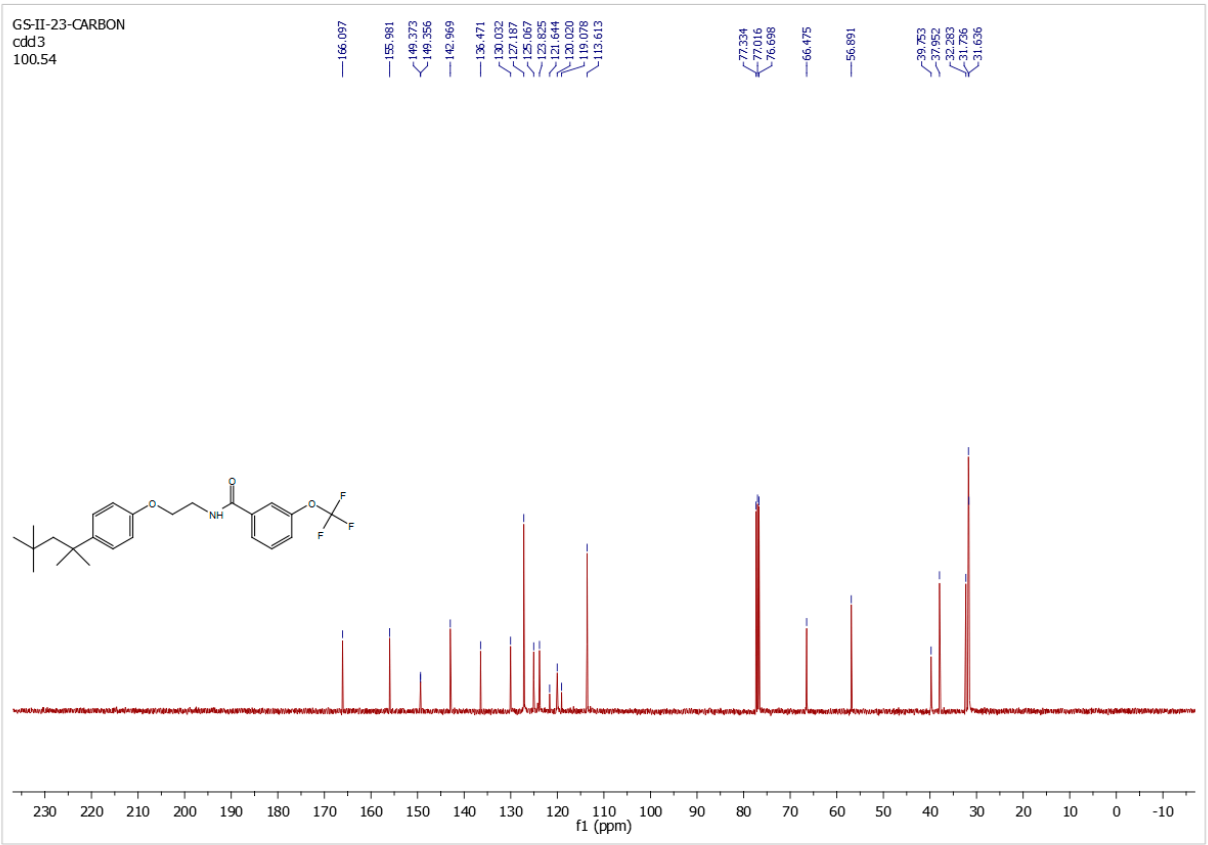

Figure S52. ^{19}F NMR (376 MHz, $CDCl_3$) spectrum of compound **44a**

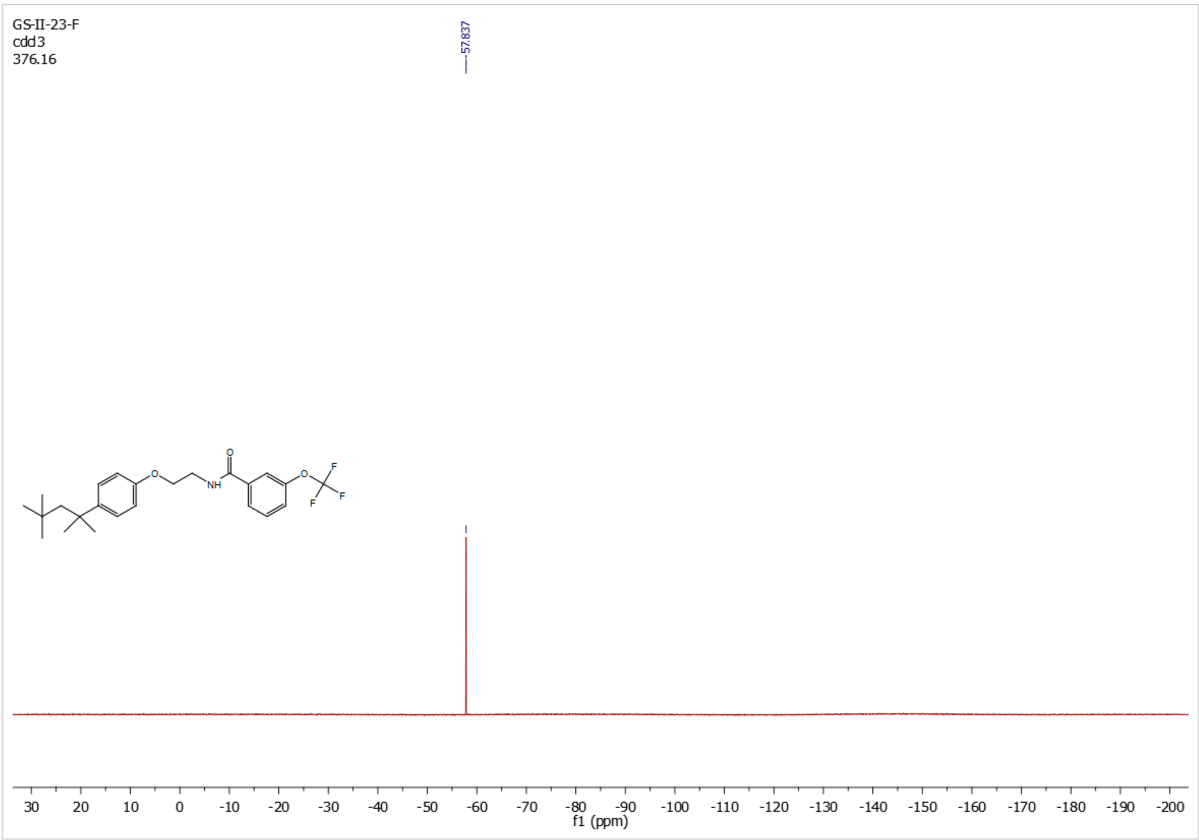

Figure S53. ^{1}H NMR (400 MHz, $CDCl_3$) spectrum of compound **44b**

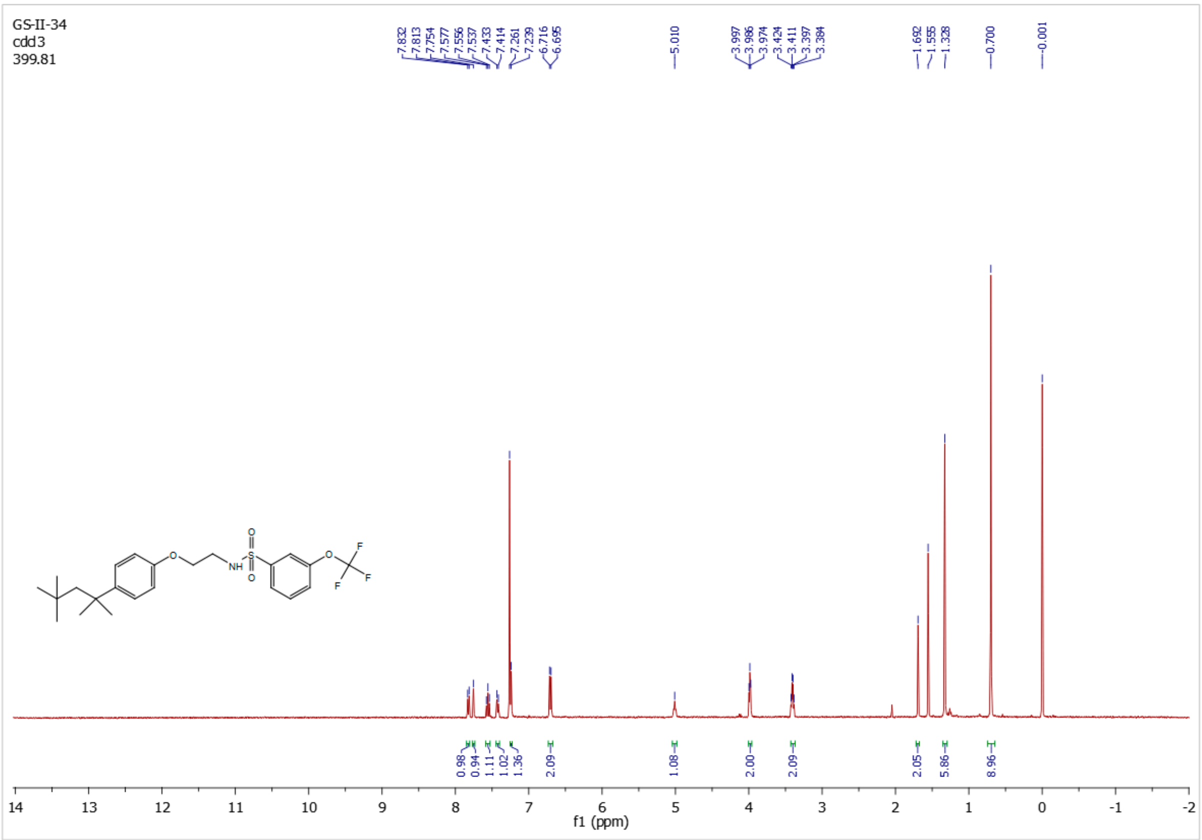

Figure S54. ^{13}C NMR (100 MHz, $CDCl_3$) spectrum of compound **44b**

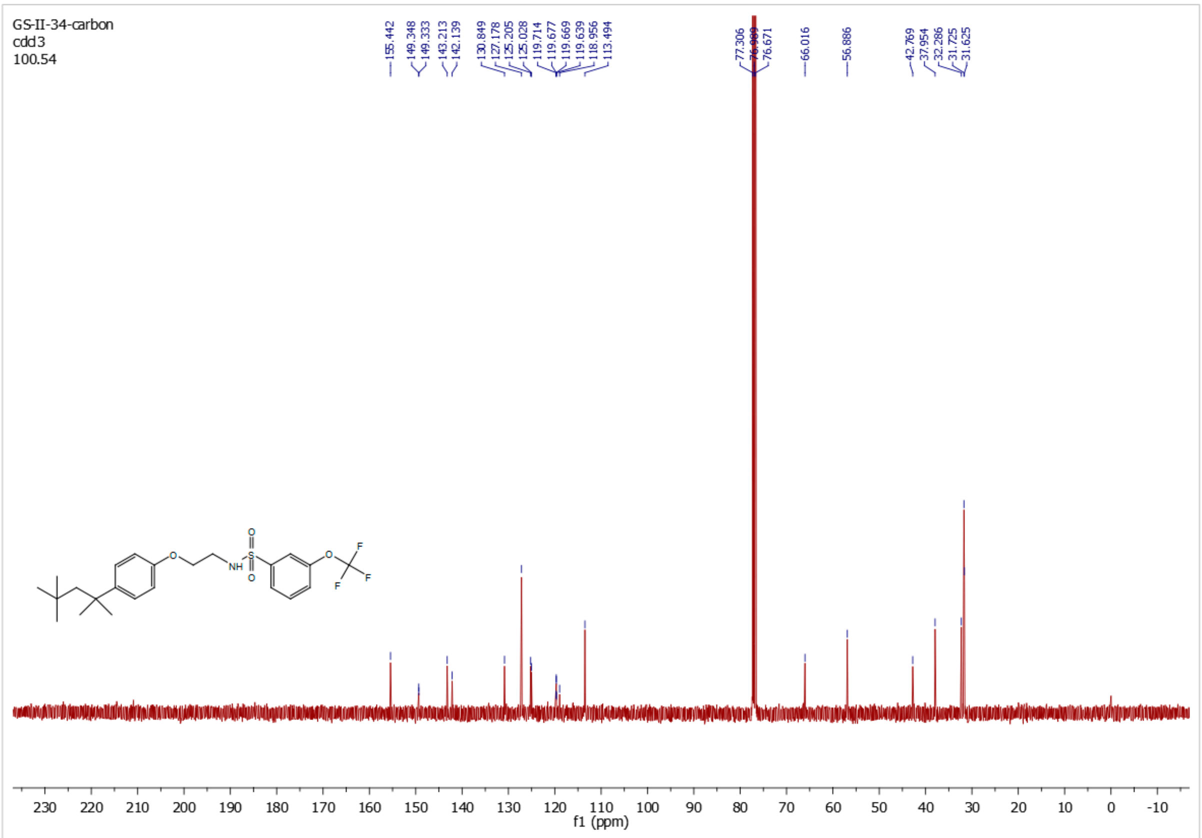

Figure S55. ^{19}F NMR (376 MHz, $CDCl_3$) spectrum of compound **44b**

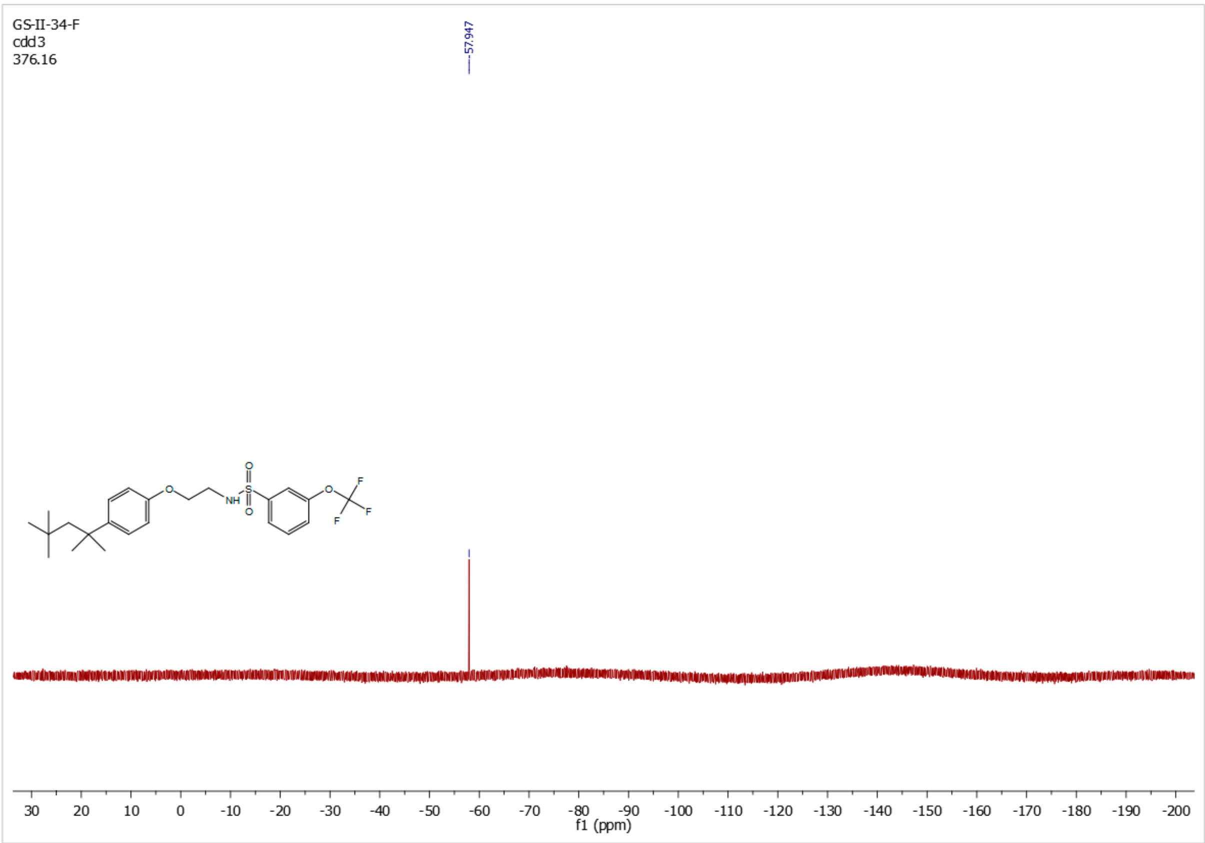

Figure S56. ^{1}H NMR (400 MHz, DMSO-d_6) spectrum of compound **50**

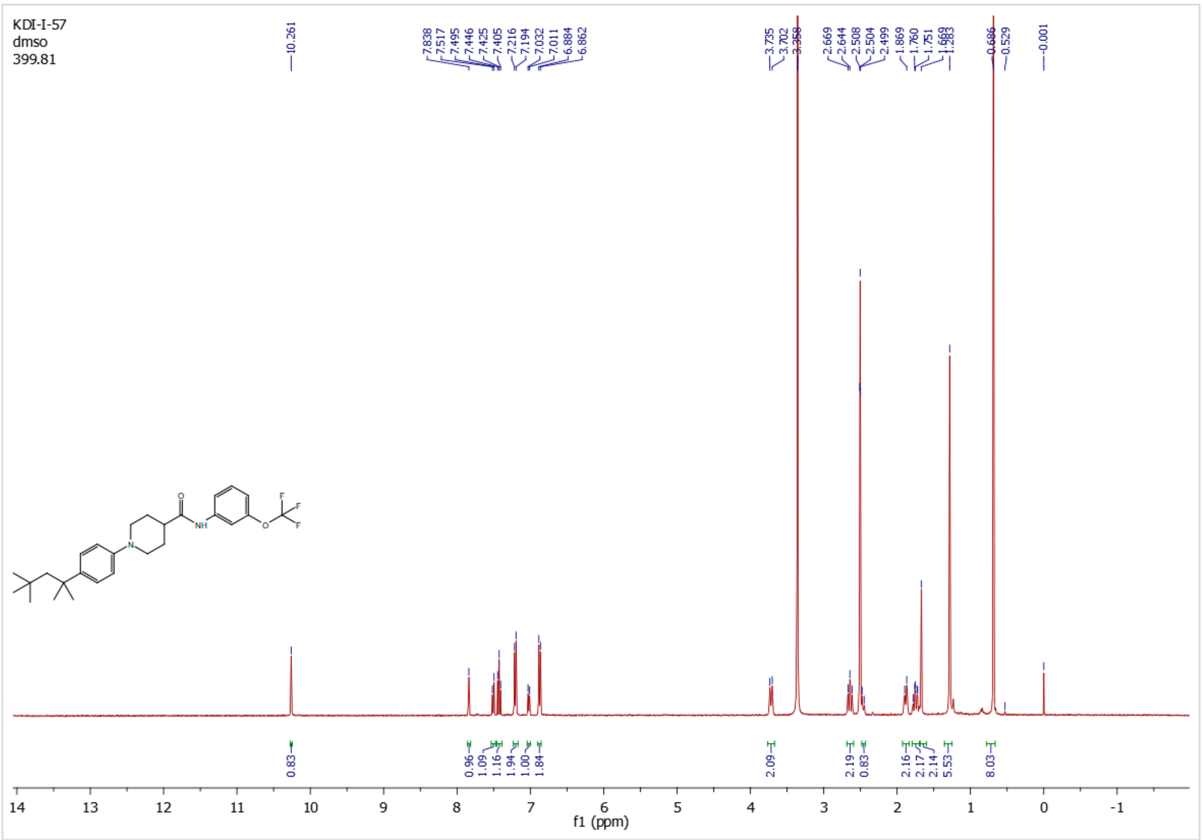

Figure S57. ^{13}C NMR (100 MHz, DMSO-d_6) spectrum of compound **50**

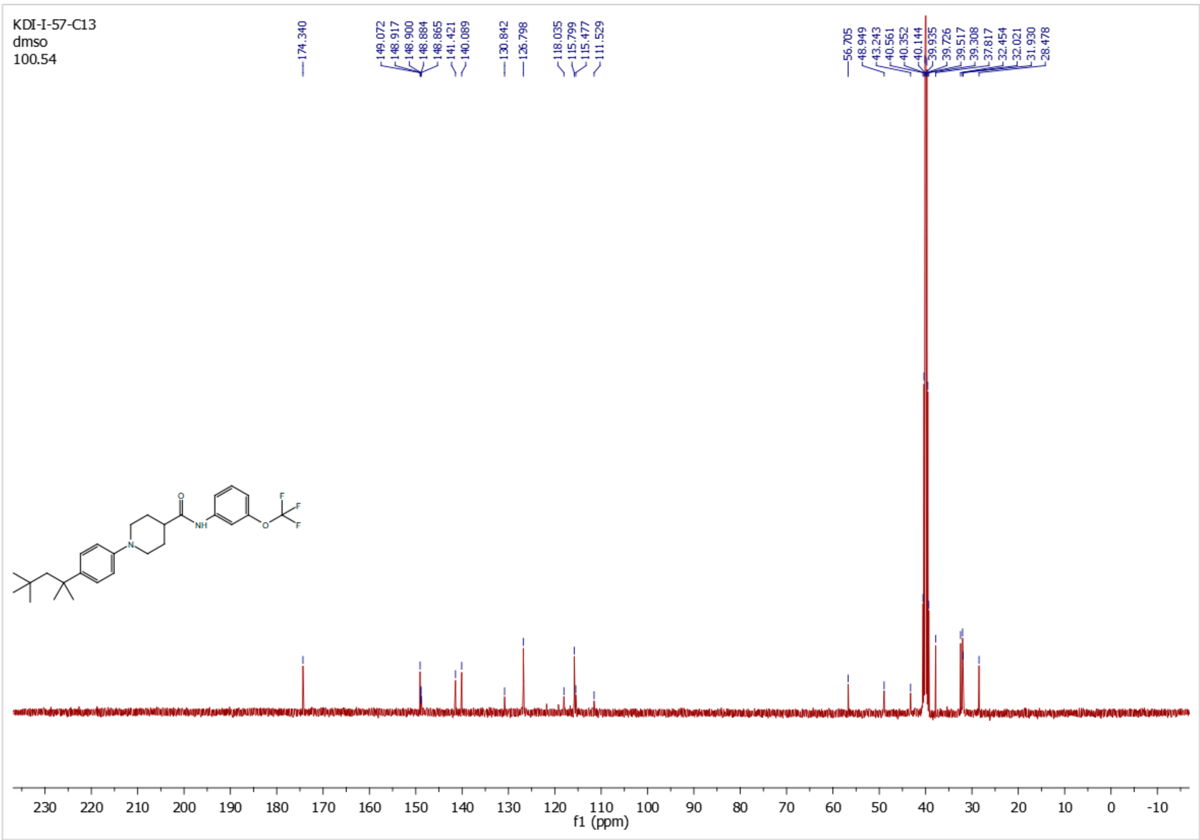

Figure S58. ^{19}F NMR (376 MHz, $CDCl_3$) spectrum of compound **50**

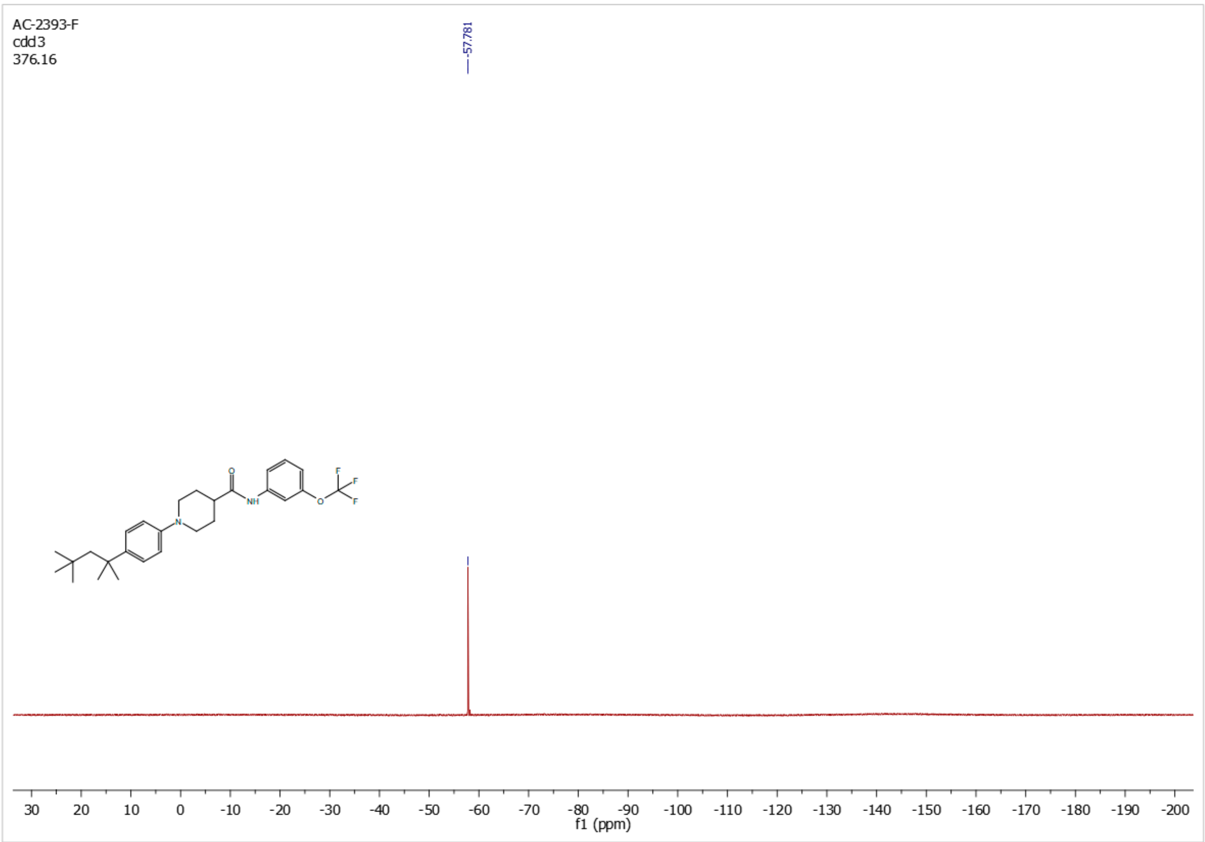

Figure S59. ^{1}H NMR (400 MHz, $CDCl_3$) spectrum of compound **51**

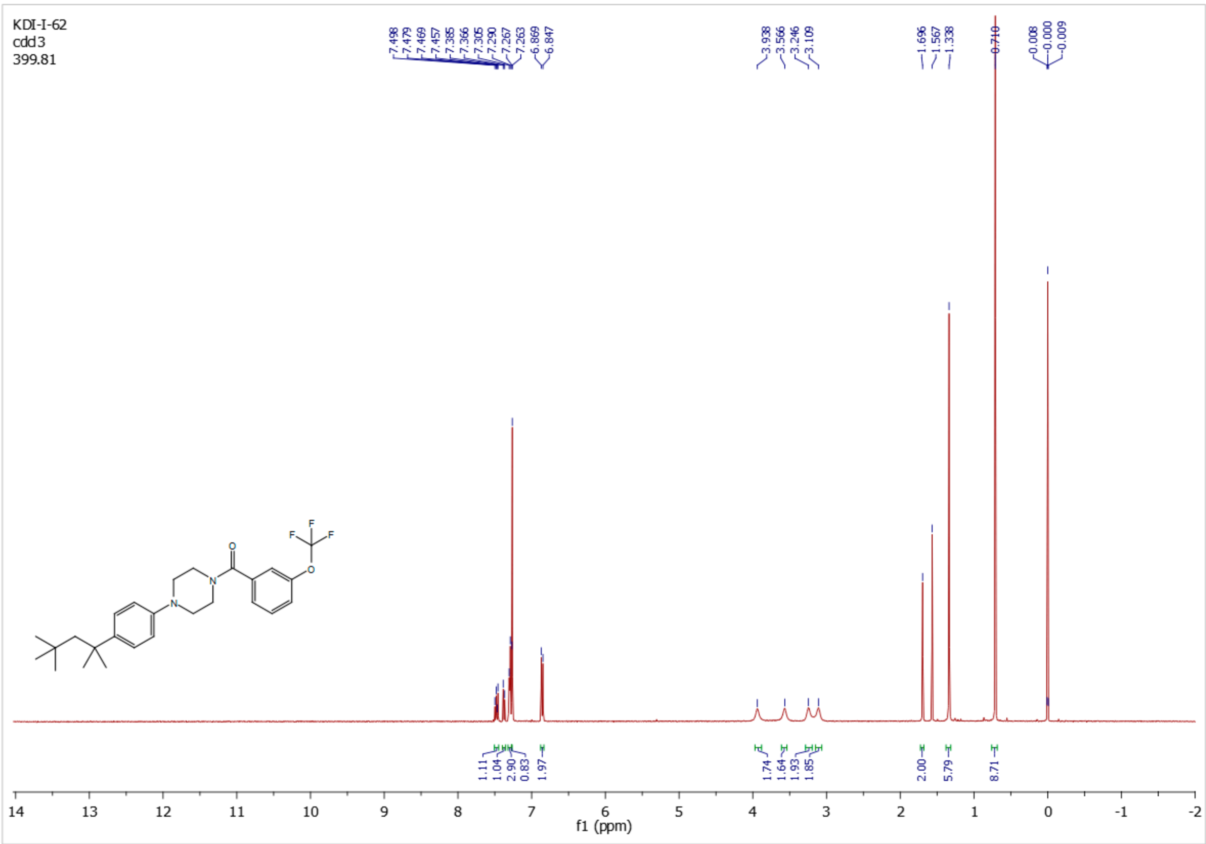

Figure S60. ^{13}C NMR (100 MHz, $CDCl_3$) spectrum of compound **51**

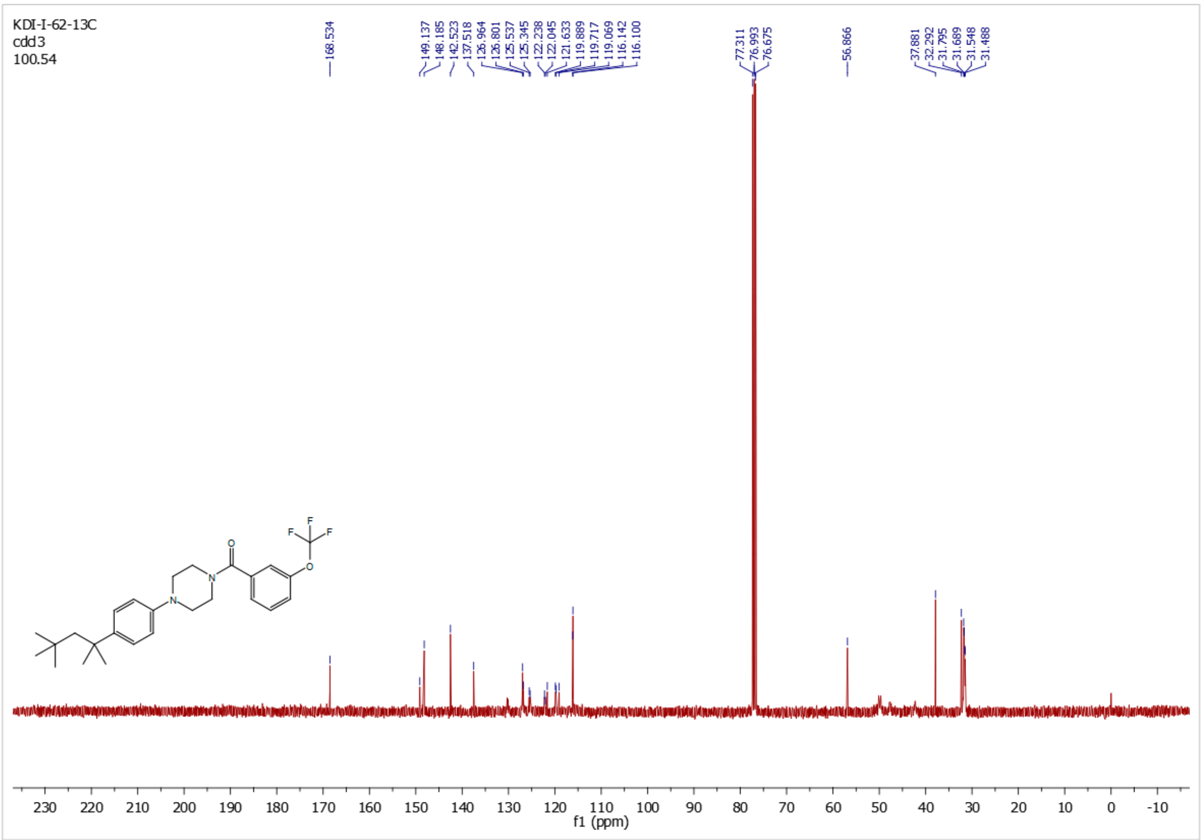

Figure S61. ^{19}F NMR (376 MHz, $CDCl_3$) spectrum of compound **51**

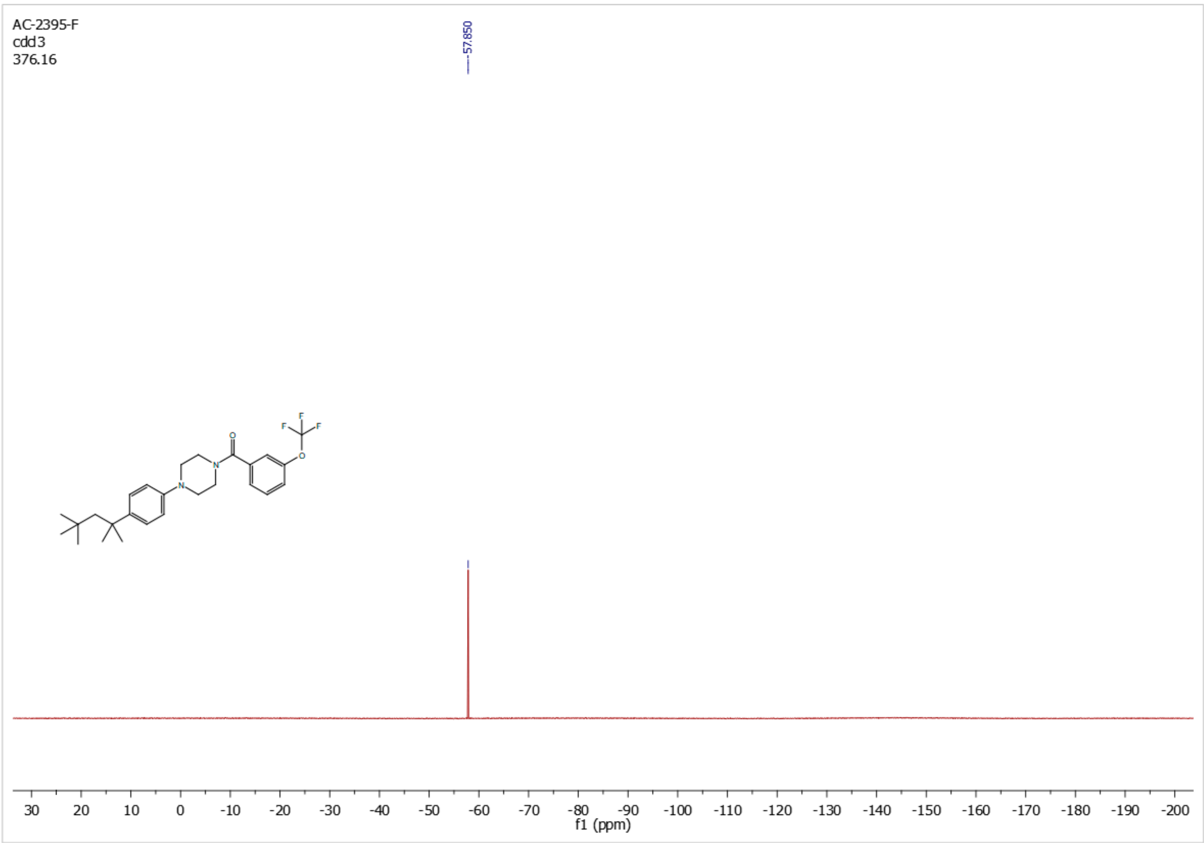

Figure S62. ^{1}H NMR (400 MHz, $CDCl_3$) spectrum of compound **56**

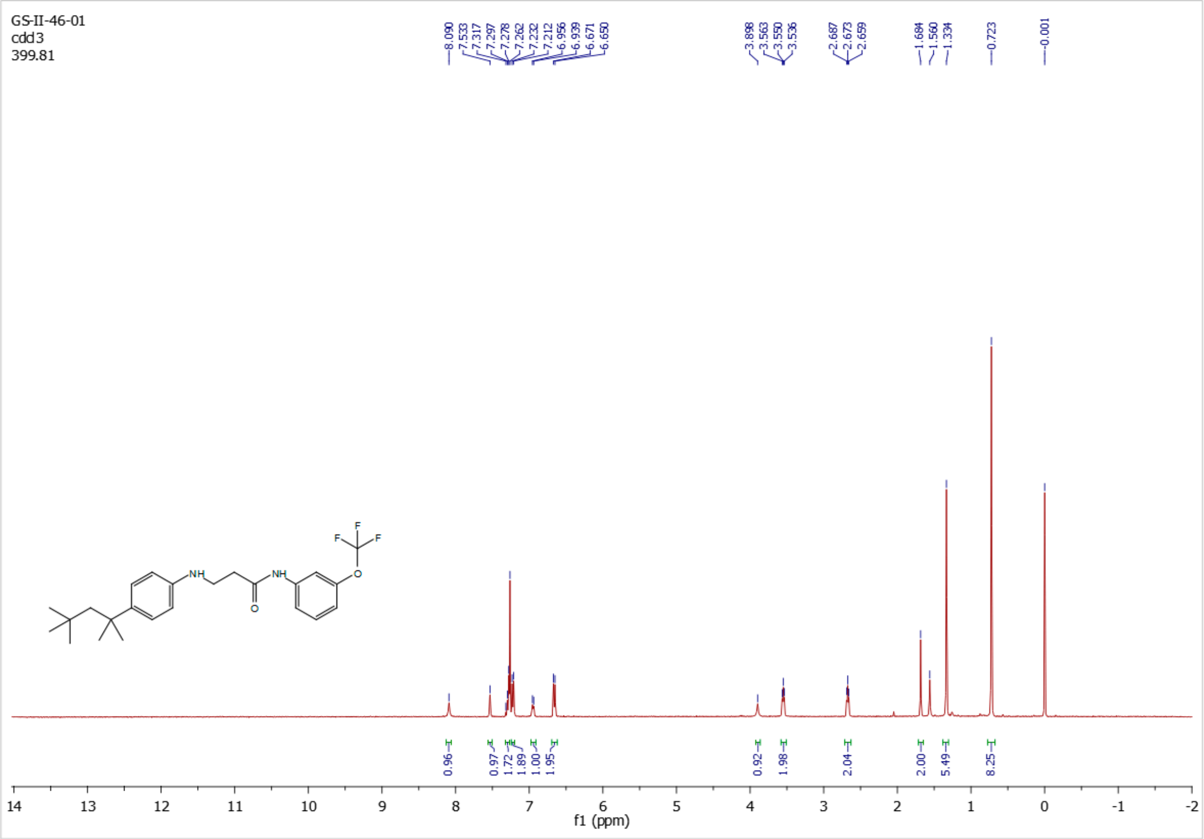

Figure S63. ^{13}C NMR (100 MHz, $CDCl_3$) spectrum of compound **56**

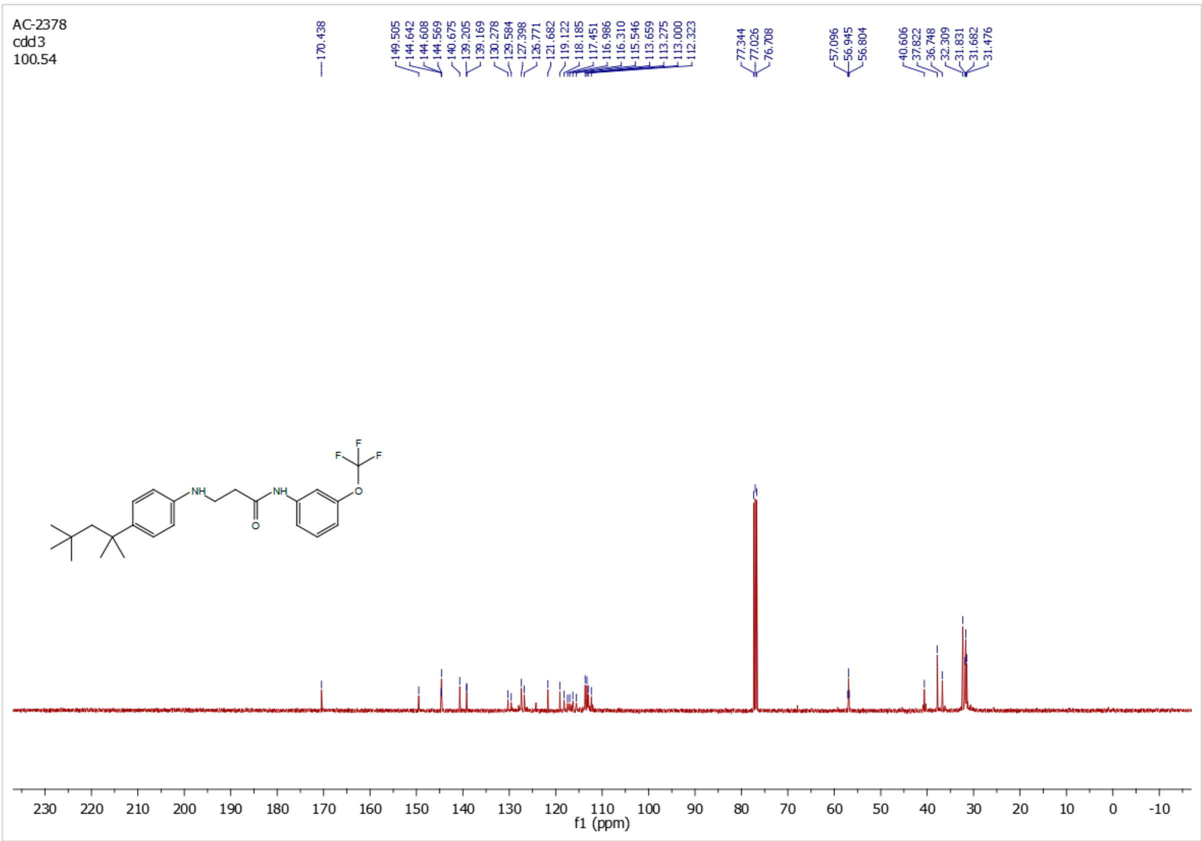

Figure S64. ^{19}F NMR (376 MHz, $CDCl_3$) spectrum of compound **56**

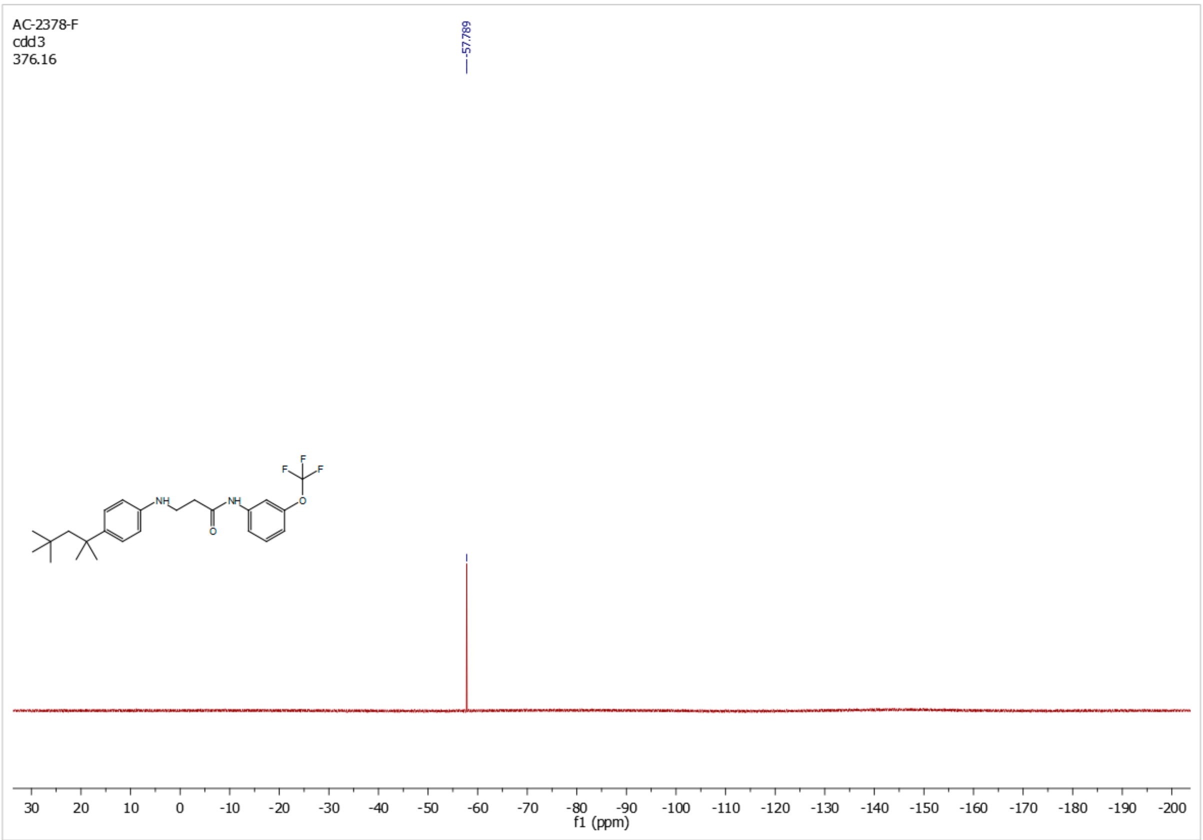

Figure S65. ^{1}H NMR (400 MHz, $CDCl_3$) spectrum of compound **57a**

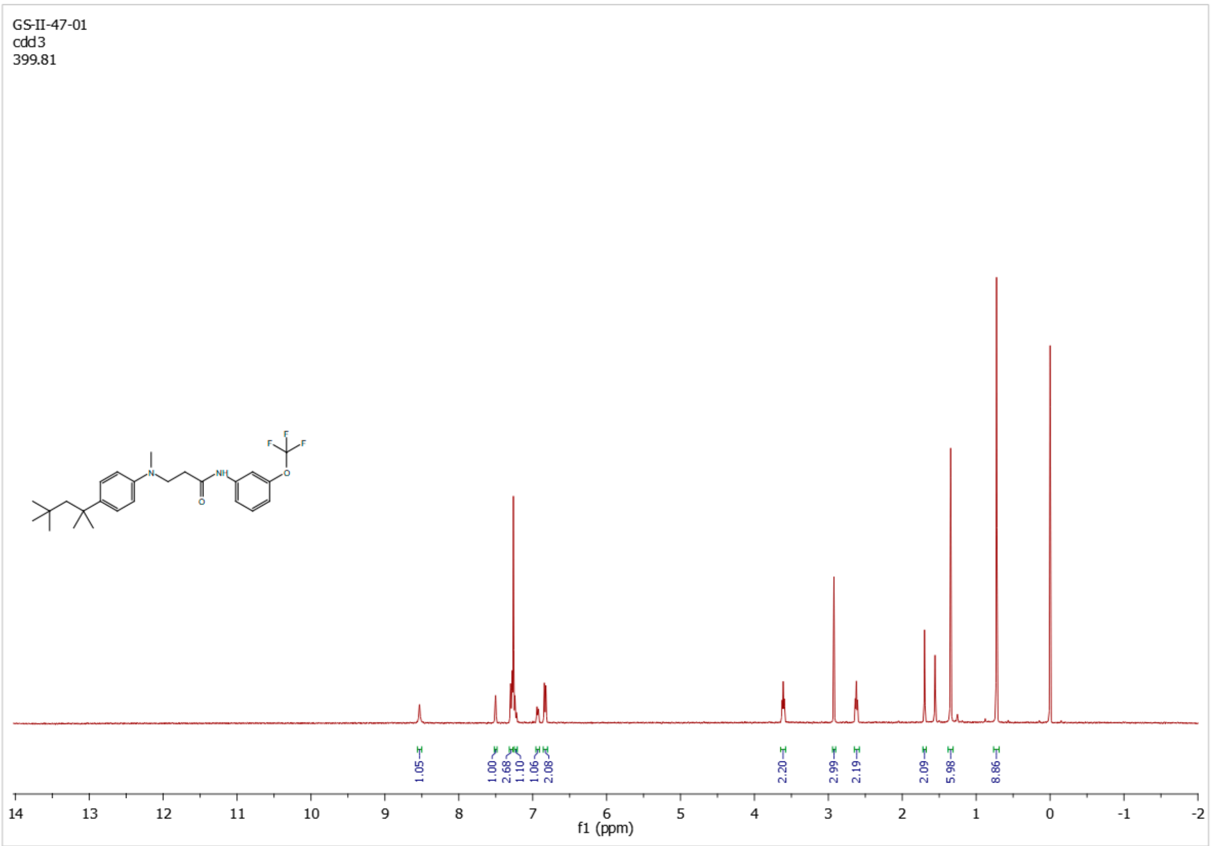

Figure S66. ^{13}C NMR (100 MHz, CD_3OD) spectrum of compound **57a**

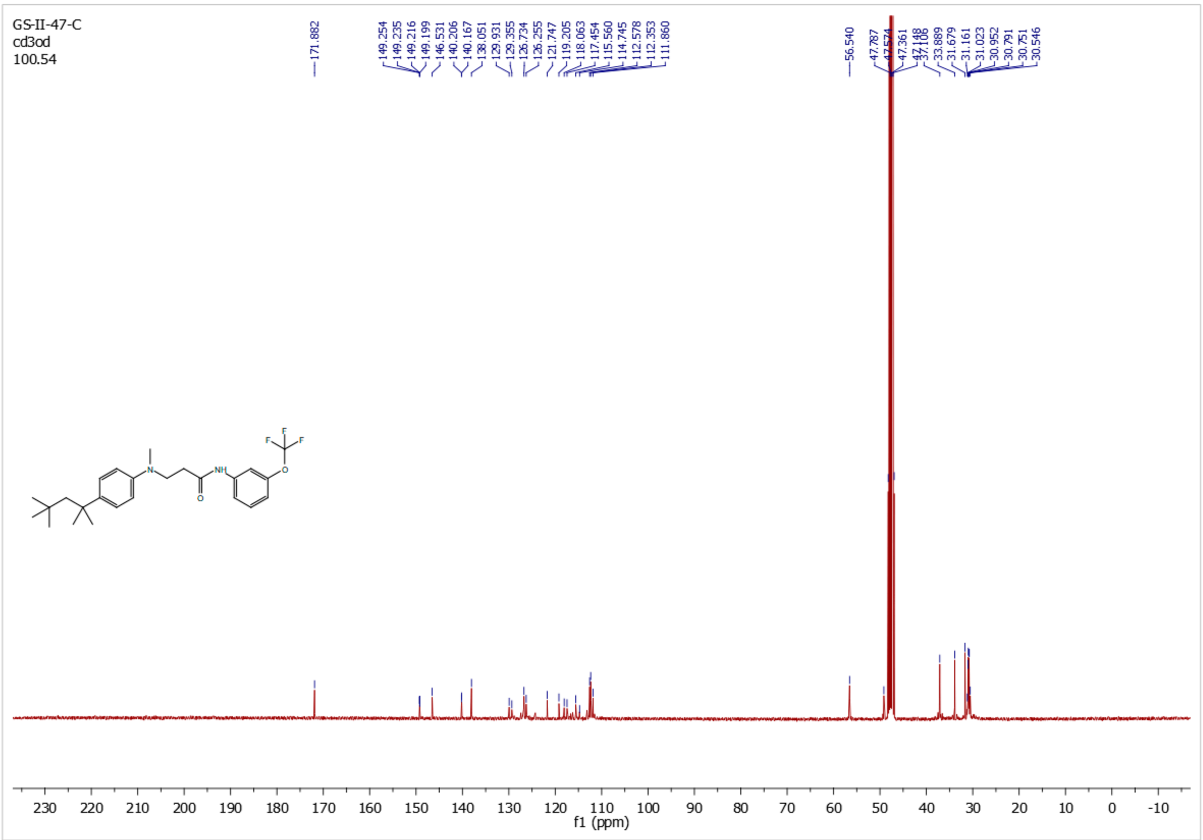

Figure S67. ^{19}F NMR (376 MHz, $CDCl_3$) spectrum of compound **57a**

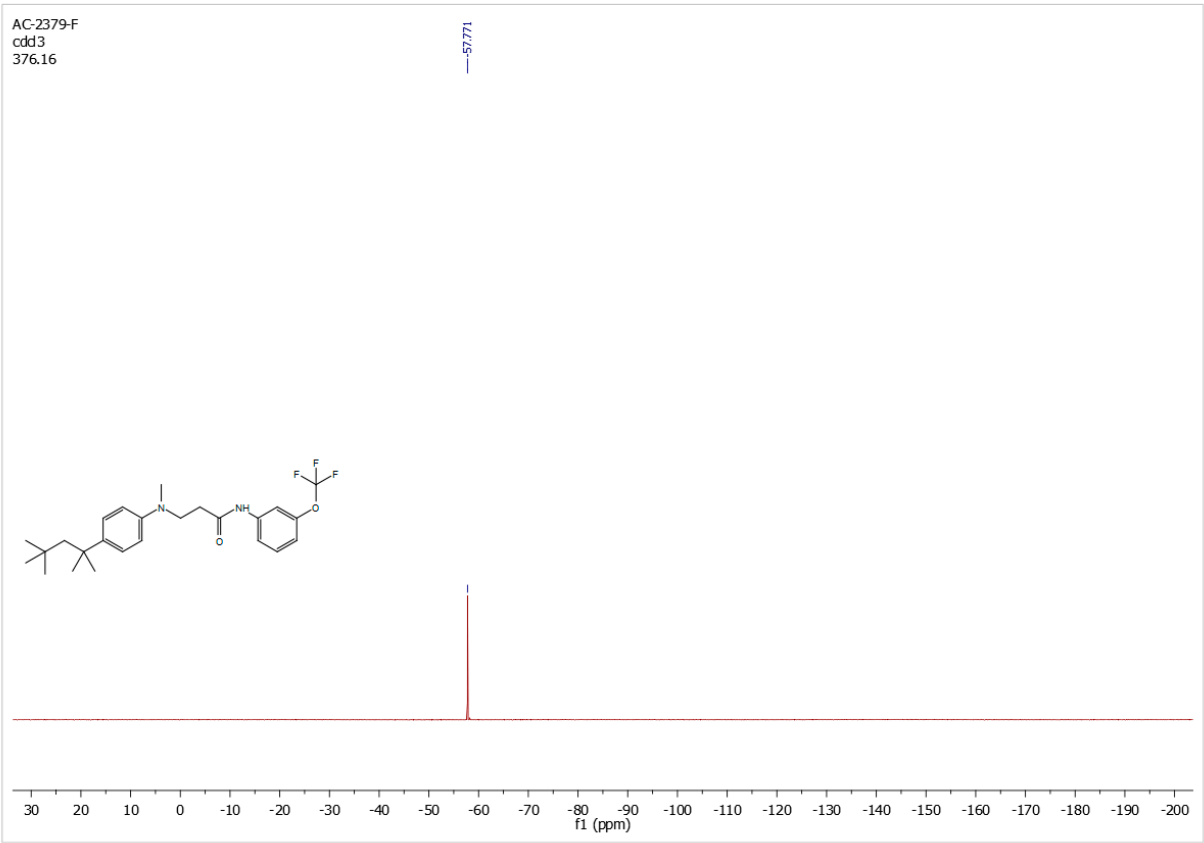

Figure S68. ^{1}H NMR (400 MHz, $CDCl_3$) spectrum of compound **57b**

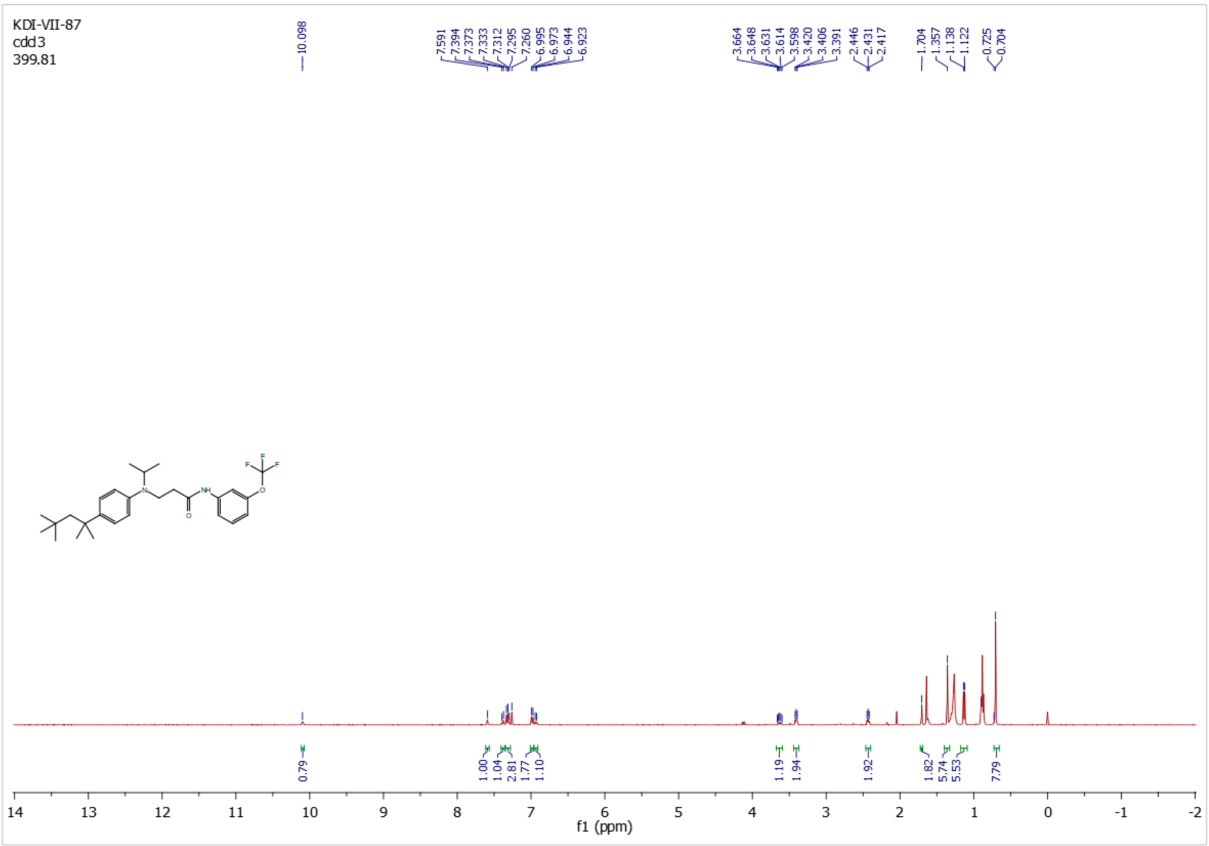

Figure S69. ^{13}C NMR (100 MHz, $CDCl_3$) spectrum of compound **57b**

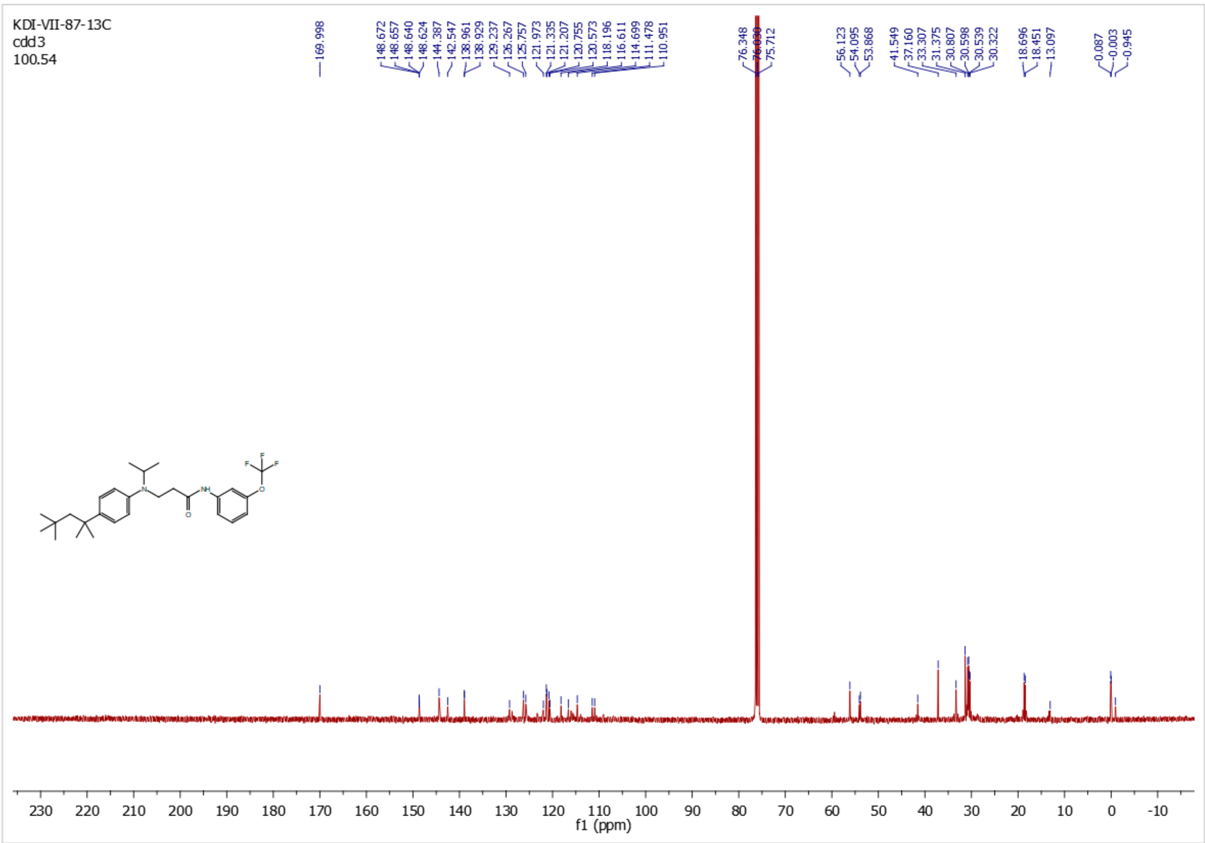

Figure S70. ^{19}F NMR (376 MHz, $CDCl_3$) spectrum of compound **57b**

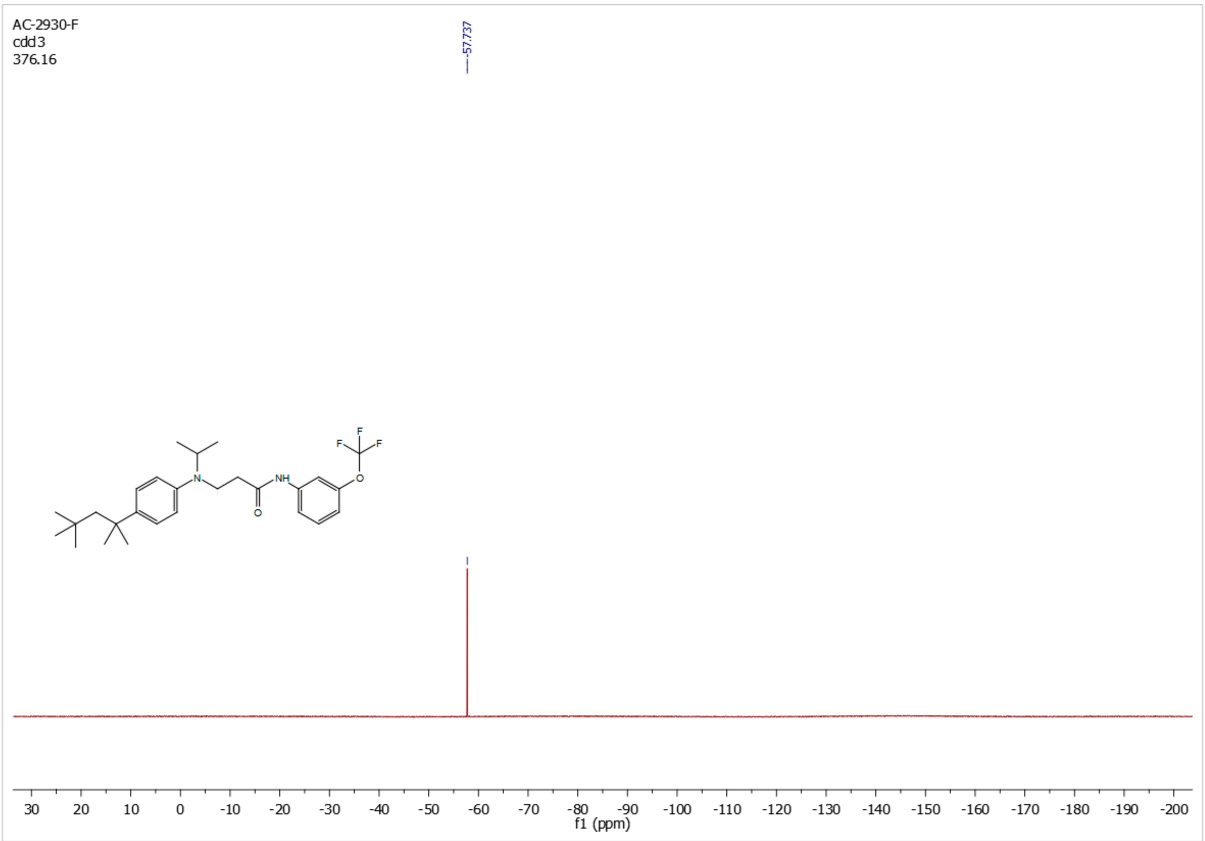

Figure S71. ^{1}H NMR (400 MHz, $CDCl_3$) spectrum of compound **57c**

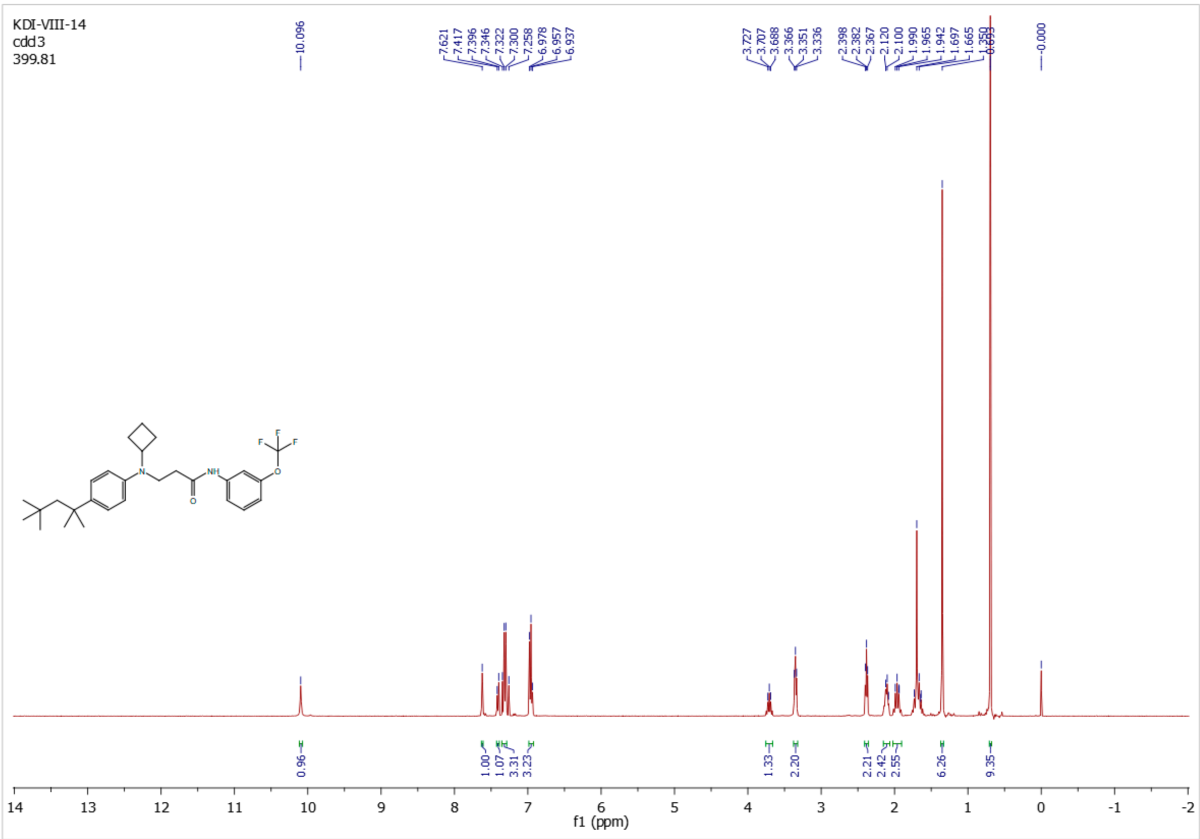

Figure S72. ^{13}C NMR (100 MHz, $CDCl_3$) spectrum of compound **57c**

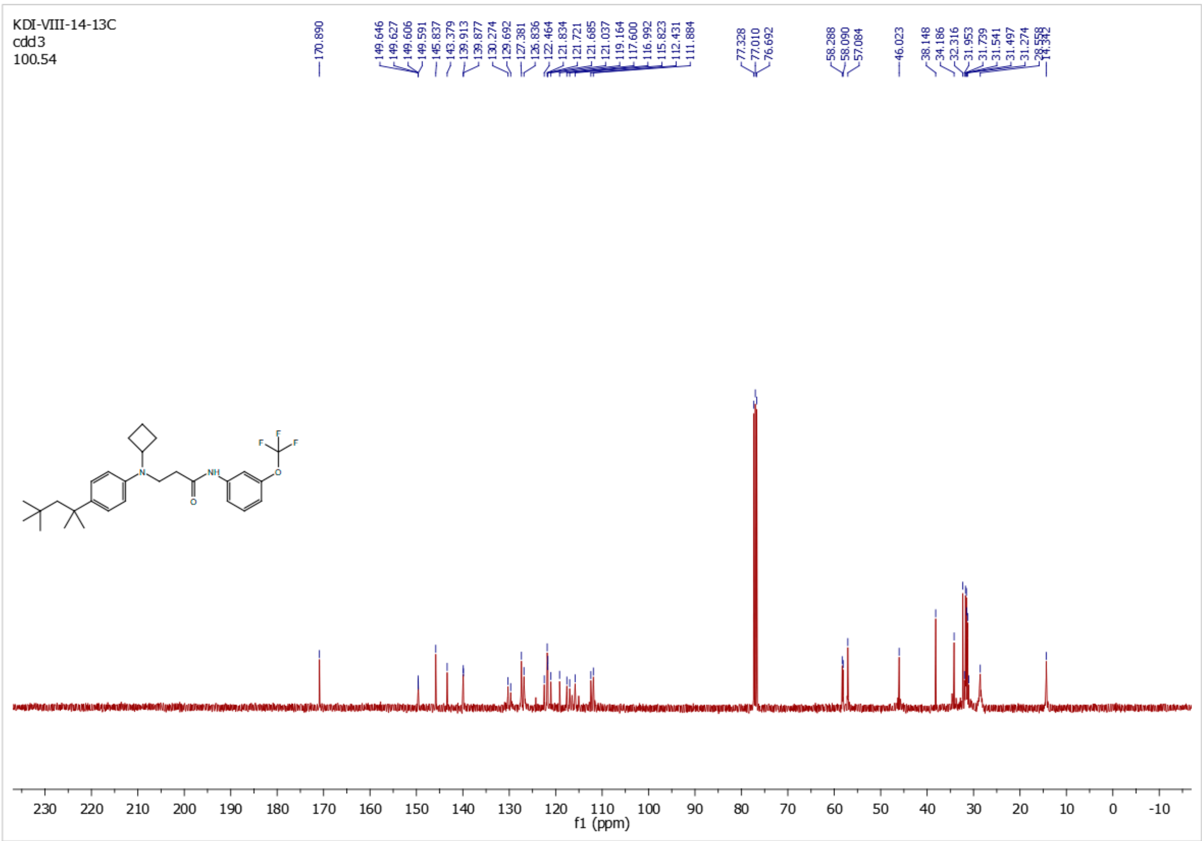

Figure S73. ^{19}F NMR (376 MHz, $CDCl_3$) spectrum of compound **57c**

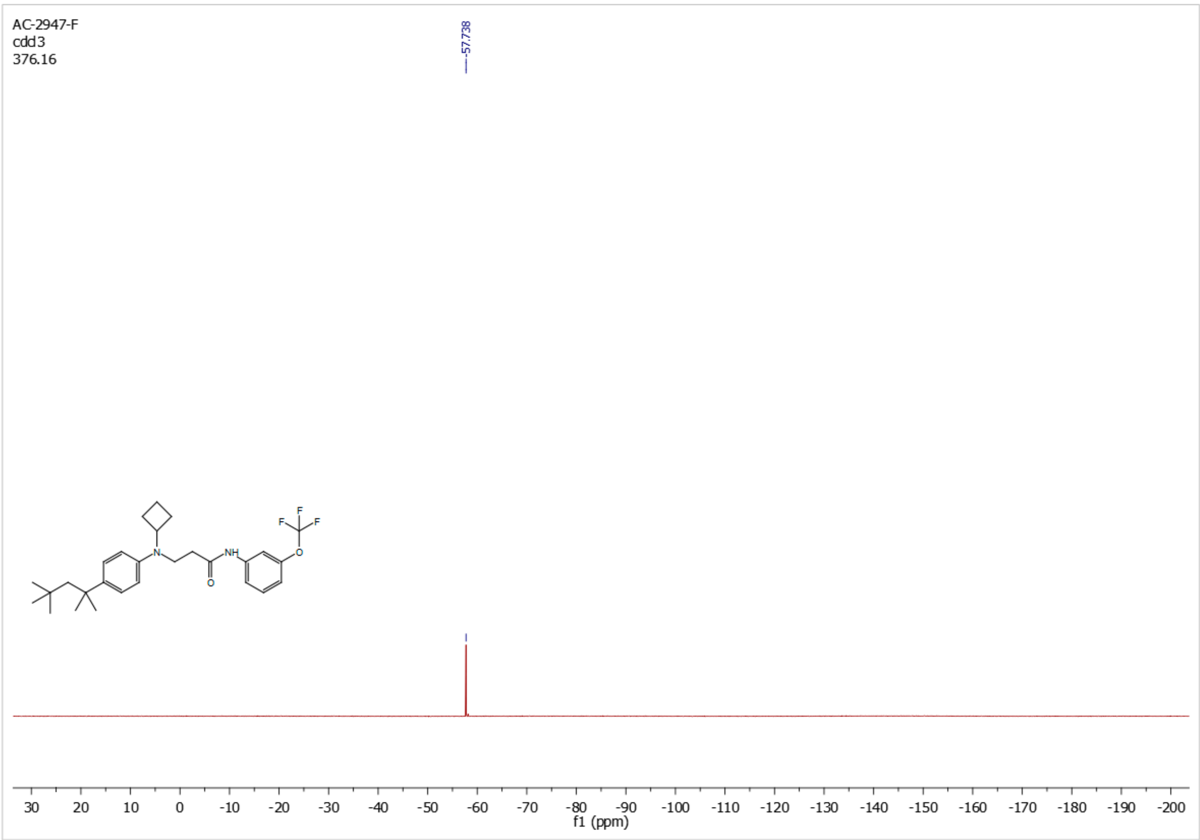

Figure S74. ^{1}H NMR (400 MHz, $CDCl_3$) spectrum of compound **57d**

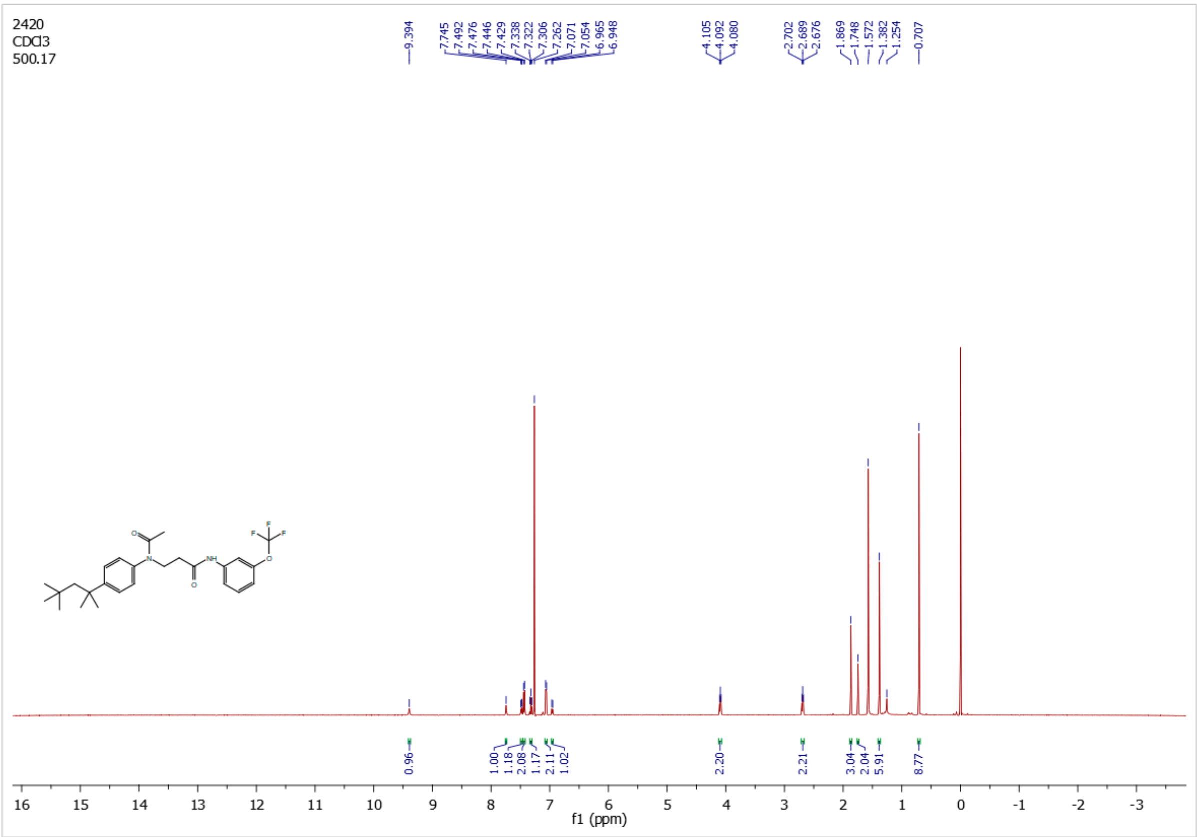

Figure S75. ^{13}C NMR (100 MHz, $CDCl_3$) spectrum of compound **57d**

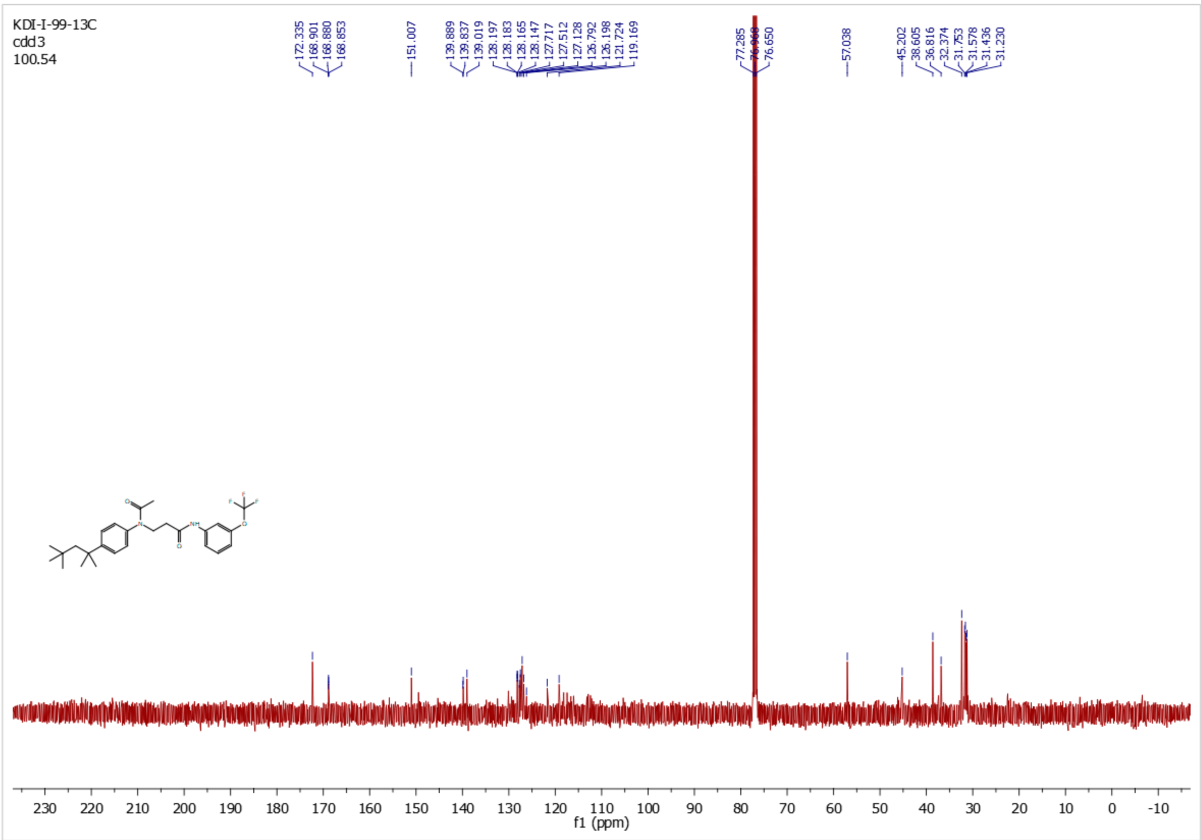

Figure S76. ^{19}F NMR (376 MHz, $CDCl_3$) spectrum of compound **57d**

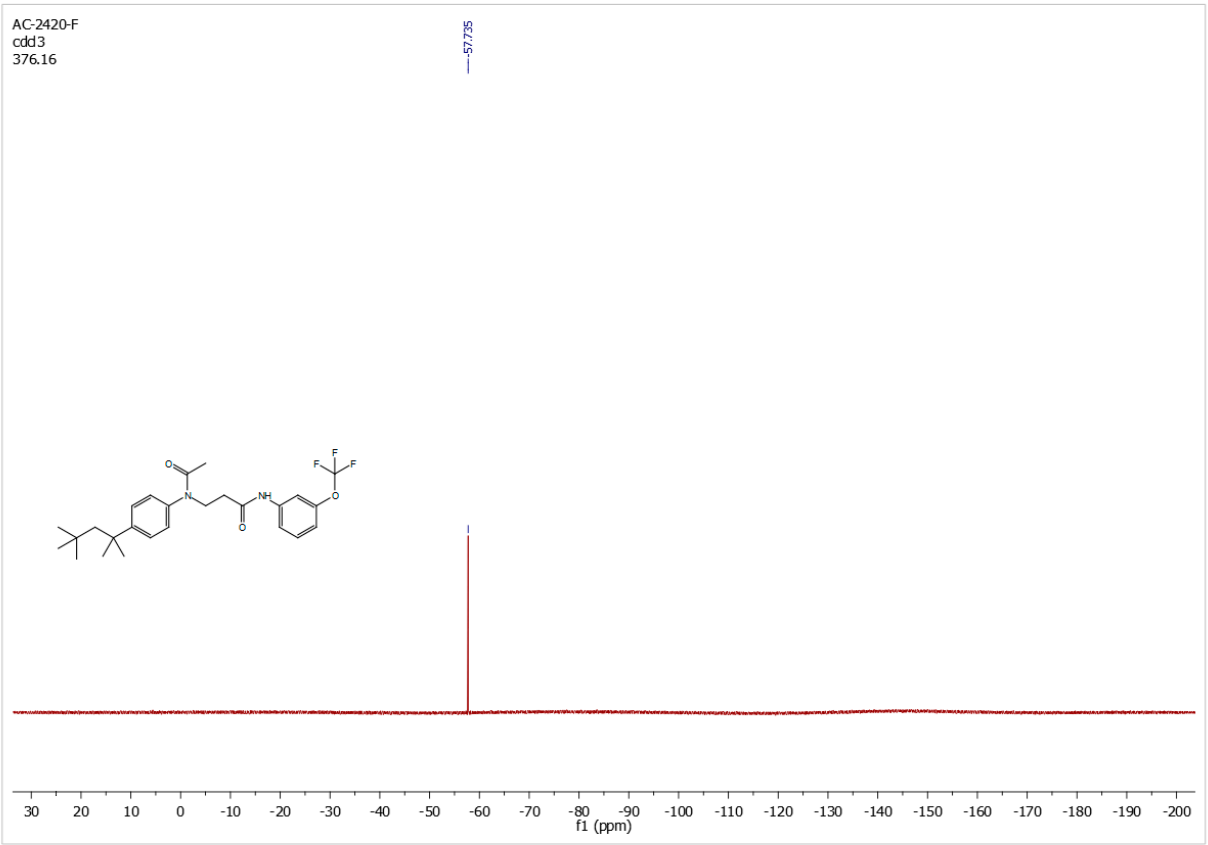

Figures S77. HPLC Analysis

Compound 5a

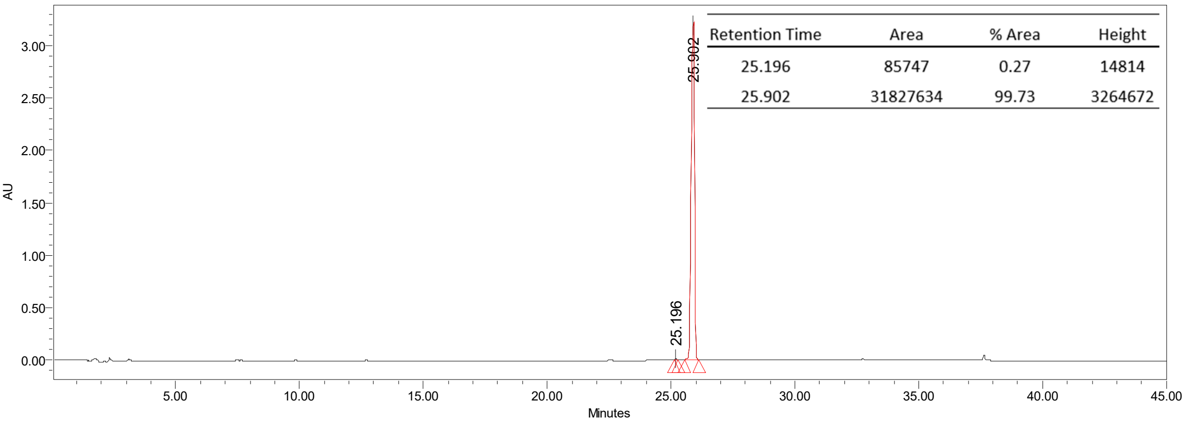

Retention Time	Area	% Area	Height
25.196	85747	0.27	14814
25.902	31827634	99.73	3264672

Compound 5b

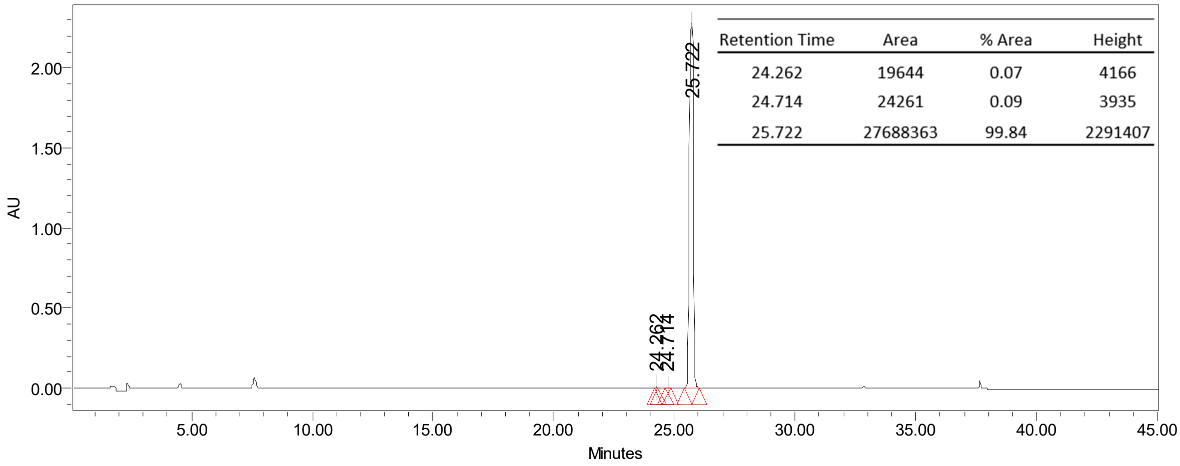

Retention Time	Area	% Area	Height
24.262	19644	0.07	4166
24.714	24261	0.09	3935
25.722	27688363	99.84	2291407

Compound 5c

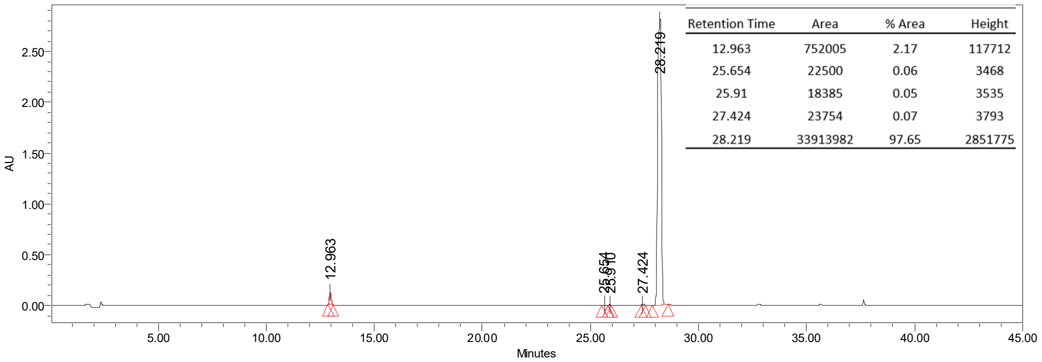

Retention Time	Area	% Area	Height
12.963	752005	2.17	117712
25.654	22500	0.06	3468
25.91	18385	0.05	3535
27.424	23754	0.07	3793
28.219	33913982	97.65	2851775

Figures S78. HPLC Analysis

Compound 5d

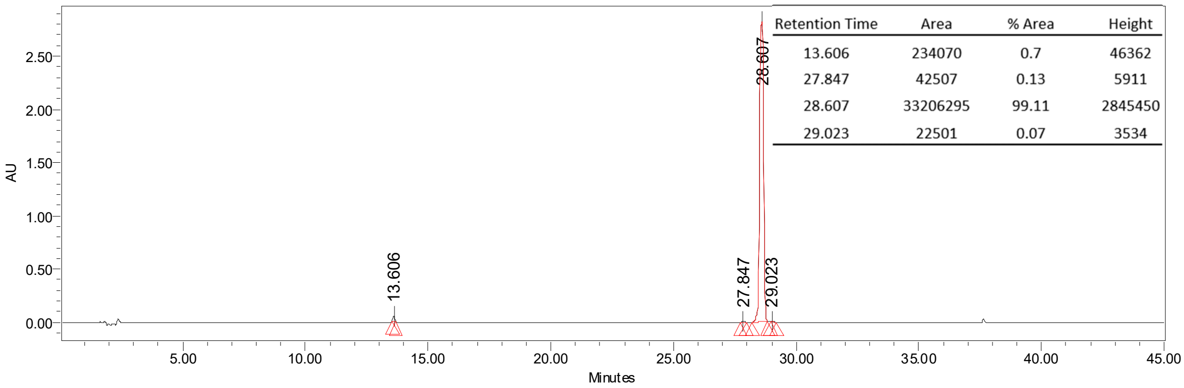

Compound 5e

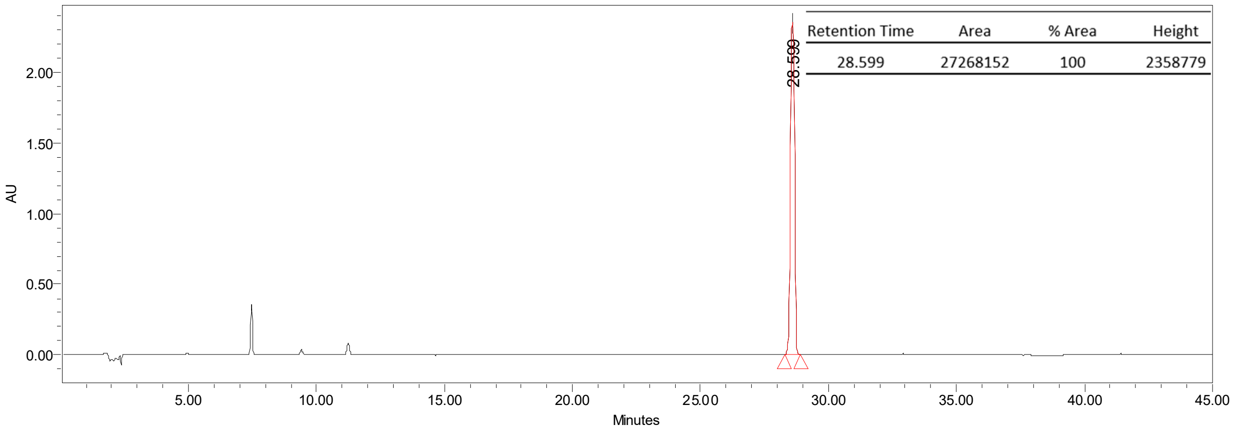

Compound 5f

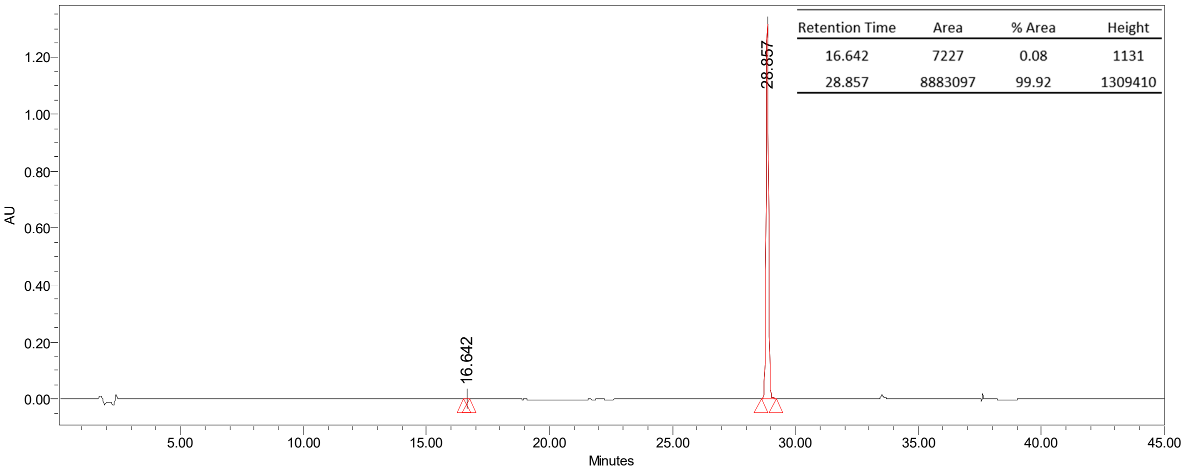

Figures S79. HPLC Analysis

Compound 14

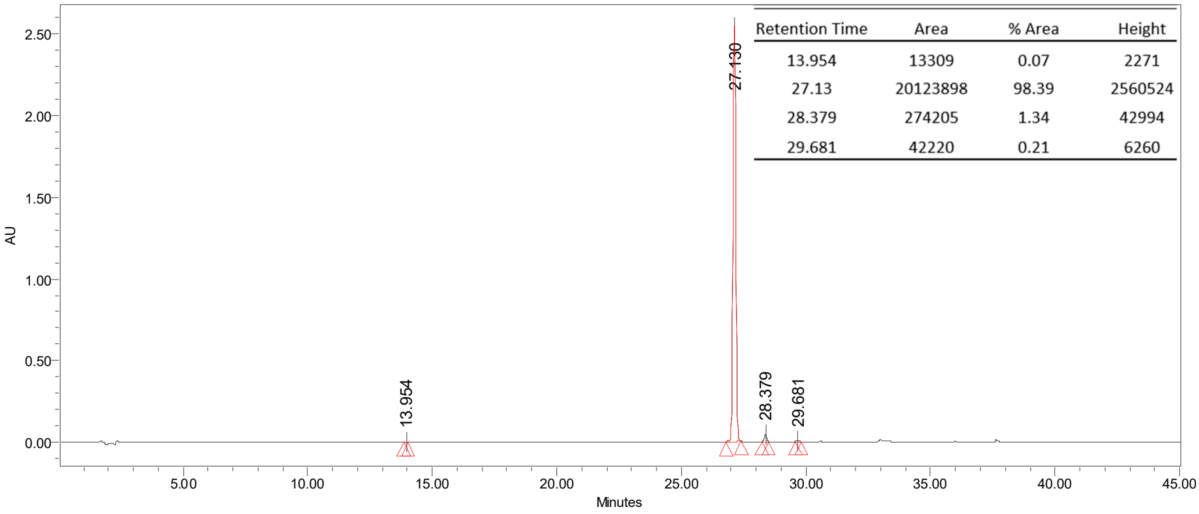

Retention Time	Area	% Area	Height
13.954	13309	0.07	2271
27.13	20123898	98.39	2560524
28.379	274205	1.34	42994
29.681	42220	0.21	6260

Compound 21

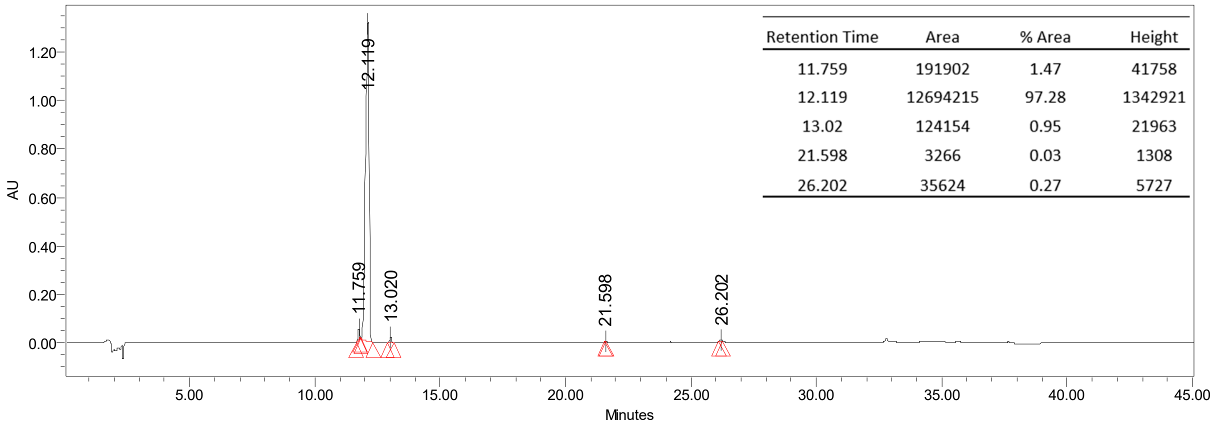

Retention Time	Area	% Area	Height
11.759	191902	1.47	41758
12.119	12694215	97.28	1342921
13.02	124154	0.95	21963
21.598	3266	0.03	1308
26.202	35624	0.27	5727

Compound 22

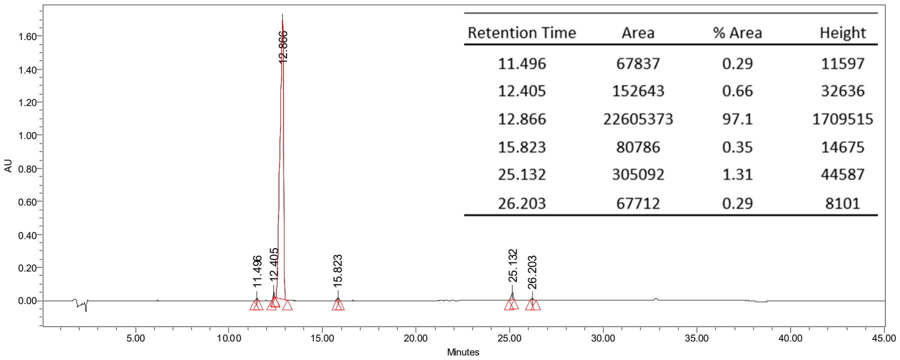

Retention Time	Area	% Area	Height
11.496	67837	0.29	11597
12.405	152643	0.66	32636
12.866	22605373	97.1	1709515
15.823	80786	0.35	14675
25.132	305092	1.31	44587
26.203	67712	0.29	8101

Figures S80. HPLC Analysis

Compound 30

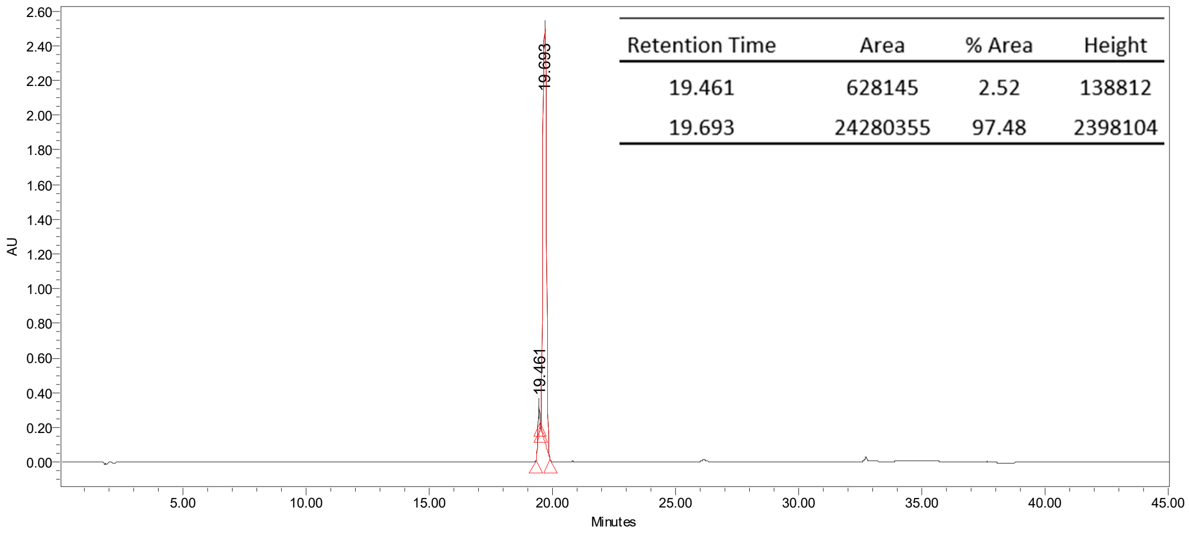

Retention Time	Area	% Area	Height
19.461	628145	2.52	138812
19.693	24280355	97.48	2398104

Compound 35a

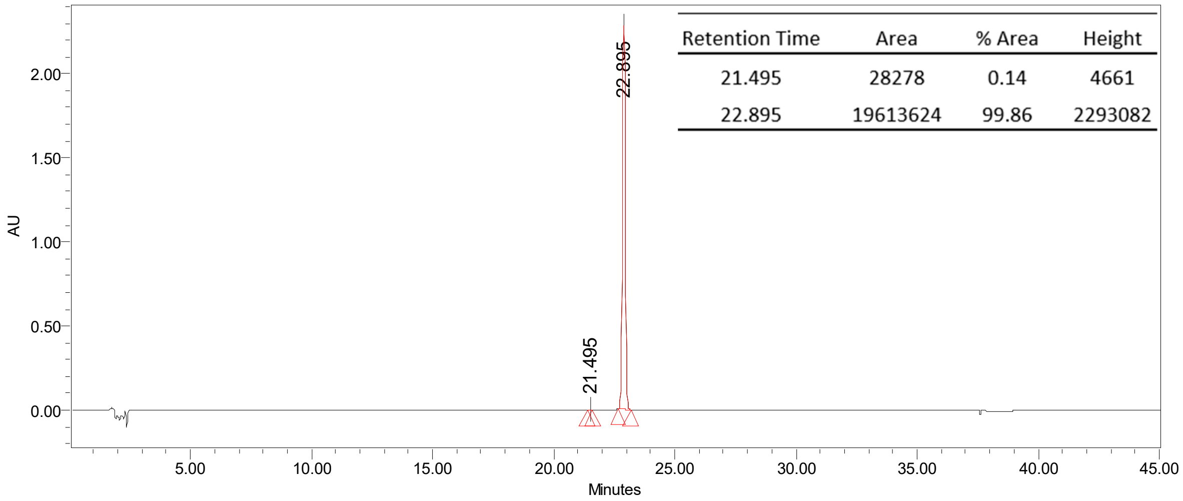

Retention Time	Area	% Area	Height
21.495	28278	0.14	4661
22.895	19613624	99.86	2293082

Figures S81. HPLC Analysis

Compound 35b

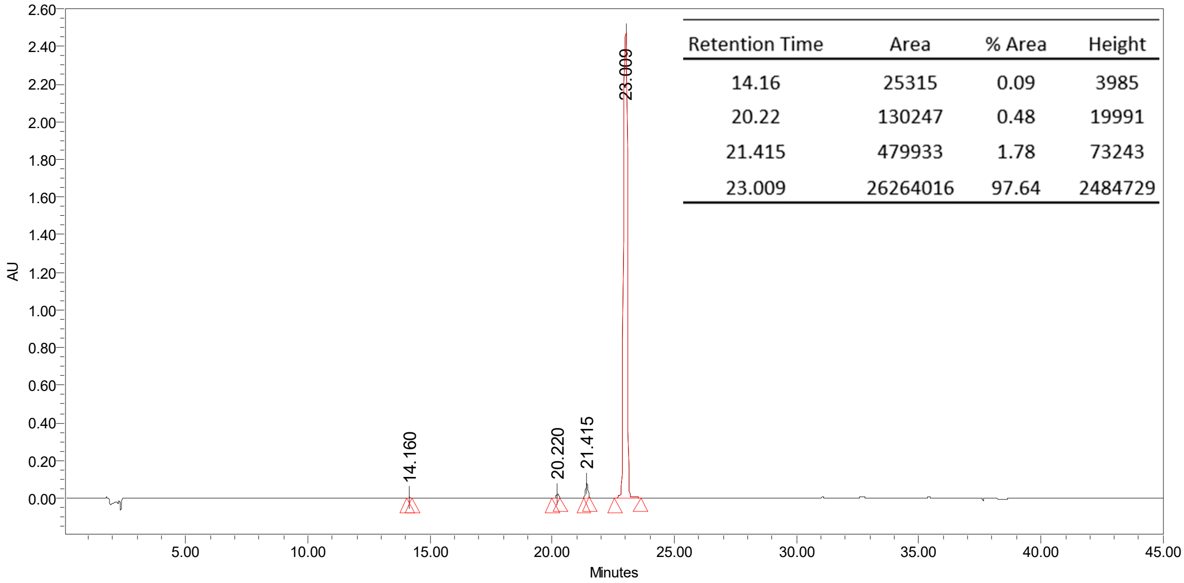

Retention Time	Area	% Area	Height
14.16	25315	0.09	3985
20.22	130247	0.48	19991
21.415	479933	1.78	73243
23.009	26264016	97.64	2484729

Compound 39a

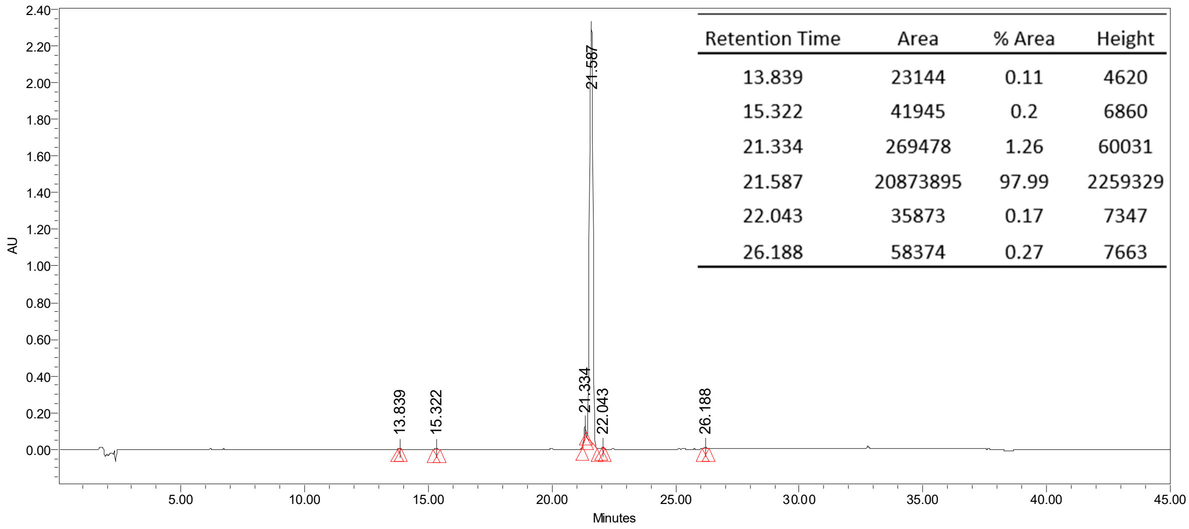

Retention Time	Area	% Area	Height
13.839	23144	0.11	4620
15.322	41945	0.2	6860
21.334	269478	1.26	60031
21.587	20873895	97.99	2259329
22.043	35873	0.17	7347
26.188	58374	0.27	7663

Figures S82. HPLC Analysis

Compound 39b

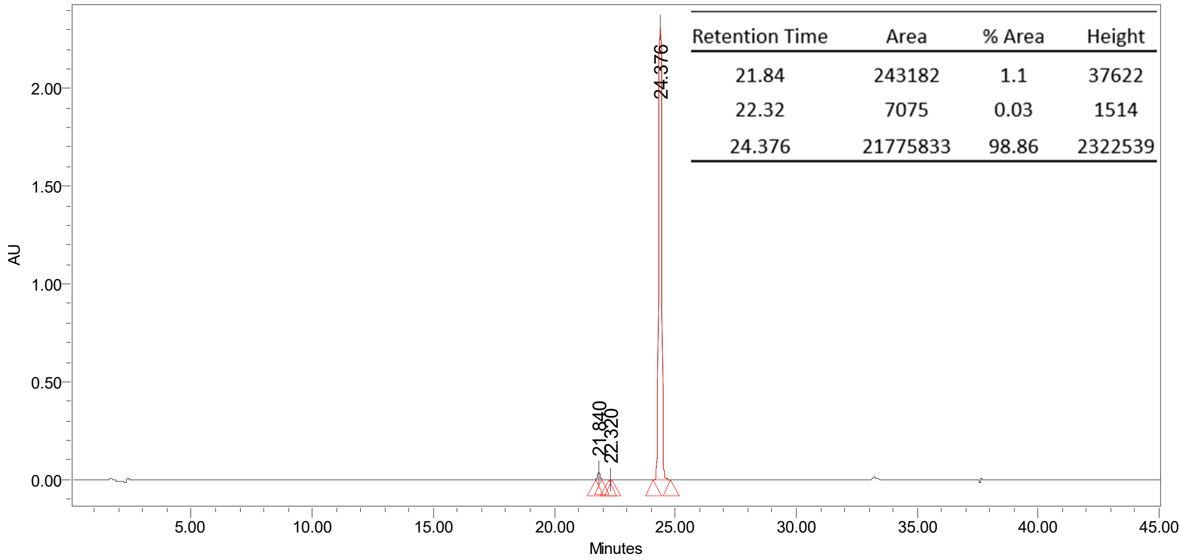

Retention Time	Area	% Area	Height
21.84	243182	1.1	37622
22.32	7075	0.03	1514
24.376	21775833	98.86	2322539

Compound 39c

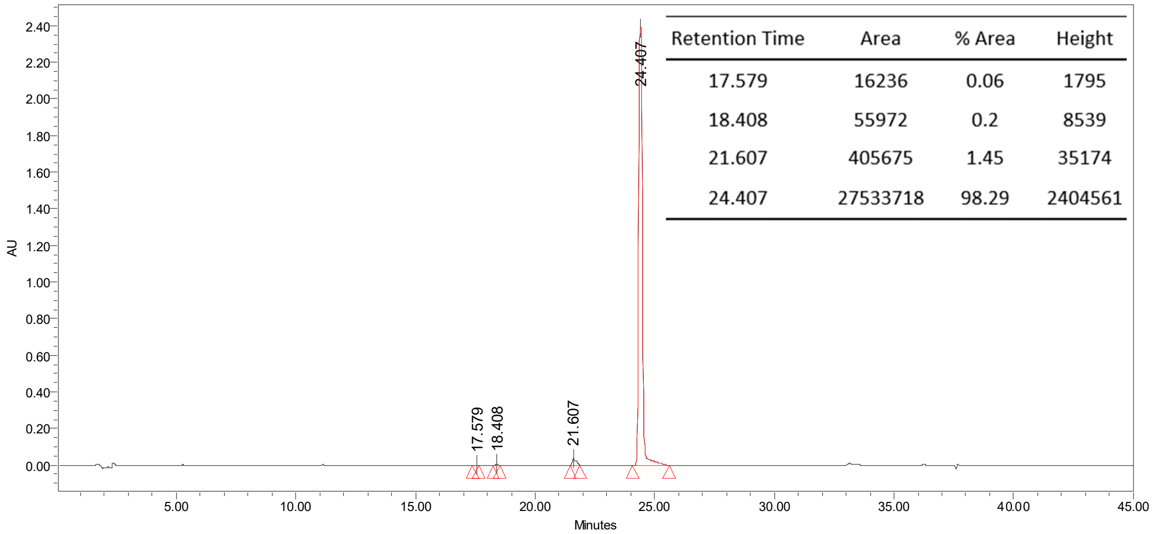

Retention Time	Area	% Area	Height
17.579	16236	0.06	1795
18.408	55972	0.2	8539
21.607	405675	1.45	35174
24.407	27533718	98.29	2404561

Figures S83. HPLC Analysis

Compound 39d

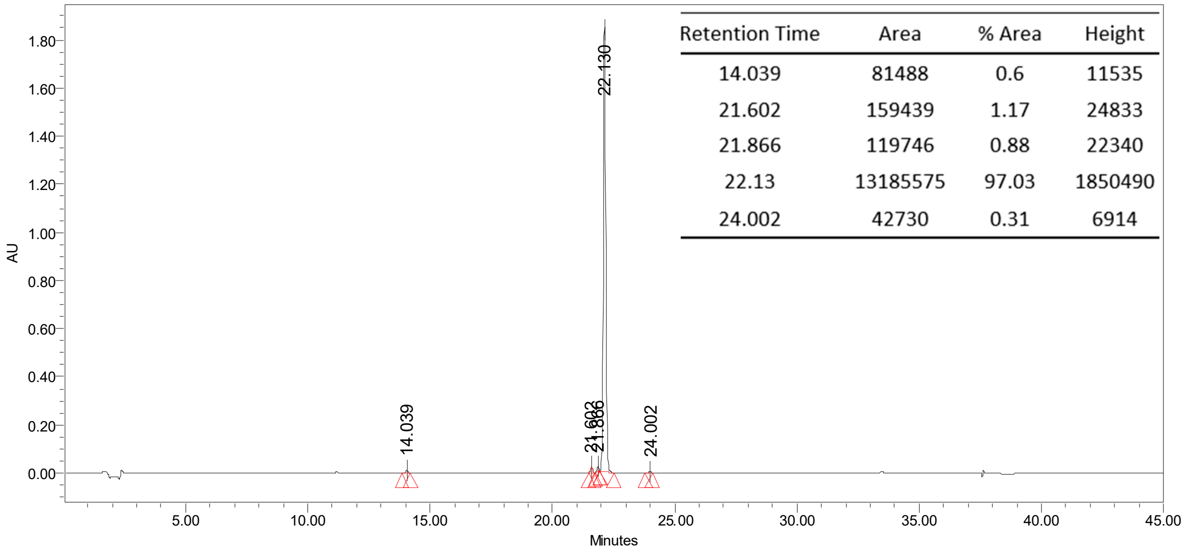

Retention Time	Area	% Area	Height
14.039	81488	0.6	11535
21.602	159439	1.17	24833
21.866	119746	0.88	22340
22.13	13185575	97.03	1850490
24.002	42730	0.31	6914

Compound 44a

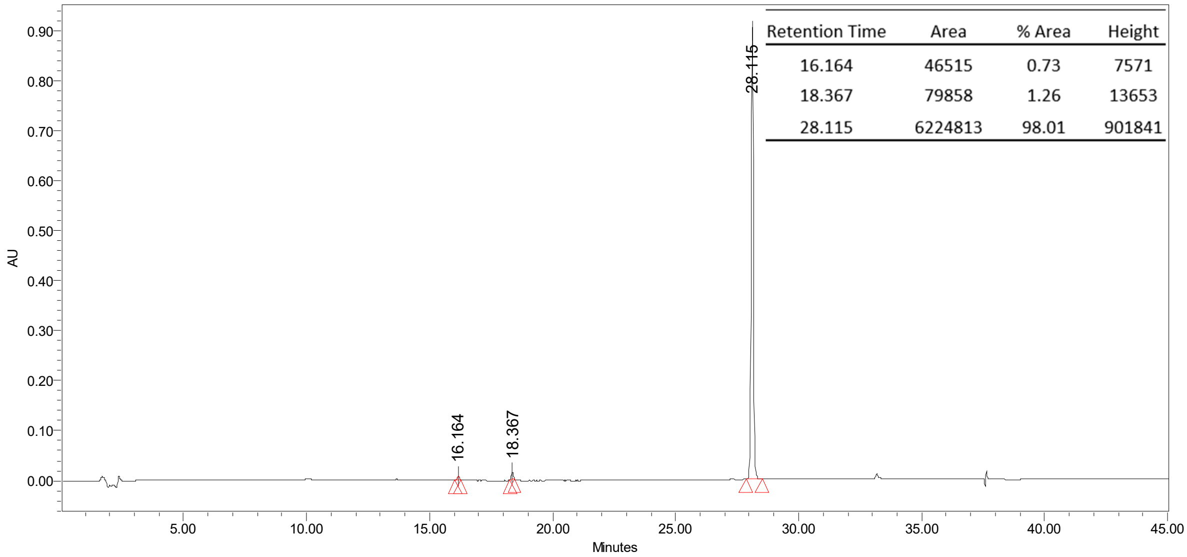

Retention Time	Area	% Area	Height
16.164	46515	0.73	7571
18.367	79858	1.26	13653
28.115	6224813	98.01	901841

Figures S84. HPLC Analysis

Compound 44b

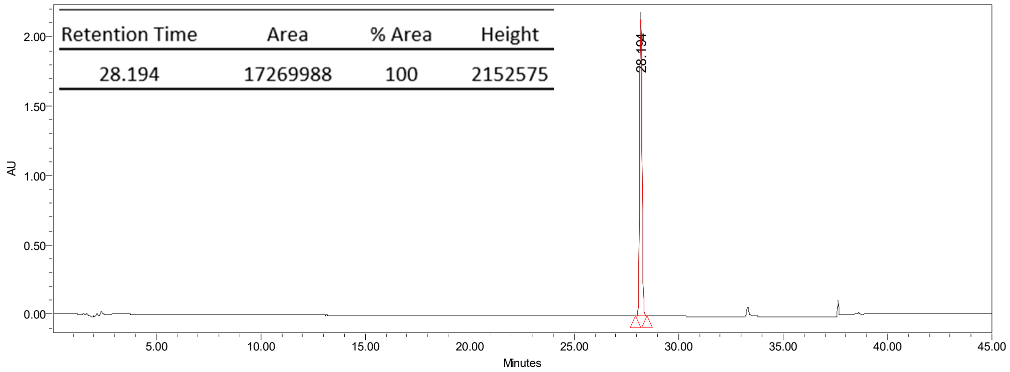

Retention Time	Area	% Area	Height
28.194	17269988	100	2152575

Compound 50

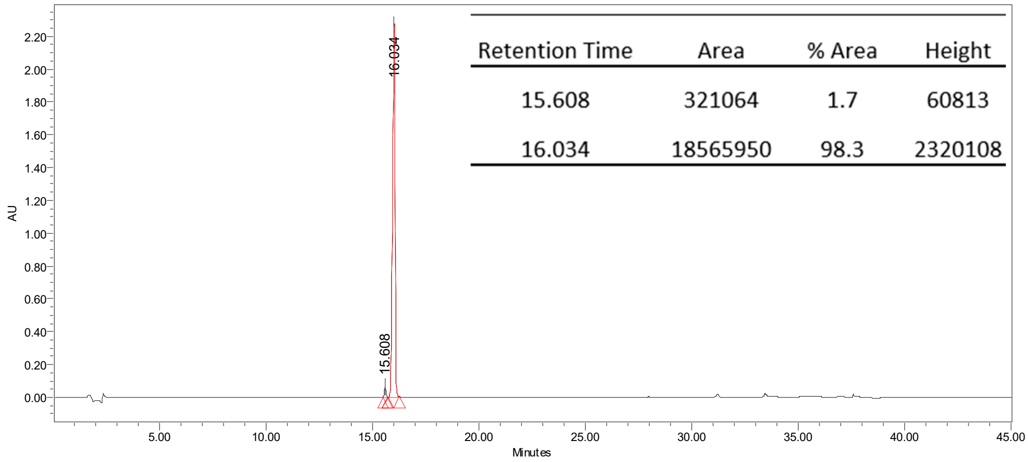

Retention Time	Area	% Area	Height
15.608	321064	1.7	60813
16.034	18565950	98.3	2320108

Figures S85. HPLC Analysis

Compound 51

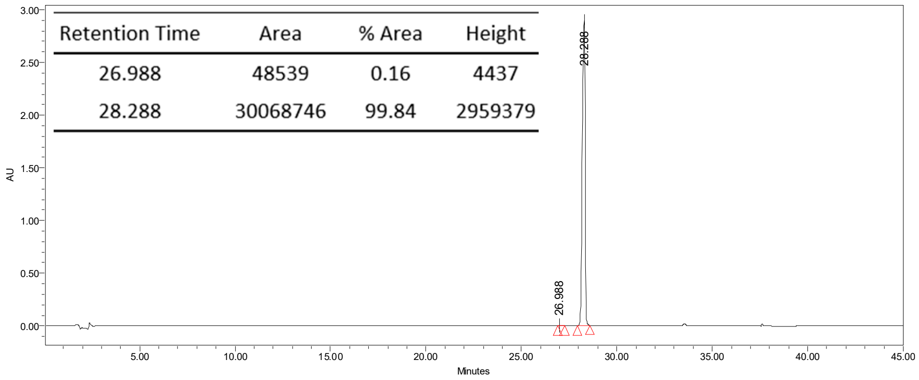

Retention Time	Area	% Area	Height
26.988	48539	0.16	4437
28.288	30068746	99.84	2959379

Figures S85. HPLC Analysis

Compound 56

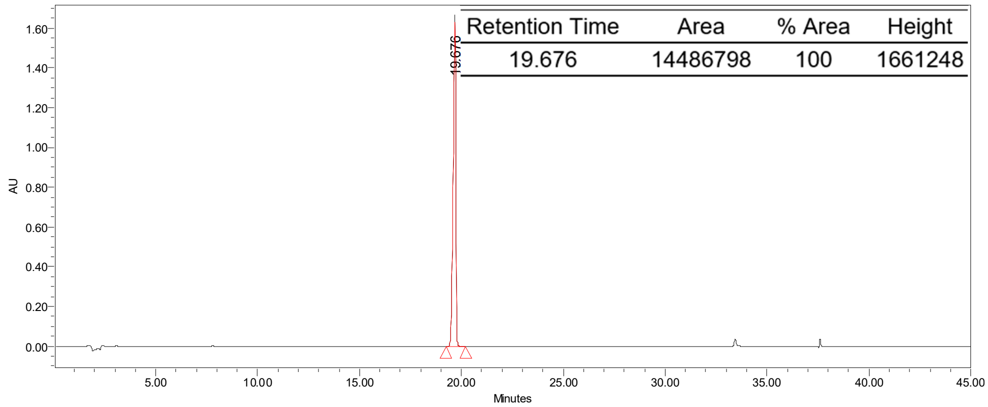

Retention Time	Area	% Area	Height
19.676	14486798	100	1661248

Compound 57a

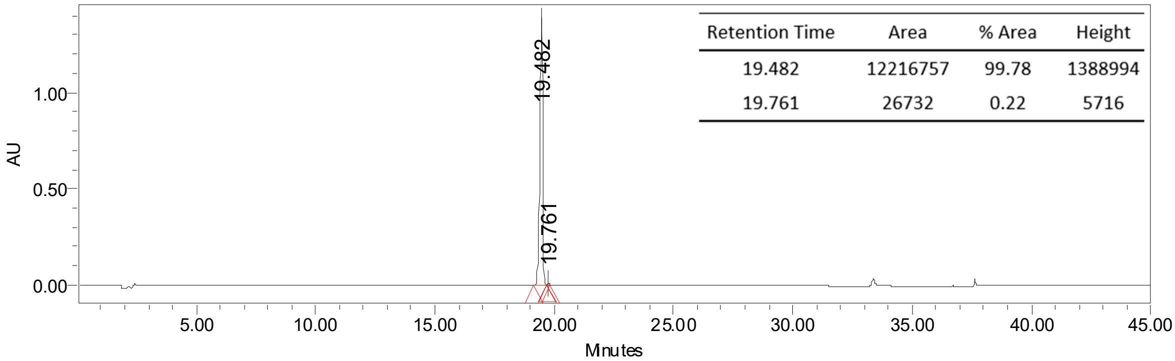

Retention Time	Area	% Area	Height
19.482	12216757	99.78	1388994
19.761	26732	0.22	5716

Compound 57b

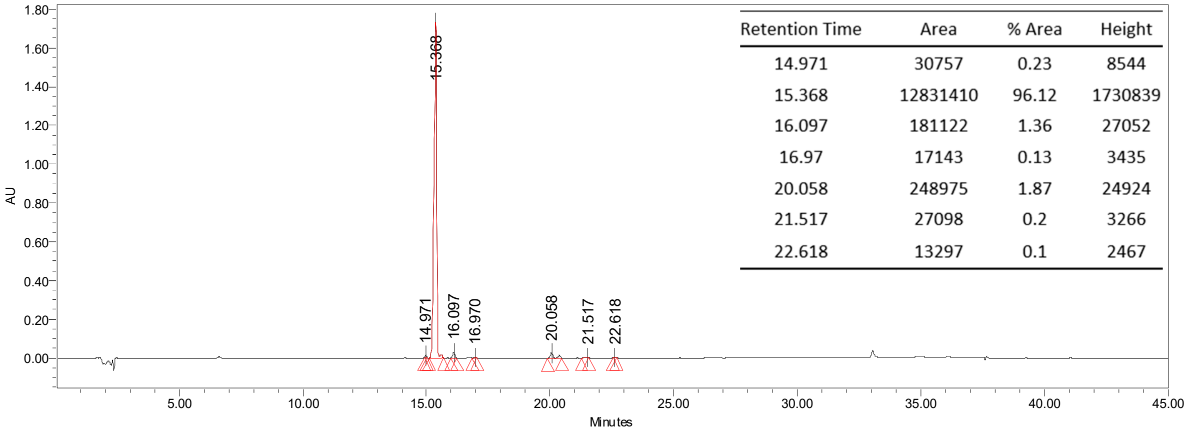

Retention Time	Area	% Area	Height
14.971	30757	0.23	8544
15.368	12831410	96.12	1730839
16.097	181122	1.36	27052
16.97	17143	0.13	3435
20.058	248975	1.87	24924
21.517	27098	0.2	3266
22.618	13297	0.1	2467

Figures S86. HPLC Analysis

Compound 57c

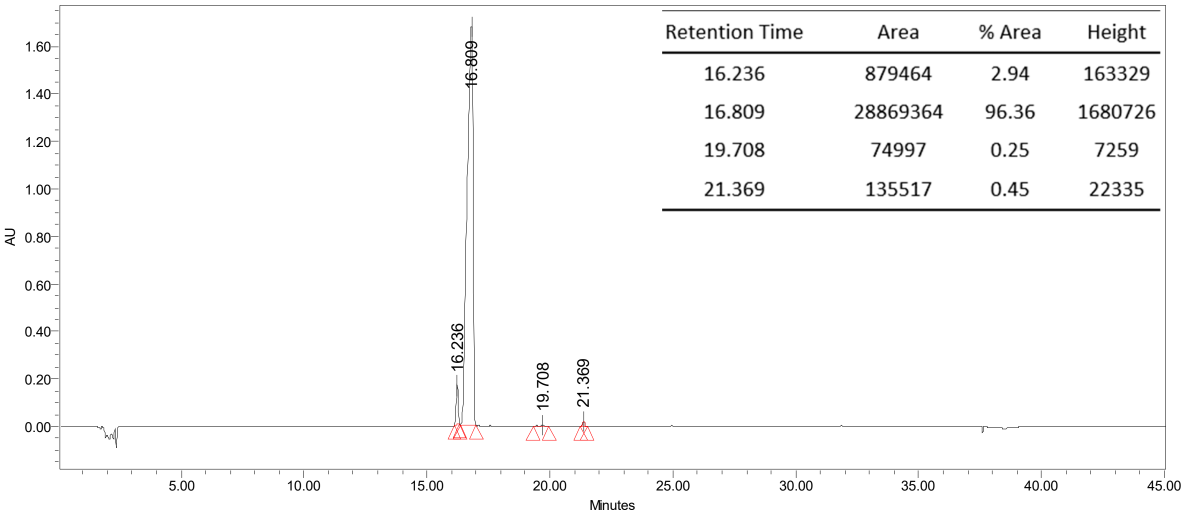

Retention Time	Area	% Area	Height
16.236	879464	2.94	163329
16.809	28869364	96.36	1680726
19.708	74997	0.25	7259
21.369	135517	0.45	22335

Compound 57d

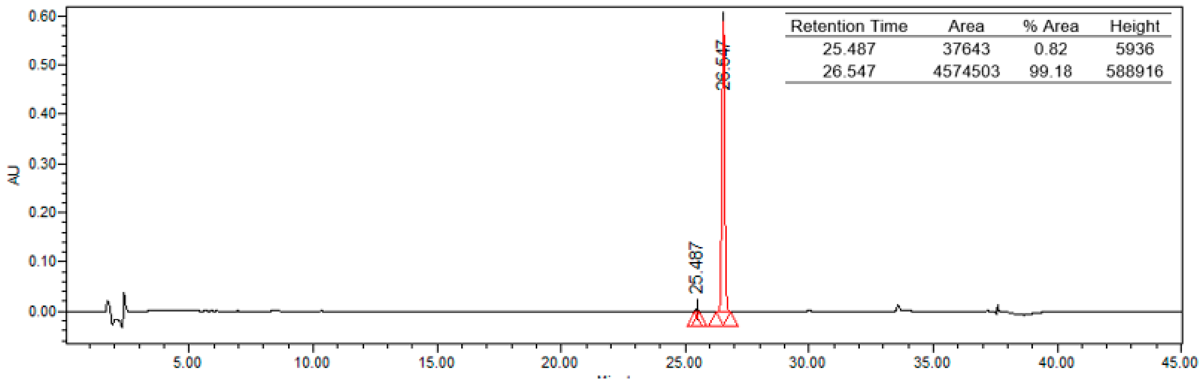

Retention Time	Area	% Area	Height
25.487	37643	0.82	5936
26.547	4574503	99.18	588916

Figures S87. HRMS Analysis

Compound 5a

Single Mass Analysis
Tolerance = 5.0 mDa / DBE: min = -1.5, max = 50.0
Element prediction: Off
Number of isotope peaks used for i-FIT = 3

Monoisotopic Mass, Even Electron Ions
703 formula(e) evaluated with 4 results within limits (all results (up to 1000) for each mass)
Elements Used:
C: 0-500 H: 0-1000 N: 0-10 O: 0-20
GS-I-109-01 130 (2.294)
1: TOF MS ES+

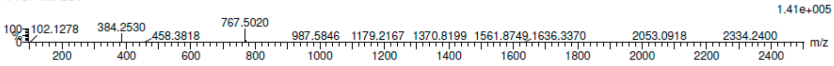

Minimum:				-1.5				
Maximum:		5.0	10.0	50.0				
Mass	Calc. Mass	mDa	PPM	DBE	i-FIT	Norm	Conf(%)	Formula
384.2530	384.2539	-0.9	-2.3	8.5	117.3	3.626	2.66	C24 H34 N O3

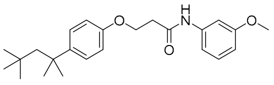

Chemical Formula: $C_{24}H_{33}NO_3$
Exact Mass: 383.2460

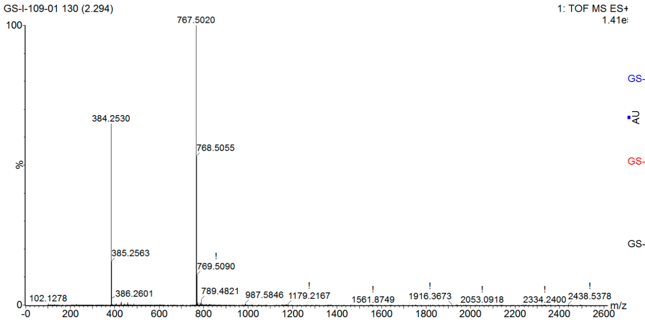

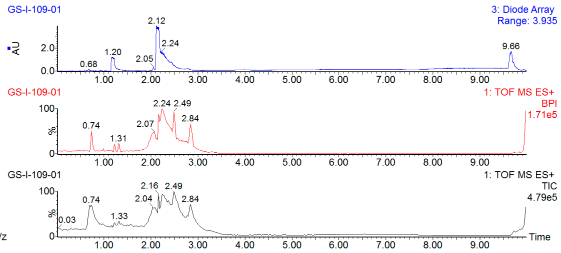

Compound 5b

Single Mass Analysis
Tolerance = 5.0 mDa / DBE: min = -1.5, max = 50.0
Element prediction: Off
Number of isotope peaks used for i-FIT = 3

Monoisotopic Mass, Even Electron Ions
705 formula(e) evaluated with 7 results within limits (all results (up to 1000) for each mass)
Elements Used:
C: 0-500 H: 0-1000 N: 0-10 O: 0-20
GS-I-106-01 124 (2.192)
1: TOF MS ES+

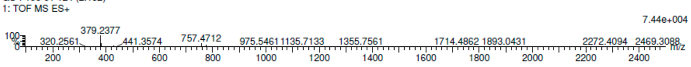

Minimum:				-1.5				
Maximum:		5.0	10.0	50.0				
Mass	Calc. Mass	mDa	PPM	DBE	i-FIT	Norm	Conf(%)	Formula
379.2377	379.2386	-0.9	-2.4	10.5	138.0	7.968	0.03	C24 H31 N2 O2

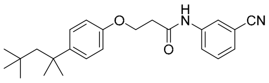

Chemical Formula: $C_{24}H_{30}N_2O_2$
Exact Mass: 378.2307

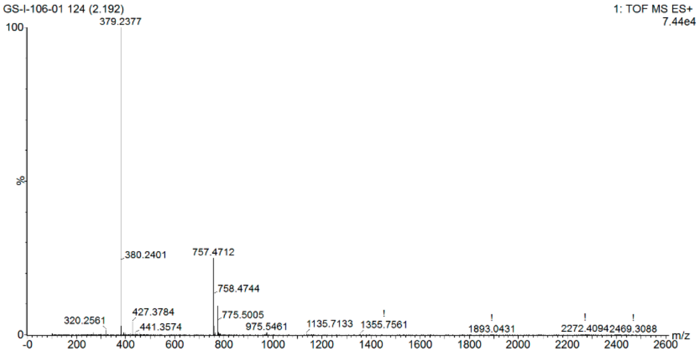

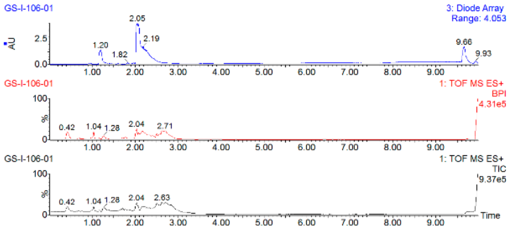

Figures S88. HRMS Analysis

Compound 5c

Single Mass Analysis
Tolerance = 5.0 mDa / DBE: min = -1.5, max = 50.0
Element prediction: Off
Number of isotope peaks used for i-FIT = 3

Monoisotopic Mass, Even Electron Ions
5337 formula(e) evaluated with 31 results within limits (all results (up to 1000) for each mass)
Elements Used:
C: 0-500 H: 0-1000 N: 0-10 O: 0-20 F: 0-10

GS-I-110-01 148 (2.611)
1: TOF MS ES+

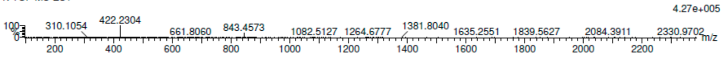

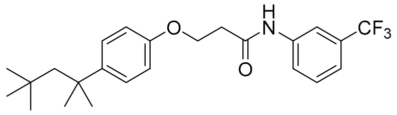

Chemical Formula: $C_{24}H_{30}F_3NO_2$
Exact Mass: 421.2229

Minimum: -1.5
Maximum: 5.0 10.0 50.0

Mass	Calc. Mass	mDa	PPM	DBE	i-FIT	Norm	Conf(%)	Formula
422.2304	422.2307	-0.3	-0.7	8.5	292.8	1.535	21.54	C24 H31 N O2 F3

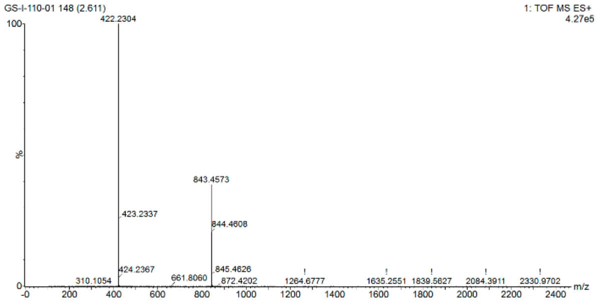

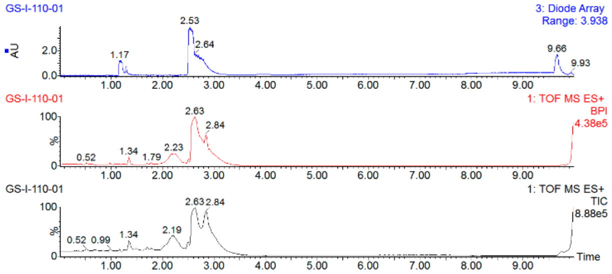

Compound 5e

Single Mass Analysis
Tolerance = 5.0 mDa / DBE: min = -1.5, max = 50.0
Element prediction: Off
Number of isotope peaks used for i-FIT = 3

Monoisotopic Mass, Even Electron Ions
5818 formula(e) evaluated with 39 results within limits (all results (up to 1000) for each mass)
Elements Used:
C: 0-500 H: 0-1000 N: 0-10 O: 0-20 F: 0-10

KDI-VIII-94 169 (2.979)
1: TOF MS ES+

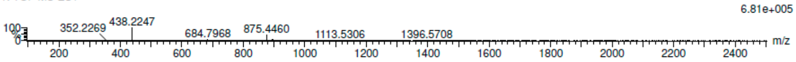

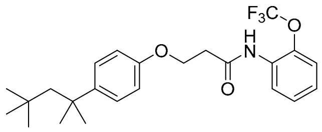

Chemical Formula: $C_{24}H_{30}F_3NO_3$
Exact Mass: 437.2178

Minimum: -1.5
Maximum: 5.0 10.0 50.0

Mass	Calc. Mass	mDa	PPM	DBE	i-FIT	Norm	Conf(%)	Formula
438.2247	438.2256	-0.9	-2.1	8.5	413.9	5.768	0.31	C24 H31 N O3 F3

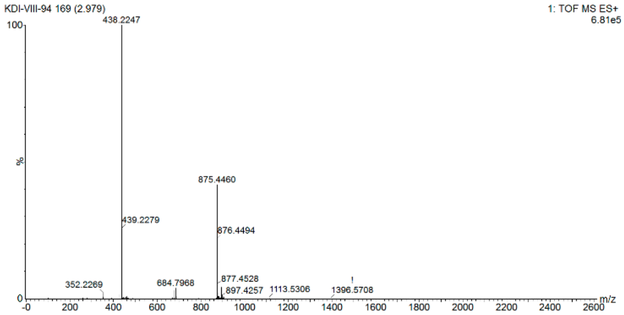

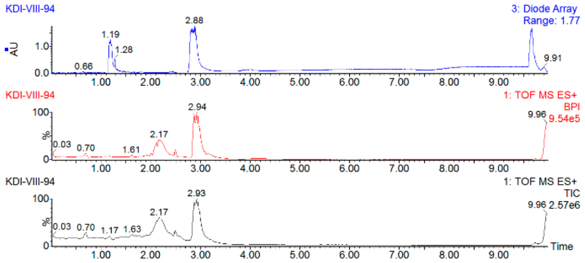

Figures S89. HRMS Analysis

Compound 14

Single Mass Analysis
Tolerance = 5.0 mDa / DBE: min = -1.5, max = 50.0
Element prediction: Off
Number of isotope peaks used for i-FIT = 3

Monoisotopic Mass, Even Electron Ions
5004 formula(e) evaluated with 39 results within limits (all results (up to 1000) for each mass)
Elements Used:
C: 0-500 H: 0-1000 N: 0-10 O: 0-20 F: 0-10
GS-II-41 130 (2.295)
1: TOF MS ES+

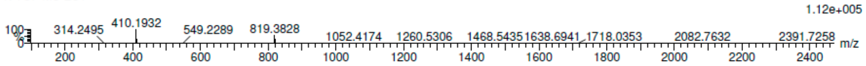

Minimum:				-1.5				
Maximum:		5.0	10.0	50.0				
Mass	Calc. Mass	mDa	PPM	DBE	i-FIT	Norm	Conf(%)	Formula
410.1932	410.1943	-1.1	-2.7	8.5	183.6	2.737	6.47	C22 H27 N O3 F3

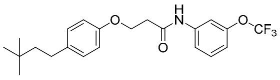

Chemical Formula: $C_{22}H_{26}F_3NO_3$
Exact Mass: 409.1865

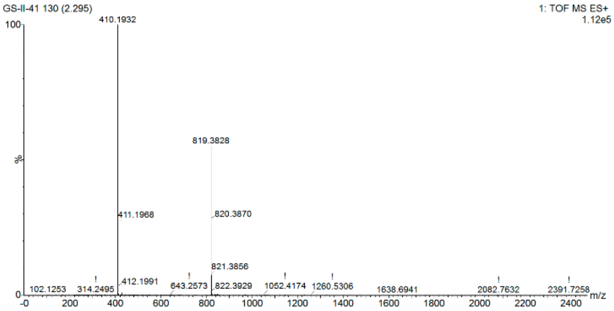

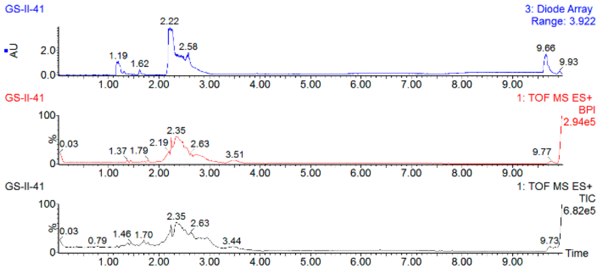

Compound 21

Single Mass Analysis
Tolerance = 5.0 mDa / DBE: min = -1.5, max = 50.0
Element prediction: Off
Number of isotope peaks used for i-FIT = 3

Monoisotopic Mass, Even Electron Ions
5047 formula(e) evaluated with 46 results within limits (all results (up to 1000) for each mass)
Elements Used:
C: 0-500 H: 0-1000 N: 0-10 O: 0-20 F: 0-10
KDI-IX-01 90 (1.592)
1: TOF MS ES+

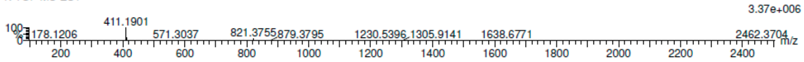

Minimum:				-1.5				
Maximum:		5.0	10.0	50.0				
Mass	Calc. Mass	mDa	PPM	DBE	i-FIT	Norm	Conf(%)	Formula
411.1901	411.1896	0.5	1.2	8.5	739.6	4.507	1.10	C21 H26 N2 O3 F3

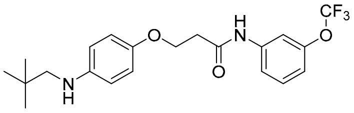

Chemical Formula: $C_{21}H_{25}F_3N_2O_3$
Exact Mass: 410.1817

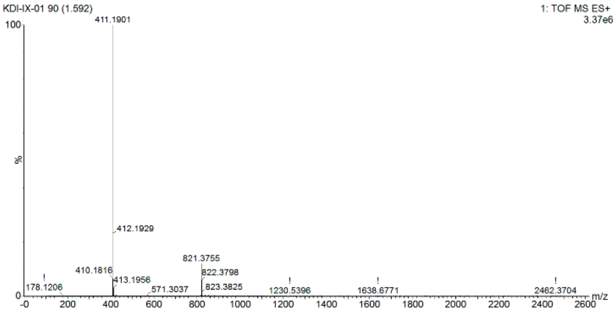

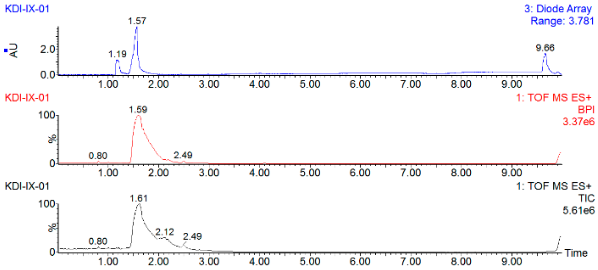

Figures S90. HRMS Analysis

Compound 22

Single Mass Analysis
Tolerance = 5.0 mDa / DBE: min = -1.5, max = 50.0
Element prediction: Off
Number of isotope peaks used for i-FIT = 3

Monoisotopic Mass, Even Electron Ions
5446 formula(e) evaluated with 47 results within limits (all results (up to 1000) for each mass)
Elements Used:
C: 0-500 H: 0-1000 N: 0-10 O: 0-20 F: 0-10
KDI-IX-04 107 (1.892)
1: TOF MS ES+

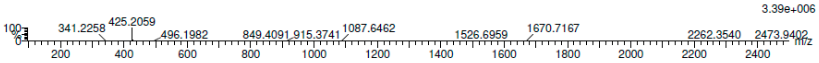

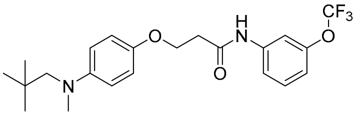

Chemical Formula: $C_{22}H_{27}F_3N_2O_3$
Exact Mass: 424.1974

Minimum:				-1.5				
Maximum:		5.0	10.0	50.0				
Mass	Calc. Mass	mDa	PPM	DBE	i-FIT	Norm	Conf(%)	Formula
425.2059	425.2052	0.7	1.6	8.5	218.8	7.541	0.05	C22 H28 N2 O3 F3

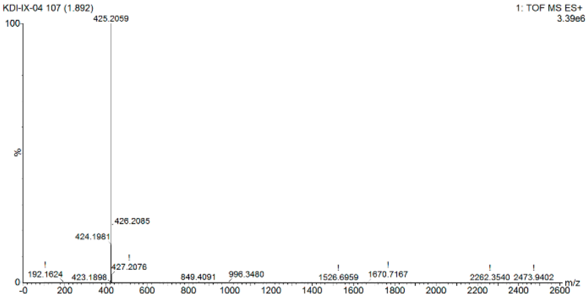

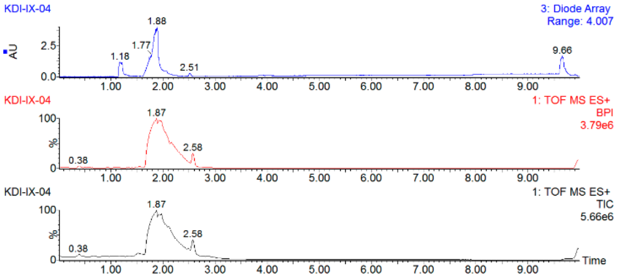

Compound 30

Single Mass Analysis
Tolerance = 5.0 mDa / DBE: min = -1.5, max = 50.0
Element prediction: Off
Number of isotope peaks used for i-FIT = 3

Monoisotopic Mass, Even Electron Ions
5883 formula(e) evaluated with 45 results within limits (all results (up to 1000) for each mass)
Elements Used:
C: 0-500 H: 0-1000 N: 0-10 O: 0-20 F: 0-10

LJH-IV-15 91 (1.609)
1: TOF MS ES+

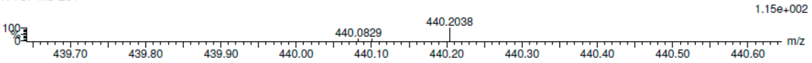

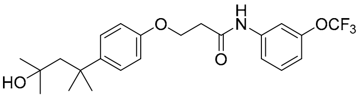

Chemical Formula: $C_{23}H_{28}F_3NO_4$
Exact Mass: 439.1970

Minimum:				-1.5				
Maximum:		5.0	10.0	50.0				
Mass	Calc. Mass	mDa	PPM	DBE	i-FIT	Norm	Conf(%)	Formula
440.2038	440.2049	-1.1	-2.5	8.5	18.6	3.674	2.54	C23 H29 N O4 F3

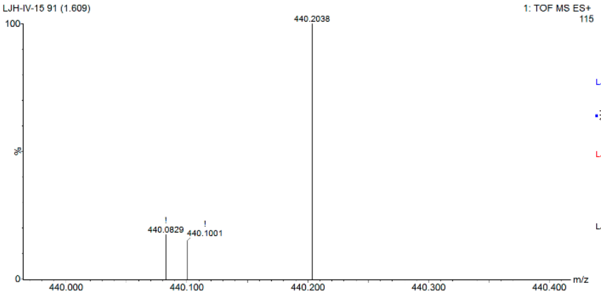

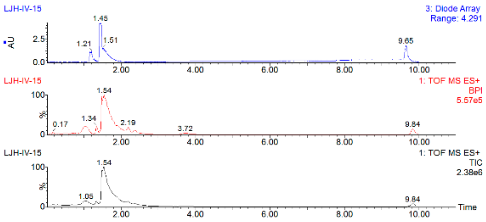

Figures S91. HRMS Analysis

Compound 35a

Single Mass Analysis
Tolerance = 5.0 mDa / DBE: min = -1.5, max = 50.0
Element prediction: Off
Number of isotope peaks used for i-FIT = 3

Monoisotopic Mass, Even Electron Ions
4799 formula(e) evaluated with 52 results within limits (all results (up to 1000) for each mass)
Elements Used:
C: 0-500 H: 0-1000 N: 0-10 O: 0-20 F: 0-10
KDI-VII-45 122 (2.157)
1: TOF MS ES+

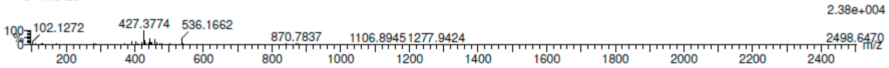

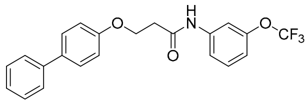

Chemical Formula: $C_{22}H_{18}F_3NO_3$
Exact Mass: 401.1239

Minimum:				-1.5				
Maximum:		5.0	10.0	50.0				
Mass	Calc. Mass	mDa	PPM	DBE	i-FIT	Norm	Conf(%)	Formula
402.1312	402.1317	-0.5	-1.2	12.5	153.2	9.185	0.01	C22 H19 N O3 F3

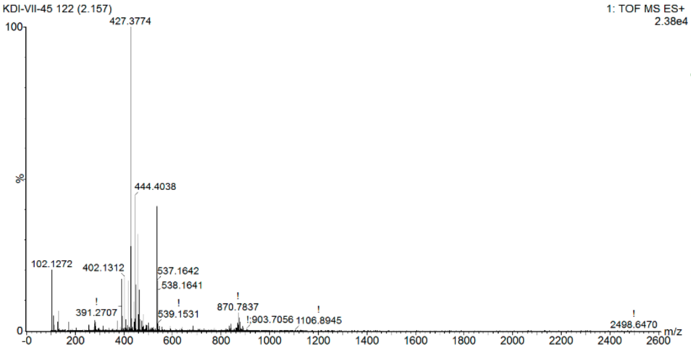

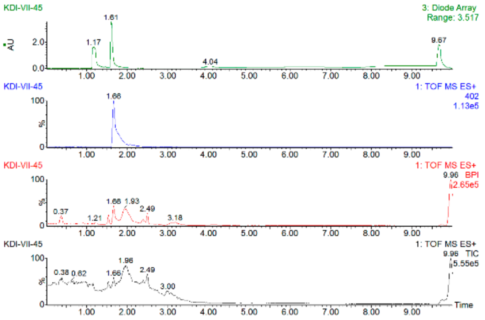

Compound 35b

Single Mass Analysis
Tolerance = 5.0 mDa / DBE: min = -1.5, max = 50.0
Element prediction: Off
Number of isotope peaks used for i-FIT = 3

Monoisotopic Mass, Even Electron Ions
5323 formula(e) evaluated with 60 results within limits (all results (up to 1000) for each mass)
Elements Used:
C: 0-500 H: 0-1000 N: 0-10 O: 0-20 F: 0-10
KDI-VIII-25 93 (1.644)
1: TOF MS ES+

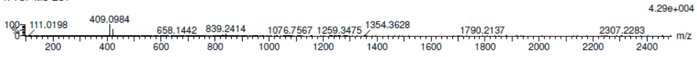

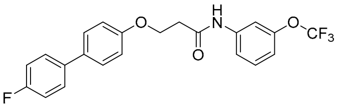

Chemical Formula: $C_{22}H_{17}F_4NO_3$
Exact Mass: 419.1145

Minimum:				-1.5				
Maximum:		5.0	10.0	50.0				
Mass	Calc. Mass	mDa	PPM	DBE	i-FIT	Norm	Conf(%)	Formula
420.1216	420.1223	-0.7	-1.7	12.5	104.3	6.914	0.10	C22 H18 N O3 F4

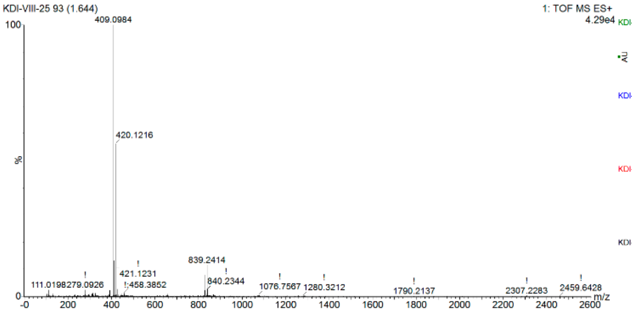

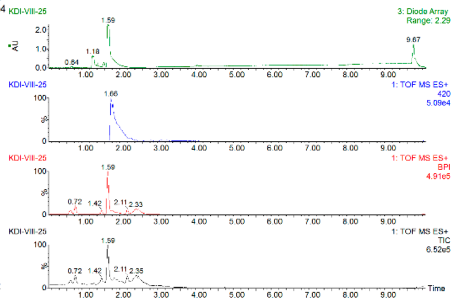

Figures S92. HRMS Analysis

Compound 39a

Single Mass Analysis
Tolerance = 5.0 mDa / DBE: min = -1.5, max = 50.0
Element prediction: Off
Number of isotope peaks used for i-FIT = 3

Monoisotopic Mass, Even Electron Ions
4081 formula(e) evaluated with 50 results within limits (all results (up to 1000) for each mass)
Elements Used:
C: 0-500 H: 0-1000 N: 0-10 O: 0-20 F: 0-10
KDI-VIII-96 90 (1.592)
1: TOF MS ES+

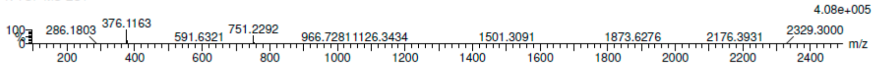

Minimum:				-1.5				
Maximum:		5.0	10.0	50.0				
Mass	Calc. Mass	mDa	PPM	DBE	i-FIT	Norm	Conf(%)	Formula
376.1163	376.1161	0.2	0.5	11.5	319.2	4.121	1.62	C20 H17 N O3 F3

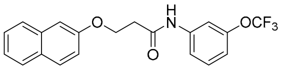

Chemical Formula: $C_{20}H_{16}F_3NO_3$
Exact Mass: 375.1082

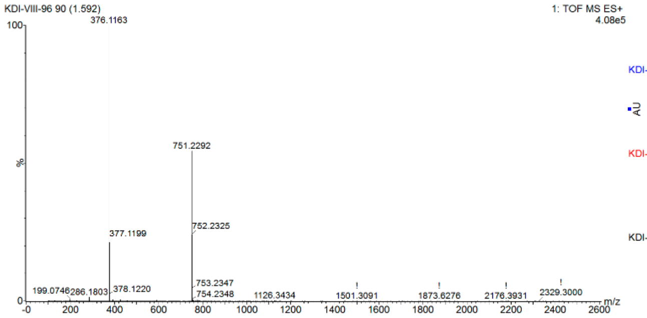

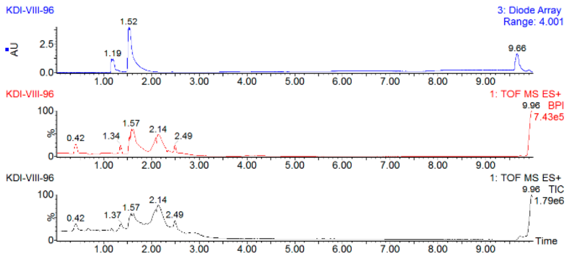

Compound 39b

Single Mass Analysis
Tolerance = 5.0 mDa / DBE: min = -1.5, max = 50.0
Element prediction: Off
Number of isotope peaks used for i-FIT = 3

Monoisotopic Mass, Even Electron Ions
13450 formula(e) evaluated with 150 results within limits (all results (up to 1000) for each mass)
Elements Used:
C: 0-500 H: 0-1000 N: 0-10 O: 0-20 Br: 0-8 F: 0-10
KDI-I-44 108 (1.909)
1: TOF MS ES+

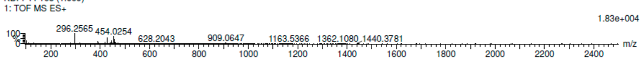

Minimum:				-1.5				
Maximum:		5.0	10.0	50.0				
Mass	Calc. Mass	mDa	PPM	DBE	i-FIT	Norm	Conf(%)	Formula
454.0254	454.0266	-1.2	-2.6	11.5	136.1	4.537	1.07	C20 H16 N O3 Br F3

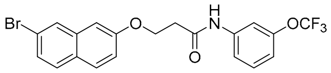

Chemical Formula: $C_{20}H_{15}BrF_3NO_3$
Exact Mass: 453.0187

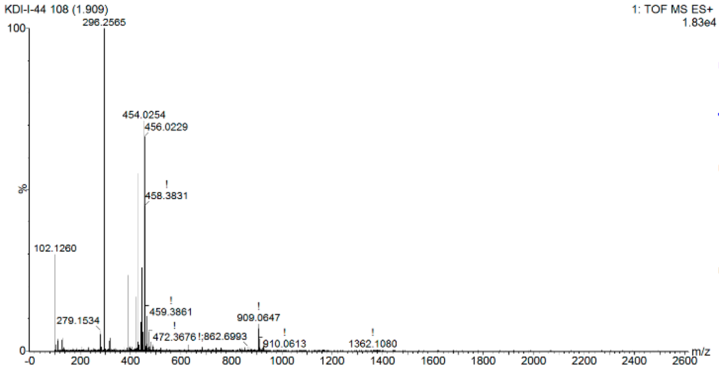

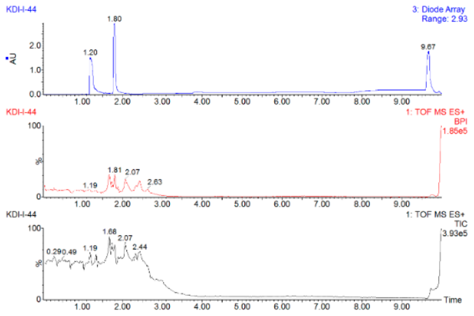

Figures S93. HRMS Analysis

Compound 39c

Single Mass Analysis
Tolerance = 5.0 mDa / DBE: min = -1.5, max = 50.0
Element prediction: Off
Number of isotope peaks used for i-FIT = 3

Monoisotopic Mass, Even Electron Ions
13451 formula(e) evaluated with 148 results within limits (all results (up to 1000) for each mass)
Elements Used:
C: 0-500 H: 0-1000 N: 0-10 O: 0-20 F: 0-10 Br: 0-8

KDI-I-45 107 (1.892)
1: TOF MS ES+

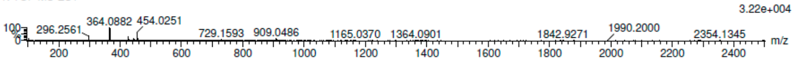

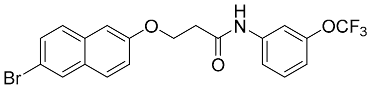

Chemical Formula: $C_{20}H_{15}BrF_3NO_3$
Exact Mass: 453.0187

Mass	Calc. Mass	mDa	PPM	DBE	i-FIT	Norm	Conf(%)	Formula
Minimum:				-1.5				
Maximum:		5.0	10.0	50.0				
454.0251	454.0266	-1.5	-3.3	11.5	166.1	6.917	0.10	C20 H16 N O3 F3 Br

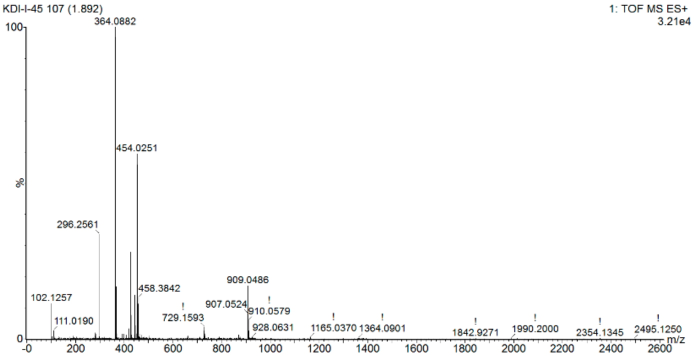

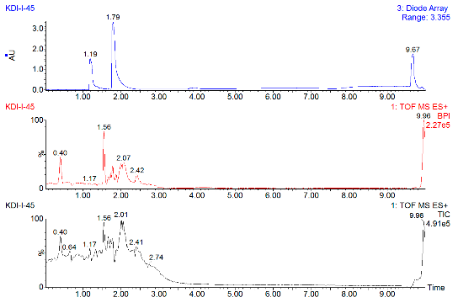

Compound 39d

Single Mass Analysis
Tolerance = 5.0 mDa / DBE: min = -1.5, max = 50.0
Element prediction: Off
Number of isotope peaks used for i-FIT = 3

Monoisotopic Mass, Even Electron Ions
4585 formula(e) evaluated with 56 results within limits (all results (up to 1000) for each mass)
Elements Used:
C: 0-500 H: 0-1000 N: 0-10 O: 0-20 F: 0-10
KDI-II-27 87 (1.541)
1: TOF MS ES+

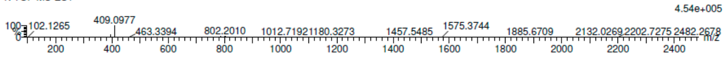

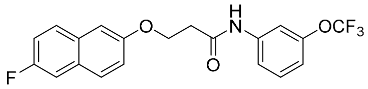

Chemical Formula: $C_{20}H_{15}F_4NO_3$
Exact Mass: 393.0988

Mass	Calc. Mass	mDa	PPM	DBE	i-FIT	Norm	Conf(%)	Formula
Minimum:				-1.5				
Maximum:		5.0	10.0	50.0				
394.1058	394.1066	-0.8	-2.0	11.5	180.8	6.929	0.10	C20 H16 N O3 F4

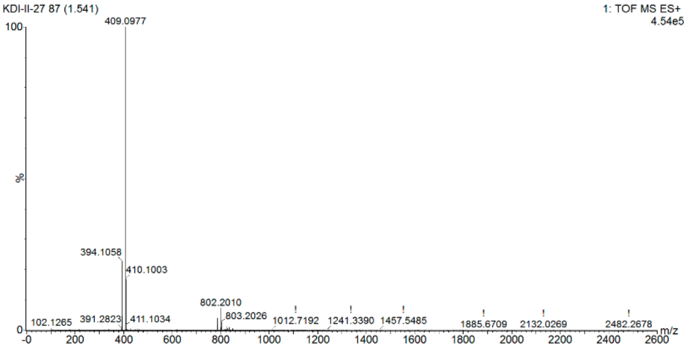

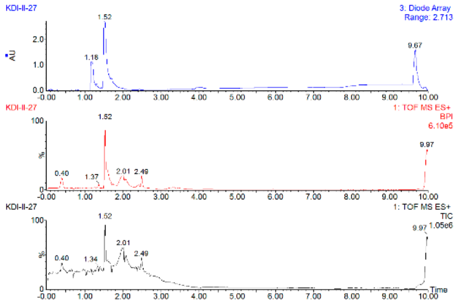

Figures S94. HRMS Analysis

Compound 44a

Single Mass Analysis
Tolerance = 5.0 mDa / DBE: min = -1.5, max = 50.0
Element prediction: Off
Number of isotope peaks used for i-FIT = 3

Monoisotopic Mass, Even Electron Ions
5818 formula(e) evaluated with 40 results within limits (all results (up to 1000) for each mass)
Elements Used:
C: 0-500 H: 0-1000 N: 0-10 O: 0-20 F: 0-10

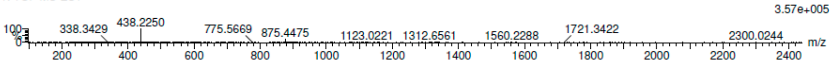

Mass	Calc. Mass	mDa	PPM	DBE	i-FIT	Norm	Conf(%)	Formula
Minimum:				-1.5				
Maximum:		5.0	10.0	50.0				
438.2250	438.2256	-0.6	-1.4	8.5	305.1	6.847	0.11	C24 H31 N O3 F3

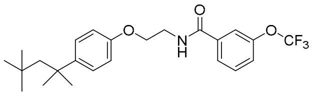

Chemical Formula: $C_{24}H_{30}F_3NO_3$
Exact Mass: 437.2178

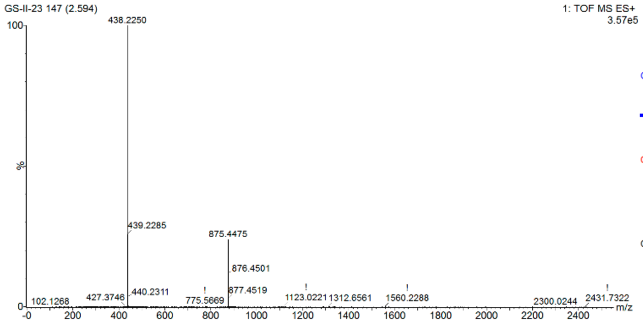

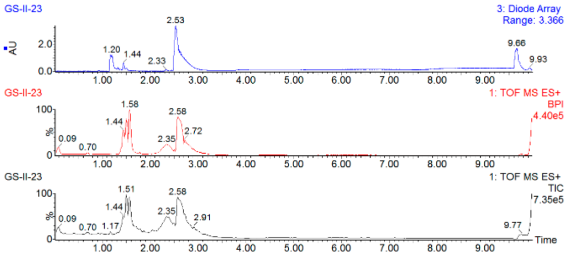

Compound 44b

Single Mass Analysis
Tolerance = 5.0 mDa / DBE: min = -1.5, max = 50.0
Element prediction: Off
Number of isotope peaks used for i-FIT = 3

Monoisotopic Mass, Even Electron Ions
17871 formula(e) evaluated with 116 results within limits (all results (up to 1000) for each mass)
Elements Used:
C: 0-500 H: 0-1000 N: 0-10 O: 0-20 S: 0-2 F: 0-10

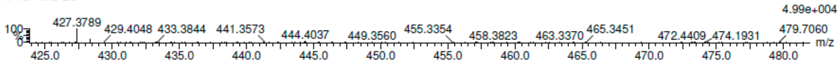

Mass	Calc. Mass	mDa	PPM	DBE	i-FIT	Norm	Conf(%)	Formula
Minimum:				-1.5				
Maximum:		5.0	10.0	50.0				
474.1931	474.1926	0.5	1.1	7.5	58.5	6.031	0.24	C23 H31 N O4 S F3

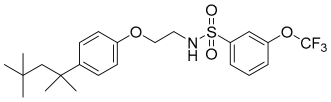

Chemical Formula: $C_{23}H_{30}F_3NO_4S$
Exact Mass: 473.1848

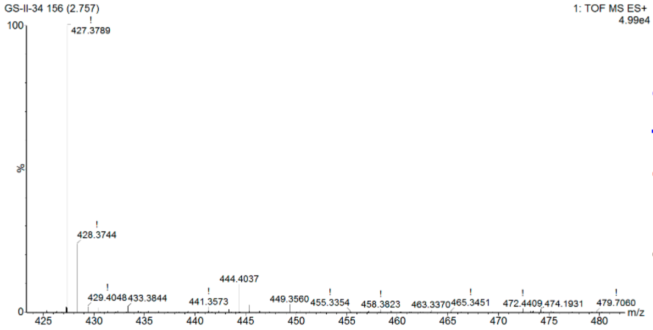

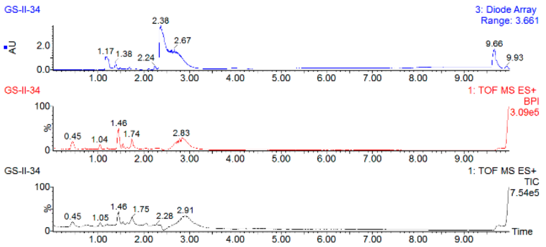

Figures S95. HRMS Analysis

Compound 50

Single Mass Analysis
Tolerance = 5.0 mDa / DBE: min = -1.5, max = 50.0
Element prediction: Off
Number of isotope peaks used for i-FIT = 3

Monoisotopic Mass, Even Electron Ions
7021 formula(e) evaluated with 38 results within limits (all results (up to 1000) for each mass)
Elements Used:
C: 0-500 H: 0-1000 N: 0-10 O: 0-20 F: 0-10

KDI-I-57 162 (2.859)
1: TOF MS ES+

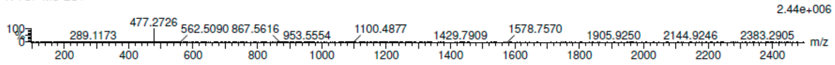

Minimum:				-1.5				
Maximum:		5.0	10.0	50.0				
Mass	Calc. Mass	mDa	PPM	DBE	i-FIT	Norm	Conf(%)	Formula
477.2726	477.2729	-0.3	-0.6	9.5	653.1	6.784	0.11	C27 H36 N2 O2 F3

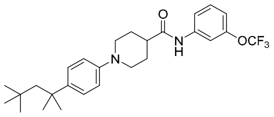

Chemical Formula: $C_{27}H_{35}F_3N_2O_2$
Exact Mass: 476.2651

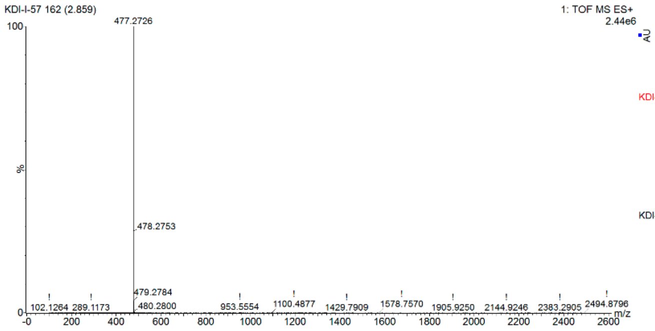

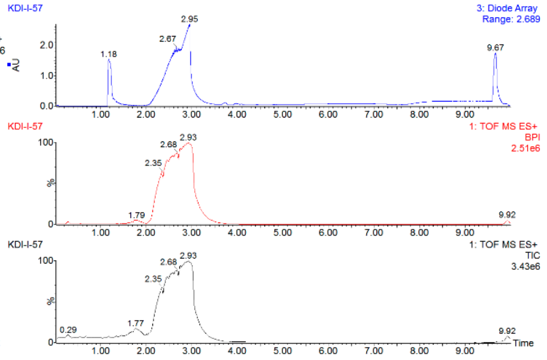

Compound 51

Single Mass Analysis
Tolerance = 5.0 mDa / DBE: min = -1.5, max = 50.0
Element prediction: Off
Number of isotope peaks used for i-FIT = 3

Monoisotopic Mass, Even Electron Ions
6595 formula(e) evaluated with 41 results within limits (all results (up to 1000) for each mass)
Elements Used:
C: 0-500 H: 0-1000 N: 0-10 O: 0-20 F: 0-10

KDI-I-62 565 (9.940)
1: TOF MS ES+

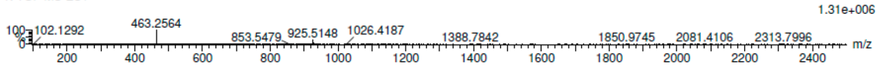

Minimum:				-1.5				
Maximum:		5.0	10.0	50.0				
Mass	Calc. Mass	mDa	PPM	DBE	i-FIT	Norm	Conf(%)	Formula
463.2564	463.2572	-0.8	-1.7	9.5	571.0	5.089	0.62	C26 H34 N2 O2 F3

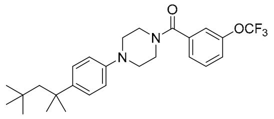

Chemical Formula: $C_{26}H_{33}F_3N_2O_2$
Exact Mass: 462.2494

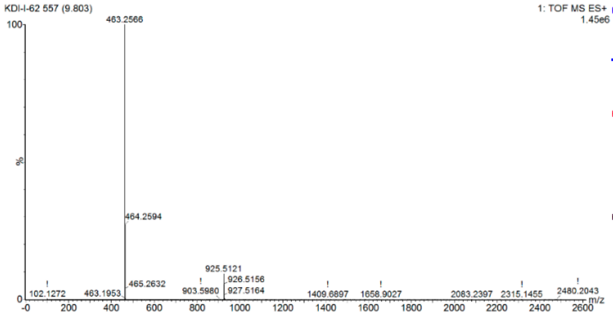

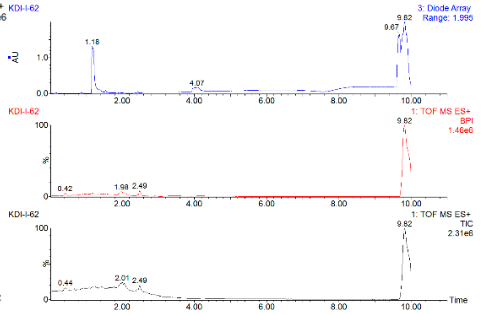

Figures S96. HRMS Analysis

Compound 56

Single Mass Analysis
Tolerance = 5.0 mDa / DBE: min = -1.5, max = 50.0
Element prediction: Off
Number of isotope peaks used for i-FIT = 3

Monoisotopic Mass, Even Electron Ions
5791 formula(e) evaluated with 37 results within limits (all results (up to 1000) for each mass)
Elements Used:
C: 0-500 H: 0-1000 N: 0-10 O: 0-20 F: 0-10
GS-II-46 143 (2.525)
1: TOF MS ES+

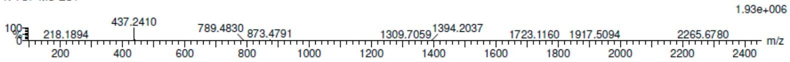

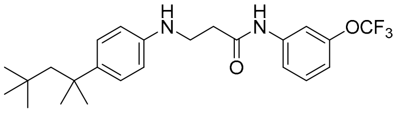

Chemical Formula: $C_{24}H_{31}F_3N_2O_2$
Exact Mass: 436.2338

Minimum:				-1.5				
Maximum:		5.0	10.0	50.0				
Mass	Calc. Mass	mDa	PPM	DBE	i-FIT	Norm	Conf(%)	Formula
437.2410	437.2416	-0.6	-1.4	8.5	627.0	2.829	5.91	C24 H32 N2 O2 F3

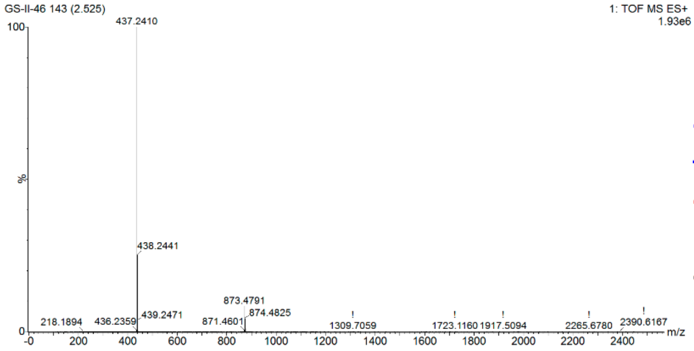

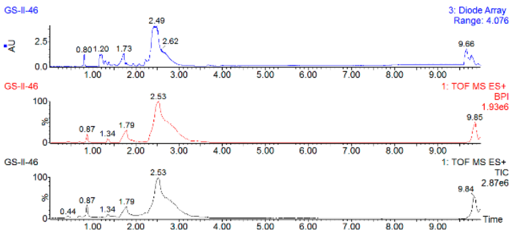

Compound 57a

Single Mass Analysis
Tolerance = 5.0 mDa / DBE: min = -1.5, max = 50.0
Element prediction: Off
Number of isotope peaks used for i-FIT = 3

Monoisotopic Mass, Even Electron Ions
6217 formula(e) evaluated with 36 results within limits (all results (up to 1000) for each mass)
Elements Used:
C: 0-500 H: 0-1000 N: 0-10 O: 0-20 F: 0-10
GS-II-47 184 (3.245)
1: TOF MS ES+

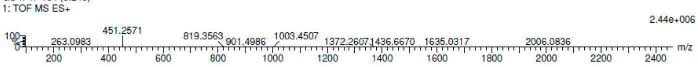

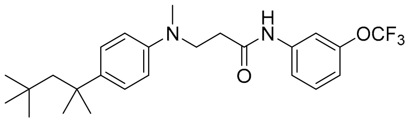

Chemical Formula: $C_{25}H_{33}F_3N_2O_2$
Exact Mass: 450.2494

Minimum:				-1.5				
Maximum:		5.0	10.0	50.0				
Mass	Calc. Mass	mDa	PPM	DBE	i-FIT	Norm	Conf(%)	Formula
451.2571	451.2572	-0.1	-0.2	8.5	671.3	1.565	20.92	C25 H34 N2 O2 F3

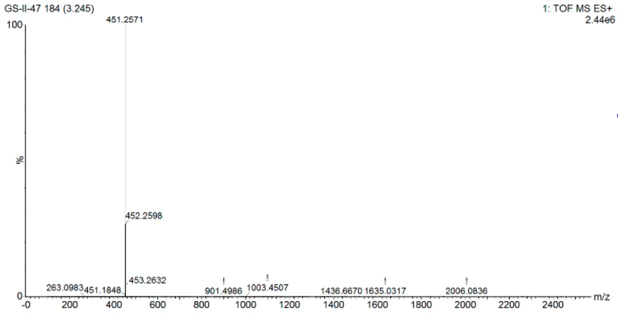

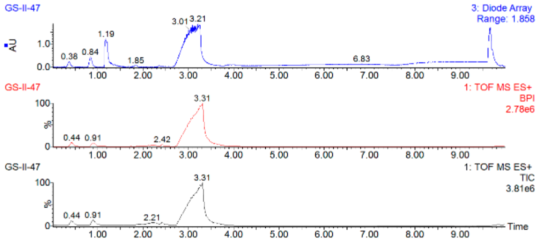

Figures S97. HRMS Analysis

Compound 57b

Single Mass Analysis
Tolerance = 5.0 mDa / DBE: min = -1.5, max = 50.0
Element prediction: Off
Number of isotope peaks used for i-FIT = 3

Monoisotopic Mass, Even Electron Ions
7078 formula(e) evaluated with 34 results within limits (all results (up to 1000) for each mass)
Elements Used:
C: 0-500 H: 0-1000 N: 0-10 O: 0-20 F: 0-10
KDI-VII-87 92 (1.627)
1: TOF MS ES+

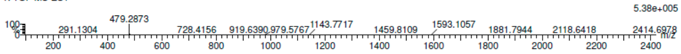

Minimum:				-1.5				
Maximum:		5.0	10.0	50.0				
Mass	Calc. Mass	mDa	PPM	DBE	i-FIT	Norm	Conf(%)	Formula
479.2873	479.2885	-1.2	-2.5	8.5	249.0	6.121	0.22	C27 H38 N2 O2 F3

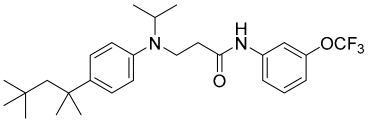

Chemical Formula: $C_{27}H_{37}F_3N_2O_2$
Exact Mass: 478.2807

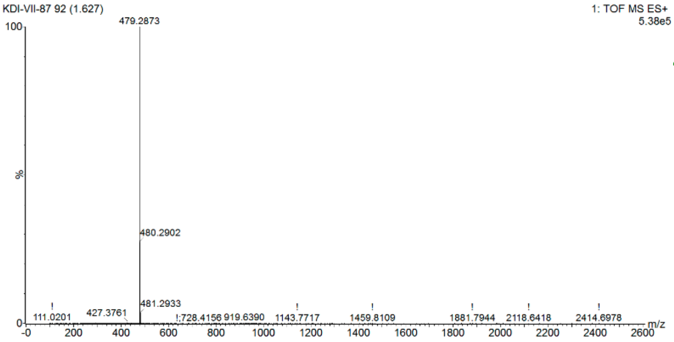

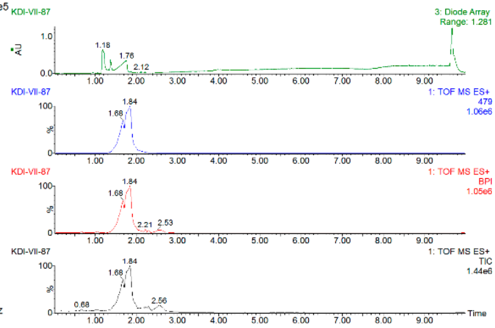

Compound 57c

Single Mass Analysis
Tolerance = 5.0 mDa / DBE: min = -1.5, max = 50.0
Element prediction: Off
Number of isotope peaks used for i-FIT = 3

Monoisotopic Mass, Even Electron Ions
7456 formula(e) evaluated with 42 results within limits (all results (up to 1000) for each mass)
Elements Used:
C: 0-500 H: 0-1000 N: 0-10 O: 0-20 F: 0-10
KDI-VIII-14 179 (3.160)
1: TOF MS ES+

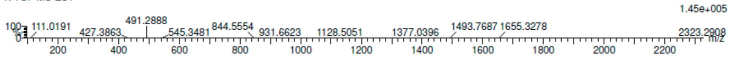

Minimum:				-1.5				
Maximum:		5.0	10.0	50.0				
Mass	Calc. Mass	mDa	PPM	DBE	i-FIT	Norm	Conf(%)	Formula
491.2888	491.2885	0.3	0.6	9.5	165.5	9.495	0.01	C28 H38 N2 O2 F3

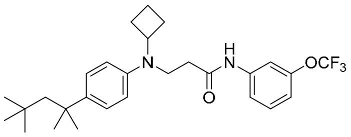

Chemical Formula: $C_{28}H_{37}F_3N_2O_2$
Exact Mass: 490.2807

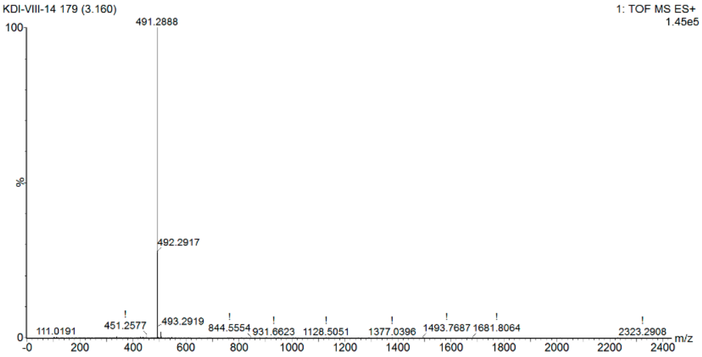

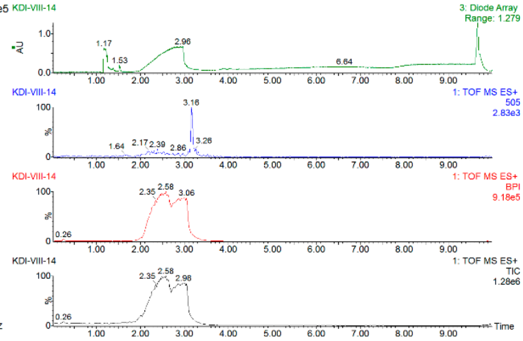

Figures S98. HRMS Analysis

Compound 57d

Single Mass Analysis
Tolerance = 5.0 mDa / DBE: min = -1.5, max = 50.0
Element prediction: Off
Number of isotope peaks used for i-FIT = 3

Monoisotopic Mass, Even Electron Ions
7092 formula(e) evaluated with 48 results within limits (all results (up to 1000) for each mass)
Elements Used:
C: 0-500 H: 0-1000 N: 0-10 O: 0-20 F: 0-10
KDI-I-99 129 (2.277)
1: TOF MS ES+

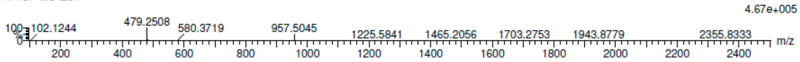

Minimum:				-1.5				
Maximum:		5.0	10.0	50.0				
Mass	Calc. Mass	mDa	PPM	DBE	i-FIT	Norm	Conf(%)	Formula
479.2508	479.2522	-1.4	-2.9	9.5	306.1	7.561	0.05	C26 H34 N2 O3 F3

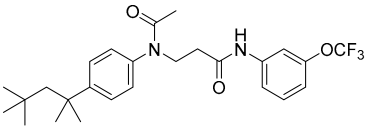

Chemical Formula: $C_{26}H_{33}F_3N_2O_3$
Exact Mass: 478.2443

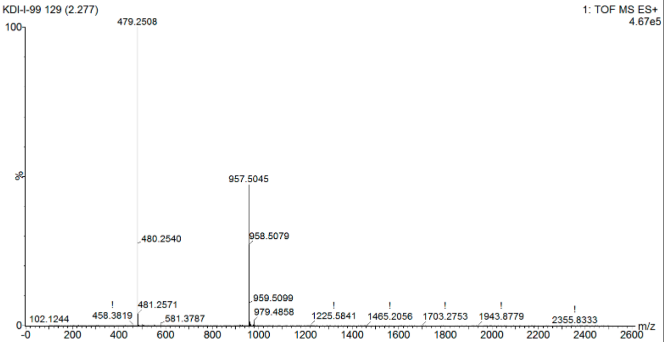

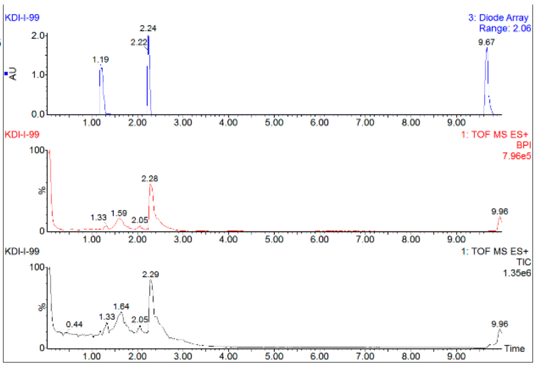

Supplement: Supplementary file 1 [file pharmaceuticals-16-00683-s001.zip › Supporting information_09052023_2357172.pdf]
